# SUMOylation of Arginyl tRNA Synthetase Modulates the *Drosophila* Innate Immune Response

**DOI:** 10.3389/fcell.2021.695630

**Published:** 2021-09-30

**Authors:** Prajna Nayak, Aarti Kejriwal, Girish S. Ratnaparkhi

**Affiliations:** Indian Institute of Science Education and Research (IISER), Pune, India

**Keywords:** MARS complex, NFkB, signaling, CRISPR, Cas9, ArgRS

## Abstract

SUMO conjugation of a substrate protein can modify its activity, localization, interaction or function. A large number of SUMO targets in cells have been identified by Proteomics, but biological roles for SUMO conjugation for most targets remains elusive. The multi-aminoacyl tRNA synthetase complex (MARS) is a sensor and regulator of immune signaling. The proteins of this 1.2 MDa complex are targets of SUMO conjugation, in response to infection. Arginyl tRNA Synthetase (RRS), a member of the sub-complex II of MARS, is one such SUMO conjugation target. The sites for SUMO conjugation are Lys 147 and 383. Replacement of these residues by Arg (RRS^*K147R,K383R*^), creates a SUMO conjugation resistant variant (RRS^*SCR*^). Transgenic *Drosophila* lines for RRS^*WT*^ and RRS^*SCR*^ were generated by expressing these variants in a *RRS loss of function (lof)* animal, using the UAS-Gal4 system. The *RRS*-*lof* line was itself generated using CRISPR/Cas9 genome editing. Expression of both *RRS*^*WT*^ and *RRS*^*SCR*^ rescue the *RRS-lof* lethality. Adult animals expressing *RRS*^*WT*^ and *RRS*^*SCR*^ are compared and contrasted for their response to bacterial infection by gram positive *M. luteus* and gram negative *Ecc15*. We find that *RRS*^*SCR*^, when compared to *RRS*^*WT*^, shows modulation of the transcriptional response, as measured by quantitative 3′ mRNA sequencing. Our study uncovers a possible non-canonical role for SUMOylation of RRS, a member of the MARS complex, in host-defense.

## Introduction

Aminoacyl-tRNA synthetases (ARSs) are ancient, evolutionary conserved enzymes whose primary housekeeping function is to catalyze the aminoacylation of transfer RNAs (tRNAs) (Schimmel and Soll, 1979; Rubio Gomez and Ibba, 2020). In addition to their primary role of charging tRNA, ARSs also have non-canonical, “moonlighting” functions (Guo and Schimmel, 2013; Yao et al., 2014). These secondary functions are driven by modifications to the polypeptide chain by mutations, domain addition or Post-Translational modifiers (PTMs) (Sampath et al., 2004). ARSs are a target of a variety of PTMs, with phosphorylation being studied extensively (Arif et al., 2017). The small ubiquitin-like modifier [SUMO; (Hay, 2005; Geiss-Friedlander and Melchior, 2007)] is one such PTM that targets ARSs. Proteomic studies on a wide range of eukaryotes have suggested (Panse et al., 2004; Golebiowski et al., 2009; Nie et al., 2009; Hendriks and Vertegaal, 2016; Pirone et al., 2017) that at least fourteen of the twenty ARSs are SUMO conjugated (SUMOylated).

In mammals, nine of the tRNA synthetases (Glu-Pro, Ile, Leu, met, Gln, Lys, Arg, and Asp) are part of a ∼1.2 MDa Multi-aminoacyl tRNA Synthetase (MARS) complex, along with three non-ARS components (AIMP1-3) (Khan et al., 2020). In addition to acting as a “depot” or reservoir for tRNA synthetases and facilitating related translational functions, the release of individual components in response to stimulus, both internal and external, regulate the non-canonical functions of these proteins, inclusive of the AIMPs. The released components can be secreted or relocated to a different cellular compartment (Ray and Fox, TIBS, 2007) (Park SG, Kim 2008, PNAS). The MARS complex is now perceived as a hub for many signaling networks within the cell (Park SG, Kim 2008, PNAS). The MARS complex is conserved from insects to mammals, with the *Drosophila* MARS complex (Kerjan et al., 1994; Havrylenko and Mirande, 2015) containing orthologs of the 11 components seen in mammals.

In an experiment to uncover proteins that are SUMO conjugated in response to infection, our laboratory identified 12 ARSs as potential targets using a quantitative proteomics screen (Handu et al., 2015). The study suggested that SUMOylation of ARSs was a response to immune signaling. Using an *in-bacto* SUMO conjugation assay (Nie et al., 2009), we validated a subset of *Drosophila* ARSs as being SUMOylated. Next, we focused our attention on one substrate, namely Arginyl tRNA synthetase (RRS). We determined that K147 and K383 in RRS were the targets of the SUMO machinery and generated transgenic wild-type and SUMO conjugation resistant (SCR) transgenic lines for RRS using a combination of CRISPR Cas9 genome editing and UAS-Gal4 system. A comparison of the transcriptome of *RRS*^*WT*^ versus *RRS*^*SCR*^ adult flies, in response to both gram positive and gram negative infection, led us to suggest that SUMOylation of RRS could modulate the host-defense response in Drosophila.

## Results

### The Multi-Aminoacyl tRNA Synthetase Complex Complex Is a Target for SUMO Machinery

**FIGURE 1:**
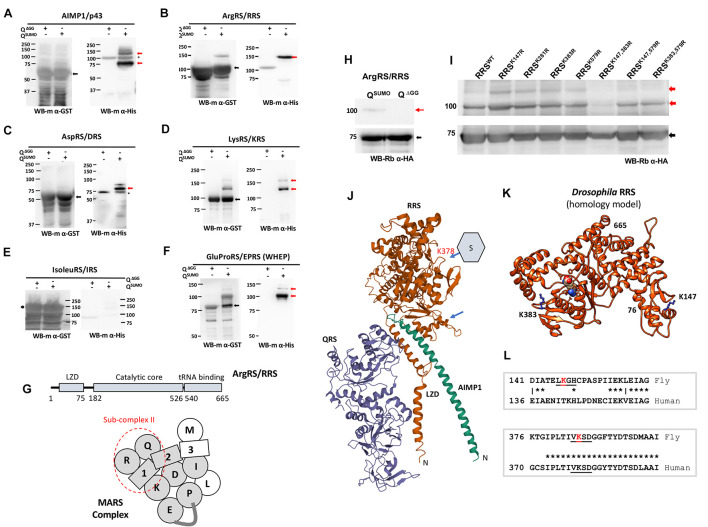
*Drosophila* MARS Complex is a target of SUMO conjugation machinery. **(A–F)** *Validation of SUMOylation by in-bacto SUMO conjugation.* Genes coding for *Drosophila* MARS components were cloned (see section “Materials and Methods”) and tested for SUMO conjugation using the *in-bacto* SUMOylation (Nie et al., 2009). Here, the proteins to be tested are fused with GST and expressed in bacteria along with *Drosophila* SAE1/SAE2, Ubc9 and the matured form of SUMO, SUMO-GG (Nie et al., 2009). AIMP1, RRS, DRS, KRS were SUMO conjugated **(A–D)** while IRS did not show conjugation **(E)**. The WHEP domain of EPRS was expressed to demonstrate SUMOylation of EPRS **(F)**. **(G)** *RRS and the MARS complex. Drosophila* RRS consists of a Leucine zipper domain (LZD), a catalytic core and C-terminal tRNA binding domain. In the MARS schematic, the ARSs are labeled with a single letter code, with the gray shading denoting mass spectrometric or *in-bacto* evidence for SUMO conjugation (Supplementary Figure 1A). RRS is part of sub-complex II (marked with red dashed line), interacting with QRS and AIMP1. **(H)** *RRS is SUMO conjugated*. When co-expressed with *Drosophila* E1, E2 and 6X-His:SUMO, RRS shows a single extra band running ∼20 KDa higher than RRS itself, in Western blots. The band also cross-reacts with an anti-His antibody (data not shown) confirming that it represents a SUMO-conjugated species. The band is not seen when a SUMO(ΔGG) variant, which is unable to conjugate to a substrate, is used. A second, faint band seen in overexposed Western blots suggests that RRS may have a second SUMOylation site. **(I)** *RRS is conjugated at K147 and K383*. Based on predictions of SUMO conjugation sites from JASSA (Beauclair et al., 2015), mutagenesis of four lysines was carried out one at a time. None of the single mutants showed loss of SUMOylation. Amongst double mutants, RRS^*K147R,K383R*^ double mutant was resistant to SUMO conjugation. **(J)** *Schematic of the structure for the human QRS:RRS:AIMP1 complex* (PDB-ID 4R3Z). The SUMOylation sites in the fly RRS were mapped to the human RRS structure, after sequence alignment. The SUMOylation sites were distant from the binding regions of both QRS and AIMP1 and did not appear to interact with any component of MARS, based on current structural models (Khan et al., 2020). The fly K383 equivalent in humans, K378, is in a loop region. (arrow) and is a predicted SUMO conjugation site. **(K)** *Homology model of Drosophila RRS*. A homology model of fly RRS, based on the Arg bound 4Q2T structure as a template. The structure includes residues 76–665 but not the N-terminal LZD (1–75). K383 is in a loop outside the Arg binding site, while K147 is at the end of a helix. **(L)** *SUMO conjugation site is conserved from flies to mammals.* SUMO conjugation sites (K147, K383) for the fly RRS are underlined, with the target Lys marked in red. Based on sequence alignment of fly and human RRS, the K383 site is in an evolutionarily conserved region, while the K147 is not.

Proteomics studies in a host of organisms suggest that members of the MARS Complex are SUMOylation targets (Figure 1G and Supplementary Figure 1; Panse et al., 2004; Tatham et al., 2011; Handu et al., 2015), including studies in *Drosophila* (Handu et al., 2015; Pirone et al., 2017). Handu et al. (2015) specifically enriched proteins that changed their SUMOylation status in response to a broad activation of immune pathways, with ARSs being significant targets. As a first step to validate the targets, we cloned members of the *Drosophila* MARS complex (Lu et al., 2015) into bacterial expression vectors and screened their ability to be SUMOylated in an *in-bacto* system (Nie et al., 2009), which uses *Drosophila* enzymes expressed in bacteria for SUMO conjugation. We find that five ARSs; EPRS, RRS, KRS, DRS, and one AIMP (AIMP1) were modified by SUMO (Figures 1A–F). MRS and LRS could not be expressed while IRS was expressed and not SUMO conjugated. The SUMOylation status for QRS, AIMP2 and 3 was inconclusive due to low protein expression and high background in western blots. Of these we choose RRS as a target to characterize, it being an understudied target showing robust SUMOylation. Prediction of SUMO conjugation sites (Beauclair et al., 2015) in the RRS sequence suggests that RRS has a strong consensus SUMO conjugation motif at K383. Our experimental data suggested that RRS can show upto two SUMO conjugates (Figures 1H,I) and multiple rounds of mutagenesis followed by *in-bacto* SUMOylation led to the finding that a mutant RRS^*K147R,K383R*^ is SUMO conjugation resistant (RRS^*SCR*^) (Figure 1I). RRS is part of subcomplex-II (Figure 1G) in the MARS complex, associating intimately with QRS and AIMP1. Analysis of the crystal structure of sub-complex-II suggests that the equivalent amino acids in the human structure [Figure 1J, 4R3Z, (Fu et al., 2014)] are not part of the protein:protein interface with either QRS or AIMP1. We generated a structural model (Supplementary Model S1) of RRS using the automated SWISS-MODEL server (Waterhouse et al., 2018), using the 4Q2T PDB structure (Kim et al., 2014a) as a homology model and mapped the two conjugation sites onto the fly model (Figure 1K). K147 is part of a low scoring SUMO target motif (L**K**GH), at the end of a predicted helix, in a region that is not conserved (Figure 1L). K383 is part of a high scoring SUMO consensus motif (V**K**SD), in a conserved loop near the Arginine bound active site. The nearest residue which interacts with the bound Arg is F388.

### Generation of a Arginyl tRNA Synthetase Loss of Function Line Using CRISPR Cas9 Genome Editing

**FIGURE 2:**
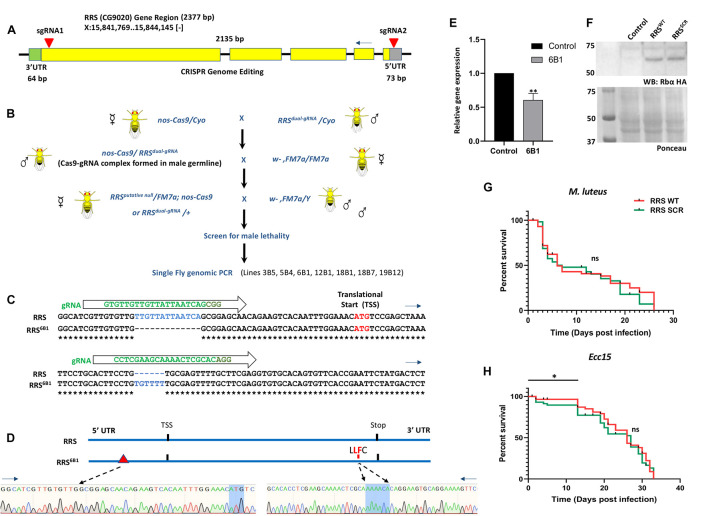
A *RRS* loss of function line generated using CRISPR Cas9 genome editing. **(A)** *Design of the dual guide-RNA for excision of the RRS locus*. The *RRS* gene is located on the X chromosome. It has five exons, which code for a single annotated transcript that spans 2,377 bp. Two gRNAs (inverted red triangles) were designed in the 5′ UTR and 3′ end of the coding region. Our goal was to excise most of the coding region and generate a RRS loss of function line. **(B)** *Excision of the RRS locus to generate a RRS-lof line*. A *UAS-RRS^*dual–gRNA*^* line was generated (see section “Materials and Methods”) and crossed to *nos-Cas9* animals. Sixty lines were balanced over a first chromosome balancer and screened for male lethality. None of the seven male lethals had the expected deletion in the *RRS* locus, based on PCR. **(C)** *RRS^6B1^ lines generated by disruption of the* 5′UTR. Genomic DNA Sequencing of the 6B1 line in the *RRS* genomic region reveals deletions/insertions in the RRS locus, at the gRNA binding site(s). A 13 bp deletion is seen in the 5′UTR and a 6 bp insertion near the 3′ end of the coding region. **(D)** *Schematic of the mutations in the RRS^6B1^ line*. Schematic showing the deletions near the translation start site and insertion in the coding region near the translational stop site. DNA sequences for each perturbation are also shown. **(E)** *RRS^6B1^ line shows lower transcript levels of RRS as compared to wildtype. RRS^6B1^* shows 40% reduction in transcript levels of RRS as compared to wildtype Control. Values on the Y-axis depict the fold change normalized to the house-keeping gene *rp49*. Values shown are Mean ± SEM. *N* = 3, *n* (larvae) = 25. Statistical analysis by Unpaired *t*-test. *p < 0.05 and **p < 0.01. **(F)** *Rescue of RRS^6B1^ by ectopic expression of RRS using UAS-Gal4 system.* Both *RRS*^*WT*^ and *RRS*^*SCR*^ lines show approximately equal expression of RRS, when probed using an anti-HA antibody. Ponceau staining on the same blot us used to show equal loading. **(G,H)** *Survival plots for RRS^*WT*^* and *RRS^*SCR*^ upon M. luteus and Ecc15 infection.* Log rank (Mantel cox) survival plot using Kalpan-Meier and Gehan-Wilcoxon tests, suggests that *RRS*^*WT*^ and *RRS*^*SCR*^ do not show a significant difference in lifespan post infection with *M. luteus.* However, post *Ecc15* infection *RRS*^*SCR*^ shows a significant (*p < 0.05) decrease in survival as compared to *RRS*^*WT*^ in the initial stages (0–15 days).

The UAS-Gal4 system is an ideal system to express RRS^*WT*^ and RRS^*SCR*^ in a RRS loss of function (*lof*) background. Since such a *lof* line is not available, as a first step we used CRISPR Cas9 genome editing to generate the same. A transgenic dual-guide RNA line (*UAS-RRS^*dual–gRNA*^*) was created (See section “Materials and Methods”) to express *gRNA* that would recognize the 5′ UTR and 3′ end of the coding region of the RRS gene (inverted red triangles, Figure 2A), near the translation start and stop sites. Our goal was to remove a major portion of the coding region to create a Δ*RRS* animal. The *UAS-RRS^*dual–gRNA*^* line was crossed to a *nos-Cas9* animal (Figure 2B) and sixty lines stabilized by balancing the putative lof’s over an X chromosome balancer, *FM7i* where the balancer chromosome expresses GFP. Of these lines, seven were male lethal, which was indicative of a successful excision of the *RRS* locus, since the absence of the *RRS* on the X chromosome would lead to lethality. Single fly genomic PCRs were conducted on these lines, but the genomic PCR products did not show the expected 2.1 *kb* deletion that would be a consequence of removal of the RRS genomic region. To probe the observed male lethality, we sequenced the genomic region of two lines 6B1 and 18B1. To our surprise, we found that even though the coding region was not deleted, the gRNA activity caused changes to the sequence of the wild-type genome in the sites targeted by both gRNA (Figures 2C,D), and these modifications presumably led to the generation of variant allele(s). *RRS*^6B1^ has a 13 bp deletion in the 5′UTR region (Figures 2C,D and Supplementary Figure 2A), while in the case of *RRS*^18B1^, there appeared to be a 11 bp insertion in the same region (Supplementary Figure 2C). In both cases, the 5′UTR is disrupted (Figures 2C,D and Supplementary Figures 2A,B). The 5′UTR serves as the entry point for the ribosome during translation and can adopt elaborate RNA secondary and tertiary structures that may regulate translation initiation (Curran and Weiss, 2016; Leppek et al., 2018). To test the stability and/or expression of the transcripts, we measured mRNA levels using quantitative real-time PCR (qRT-PCR) in 1st Instar larvae. Both *RRS* alleles die during IInd instar larval stages, with embryonic survival till 1st Instar presumably driven by maternal *RRS*. *RRS*^6B1^ homozygous larvae, identified by their lack of GFP fluorescence, show 40% reduction in *RRS* transcripts as compared to *wt* (Figure 2E), with similar results for *RRS*^18B1^ (data not shown). We believe that maternal RNA still perdures at this stage, and reduces as the animals transit to the 2nd Instar. The transcript levels measured are thus a sum of maternal and zygotic RNA.

Sequence changes in the coding region were also seen in both lines (Supplementary Figures 2A–C). For *RRS*^6B1^, a 6 bp insert would lead to incorporation of a Leu and Phe (Figures 2C,D) (Supplementary Figure 2A) in positions 604 and 605, within the RRS sequence; For *RRS*^18B1^, the sequence corresponding to the C-terminal domain could not be elucidated in spite of multiple sequencing attempts (Supplementary Figure 2C). For *RRS*^6B1^, the insertion may perturb the structure of the C-terminal tRNA binding domain. One possible scenario is the disruption of the predicted (Craig and Dombkowski, 2013) C515:C604 disulfide bond (Supplementary Figure 2B), in the *Drosophila* structural model (Suppl. Model S1), which could lead to significant destabilization of RRS^6B1^ and lower its functionality. The *RRS*^6B1^ line with defined mutations in the 5′ UTR and coding region, and with homozygotes dying in the 1st to 2nd Instar transition was used for all further experiments. The 6B1 line is in all probability a hypomorphic, *lof* allele of RRS. The *RRS^6B1^/* + and *RRS^18B1^/* + lines are haplo-sufficient, showing normal lifespan at 25 and 29°C (Supplementary Figure 2D) and do not show any embryonic or larval lethality.

### Generation of a Transgenic RRS^*SCR*^ Line

The successful generation of the lof *RRS*^6B1^ line meant that the UAS-Gal4 system could be used to rescue the larval lethality. For this, *RRS-WT* and *RRS-SCR* sequences were cloned into a UAS vector (see section “Materials and Methods”) and *UAS-RRS^*WT*^* and *UAS-RRS^*SCR*^* lines were created on the IIIrd chromosome. *Actin-Gal4*; *UAS-RRS^*WT*^* and *Actin-Gal4*; *UAS-RRS^*SCR*^* lines were balanced and crossed to *RRS^6B1^/FM7i* females. Both these lines could rescue the lethality of the Δ*RRS* male in the F1 generation, with the lines of the genotype, *RRS^6B1^; Actin-Gal4; UAS-RRS^*WT*^* (referred to as *RRS*^*WT*^) and *RRS^6B1^; Actin-Gal4; UAS-RRS^*SCR*^* (referred to as *RRS*^*SCR*^) being used for further experiments. Similar rescue was seen when a *Ubiquitin-Gal4* was used instead of *Actin-Gal4*. Both the “rescued” lines were homozygous viable, had a normal lifespan, suggesting that the SCR allele was functionally equivalent to the WT in terms of its canonical function. Western blots of adult males, rescued by expression of *UAS-RRS^*SCR*^*, showed equal expression of RRS, when compared to *UAS-RRS^*WT*^* (Figure 2F).

*Drosophila* reacts to immune challenge under laboratory conditions with a characteristic transcriptional upregulation and downregulation of defense genes. Infection with gram positive *Micrococcus luteus* (*M. luteus*) and gram negative *Erwinia carotovora carotovora (Ecc15)* were used to trigger the host-defense response. We measured the lifespan of *RRS*^*WT*^ and *RRS*^*SCR*^ animals post-infection. We find that there is no significant difference in lifespan for *M. luteus* infections, while for *Ecc15*, there is an increase in lethality for *RRS*^*SCR*^, for younger animals (1–15 day), while not for older animals (20 day) (Figures 2G,H).

### Transcriptomics of Immune Challenged, RRS^*WT*^ and RRS^*SCR*^ Transgenic Animals

In order to uncover the role of SUMO conjugation in host-defense, we infected 7–8 day old adult flies with *M. luteus* and *Ecc15* and measured transcript levels in both *RRS^*WT*^ and RRS^*SCR*^* using quantitative 3′ RNA sequencing (QuantSeq; see section “Materials and Methods”).

**FIGURE 3:**
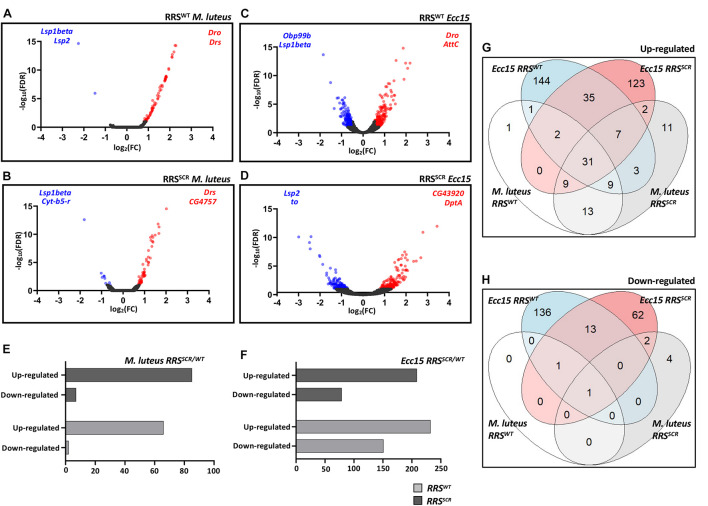
*RRS*^*WT*^ and *RRS*^*SCR*^ show a robust immune response to bacterial infection. **(A–D)** *Volcano plot(s) for The differentially expressed genes*. log_2_(FC) for each gene is plotted against its –log_10_(FDR) value to display differentially expressed genes upon infection as compared to the baseline. Red and blue dots represent the genes which are significantly differentially expressed with log_2_(FC) of >0.55 and <–0.55, respectively, with *p-value* < 0.05 [–log_10_(FDR) of > 2] whereas black dots represent the genes which are uniformly expressed. Representative differentially expressed genes are mentioned on the right (upregulated) and left hand (downregulated) corner of each plot. The time point for *M. luteus* infection is 22 h and for *Ecc15*, it is 12 h. **(E,F)** *Total Number of transcripts upregulated and downregulated in response to infection*. Genes modulated by infection by *M. luteus* **(E)** and *Ecc15* **(F)**, for both *RRS*^*WT*^ and *RRS*^*SCR*^. **(G,H)** *Differential expression of genes*. Venn diagram showing sub-division of upregulated **(G)** and downregulated **(H)** genes for experiments conducted, as defined earlier.

Infection with the bacteria gave a robust immune response (Figures 3A–D and Supplementary Figures 3A–D). Gene Ontology analysis of the modulated genes revealed immune responsive genes associated with Gram Positive and Gram Negative infection for both common and differentially expressed genes, as expected by Toll/NFκB and Immune Deficient (Imd)/NFκB pathway activation (De Gregorio et al., 2002; Supplementary Figures 4A,B). For *RRS*^*WT*^ flies, infection by *M. luteus* led to an upregulation of 66 genes and a downregulation of 2 genes, 22 h post infection. As expected, targets of the Toll pathway such as *drosomycin* (*Drs*) and *metchnikowin* (*Mtk*) were upregulated (Figure 3E and Supplementary Table 1). For the *RRS*^*SCR*^ files, 85 genes were upregulated and 7 genes were downregulated. In a similar vein, infection by *Ecc15* led to 232 upregulated and 151 downregulated in *RRS*^*WT*^ and 209 upregulated and 79 downregulated in the *RRS*^*SCR*^ (Figure 3F and Supplementary Table 2). As expected, targets of the Imd pathway were strongly modulated. In order to examine the extent of overlap among upregulated and downregulated genes between different data sets, Venn diagrams were drawn (Figures 3G,H). A majority of the genes were uniquely expressed among the data sets. Uniquely differentially expressed genes are listed in Supplementary Table 3. Common genes between *RRS*^*WT*^ and *RRS*^*SCR*^ for each infection category were used for further analysis. At basal level, before infection, *RRS*^*WT*^ and *RRS*^*SCR*^ showed minor differences in their transcriptome (Supplementary Tables 4, 5).

### Modulation of the Immune Transcriptome in *RRS*^*SCR*^ Transgenics

**FIGURE 4:**
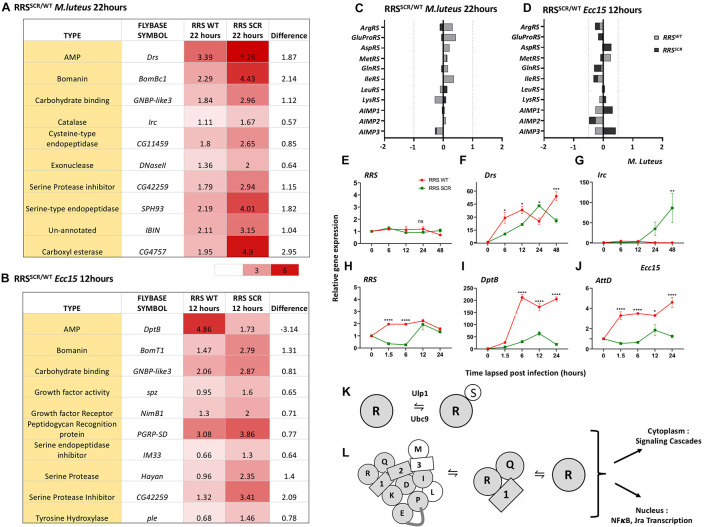
RRS^*WT*^ and RRS^*SCR*^ show differential expression of immune target genes. **(A,B)**. *Tabulation of differentially expressed genes*. Representative genes with differential expression for *RRS*^*SCR*^ for *M. luteus* **(A)** and *Ecc15* **(B)** infections. For *M. luteus*, there is moderate upregulation for most genes, while for *Ecc15*, metabolic genes are both up and down regulated. **(C,D)** *Transcriptome changes for MARS Complex genes*. On infection by *M. luteus* **(C)** and *Ecc15* **(D)**, the changes in transcript levels for genes that code for proteins in the MARS complex is well below the significance cut-off of 0.5 log_2_(FC). **(E–G)** *Expression of RRS, Toll pathway target gene Drosomycin and Immune regulated catalase (Irc) in RRS^*WT*^* and *RRS^*SCR*^ upon M. luteus infection across 0–48 h.* Values on the Y-axis depict the fold change normalized to the house-keeping gene *rp49*. Values shown are Mean ± SEM. *N* = 3, *n* = 5. Statistical analysis by two-way ANOVA followed by Tukey’s multiple comparison test. *p < 0.05, **p < 0.01, ***p < 0.001, and ****p < 0.0001. **(H–J)** *Expression of RRS, Imd pathway target genes Diptericin B and Attacin D in RRS^*WT*^* and *RRS^*SCR*^ upon Ecc15 infection across 0–24 h.* Values on the Y-axis depict the fold change normalized to the house-keeping gene *rp49*. Values shown are Mean ± SEM. *N* = 3, *n* = 5. Statistical analysis by two-way ANOVA followed by Tukey’s multiple comparison test. *p < 0.05, **p < 0.01, ***p < 0.001, and ****p < 0.0001. **(K)** *SUMO Conjugation of RRS*. RRS is a target for the cellular SUMO conjugation machinery. A small fraction of RRS is SUMO conjugated and is in equilibrium with non-conjugated RRS. **(L)** *Model for immune regulation by RRS*. RRS may influence the immune response either as part of the MARS complex, or as part of the AIMP1:RRS:QRS complex or as free RRS. In a SUMO-conjugated state, RRS may influence signaling cascades, interacting with partners containing SIM sites and modify their function. These influences can be either cytoplasmic or nuclear.

Next, we compared the change in immune transcriptome for *RRS*^*SCR*^ with reference to *RRS*^*WT*^ (Figures 4A,B and Supplementary Figures 5, 6). In case of *M. luteus* infection, a total of 22 immune responsive genes including AMPs, Bomanins, Serine hydrolases and genes involved in ROS production were significantly differentially up-regulated (Supplementary Table 1 and Supplementary Figures 5A, 6A) in *RRS*^*SCR*^. Both *Drosomycin (Drs)* and *Bomanin Bicipital 1*(BomBc1) are upregulated 5–6 fold in *RRS*^*SCR*^, while other AMP genes (Figure 4A) were not strongly or significantly upregulated.

In case of *Ecc15* infection, the trends were stronger. A total of 28 genes showed enhanced upregulation and 13 genes showed enhanced repression in *RRS^*SCR*^.* Genes involved in metabolism, such as hydrolases, esterases, non-coding RNA and AMP genes were modulated. Amongst the strongly expressed genes (Figure 4B, Supplementary Table 2, and Supplementary Figures 5B, 6B) were immune responsive genes involved in gram-negative bacterial recognition (*PGRP-LB, PGRP-LF, and PGRP-SD)*, and melanization (*Hayan, Pale, and Punch)*. Genes involved in oxido-reductase pathways like *Sodh 1* and Larval serum proteins *Lsp1, Lsp2* were repressed. For the AMP genes (Figure 4B), *CecA1* and *AttD* were upregulated 2–4 fold, while *AttC, AttA*, and *DptB* downregulated 3–9 fold. We also looked at the transcriptional changes in the genes of the MARS complex. For both *M. luteus* and for *Ecc15*, the transcriptional changes on infection were minimal, with none of the transcript levels crossing our cut-off of significance, 0.55 log_2_(FC) (Figures 4C,D).

Next, we validated the QuantSeq data by qRT-PCR for a few targets at time points ranging from 0–48 h. For *M. luteus*, *RRS* levels did not change significantly from 0–48 h (Figure 4E), while *Drs* levels, though significant, showed similar trends over 48 h (Figure 4F). *Irc* levels were distinctly higher in *RRS*^*SCR*^ animals at later time points (Figure 4G). For Ecc15, RRS transcript levels were different, showing fivefold decrease in *RRS*^*SCR*^ animals, for the time-points 1.5 and 6 h (Figure 4H). *DptB* and *AttD* transcripts are significantly lower in the case of *RRS*^*SCR*^ animals (Figures 4I,J).

## Discussion

The MARS Complex has been implicated as a sensor and regulator of the immune response (Guo and Schimmel, 2013; Kim et al., 2014b; Arif et al., 2018; Nie et al., 2019). Mutations and mis-regulation of MARS function can lead to immune disease (Lee et al., 2018; Nie et al., 2019). In the best studied mechanistic example, in response to infection and release of IFN-γ, EPRS dissociates from the MARS Complex (Sampath et al., 2004). The dissociation is triggered by phosphorylation of the WHEP domain. EPRS now associates with L13a, NSAP1 and GAPDH to form a “GAIT complex” which can now bind to a GAIT RNA element. The GAIT-RNA element (interferon-gamma-activated inhibitor of translation) (Sampath et al., 2003; Marquez-Jurado et al., 2015) is present in UTRs of mRNA transcripts and binding leads to a block of translation of the transcript.

Roles for RRS in the immune response are unknown. In terms of disease, mutations in RRS have been implicated in neuronal hypomyelination with severe spasticity and nystagmus (Antonellis and Green, 2008; Wolf et al., 2014). Autoantibodies against ARSs were found in anti-synthetase syndrome (ASSD), suggesting that ARSs are likely to be involved in the development and progression of autoimmune disease. In *Drosophila*, RRS is not studied in any physiological context.

How then does RRS modulate transcription of defense genes? In mammals, the MARS complex itself is believed to be a cytoplasmic complex, though a few studies suggest nuclear localization (Wolfe et al., 2003; Cui et al., 2020). RRS could be available in at least three species, one as a free, unbound entity, second as a complex with AIMP1 and QRS and finally as part of the MARS Complex (Figure 4L). Deletion of the RRS LZD leads to its dissociation of the MARS complex, but this does not affect charging (Cui et al., 2020). Interestingly, the nuclear fraction of MARS decreases when cells contain RRS (ΔLZD). In its dissociated state, RRS’s canonical functions are unaffected, but developmental genes such as homeobox and forkhead box genes are modulated (Cui et al., 2020).

Each of the RRS species could exist in a SUMO conjugated or unconjugated state (Figure 4K). These species can ultimately regulate gene expression either by influencing signaling pathways in the cytoplasm or by affecting the transcription of the nuclear localized NFκBs. SUMOylation could affect the stability or interaction with other proteins. RRS lacks a nuclear localization signal (NLS), as does *Drosophila* SUMO. Transport to the nucleus would require RRS to be part of a complex that includes a NLS, for example the AIMP1:RRS:QRS complex, as AIMP1 may travel to the nucleus (Lee et al., 2008; Park et al., 2010). AIMP1 in mammals is a precursor for EMAPII which can trigger an inflammatory response (Lee et al., 2019) and a similar mechanism may exist in flies. Other possible mechanisms include modulation of NFκB (Ko et al., 2001) by regulation of secretion of AIMP1 or by regulation of Jun signaling (Park et al., 2002), which in turn can regulate the immune response.

In Summary, RRS is SUMO conjugated and SUMOylation appears to modulate, indirectly the transcriptional host-defense response. The mechanisms underlying these phenomena are currently unknown.

## Materials and Methods

### SUMO Conjugation Assay

SUMOylation of constituents of the MARS complex was tested by expressing the target/substrate protein simultaneously with the *Drosophila* SUMO cycle components based on a published protocol (Nie et al., 2019). Target proteins from the *Drosophila* Gold cDNA collection, procured from the *Drosophila* Genome Resource Center (DGRC), Bloomington, Indiana were sub-cloned into *pGEX-4T1* (Promega) and *pET-45b*, and subsequently sequenced for validation. For visualization of SUMO conjugation, bacterial lysates were affinity purified using Glutathione-Agarose beads (Invitrogen) or Ni NTA-Agarose beads (Qiagen), run on an SDS-PAGE gel and monitored using mouse anti-GST antibody (sc53909, 1:5,000; Santa-Cruz-Biotechnology), Rabbit anti-HA antibody (DW2, 1:3,000; Millipore) and mouse anti-6X-His antibody (H1029, 1:1,000; SIGMA) using Western blotting. The SUMO conjugated forms appear as bands of a higher molecular weight.

### SUMO-Binding-Motif and SIM-Motif Prediction

Putative SUMO acceptor lysines and SIM-motifs of all the MARS complex components of *Drosophila* were predicted *in silico*, using Joined Advanced SUMOylation and Sim motif Analyzer (JASSA) tool with a threshold cut-off criteria set at “high” (Beauclair et al., 2015).

### Identification of Evolutionarily Conserved SUMO Target Lysine Residues *in silico*

FASTA sequences of RRS for model organisms belonging to different eukaryotic groups were procured from the Uniprot protein database. Multiple sequence alignment (MSA) was done on the basis of homology extension using PSI-COFFEE (Chang et al., 2012). SUMO acceptor lysines were compared across different representative organisms, post-alignment.

### Homology Model for *Drosophila* Arginyl tRNA Synthetase

The automated SWISS-MODEL server (Waterhouse et al., 2018) was used to generate a structural models (RRS^*WT*^, RRS^6B1^) using default parameters. The human 4Q2T PDB structure (Kim et al., 2014a), solved at a resolution of 2.4 Å, containing a bound Arginine at the active site was used as a template.

### Generation of Arginyl tRNA Synthetase Loss of Function Lines Using CRISPR Cas9 Technology

CRISPR Cas9 technology was employed to generate RRS loss of function fly lines. Single guide (sg)-RNAs targeting the RRS coding region in the 5′UTR and Exon-5 were designed using CRISPR Optimal Target Finder [COTF; (Gratz et al., 2014)], a web tool for identifying CRISPR target sites and evaluating their specificity. The RRS gene region was sequenced prior to the experiment to designing the gRNAs to account for SNPs at the sgRNA target sites. The sgRNAs were cloned into the *pU6-Bbs*I*-chiRNA* (Addgene # 45946) plasmid, which was then docked into *y^1^ v^1^; P{CaryP}attP40 Drosophila* line (BDSC 36304), by transgenic injections, at the NCBS-CCAMP transgenic facility, Bangalore, India. The transgenic dual sgRNA line was crossed to *nanos-Cas9* (BDSC 54591) line. The founder male progenies obtained were crossed to *w-; FM7a* balancer females wherein the Cas9-sgRNA complex is formed in the germline. In the next generation, three heterozygous female progenies from each cross (60 lines, each labeled A, B, and C) were maintained as a separate line over a *FM7a* balancer. Since the genomic *RRS* is located on the X chromosome, putative *RRS* lof lines were screened for male lethality. Lines showing male lethality were chosen for PCR-based confirmation of the deletion. Single fly genomic PCR for the extended gene region of *RRS* was performed on heterozygous females and the mutations were confirmed through sequencing.

### pUASp-AttB Fly Lines/Strains

Arginyl tRNA Synthetase*-WT* and *RRS-SCR(K147R,K383R)* were sub-cloned into *pUASp-attP2* using a homology based recombination technique, a modification of the SLiCE protocol (Zhang et al., 2014). These were injected into *AttB* lines for generation of transgenic fly lines. Fly lines were balanced with ubiquitously expressing Gal4s (Actin-Gal4/Ubiquitin-Gal4) of the following genotype *Actin-Gal4/* + *; UAS-RRS-WT/* + , *Actin-Gal4/* + *; UAS-RRS-SCR(2MT), Ubiquitin-gal4/* + *; UAS-RRS-WT/* + and *Ubiquitin-Gal4/* + *:UAS SCR(2MT).* All experiments were carried out with the *Actin-Gal4* line.

### Culturing and Processing Bacteria for Infections

*Micrococcus luteus* and *Ecc15* were plated on Luria-Bertani (LB) agar plates and grown in LB broth under antibiotic selection. Bacteria were collected from the plate or pelleted and re-suspended in 1X PBS to make a concentrated solution.

### Fly Infections

Six to eight day-old males were collected and placed at 29°C for 48 h, to acclimatize the flies to infection temperature. To cause septic injury, flies were pricked in the thorax with a needle dipped in the concentrated solution of bacteria. To activate the Toll-pathway and Imd-pathway, flies were infected with *M. luteus* and *Ecc15*, respectively, at an Optical density of 100, measured at 600 nm. To measure gene expression levels, infected flies and non-infected controls were incubated at 29°C for the time required, after which they were collected by snap freezing them in liquid nitrogen and stored at −80°C until RNA extraction. Infectivity assays were done in three biological replicates, ten flies per replicate. For survival experiments, flies were pricked in the same way as for the gene expression measurements.

### Total RNA Extraction cDNA Library Construction and Sequencing

Total RNA was extracted from adult flies with following genotypes *RRS^6B1^/Y; Actin Gal4/* + *; UAS-RRS WT/* + and *RRS^6B1^/Y; Actin Gal4/* + *; UAS-RRS ^*SCR*^/* +, 10 days post eclosion, in triplicates using RNeasy Plus Universal Kits (Qiagen; Part #74104) under control and infected conditions, according to manufacturer’s instructions and RNA integrity was assessed. 3′ mRNA specific libraries were amplified using QuantSeq 3′ mRNA-Seq Library Prep Kit FWD using the manufacturer’s instructions. Quality assessment for the cDNA libraries was done using Bioanalyzer 2100 (Agilent Technologies). Single end 75 bp sequencing of the pooled libraries were performed on the Illumina NextSeq 500 platform.

### Demultiplexing, Adapter Trimming, Read Mapping, Counts Generation and Differential Expression Analysis

On average 4–5 million reads were generated per sample. The raw reads were demultiplexed using bcl2fastq and the adapters were trimmed using bbduk v35.92. Sequencing quality was assessed using FastQC v0.11.5. Post quality control, the reads were mapped to the *Drosophila* genome (dm6) using STAR aligner v.2.5.2a (Dobin et al., 2013). Gene expression levels were measured using the counts generated by HTSeq-count v 0.6.0 (Anders et al., 2015). The gene expression counts were normalized for all samples together and the biological conditions were compared pairwise using DESeq2 (Love et al., 2014). The Principle Component Analysis using the “R” package of the regularized log counts were used to remove outliers from the final differential expression analysis. The regularized log transformed counts of the transcripts from DESeq2 were used to determine upregulated and downregulated genes across biological samples. Genes with log_2_(FC) values ≥ 0.55, ≤ −0.55 and -log_10_(FDR) values ≥ 2 were considered for further analysis. Gene Ontology analysis was done using a subroutine in Flybase.

Custom Venn diagrams were made using the Venneuler package in R to show the overlap and differences between the differentially expressed gene lists. Volcano plots were made using Graphpad Prism 8.0.2 for visual identification of genes with large fold changes that are also statistically significant.

### Survival Analysis

Survival assays were carried out on *RRS*^*WT*^ and *RRS*^*SCR*^ flies (Figures 2G,H). For each experiment flies were infected 10 days post eclosion. ∼40 age-matched male flies of the desired genotype were collected, each vial containing 10 flies. Animals were flipped to a fresh vial every 5 days, with number of flies recorded per vial daily. The survival data was plotted and analyzed using the log-rank test in Prism 8.

### Real Time-PCR

mRNA was extracted from 10-day old adults post infection using Qiagen RNeasy mini kit (74104). 500 ng of RNA was used for the cDNA synthesis using the High Capacity cDNA Reverse Transcriptase Kit (4368814) by Applied Biosystems. The qPCR reaction was carried out using KAPA SYBR FAST (KK4602) Sigma using Analytik Jena—qTOWER^3^—Real Time PCR Thermal Cycler. The experiments were carried out in triplicates with two technical replicates each. The relative fold change for each genotype was calculated by normalizing to house-keeping gene rp49. The data was analyzed by Two-way ANOVA followed by Tukey’s test for multiple comparison. The primer pairs used are listed in the Resource Table (Supplementary Figure 7).

## Data Availability Statement

The original contributions presented in the study are included in the article/Supplementary Material, further inquiries can be directed to the corresponding author/s.

## Author Contributions

PN and GR conceptualized the project, designed the experiments, analyzed the data, and wrote the manuscript. PN and AK performed all the experiments. GR supervised the project and acquired funding. All authors contributed to the article and approved the submitted version.

## Conflict of Interest

The authors declare that the research was conducted in the absence of any commercial or financial relationships that could be construed as a potential conflict of interest.

## Publisher’s Note

All claims expressed in this article are solely those of the authors and do not necessarily represent those of their affiliated organizations, or those of the publisher, the editors and the reviewers. Any product that may be evaluated in this article, or claim that may be made by its manufacturer, is not guaranteed or endorsed by the publisher.

## Supplementary Material

Supplementary Figure 1 SUMO conjugated proteins based on proteomic studies.

Supplementary Figure 2 **(A)** Multiple sequence alignment of *Drosophila RRS* and *RRS*^6B1^ DNA sequences **(B)**. Structural consequences of CRISPR/Cas9 mediated insertions in *RRS*^6B1^ flies **(C)**. DNA sequencing data for *RRS*^18B1^ **(D)** RRS CRISPR variant flies are haplo-sufficient and do not show embryonic lethality.

Supplementary Figure 3 **(A–D)** Expanded volcano plots for differentially expressed genes.

Supplementary Figure 4 Gene Ontology (GO) analysis of the significantly differentially regulated genes in RRS^*WT*^ and RRS^*SCR*^ post infection *M. luteus* **(A)** *Ecc15* **(B)**.

Supplementary Figure 5 **(A,B)** Gene Ontology (GO) analysis of the significantly differentially regulated genes in *RRS*^*SCR/WT*^ post infection.

Supplementary Figure 6 **(A,B)** Gene Ontology (GO) Enrichment Analysis of the significantly differentially regulated genes in *RRS*^*SCR/WT*^ post infection.

Supplementary Figure 7 Resource Table.

Supplementary Table 1 XLS file with processed data on gene expression levels after *M. luteus* infection.

Supplementary Table 2 XLS file with processed data on gene expression levels after *Ecc 15* infection.

Supplementary Table 3 XLS file with processed data for uniquely expressed genes for both *M. luteus* and *Ecc 15*, post infection.

Supplementary Table 4 XLS file with processed data for differentially expressed genes for *M. luteus*, without infection (0 h).

Supplementary Table 5 XLS file with processed data for differentially expressed genes *Ecc 15*, without infection (0 h).

Supplementary Model S1 *Drosophila* RRS structural model generated by the automated SWISS-MODEL server, in PDB format.
