# Supplementary material for: SUMOylation of Arginyl tRNA Synthetase Modulates the Drosophila Innate Immune Response
Source: Front Cell Dev Biol. 2021 Sep 30;9:695630. doi: 10.3389/fcell.2021.695630 (PMC8514731; doi:10.3389/fcell.2021.695630)
Supplement: Supplementary Figure 1 — SUMO conjugated proteins based on proteomic studies. [file Data_Sheet_1.pdf]

Suppl. Figure 1. **SUMO conjugated proteins based on Proteomic studies**. Data in literature suggests that elements of the MARS complex are SUMO Conjugated. The table compiles studies from yeast, flies and mammals. Cells marked with grey indicate that the protein in the corresponding row is SUMO conjugated, based on Proteomics data.

		Yeast	Insect (*Drosophila*)			Mammals
			0-3hr embryos	S2 cells	S2 cells	Hela and U2OS cells
	**NAME**	*Panse et al., 2004*	*Nie et al., 2009*	*Handu et al., 2015*	*Pirone et al., 2017*	*Hendriks et al.,2016*
**MARS (tRNA Synthetases)**	Lysyl-tRNA synthetase			■	■	■
	Leucyl-tRNA synthetase					■
	Arginyl-tRNA synthetase		■	■	■	
	Aspartyl-tRNA synthetase				■	■
	Glutaminyl-tRNA synthetase		■	■	■	■
	Glutamyl-prolyl-tRNA synthetase	■	■	■	■	■
	Isoleucyl-tRNA synthetase	■		■		■
	Methionyl-tRNA synthetase	■				■
**MARS (AIMPs)**	aaRS-interacting multifunctional protein 1					
	aaRS-interacting multifunctional protein 2		■		■	
	aaRS-interacting multifunctional protein 3					
**Non-MARS (tRNA Synthetases)**	Glycyl-tRNA synthetase	■		■		■
	Histidyl-tRNA synthetase			■	■	■
	Threonyl-tRNA synthetase					■
	Alanyl-tRNA synthetase			■	■	■
	Asparaginyl-tRNA synthetase	■		■	■	■
	Cysteinyl-tRNA synthetase			■		■
	Phenylalanyl-tRNA synthetase, alpha-subunit		■			■
	Phenylalanyl-tRNA synthetase, beta-subunit					■
	Seryl-tRNA synthetase					■
	Tryptophanyl-tRNA synthetase			■	■	■
	Tyrosyl-tRNA synthetase			■	■	■
	Valyl-tRNA synthetase				■	■

Suppl. Figure 2. Multiple Sequence Alignment of D*rosophila* RRS and RRS variant lines. The genomic region of *RRS*^{6B1} and *RRS*^{18B1} were sequenced and the DNA sequence aligned to that of wild-type RRS.

Suppl. Figure 2A. Forward and reverse sequencing of line 6B1.

```
Forward Primer (+ strand; RRS gene region) LINE 6B1

                                                sgRNA2
RRS      --------CCATGTGGCATCGTTGTGTTGTTGTTATTAATCAGCGGAGCAACAGAAGTCA 52
6B1      ACTTGCCACCATGTGGCATCGTTGTGTTG-------------GCGGAGCAACAGAAGTCA
                 *********************             ******************        13 bp deletion in 5' UTR
                 Translational Start
RRS      CAATTTGGAAACATGTCCGAGCTAAATATGGAGCTGAAAAAACTCAGGGAGCTGGTAAGC 112
6B1      CAATTTGGAAACATGTCCGAGCTAAATATGGAGCTGAAAAAACTCAGGGAGCTGGTAAGC
         ************************************************************

RRS      GAATAGCTGTTACTTTACGCCAACATTTTGACATGACATGCCGGCTTTAAAGGAACTGAA 172
6B1      GAATAGCTGTTACTTTACGCCAACATTTTGACATGACATGCCGGCTTTAAAGGAACTGAA
         ************************************************************

RRS      GACCCAAGGCCTTGCCGCCAGAATACAAACTGCCAAAAGCGGTGAACAGTTGGACGTCGA 232
6B1      GACCCAAGGCCTTGCCGCCAGAATACAAACTGCCAAAAGCGGTGAACAGTTGGACGTCGA
         ************************************************************

RRS      TCTTGTTCAGCTTCAAATTGAAAATAAGAAGCTGAAGAACCGCCTGTTTATCCTAAAGAA 292
6B1      TCTTGTTCAGCTTCAAATTGAAAATAAGAAGCTGAAGAACCGCCTGTTTATCCTAAAGAA
         ************************************************************

RRS      GGTGAGTTTATGACCCCCAATGTGTTTAAGCTTAAGAATTTCATGTCTTTTATTTCCCTG 352
6B1      GGTGAGTTTATGACCCCCAATGTGTTTAAGCTTAAGAATTTCATGTCTTTTATTTCCCTG
         ************************************************************

RRS      TTAGTCCATTGCTGAGGAATCAACTGCCGCCGGCGGCGACGTTTCGAAGCCCAAGGAATC 412
6B1      TTAGTCCATTGCTGAGGAATCAACTGCCGCCGGCGGCGACGTTTCGAAGCCCAAGGAATC
         ************************************************************

RRS      CTCTTCGATCACCGAACACCTGGAAAGCGTCTTTCGCCAGGCGATTGCATCAGCTTTCCC 472
6B1      CTCTTCGATCACCGAACACCTGGAAAGCGTCTTTCGCCAGGCGATTGCATCAGCTTTCCC
         ************************************************************

RRS      GGAATTCAGAGATACGCCTGTTATAATTGCACCAGTTAATAGTACGTCTGCGAAATTCGG 532
6B1      GGAATTCAGAGATACGCCTGTTATAATTGCACCAGTTAATAGTACGTCTGCGAAATTCGG
         ************************************************************

RRS      CGACTATCAGTGCAACAATGCCATGGGATTGTCCAAGAAGCTGAAAGAGAAGGGCATTAA 592
6B1      CGACTATCAGTGCAACAATGCCATGGGATTGTCCAAGAAGCTGAAAGAGAAGGGCATTAA
         ************************************************************

RRS      TAAAGCACCACGTGATATTGCAACCGAGTTGAAAGGACACTGCCCAGCATCACCAATCAT 652
6B1      TAAAGCACCACGTGATATTGCAACCGAGTTGAAAGGACACTGCCCAGCATCACCAATCAT
         ************************************************************
```

```
Reverse Primer (+ strand; RRS gene region) LINE 6B1

RRS     ACCAATTTACCCGAAATACTGAAGAAGACCAACATTGTGTTGGACCACGAAAAGGAATGG 2040
6B1     ACCAATTTACCCGAAATACTGAAGAAGACCAACATTGTGTTGGACCACGAAAAGGAATGG
        ************************************************************

RRS     AAGCTGGCTAAGACTCTACTGAAACTCCACGACATACTCATCAAGTGCTCAAAGGAACTT 2100
6B1     AAGCTGGCTAAGACTCTACTGAAACTCCACGACATACTCATCAAGTGCTCAAAGGAACTT
        ************************************************************
                                      sgRNA1
RRS     TTCCTGCACTTCCTG------TGCGAGTTTTGCTTCGAGGTGTGCACAGTGTTCACCGAA 2154
6B1     TTCCTGCACTTCCTGTGTTTTTGCGAGTTTTGCTTCGAGGTGTGCACAGTGTTCACCGAA
        ***************      ***************************************

RRS     TTCTATGACTCTTGTTATTGCATCGAAAAGAACAAACAAGGCGATATTATTGGGGTCAAT 2214
6B1     TTCTATGACTCTTGTTATTGCATCGAAAAGAACAAACAAGGCGATATTATCGGGGTCAAT
        ************************************************** *********

RRS     CATAGCCGAATTCTATTGTGCGAGGCAACTGCGGCTGTGTTGCGCCAATGCTTTTATATA 2274
6B1     CATAGCCGAATTCTATTGTGCGAGGCAACTGCGGCTGTGTTGCGCCAATGCTTTTATATA
        ************************************************************
            Translational Stop
RRS     CTAGGCCTTAAACCAGTTTCGAAAATGTAAAAAGTTCTACTACTGACATCATGTGTACGC 2334
6B1     CTAGGCCTTAAACCAGTTTCGAAAATGTAAAAAGTTCTACTACTGACATCATGTGTACGC
        ************************************************************
```

6 bp insertion in Coding region

Suppl. Figure 2B. **Structural consequences of CRISPR/Cas9 mediated insertions in *RRS*[6B1]**.
B1-B2. The *Drosophila* RRS structure, modeled by SWISS-MODEL predicts a potential disulphide bond between C515:C604, two alpha helices that connect that catalytic and C-terminal tRNA binding domains. Prediction was based on analysis by the Disulfide by Designs server (http://cptweb.cpt.wayne.edu/DbD2/) (Craig et. al., 2013). This disulfide if present, could be a significant factor for folding and stability of RRS. A second potential disulfide is between C607:C653 (panel B2).
B3-B4. For line 6B1 An insertion of two amino-acids, Leucine and Phenylalanine in positions 604 and 605 would cause a shift in the Cystine (604 in wt) to position 606, on the opposite face of the helix, which would lead to a disruption of the disulfide bond. The insertion could also perturb the side-chain interactions and further add to the destabilization of RRS.

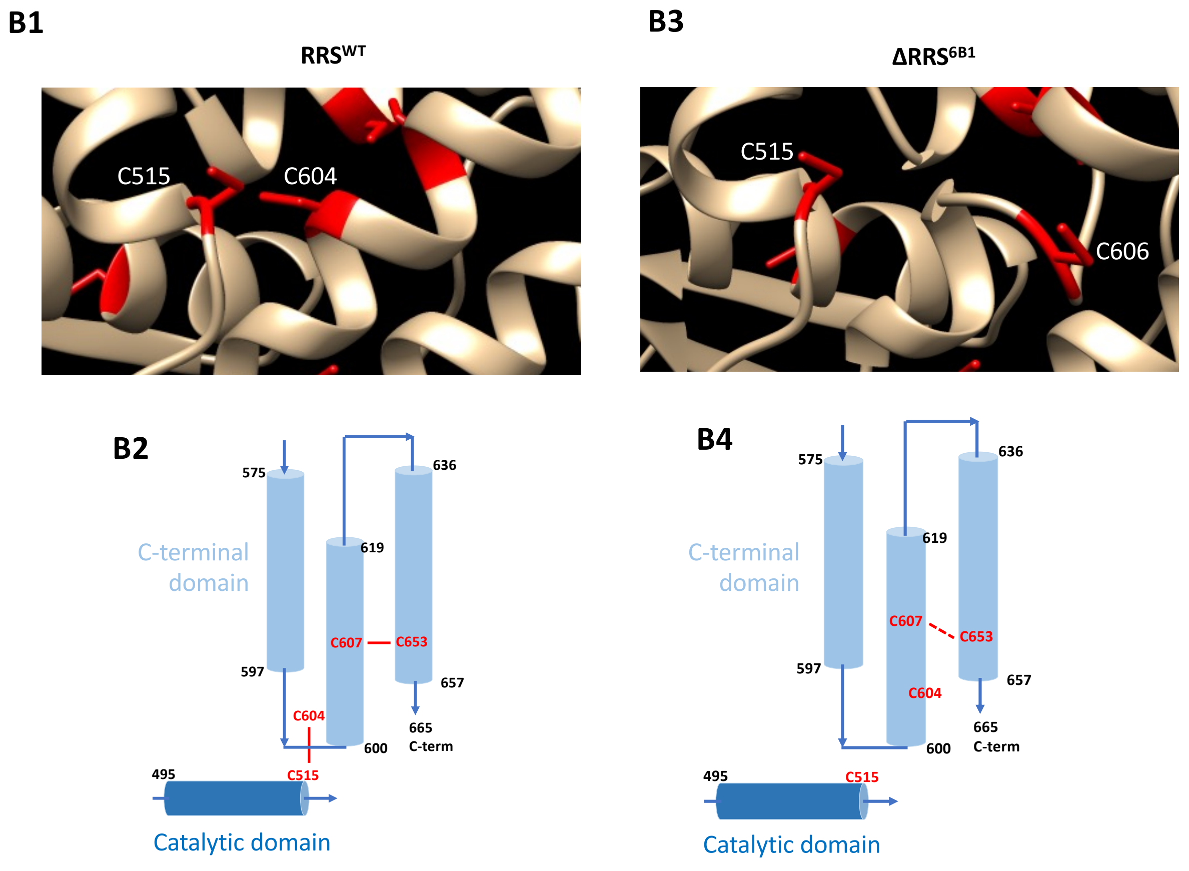

Suppl. Figure 2C. Forward and reverse sequencing of line 18B1.

```
Forward Primer (+ strand; RRS gene region) LINE 18B1

RRS          ----------------------------------------------------CCATGTGG 8
18B1         GGANCCAGCTGTTATCGTTATCGATAGGCGACGTGTGCACACTACTTGCCATCCATGTGG
                                                                 ********
                                                sgRNA2
RRS          CATCGTTGTGTTGTTGTTATTAA-----------TCAGCGGAGCAACAGAAGTCACAATT 57
18B1         CATCGTTGTGTTGTTGTTATTAGCGTTAACGCTAATAGCGGAGCAACAGAAGTCACAATT
             **********************              ************************
               Translational Start
RRS          TGGAAACATGTCCGAGCTAAATATGGAGCTGAAAAAACTCAGGGAGCTGGTAAGCGAATA 117
18B1         TGGAAACATGTCCGAGCTAAATATGGAGCTGAAAAAACTCAGGGAGCTGGTAAGCGAATA
             ************************************************************

RRS          GCTGTTACTTTACGCCAACATTTTGACATGACATGCCGGCTTTAAAGGAACTGAAGACCC 177
18B1         GCTGTTACTTTACGCCAACATTTTGACATGACATGCCGGCTTTAAAGGAACTGAAGACCC
             ************************************************************

RRS          AAGGCCTTGCCGCCAGAATACAAACTGCCAAAAGCGGTGAACAGTTGGACGTCGATCTTG 237
18B1         AAGGCCTTGCCGCCAGAATACAAACTGCCAAAAGCGGTGAACAGTTGGACGTCGATCTTG
             ************************************************************

RRS          TTCAGCTTCAAATTGAAAATAAGAAGCTGAAGAACCGCCTGTTTATCCTAAAGAAGGTGA 297
18B1         TTCAGCTTCAAATTGAAAATAAGAAGCTGAAGAACCGCCTGTTTATCCTAAAGAAGGTGA
             ************************************************************

RRS          GTTTATGACCCCCAATGTGTTTAAGCTTAAGAATTTCATGTCTTTTATTTCCCTGTTAGT 357
18B1         GTTTATGACCCCCAATGTGTTTAAGCTTAAGAATTTCATGTCTTTTATTTCCCTGTTAGT
             ************************************************************

RRS          CCATTGCTGAGGAATCAACTGCCGCCGGCGGCGACGTTTCGAAGCCCAAGGAATCCTCTT 417
18B1         CCATTGCTGAGGAATCAACTGCCGCCGGCGGCGACGTTTCGAAGCCCAAGGAATCCTCTT
             ************************************************************

RRS          CGATCACCGAACACCTGGAAAGCGTCTTTCGCCAGGCGATTGCATCAGCTTTCCCGGAAT 477
18B1         CGATCACCGAACACCTGGAAAGCGTCTTTCGCCAGGCGATTGCATCAGCTTTCCCGGAAT
             ************************************************************

RRS          TCAGAGATACGCCTGTTATAATTGCACCAGTTAATAGTACGTCTGCGAAATTCGGCGACT 537
18B1         TCAGAGATACGCCTGTTATAATTGCACCAGTTAATAGTACGTCTGCGAAATTCGGCGACT
             ************************************************************

RRS          ATCAGTGCAACAATGCCATGGGATTGTCCAAGAAGCTGAAAGAGAAGGGCATTAATAAAG 597
18B1         ATCAGTGCAACAATGCCATGGGATTGTCCAAGAAGCTGAAAGAGAAGGGCATTAATAAAG
             ************************************************************

RRS          CACCACGTGATATTGCAACCGAGTTGAAAGGACACTGCCCAGCATCACCAATCATTGAAA 657
18B1         CACCACGTGATATTGCAACCGAGTTGAAAGGACACTGCCCAGCATCACCAATCATTGAAA
             ************************************************************

RRS          AGCTGGAAATTGCCGGAGCTGGCTTCGTAAACGTGTTCCTTAGCAAGTGAGTGGCATAAG 717
18B1         AGCTGGAAATTGCCGGAGCTGGCTTCGTAAACGTGTTCCTTAGCAAGTGAGTGGCATAAG
             ************************************************************

RRS          TACACAAAGTCCATTATAAACATTAAAAATGTTAATTTTTGCAGAGATTATGCATCTTTA 777
18B1         TACACAAAGTCCATTATAAACATTAAAAATGTTAATTTTTGCAGAGATTATGCATCTTTA
             ************************************************************
```

ATC, 6 bp upstream of PAM site of gRNA sequence is replaced by a 14 bp insertion in 5' UTR.

Suppl. Figure 2

Reverse Primer (Sequencing Run 1) (- strand; RRS gene region) LINE 18B1

```
RRS        ---------------------------------------------------ATTCGCGTA 2329
18B1a      ACCAATTCTTTTCAAGGTATATAATTTTACTTTTATAAAATGTTTCATTTTATTCGCGTA
                                                              *********
                                                  Translational Stop
RRS        CACATGATGTCAGTAGTAGAACTTTTTACATTTTCGAAACTGGTTTAAGGCCTAGTATAT 2269
18B1a      CACATGATGTCAGTAGTAGAACTTTTTACATTTTCGAAACTGGTTTAAGGCCTAGTATAT
           ************************************************************

RRS        AAAAGCATTGGCGCAACACAGCCGCAGTTGCCTCGCACAATAGAATTCGGCTATGATTGA 2209
18B1a      AAAAGCATTGGCGCAACACAGCCGCAGTTGCCTCGCACAATAGAATTCGGCTATGATTGA
           ************************************************************

RRS        CCCCAATAATATCGCCTTGTTTGTTCTTTTCGATGCAATAACAAGAGTCATAGAATTCGG 2149
18B1a      CCCCGATAATATCGCCTTGTTTGTTCTTTTCGATGCAATAACAAGAGTCATAGAATTCGG
           **** *******************************************************
                             sgRNA1
RRS        TGAACACTGTGCACACCTCGAAGCAAAACTCGCACAGGAAGTGCAGGAAAAGTTCCTTTG 2089
18B1a      TGAACACTGTGCACACCTCGAAGCAAAACTCGCACAAGAACTGGAAGAGCAGGACCTTTG
           ************************************ *** ** * ** **  ******

RRS        AGCACTTGATGAGTATGTCGTGGAGTTTCAGTAGAGTCTTAGCCAGCTTCCATTCCTTTT 2029
18B1a      ACTTCTTGATCATTATGACTATGACTTGCAGTATAGTCTTAGCCATCTTCCATTCCTTTT
           *   ****** * **** *   ** ** ***** *********** **************

RRS        CGTGGTCCAACACAATGTTGGTCTTCTTCAGTATTT------------------------ 1969
18B1a      CGTGTTCCAGCACCATGTTGGTGTTGTTCTTCATCTCTATTACATTGATGATGTCTTAAT
           **** **** *** ******** ** ***   ** *
```

Sequencing fails after sgRNA1 region (attempt 1)

Reverse Primer (Sequencing Run 2)(- strand; RRS gene region) LINE 18B1

```
RRS        ------TTATTCGCGTACACATGATGTCAGTAGTAGAACTTTTTACATTTTCGAAACTGG 2083
18B1b      TTCATTTTATTCGCGTACACATGATGTCAGTAGTAGAACTTTTTACATTTTCGAAACTGG
                 ******************************************************
     Translational Stop
RRS        TTTAAGGCCTAGTATATAAAAGCATTGGCGCAACACAGCCGCAGTTGCCTCGCACAATAG 2023
18B1b      TTTAAGGCCTAGTATATAAAAGCATTGGCGCAACACAGCCGCAGTTGCCTCGCACAATAG
           ************************************************************

RRS        AATTCGGCTATGATTGACCCCAATAATATCGCCTTGTTTGTTCTTTTCGATGCAATAACA 1963
18B1b      AATTCGGCTATGATTGACCCCGATAATATCGCCTTGTTTGTTCTTTTCGATGCAATAACA
           ********************* **************************************
                                                sgRNA1
RRS        AGAGTCATAGAATTCGGTGAACACTGTGCACACCTCGAAGCAAAACTCGCACAGGAAGTG 1903
18B1b      AGAGTCATAGAATTCGGTGAACACTGTGCACACCTCGAAGCAAAACTCGCACAAGAACTG
           ***************************************************** *** **

RRS        CAGGAAAAGTTCCTTTGAGCACTTGATGAGTATGTCGTGGAGTTTCAGTAGAGTCTTAGC 1843
18B1b      GAAGAGCAGGACCTTTGACCTCTAGATCATGATGACTAGGACTTGCAGTATAGTCATAGC
            * **  ** ******* * ** *** *  *** *  *** ** ***** **** ****

RRS        CAGCTTCCATTCCTTTTCGTGGTCCAACACAATGTTGGTCTTCTTCAGTATTTCGGGTAA 1783
18B1b      CTTATTCCATTCCTTTTCCTGGTCCAGCACCATGTCGGTGTTGGTCTTCATTTCTATTAC
           *   ************** ******* *** **** *** **  **   *****   **
RRS        ATTGGTGAAATCTTCGCCAGAGTTT----------C 380
18B1b      ATTGATGATGTCGACATCATAGCCTCATGCTCTGGA 396
           **** ***  **  *  ** **  *
```

Sequencing fails after sgRNA1 region (attempt 2)

Suppl. Figure 2D. **ΔRRS flies are haplo-sufficient**. The RRS variant animals, generated by CRISPR Cas9 genome editing showed lifespan (at 29 °C), similar to that of *w1118* files (panel A) for genotypes RRS^{6B1}/+ and RRS^{18B1} /+ . Log-rank(Mantel Cox) survival plot using GraphPad Prism 8.0.2 using Kaplan-Meier and Gehan-Wilcoxon tests suggests that RRS^{6B1}/+ and RRS^{18B1} /+ flies show enhanced survival as compared to the control.(**** P<0.0001). The RRS^{6B1}/+ and RRS^{18B1}/+ flies also did not show any defects in embryonic development, based on hatching of eggs, which was again at par with *w1118.* Homozygous RRS^{6B1} and RRS^{18B1} flies are 2nd instar larval lethals. *W1118* is not the ideal line for this comparison as it shows short life span and neurodegenerative phenotypes (Ferreiro *et. al.,* 2017).

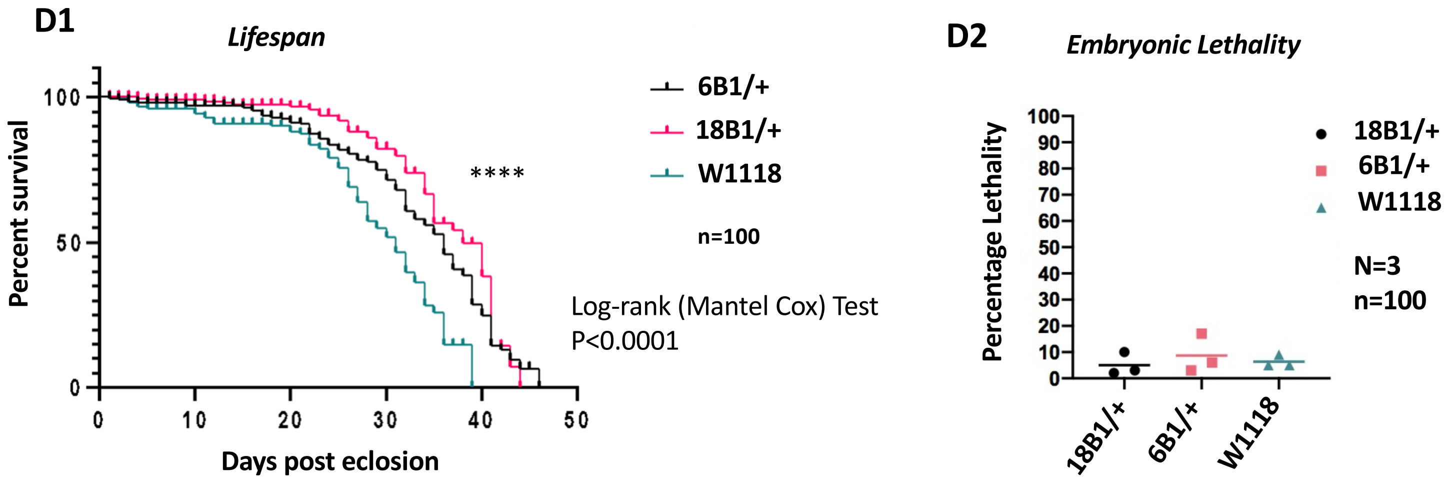

Suppl. Figure 3: Volcano plot(s) for The differentially expressed genes. $\log_2$(FC) for each gene is plotted against its $-\log_{10}$(FDR) value to display differentially expressed genes upon infection as compared to the baseline. Red and blue dots represent the genes which are significantly differentially expressed with $\log_2$(FC) of > 0.55 and < -0.55 respectively with *p-value* < 0.05 {-$\log_{10}$(FDR) of > 2} whereas black dots represent the genes which are uniformly expressed. Representative genes most significantly differentially expressed are highlighted.

3A. ***M. luteus; RRS***^{WT} **22hours**

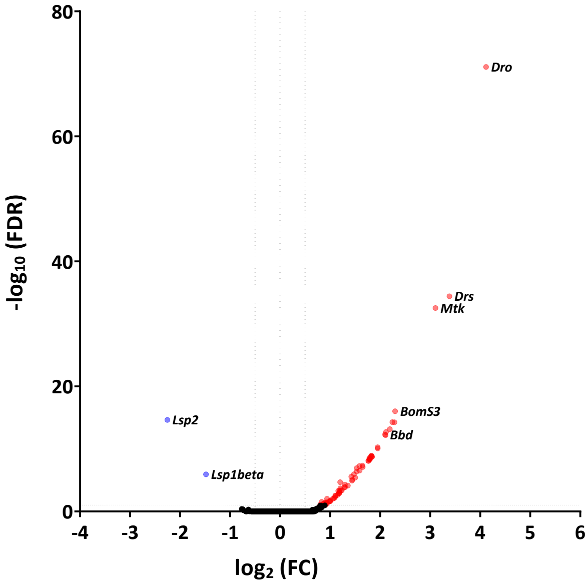

3B. ***M. luteus; RRS***^{SCR} **22hours**

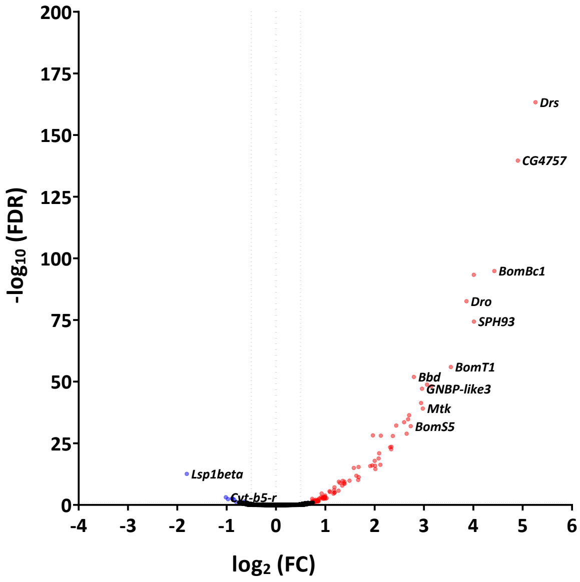

3C. ***Ecc15; RRS***^{WT} **12hours**

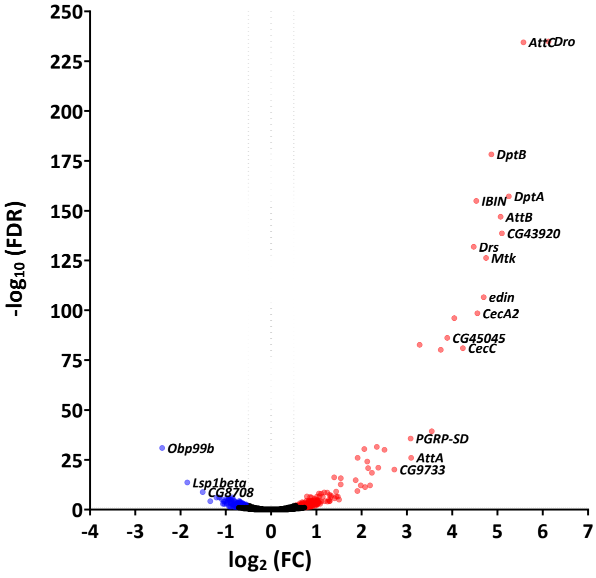

3D. ***Ecc15; RRS***^{SCR} **12hours**

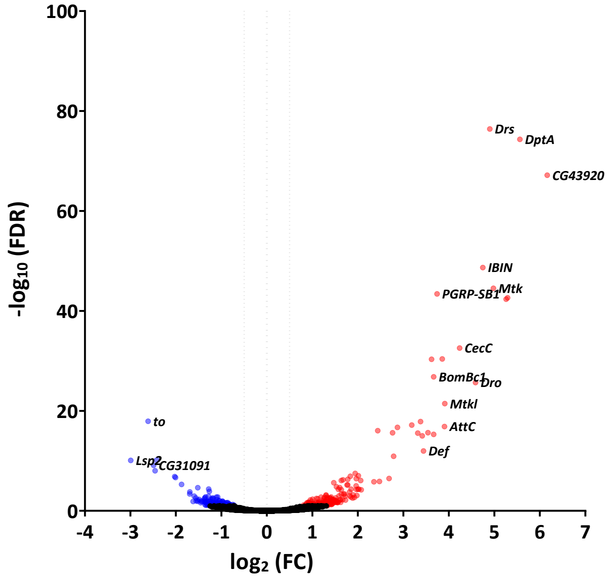

Suppl. Figure 4. **Gene Ontology Enrichment Analysis (GOEA) of the significantly differentially expressed genes in *RRS^{WT}* and RRS^{SCR} post infection with *M. luteus* and *Ecc15* .** Both *RRS^{WT}* and *RRS^{SCR}* show differential expression of immune responsive genes post infection.

Suppl. Figure 4A.. GOEA is done for 4 different categories (A1) Biological Process, (A2) Molecular Function, (A3) Cellular Component and (A4) Protein Class for *RRS^{WT}* and *RRS^{SCR}* post infection with *M. luteus.* The number of genes enriched in each sub-category is plotted against its corresponding {-log$_{10}$(FDR)} value. A few key GO terms are highlighted and listed (A5).

A1 Biological Process

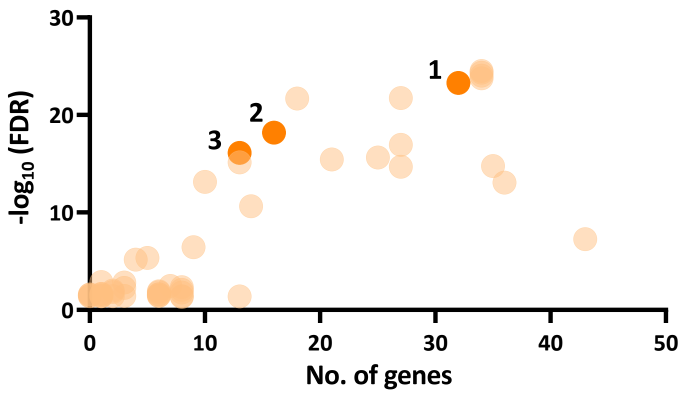

A2 Molecular Function

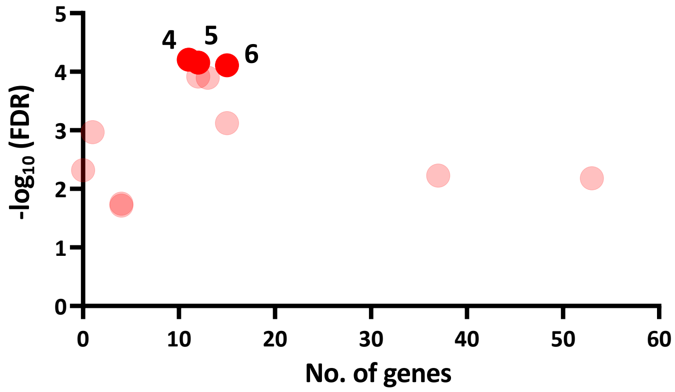

A3 Cellular Component

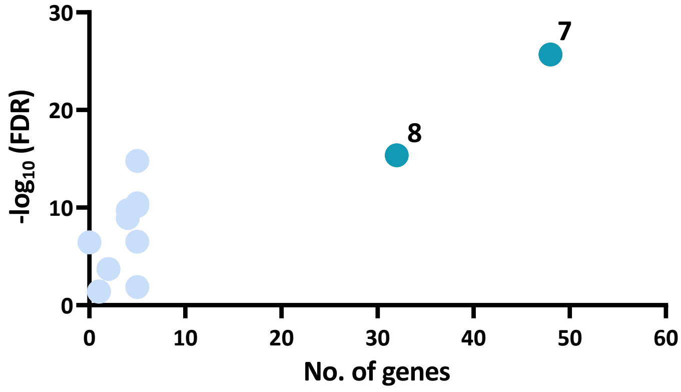

A4 Protein Class

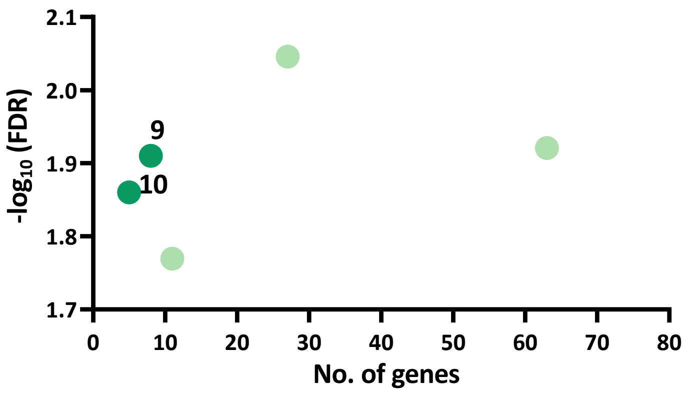

A5

1. Defense response
2. Defense response to Gram-positive bacterium
3. Antibacterial humoral response
4. Binding
5. Serine-type endopeptidase activity
6. Endopeptidase activity
7. Extracellular region
8. Extracellular space
9. Serine protease
10. Protease inhibitor

Suppl. Figure 4B. GOEA is done for 4 different categories (B1) Biological Process, (B2) Molecular Function, (B3) Cellular Component and (B4) Protein Class for *RRS*^{WT} and *RRS*^{SCR} post infection with *Ecc15*. The number of genes enriched in each sub-category is plotted against its corresponding {-log$_{10}$(FDR)} value. A few key GO terms are highlighted and listed (B5).

B1 Biological Process

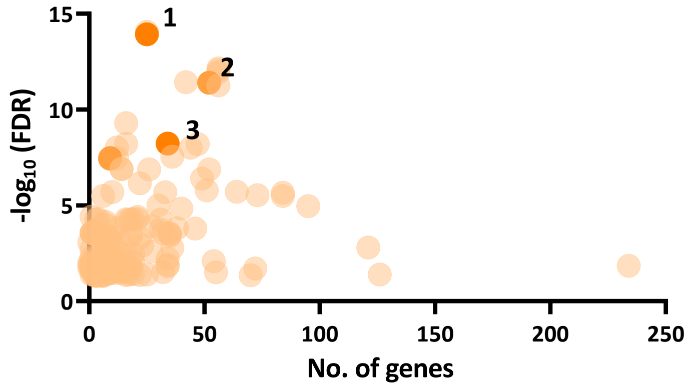

B2 Molecular Function

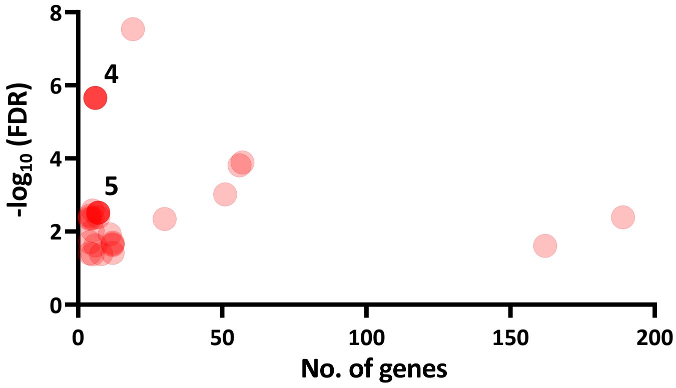

B3 Cellular Component

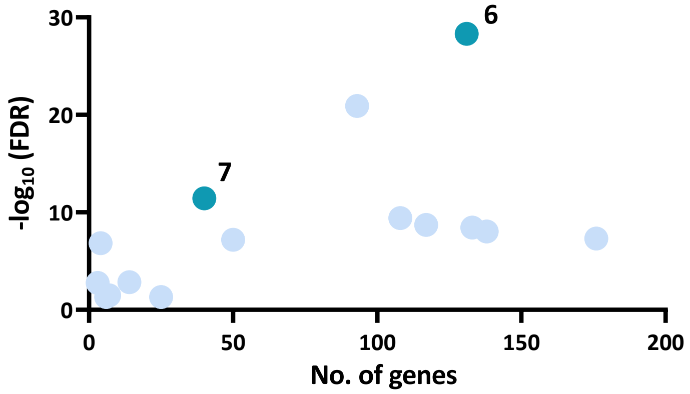

B4 Protein Class

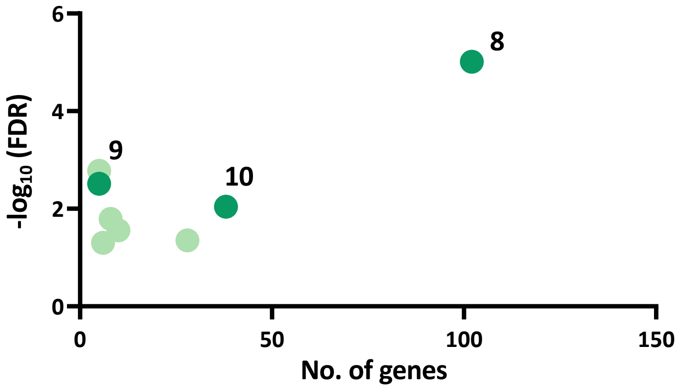

B5

1. Humoral immune response
2. Defense response to bacterium
3. Regulation of transcription, DNA-templated
4. DNA binding
5. Transcription regulator activity
6. Extracellular region
7. Nucleus
8. Metabolite interconversion enzyme
9. DNA-binding transcription factor
10. Oxidoreductase

Suppl. Figure 5. Gene Ontology (GO) Analysis for significantly differentially expressed genes for RRS$^{SCR/WT}$ post infection with *M. luteus* (A) and *Ecc15* (B). Number of genes enriched in each category is plotted against their Panther Gene Ontology terms.

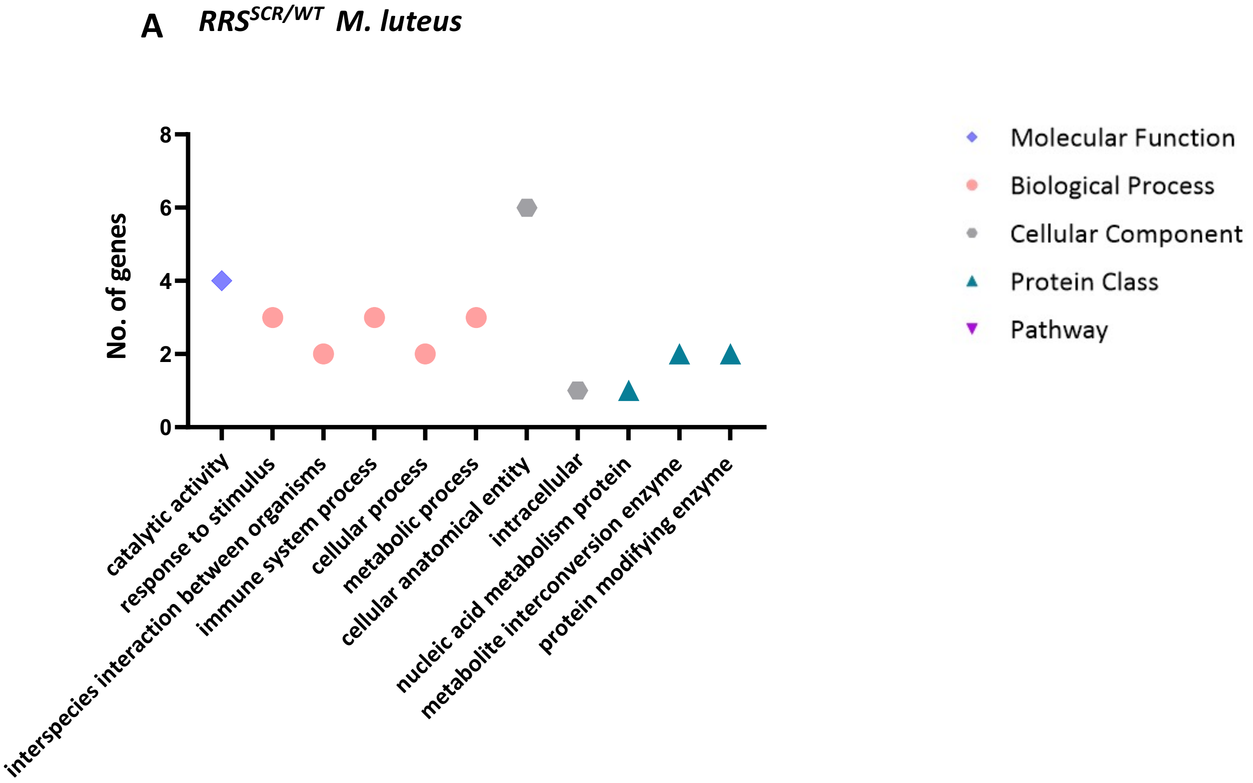

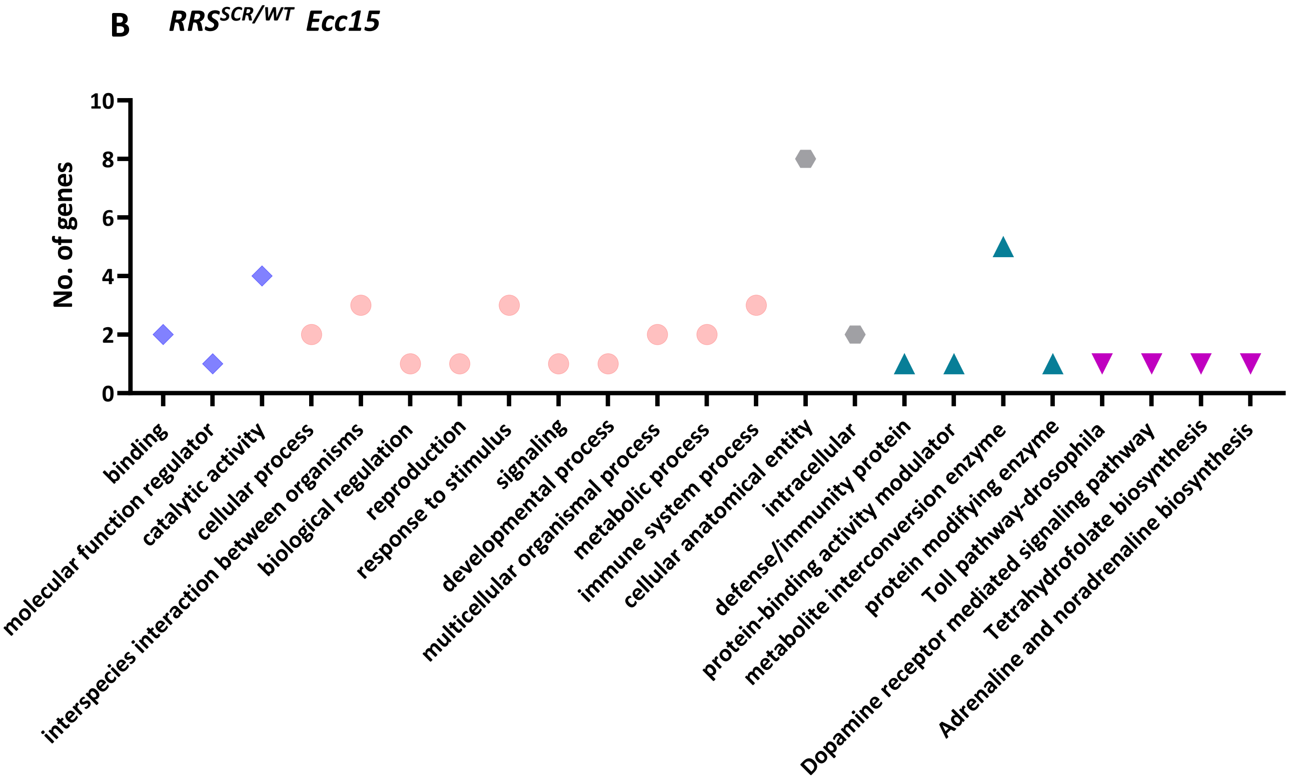

Suppl. Figure 6. Gene Ontology Enrichment Analysis (GOEA) for significantly differentially expressed genes for *RRS^SCR/WT^* post infection with *M. luteus* and *Ecc15.*

Suppl. Figure 6A. GOEA is done for 2 different categories (A1) Biological process and (A2) Cellular Component and for *RRS^SCR/WT^* post infection with *M. luteus.* The number of genes enriched in each sub-category is plotted against its corresponding {-$\log_{10}$(FDR)} value. A few key GO terms are highlighted in the expanded list (A3).

***RRS^SCR/WT^* M. luteus**

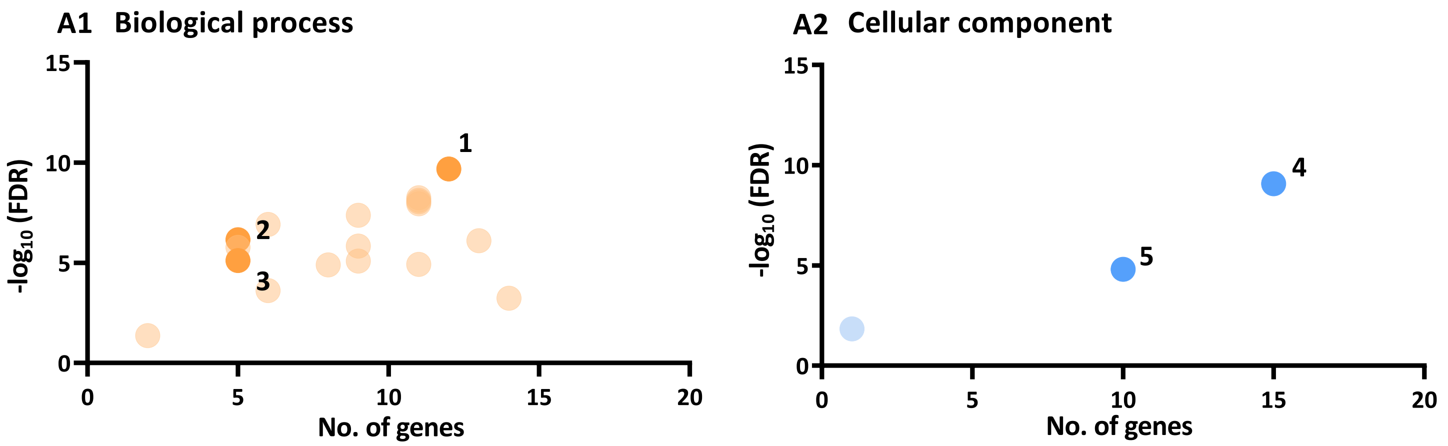

A3

Biological Process		
**Category**	**No.of genes**	**-$\log_{10}$(FDR)**
Defense response (1)	12	9.681936665
Response to biotic stimulus	11	8.248720896
Response to other organism	11	8.123782159
Biological process involved in interspecies interaction between organisms	11	8.049635146
Response to external biotic stimulus	11	7.946921557
Response to bacterium	9	7.374687549
Humoral immune response	6	6.913640169
Antibacterial humoral response (2)	5	6.158015195
Response to stress	13	6.107905397
Immune response	9	5.835647144
Antimicrobial humoral response	5	5.777283529
Defense response to Gram-positive bacterium (3)	5	5.107348966
Immune system process	9	5.083019953
Response to external stimulus	11	4.924453039
Defense response to other organism	8	4.896196279
Defense response to bacterium	6	3.614393726
Response to stimulus	14	3.234331445
Defense response to insect	2	1.370590401

Cellular Component		
**Category**	**No.of genes**	**-$\log_{10}$(FDR)**
Extracellular region (4)	15	9.07109231
Extracellular space (5)	10	4.806875402
Intracellular anatomical structure	1	1.838631998

Suppl. Figure 6B. GOEA is done for 2 different categories (B1) Biological process and (B2) Cellular Component and for *RRS*$^{SCR/WT}$ post infection with *Ecc15*. The number of genes enriched in each sub-category is plotted against its corresponding {-$\log_{10}$(FDR)} values. A few key GO terms are highlighted in the expanded list (B3).

RRS*$^{SCR/WT}$ *Ecc15

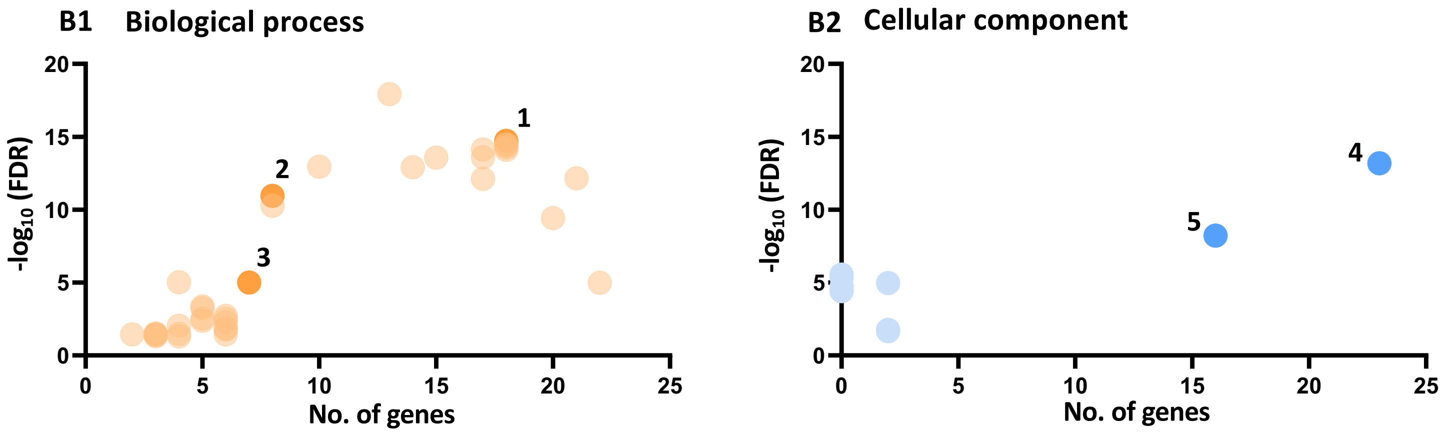

B3

Biological Process

Category	No.of genes	-$\log_{10}$(FDR)
Defense response to Gram-positive bacterium	13	17.91721463
Defense response (1)	18	14.73282827
Response to biotic stimulus	18	14.49485002
Response to other organism	18	14.3990271
Response to external biotic stimulus	18	14.27327279
Defense response to other organism	17	14.1266794
Biological process involved in interspecies interaction between organisms	18	14.09691001
Immune response	17	13.58838029
Response to bacterium	15	13.57839607
Humoral immune response	10	12.94309515
Defense response to bacterium	14	12.91009489
Response to external stimulus	21	12.14691047
Immune system process	17	12.1214782
Antibacterial humoral response (2)	8	10.95467702
Antimicrobial humoral response	8	10.26760624
Response to stress	20	9.42945706
Defense response to insect	4	5.014573526
Defense response to Gram-negative bacterium (3)	7	5.005682847
Response to stimulus	22	4.987162775
Cellular process	5	3.359518563
Regulation of defense response to bacterium	5	3.238824187
Regulation of defense response	6	2.723538196
Response to wounding	5	2.536107011
Regulation of response to biotic stimulus	6	2.511449283
Innate immune response	5	2.380906669

Biological Process		
Category	No.of genes	$-log_{10}(FDR)$
Innate immune response	5	2.380906669
Regulation of immune response	6	2.283996656
Response to fungus	4	2.045757491
Regulation of response to external stimulus	6	1.850780887
Regulation of immune system process	6	1.790484985
Regulation of peptidoglycan recognition protein signaling pathway	3	1.512861625
Positive regulation of defense response	4	1.484126156
Regulation of response to stress	6	1.454692884
Detection of biotic stimulus	2	1.452225295
Positive regulation of Toll signaling pathway	3	1.448550002
Regulation of antibacterial peptide biosynthetic process	3	1.441291429
Regulation of antibacterial peptide production	3	1.390405591
Regulation of antimicrobial peptide biosynthetic process	3	1.312471039
Positive regulation of response to biotic stimulus	4	1.304518324

Cellular Component		
Category	No.of genes	$-log_{10}(FDR)$
Extracellular region (4)	23	13.18708664
Extracellular space (5)	16	8.214670165
Organelle	0	5.55129368
Intracellular organelle	0	5.36251027
Intracellular anatomical structure	2	4.970616222
Membrane-bounded organelle	0	4.721246399
Intracellular membrane-bounded organelle	0	4.416801226
Larval serum protein complex	2	1.759450752
Cytoplasm	2	1.671620397

Suppl. Figure 7. **Resource Table**

Reagent Type (Species/Resource)	Designation	Source/Resource	Identifiers	Additional Information
Genetic reagent	*Nos-Cas9/Cyo*	*Bloomington Drosophila Stock Center, Indiana*	*BDSC:78781*	*Ren et. al., / Perrimon Lab* *Expresses Cas9 in the germline under the control of nos.*
Genetic reagent	*CG9020-UASgRNA/Cyo*	*This Study*		*Transgene dual gRNA inserted at Attp40 site*
Genetic reagent	*RRS CRISPR control/Fm7i, Actin-GFP*	*This Study*		*RRS CRISPR Control*
Genetic reagent	*RRS-6B1/ Fm7i, Actin-GFP*	*This Study*		*RRS CRISPR lof line*
Genetic reagent	*RRS-18B1/Fm7i, Actin-GFP*	*This Study*		*RRS CRISPR lof line*
Genetic reagent	*Actin Gal4/Cyo (y[1] w[*]; P{Act5C-GAL4-w}E1/CyO)*	*Bloomington Drosophila Stock Center, Indiana*	*BDSC:25374*	*Gal4 driven under Actin Promoter*
Genetic reagent	*Ubiquitin Gal4/Cyo (w[*]; P{w[+m*]=Ubi-GAL4.U}2/CyO)*	*Bloomington Drosophila Stock Center, Indiana*	*BDSC:32551*	*Gal4 driven under Ubiquitin Promoter*
Genetic reagent	*Actin Gal4/Cyo; HA-RRS WT/Ser*	*This Study*		*UAS-Gal4 line for RRS wildtype rescue*
Genetic reagent	*Actin Gal4/Cyo; HA-RRS SCR/Ser*	*This Study*		*UAS-Gal4 line for RRS SCR mutant rescue*
Genetic reagent	*Ubiquitin Gal4/Cyo; HA-RRS WT/Ser*	*This Study*		*UAS-Gal4 line for RRS wildtype rescue*
Genetic reagent	*Ubiquitin Gal4/Cyo; HA-RRS SCR/Ser*	*This Study*		*UAS-Gal4 line for RRS SCR mutant rescue*
cDNA construct	*Aats-arg (RRS)*	*BDGP DGC clones*	*RE02962*	*AU.46, 35, pFlc-1*
cDNA construct	*Aats-glupro (EPRS)*	*BDGP DGC clones*	*LD42739*	*AU.45,25, pOT2*
cDNA construct	*Aats-Isoleu (IRS)*	*BDGP DGC clones*	*LD27166*	*AU.44,17, pOT2*
cDNA construct	*Aats-Asp (DRS)*	*BDGP DGC clones*	*GM14334*	*AU.24,41, pOT2*
cDNA construct	*Aats-Leu (LRS)*	*BDGP DGC clones*	*LD44376*	*AU.44,32, pOT2*
cDNA construct	*Aats-Gln (QRS)*	*BDGP DGC clones*	*GH11673*	*AU.18,47, pOT2*
cDNA construct	*Aats-Lys (KRS)*	*BDGP DGC clones*	*LD41976*	*AU.132,86, pOT2*
cDNA construct	*Aats-Met (MRS)*	*BDGP DGC clones*	*GH13807*	*AU.27,26, pOT2*
cDNA construct	*AIMP1*	*cDNA (S2 cells)*		
cDNA construct	*AIMP2*	*BDGP DGC clones*	*LD25772*	*AU.79,13, pOT2*
cDNA construct	*AIMP3*	*BDGP DGC clones*	*RH48588*	*AU.57,60, pFlc-1*

Reagent Type (Species/Resource)	Designation	Source/Resource	Identifiers	Additional Information
Sequence-based reagent	*CG9020 FP2*	*This study (Sigma-Aldrich)*	*Screening primer forward*	*ACGACATCCTAGAAGTGACTG*
Sequence-based reagent	*CG9020 RP*	*This study (Sigma-Aldrich)*	*Screening primer reverse*	*CAGCTAGTGTGAATGCGAAC*
Sequence-based reagent	*RRS GENOMIC RP*	*This study (Sigma-Aldrich)*	*Screening primer reverse*	*CCAGATTACGCTGGCGGCATGTCCGAGCTAAA TATGGAGCTG*
Sequence-based reagent	*rp49 FP*	*This study (Sigma-Aldrich)*	*RT-PCR primer*	*GACGCTTCAAGGGACAGTATC*
Sequence-based reagent	*rp49 RP*	*This study (Sigma-Aldrich)*	*RT-PCR primer*	*AAACGCGGTTCTGCATGAG*
Sequence-based reagent	*RRS RT FP*	*This study (Eurofins)*	*RT-PCR primer*	*GCCTTGCCGCCAGAATACA*
Sequence-based reagent	*RRS RT RP*	*This study (Eurofins)*	*RT-PCR primer*	*GGCAGTTGATTCCTCAGCAAT*
Sequence-based reagent	*AttD FP*	*This study (Sigma-Aldrich)*	*RT-PCR primer*	*CGGTCAACGCCAATGGTCAT*
Sequence-based reagent	*AttD RP*	*This study (Sigma-Aldrich)*	*RT-PCR primer*	*CATTCAGAGCGGCGTTATTG*
Sequence-based reagent	*DptB FP*	*This study (Sigma-Aldrich)*	*RT-PCR primer*	*ACCGCAGTACCCACTCAATC*
Sequence-based reagent	*DptB RP*	*This study (Sigma-Aldrich)*	*RT-PCR primer*	*GGTCCACACCTTCTGGTGAC*
Sequence-based reagent	*Drs FP*	*This study (Sigma-Aldrich)*	*RT-PCR primer*	*CTGTCCGGAAGATACAAGGG*
Sequence-based reagent	*Drs RP*	*This study (Sigma-Aldrich)*	*RT-PCR primer*	*TCGCACCAGCACTTCAGACT*
Sequence-based reagent	*Irc FP*	*This study (Sigma-Aldrich)*	*RT-PCR primer*	*TAGGCAAAAGCGACTGGAGGACA*
Sequence-based reagent	*Irc RP*	*This study (Sigma-Aldrich)*	*RT-PCR primer*	*GCAAGCTGGACTTAAGGATCTTC*
Antibody	*Anti-HA (Rabbit monoclonal)*	*Millipore*	*DW2, Millipore*	*WB (1:3000)*
Antibody	*Anti-His (Mouse monoclonal*	*SIGMA*	*H1029, SIGMA*	*WB (1:1000)*
Antibody	*Anti-GST (Mouse monoclonal)*	*Santa-Cruz-Biotechnology*	*sc53909, Santa-Cruz-Biotechnology*	*WB (1:5000)*
Software, Algorithm	*GraphPad Prism 8.0.2*	*Prism*		
Software, Algorithm	*ImageJ, Fiji*	*National Institutes of Health (Rueden etal.,2017, Schindelin et al.,2012, Schneider et al., 2012)*		

REFERENCE

Ferreiro MJ, Pérez C, Marchesano M, Ruiz S, Caputi A, Aguilera P, Barrio R, Cantera R. (2017) *Drosophila melanogaster* White Mutant w(1118) Undergo Retinal Degeneration. *Front Neurosci*. 11:732. doi: 10.3389/fnins.2017.00732.
