# Supplementary material for: SUMOylation of Arginyl tRNA Synthetase Modulates the Drosophila Innate Immune Response
Source: Front Cell Dev Biol. 2021 Sep 30;9:695630. doi: 10.3389/fcell.2021.695630 (PMC8514731; doi:10.3389/fcell.2021.695630)
Supplement: Supplementary Model S1 — Drosophila RRS structural model generated by the automated SWISS-MODEL server, in PDB format. [file Data_Sheet_2.PDF]

```
TITLE     SWISS-MODEL SERVER (https://swissmodel.expasy.org)
TITLE    2 DromeRRS
EXPDTA    THEORETICAL MODEL (SWISS-MODEL SERVER)
AUTHOR    SWISS-MODEL SERVER (SEE REFERENCE IN JRNL Records)
REVDAT   1   13-AUG-21 1MOD    1       16:49
JRNL        AUTH
A.WATERHOUSE,M.BERTONI,S.BIENERT,G.STUDER,G.TAURIELLO,
JRNL        AUTH 2 R.GUMIENNY,F.T.HEER,T.A.P.DE
BEER,C.REMPFER,L.BORDOLI,
JRNL        AUTH 3 R.LEPORE,T.SCHWEDE
JRNL        TITL   SWISS-MODEL: HOMOLOGY MODELLING OF PROTEIN
STRUCTURES AND
JRNL        TITL 2 COMPLEXES
JRNL        REF    NUCLEIC.ACIDS.RES..           V.  46 W296  2018
JRNL        PMID   29788355
JRNL        DOI    10.1093/nar/gky427
REMARK   1
REMARK   1 REFERENCE 1
REMARK   1  AUTH   S.BIENERT,A.WATERHOUSE,T.A.P.DE
BEER,G.TAURIELLO,G.STUDER,
REMARK   1  AUTH 2 L.BORDOLI,T.SCHWEDE
REMARK   1  TITL   THE SWISS-MODEL REPOSITORY - NEW FEATURES AND
FUNCTIONALITY
REMARK   1  REF    NUCLEIC.ACIDS.RES..           V.  45       2017
REMARK   1  REFN                   ISSN 0305-1048
REMARK   1  PMID   27899672
REMARK   1  DOI    10.1093/nar/gkw1132
REMARK   1
REMARK   1 REFERENCE 2
REMARK   1  AUTH   N.GUEX,M.C.PEITSCH,T.SCHWEDE
REMARK   1  TITL   AUTOMATED COMPARATIVE PROTEIN STRUCTURE MODELING
WITH
REMARK   1  TITL 2 SWISS-MODEL AND SWISS-PDBVIEWER: A HISTORICAL
PERSPECTIVE
REMARK   1  REF    ELECTROPHORESIS               V.  30       2009
REMARK   1  REFN                   ISSN 0173-0835
REMARK   1  PMID   19517507
REMARK   1  DOI    10.1002/elps.200900140
REMARK   1
REMARK   1 REFERENCE 3
REMARK   1  AUTH
G.STUDER,G.TAURIELLO,S.BIENERT,M.BIASINI,N.JOHNER,T.SCHWEDE
REMARK   1  TITL   PROMOD3 - A VERSATILE HOMOLOGY MODELLING TOOLBOX
REMARK   1  REF    PLOS COMP. BIOL.              V.  17       2021
REMARK   1  REFN                   ISSN
REMARK   1  PMID   33507980
REMARK   1  DOI    https://doi.org/10.1371/journal.pcbi.1008667
REMARK   1
REMARK   1 REFERENCE 4
REMARK   1  AUTH
G.STUDER,C.REMPFER,A.WATERHOUSE,R.GUMIENNY,J.HAAS,T.SCHWEDE
REMARK   1  TITL   QMEANDISCO - DISTANCE CONSTRAINTS APPLIED ON
MODEL QUALITY
REMARK   1  TITL 2 ESTIMATION
```

```
REMARK   1  REF    BIOINFORMATICS               V.  36       2020
REMARK   1  REFN                  ISSN
REMARK   1  PMID   31697312
REMARK   1  DOI    https://doi.org/10.1093/bioinformatics/btz828
REMARK   1
REMARK   1 REFERENCE 5
REMARK   1  AUTH   P.BENKERT,M.BIASINI,T.SCHWEDE
REMARK   1  TITL   TOWARD THE ESTIMATION OF THE ABSOLUTE QUALITY OF
INDIVIDUAL
REMARK   1  TITL 2 PROTEIN STRUCTURE MODELS
REMARK   1  REF    BIOINFORMATICS               V.  27       2011
REMARK   1  REFN                  ISSN 1367-4803
REMARK   1  PMID   21134891
REMARK   1  DOI    10.1093/bioinformatics/btq662
REMARK   1
REMARK   1 REFERENCE 6
REMARK   1  AUTH   M.BERTONI,F.KIEFER,M.BIASINI,L.BORDOLI,T.SCHWEDE
REMARK   1  TITL   MODELING PROTEIN QUATERNARY STRUCTURE OF HOMO-
AND
REMARK   1  TITL 2 HETERO-OLIGOMERS BEYOND BINARY INTERACTIONS BY
HOMOLOGY
REMARK   1  REF    SCI.REP.                     V.   7       2017
REMARK   1  REFN                  ISSN
REMARK   1  PMID   28874689
REMARK   1  DOI    10.1038/s41598-017-09654-8
REMARK   1
REMARK   1 DISCLAIMER
REMARK   1 The SWISS-MODEL SERVER produces theoretical models for
proteins.
REMARK   1 The results of any theoretical modelling procedure is
REMARK   1 NON-EXPERIMENTAL and MUST be considered with care. These
models may
REMARK   1 contain significant errors. This is especially true for
automated
REMARK   1 modeling since there is no human intervention during
model
REMARK   1 building. Please read the header section and the logfile
carefully
REMARK   1 to know what templates and alignments were used during
the model
REMARK   1 building process. All information by the SWISS-MODEL
SERVER is
REMARK   1 provided "AS-IS", without any warranty, expressed or
implied.
REMARK   2
REMARK   2 COPYRIGHT NOTICE
REMARK   2 This SWISS-MODEL protein model is copyright. It is
produced by the
REMARK   2 SWISS-MODEL server, developed by the Computational
Structural
REMARK   2 Biology Group at the SIB Swiss Institute of
Bioinformatics at the
REMARK   2 Biozentrum, University of Basel (https://
swissmodel.expasy.org). This
```

```
REMARK   2 model is licensed under the CC BY-SA 4.0 Creative Commons
REMARK   2 Attribution-ShareAlike 4.0 International License
REMARK   2 (https://creativecommons.org/licenses/by-sa/4.0/
legalcode), i.e. you
REMARK   2 can copy and redistribute the model in any medium or
format,
REMARK   2 transform and build upon the model for any purpose, even
REMARK   2 commercially, under the following terms:
REMARK   2 Attribution - You must give appropriate credit, provide a
link to
REMARK   2 the license, and indicate if changes were made. You may
do so in any
REMARK   2 reasonable manner, but not in any way that suggests the
licensor
REMARK   2 endorses you or your use. When you publish, patent or
distribute
REMARK   2 results that were fully or partially based on the model,
please cite
REMARK   2 the corresponding papers mentioned under JRNL.
REMARK   2 ShareAlike - If you remix, transform, or build upon the
material,
REMARK   2 you must distribute your contributions under the same
license as the
REMARK   2 original.
REMARK   2 No additional restrictions - you may not apply legal
terms or
REMARK   2 technological measures that legally restrict others from
doing
REMARK   2 anything the license permits.
REMARK   2 Find a human-readable summary of (and not a substitute
for) the
REMARK   2 CC BY-SA 4.0 license at this link:
REMARK   2 https://creativecommons.org/licenses/by-sa/4.0/
```
```
REMARK   3
REMARK   3 MODEL INFORMATION
REMARK   3  ENGIN   PROMOD3
REMARK   3  VERSN   3.2.0
REMARK   3  OSTAT   monomer
REMARK   3  OSRSN   MONOMER (USER)
REMARK   3  QSPRD   0.000
REMARK   3  GMQE    0.77
REMARK   3  QMNV    4.2.0
REMARK   3  QMNDG   0.81
REMARK   3  MODT    FALSE
REMARK   3
REMARK   3 MODEL LIGAND 1
REMARK   3  NAME    ARG
REMARK   3  BIND    A.201
REMARK   3  BIND 2  A.204
REMARK   3  BIND 3  A.206
REMARK   3  BIND 4  A.215
REMARK   3  BIND 5  A.241
REMARK   3  BIND 6  A.243
REMARK   3  BIND 7  A.390
```

```
REMARK   3  BIND 8  A.394
REMARK   3  BIND 9  A.412
REMARK   3  BIND 10  A.414
REMARK   3  BIND 11  A.418
REMARK   3  BIND 12  A.422
REMARK   3  BIND 13  _.1
REMARK   3
REMARK   3 TEMPLATE 1
REMARK   3  PDBID   4q2t
REMARK   3  CHAIN   A
REMARK   3  MMCIF   A
REMARK   3  PDBV    2021-08-06
REMARK   3  SMTLE   4q2t.1.A
REMARK   3  SMTLV   2021-08-11
REMARK   3  MTHD    X-RAY DIFFRACTION 2.40 A
REMARK   3  FOUND   HHblits
REMARK   3  GMQE    0.82
REMARK   3  SIM     0.47
REMARK   3  SID     59.90
REMARK   3  OSTAT   monomer
REMARK   3  LIGND   ARG
REMARK   3  ALN A TRG
MSELNMELKKLRELELKTQGLAARIQTAKSGEQLDVDLVQLQIENKKLKNRLFILKKS
REMARK   3  ALN A TRG
IAEESTAAGGDVSKPKESSSITEHLESVFRQAIASAFPEFRDTPVIIAPVNSTSAKFG
REMARK   3  ALN A TRG
DYQCNNAMGLSKKLKEKGINKAPRDIATELKGHCPASPIIEKLEIAGAGFVNVFLSKD
REMARK   3  ALN A TRG YASLALSNLLRNGVKPPE-
VIKKRVLVDFSSPNIAKQMHVGHLRSTIIGESLCRLLEF
REMARK   3  ALN A TRG
LQHDVIRINHLGDWGTQFGMLIAHLEDRFPNYLNESPPISDLQLFYKESKKRFDEDEE
REMARK   3  ALN A TRG
FKKRAYSRVVSLQKGVPNSIKAWELICNVSRKEFQTIYERLDISVKERGESFYQSRML
REMARK   3  ALN A TRG
SVVEYLRGKGLLEVDEGREIMWPDDTKTGIPLTIVKSDGGFTYDTSDMAAIRHRLEEE
REMARK   3  ALN A TRG
LCDWIIYVVDSGQSTHFNTIFKAAERSAILNPLSHRVDHVQFGVVLGEDGKKFKTRSG
REMARK   3  ALN A TRG
DTVKLSDLLDEGMKRSLQQLESRGRDKVLTPQELKDAQESLAYGCIKYSDLCHNRISD
REMARK   3  ALN A TRG
YIFSFDKMLEDRGNTAVYLLYTYTRICSIARNSGEDFTNLPEILKKTNIVLDHEKEWK
REMARK   3  ALN A TRG
LAKTLLKLHDILIKCSKELFLHFLCEFCFEVCTVFTEFYDSCYCIEKNKQ-GDIIGVN
REMARK   3  ALN A TRG HSRILLCEATAAVLRQCFYILGLKPVSKM
REMARK   3  ALN A TPL
----------------------------------------------------------
REMARK   3  ALN A TPL ----------------
MINIISRLQEVFGHAIKAAYPDLENPPLLV--TPSQQAKFG
REMARK   3  ALN A TPL
DYQCNSAMGISQMLKTKEQKVNPREIAENITKHLPDNECIEKVEIAGPGFINVHLRKD
REMARK   3  ALN A TPL
FVSEQLTSLLVNGVQLPALGENKKVIVDFSSPNIAKEMHVGHLRSTIIGESISRLFEF
REMARK   3  ALN A TPL
AGYDVLRLNHVGDWGTQFGMLIAHLQDKFPDYLTVSPPIGDLQVFYKESKKRFDTEEE
```

```
REMARK   3  ALN A TPL
FKKRAYQCVVLLQGKNPDITKAWKLICDVSRQELNKIYDALDVSLIERGESFYQDRMN
REMARK   3  ALN A TPL DIVKEFEDRGFVQVDDGRKIVFVPG--
CSIPLTIVKSDGGYTYDTSDLAAIKQRLFEE
REMARK   3  ALN A TPL
KADMIIYVVDNGQSVHFQTIFAAAQMIGWYDPKVTRVFHAGFGVVLGEDKKKFKTRSG
REMARK   3  ALN A TPL
ETVRLMDLLGEGLKRSMDKLKEKERDKVLTAEELNAAQTSVAYGCIKYADLSRNRLND
REMARK   3  ALN A TPL
YIFSFDKMLDDRGNTAAYLLYAFTRIRSIARLANIDEEMLQKAARETKILLDHEKEWK
REMARK   3  ALN A TPL
LGRCILRFPEILQKILDDLFLHTLCDYIYELATAFTEFYDSCYCVEKDRQTGKILKVN
REMARK   3  ALN A TPL MWRMLLCEAVAAVMAKGFDILGIKPVQRM
REMARK   3  ALN A OFF 19
ATOM      1  N   SER A  76      69.238  43.255 253.969  1.00  0.83
N
ATOM      2  CA  SER A  76      67.785  43.581 253.739  1.00  0.83
C
ATOM      3  C   SER A  76      67.250  42.861 252.527  1.00  0.83
C
ATOM      4  O   SER A  76      67.998  42.143 251.875  1.00  0.83
O
ATOM      5  CB  SER A  76      67.591  45.122 253.594  1.00  0.83
C
ATOM      6  OG  SER A  76      68.543  45.669 252.678  1.00  0.83
O
ATOM      7  N   SER A  77      65.937  42.985 252.244  1.00  0.83
N
ATOM      8  CA  SER A  77      65.293  42.243 251.172  1.00  0.83
C
ATOM      9  C   SER A  77      64.615  43.229 250.261  1.00  0.83
C
ATOM     10  O   SER A  77      63.893  44.110 250.722  1.00  0.83
O
ATOM     11  CB  SER A  77      64.198  41.255 251.663  1.00  0.83
C
ATOM     12  OG  SER A  77      64.777  40.053 252.164  1.00  0.83
O
ATOM     13  N   SER A  78      64.813  43.106 248.933  1.00  0.84
N
ATOM     14  CA  SER A  78      64.128  43.955 247.968  1.00  0.84
C
ATOM     15  C   SER A  78      62.715  43.461 247.760  1.00  0.84
C
ATOM     16  O   SER A  78      62.460  42.443 247.117  1.00  0.84
O
ATOM     17  CB  SER A  78      64.845  44.023 246.602  1.00  0.84
C
ATOM     18  OG  SER A  78      64.207  44.932 245.699  1.00  0.84
O
ATOM     19  N   ILE A  79      61.735  44.168 248.353  1.00  0.83
N
ATOM     20  CA  ILE A  79      60.342  43.758 248.353  1.00  0.83
C
```

ATOM	21	C	ILE	A	79	59.754	43.711	246.957	1.00	0.83	C
ATOM	22	O	ILE	A	79	59.049	42.767	246.612	1.00	0.83	O
ATOM	23	CB	ILE	A	79	59.508	44.604	249.307	1.00	0.83	C
ATOM	24	CG1	ILE	A	79	60.052	44.420	250.745	1.00	0.83	C
ATOM	25	CG2	ILE	A	79	58.020	44.198	249.203	1.00	0.83	C
ATOM	26	CD1	ILE	A	79	59.342	45.261	251.812	1.00	0.83	C
ATOM	27	N	THR	A	80	60.093	44.691	246.098	1.00	0.82	N
ATOM	28	CA	THR	A	80	59.726	44.698	244.686	1.00	0.82	C
ATOM	29	C	THR	A	80	60.220	43.470	243.953	1.00	0.82	C
ATOM	30	O	THR	A	80	59.426	42.771	243.342	1.00	0.82	O
ATOM	31	CB	THR	A	80	60.272	45.930	243.995	1.00	0.82	C
ATOM	32	OG1	THR	A	80	59.643	47.087	244.526	1.00	0.82	O
ATOM	33	CG2	THR	A	80	60.007	45.963	242.491	1.00	0.82	C
ATOM	34	N	GLU	A	81	61.513	43.098	244.076	1.00	0.78	N
ATOM	35	CA	GLU	A	81	62.068	41.910	243.443	1.00	0.78	C
ATOM	36	C	GLU	A	81	61.440	40.610	243.935	1.00	0.78	C
ATOM	37	O	GLU	A	81	61.159	39.688	243.168	1.00	0.78	O
ATOM	38	CB	GLU	A	81	63.590	41.823	243.676	1.00	0.78	C
ATOM	39	CG	GLU	A	81	64.412	42.911	242.942	1.00	0.78	C
ATOM	40	CD	GLU	A	81	65.884	42.928	243.356	1.00	0.78	C
ATOM	41	OE1	GLU	A	81	66.247	42.265	244.360	1.00	0.78	O
ATOM	42	OE2	GLU	A	81	66.650	43.670	242.694	1.00	0.78	O
ATOM	43	N	HIS	A	82	61.175	40.509	245.256	1.00	0.81	N
ATOM	44	CA	HIS	A	82	60.443	39.387	245.821	1.00	0.81	C
ATOM	45	C	HIS	A	82	59.016	39.292	245.315	1.00	0.81	C
ATOM	46	O	HIS	A	82	58.570	38.230	244.889	1.00	0.81	O
ATOM	47	CB	HIS	A	82	60.402	39.442	247.363	1.00	0.81	C

```
ATOM      48  CG  HIS A  82      61.712  39.099 247.997  1.00  0.81
C
ATOM      49  ND1 HIS A  82      62.199  37.818 247.851  1.00  0.81
N
ATOM      50  CD2 HIS A  82      62.566  39.845 248.743  1.00  0.81
C
ATOM      51  CE1 HIS A  82      63.343  37.805 248.504  1.00  0.81
C
ATOM      52  NE2 HIS A  82      63.615  39.008 249.067  1.00  0.81
N
ATOM      53  N   LEU A  83      58.266  40.410 245.294  1.00  0.84
N
ATOM      54  CA  LEU A  83      56.930  40.446 244.734  1.00  0.84
C
ATOM      55  C   LEU A  83      56.927  40.172 243.231  1.00  0.84
C
ATOM      56  O   LEU A  83      56.094  39.412 242.742  1.00  0.84
O
ATOM      57  CB  LEU A  83      56.195  41.765 245.076  1.00  0.84
C
ATOM      58  CG  LEU A  83      55.786  41.947 246.554  1.00  0.84
C
ATOM      59  CD1 LEU A  83      55.189  43.346 246.754  1.00  0.84
C
ATOM      60  CD2 LEU A  83      54.771  40.893 247.018  1.00  0.84
C
ATOM      61  N   GLU A  84      57.899  40.716 242.462  1.00  0.80
N
ATOM      62  CA  GLU A  84      58.091  40.435 241.046  1.00  0.80
C
ATOM      63  C   GLU A  84      58.329  38.957 240.807  1.00  0.80
C
ATOM      64  O   GLU A  84      57.720  38.360 239.930  1.00  0.80
O
ATOM      65  CB  GLU A  84      59.221  41.298 240.407  1.00  0.80
C
ATOM      66  CG  GLU A  84      58.833  42.789 240.168  1.00  0.80
C
ATOM      67  CD  GLU A  84      59.931  43.675 239.562  1.00  0.80
C
ATOM      68  OE1 GLU A  84      61.042  43.160 239.280  1.00  0.80
O
ATOM      69  OE2 GLU A  84      59.656  44.894 239.375  1.00  0.80
O
ATOM      70  N   SER A  85      59.147  38.287 241.639  1.00  0.85
N
ATOM      71  CA  SER A  85      59.282  36.838 241.611  1.00  0.85
C
ATOM      72  C   SER A  85      58.005  36.067 241.897  1.00  0.85
C
ATOM      73  O   SER A  85      57.696  35.125 241.173  1.00  0.85
O
ATOM      74  CB  SER A  85      60.370  36.330 242.586  1.00  0.85
C
```

```
ATOM     75  OG  SER A  85      61.670  36.542 242.043  1.00  0.85
O
ATOM     76  N   VAL A  86      57.219  36.453 242.924  1.00  0.85
N
ATOM     77  CA  VAL A  86      55.955  35.798 243.256  1.00  0.85
C
ATOM     78  C   VAL A  86      54.914  35.941 242.151  1.00  0.85
C
ATOM     79  O   VAL A  86      54.315  34.966 241.696  1.00  0.85
O
ATOM     80  CB  VAL A  86      55.383  36.341 244.569  1.00  0.85
C
ATOM     81  CG1 VAL A  86      53.989  35.756 244.875  1.00  0.85
C
ATOM     82  CG2 VAL A  86      56.321  35.980 245.736  1.00  0.85
C
ATOM     83  N   PHE A  87      54.709  37.170 241.632  1.00  0.82
N
ATOM     84  CA  PHE A  87      53.779  37.421 240.548  1.00  0.82
C
ATOM     85  C   PHE A  87      54.215  36.783 239.239  1.00  0.82
C
ATOM     86  O   PHE A  87      53.392  36.276 238.491  1.00  0.82
O
ATOM     87  CB  PHE A  87      53.500  38.932 240.371  1.00  0.82
C
ATOM     88  CG  PHE A  87      52.488  39.397 241.384  1.00  0.82
C
ATOM     89  CD1 PHE A  87      51.120  39.142 241.184  1.00  0.82
C
ATOM     90  CD2 PHE A  87      52.878  40.091 242.539  1.00  0.82
C
ATOM     91  CE1 PHE A  87      50.170  39.571 242.119  1.00  0.82
C
ATOM     92  CE2 PHE A  87      51.935  40.497 243.488  1.00  0.82
C
ATOM     93  CZ  PHE A  87      50.579  40.238 243.277  1.00  0.82
C
ATOM     94  N   ARG A  88      55.525  36.741 238.942  1.00  0.74
N
ATOM     95  CA  ARG A  88      56.093  36.049 237.802  1.00  0.74
C
ATOM     96  C   ARG A  88      55.763  34.562 237.767  1.00  0.74
C
ATOM     97  O   ARG A  88      55.389  34.031 236.723  1.00  0.74
O
ATOM     98  CB  ARG A  88      57.624  36.241 237.877  1.00  0.74
C
ATOM     99  CG  ARG A  88      58.491  35.549 236.811  1.00  0.74
C
ATOM    100  CD  ARG A  88      59.992  35.560 237.146  1.00  0.74
C
ATOM    101  NE  ARG A  88      60.214  34.726 238.378  1.00  0.74
N
```

ATOM	102	CZ	ARG	A	88	60.283	33.388	238.398	1.00	0.74	C
ATOM	103	NH1	ARG	A	88	60.536	32.772	239.551	1.00	0.74	N
ATOM	104	NH2	ARG	A	88	60.120	32.659	237.298	1.00	0.74	N
ATOM	105	N	GLN	A	89	55.851	33.852	238.916	1.00	0.78	N
ATOM	106	CA	GLN	A	89	55.416	32.467	239.014	1.00	0.78	C
ATOM	107	C	GLN	A	89	53.918	32.308	238.799	1.00	0.78	C
ATOM	108	O	GLN	A	89	53.469	31.414	238.085	1.00	0.78	O
ATOM	109	CB	GLN	A	89	55.820	31.850	240.376	1.00	0.78	C
ATOM	110	CG	GLN	A	89	57.349	31.678	240.531	1.00	0.78	C
ATOM	111	CD	GLN	A	89	57.734	31.150	241.917	1.00	0.78	C
ATOM	112	OE1	GLN	A	89	57.149	31.486	242.939	1.00	0.78	O
ATOM	113	NE2	GLN	A	89	58.799	30.313	241.972	1.00	0.78	N
ATOM	114	N	ALA	A	90	53.112	33.213	239.383	1.00	0.87	N
ATOM	115	CA	ALA	A	90	51.681	33.242	239.200	1.00	0.87	C
ATOM	116	C	ALA	A	90	51.224	33.494	237.766	1.00	0.87	C
ATOM	117	O	ALA	A	90	50.361	32.786	237.254	1.00	0.87	O
ATOM	118	CB	ALA	A	90	51.087	34.294	240.148	1.00	0.87	C
ATOM	119	N	ILE	A	91	51.832	34.465	237.047	1.00	0.84	N
ATOM	120	CA	ILE	A	91	51.558	34.692	235.631	1.00	0.84	C
ATOM	121	C	ILE	A	91	51.911	33.484	234.781	1.00	0.84	C
ATOM	122	O	ILE	A	91	51.136	33.085	233.919	1.00	0.84	O
ATOM	123	CB	ILE	A	91	52.292	35.918	235.069	1.00	0.84	C
ATOM	124	CG1	ILE	A	91	51.967	37.232	235.830	1.00	0.84	C
ATOM	125	CG2	ILE	A	91	52.013	36.080	233.551	1.00	0.84	C
ATOM	126	CD1	ILE	A	91	53.022	38.319	235.577	1.00	0.84	C
ATOM	127	N	ALA	A	92	53.075	32.849	235.021	1.00	0.85	N
ATOM	128	CA	ALA	A	92	53.507	31.674	234.296	1.00	0.85	C

ATOM	129	C	ALA	A	92	52.579	30.476	234.482	1.00	0.85	C
ATOM	130	O	ALA	A	92	52.274	29.751	233.543	1.00	0.85	O
ATOM	131	CB	ALA	A	92	54.953	31.342	234.711	1.00	0.85	C
ATOM	132	N	SER	A	93	52.075	30.252	235.707	1.00	0.81	N
ATOM	133	CA	SER	A	93	51.043	29.257	235.972	1.00	0.81	C
ATOM	134	C	SER	A	93	49.691	29.542	235.338	1.00	0.81	C
ATOM	135	O	SER	A	93	49.071	28.650	234.764	1.00	0.81	O
ATOM	136	CB	SER	A	93	50.805	29.108	237.487	1.00	0.81	C
ATOM	137	OG	SER	A	93	51.949	28.545	238.129	1.00	0.81	O
ATOM	138	N	ALA	A	94	49.188	30.791	235.427	1.00	0.88	N
ATOM	139	CA	ALA	A	94	47.933	31.179	234.813	1.00	0.88	C
ATOM	140	C	ALA	A	94	47.976	31.195	233.290	1.00	0.88	C
ATOM	141	O	ALA	A	94	47.071	30.721	232.606	1.00	0.88	O
ATOM	142	CB	ALA	A	94	47.523	32.571	235.328	1.00	0.88	C
ATOM	143	N	PHE	A	95	49.068	31.730	232.719	1.00	0.79	N
ATOM	144	CA	PHE	A	95	49.209	31.939	231.296	1.00	0.79	C
ATOM	145	C	PHE	A	95	50.556	31.432	230.775	1.00	0.79	C
ATOM	146	O	PHE	A	95	51.453	32.234	230.501	1.00	0.79	O
ATOM	147	CB	PHE	A	95	49.141	33.448	230.982	1.00	0.79	C
ATOM	148	CG	PHE	A	95	47.824	34.031	231.372	1.00	0.79	C
ATOM	149	CD1	PHE	A	95	46.680	33.723	230.624	1.00	0.79	C
ATOM	150	CD2	PHE	A	95	47.708	34.847	232.507	1.00	0.79	C
ATOM	151	CE1	PHE	A	95	45.434	34.231	230.995	1.00	0.79	C
ATOM	152	CE2	PHE	A	95	46.461	35.343	232.893	1.00	0.79	C
ATOM	153	CZ	PHE	A	95	45.331	35.043	232.130	1.00	0.79	C
ATOM	154	N	PRO	A	96	50.749	30.129	230.595	1.00	0.76	N
ATOM	155	CA	PRO	A	96	52.082	29.548	230.414	1.00	0.76	C

ATOM	156	C	PRO	A	96	52.759	29.890	229.107	1.00	0.76	C
ATOM	157	O	PRO	A	96	53.985	29.894	229.049	1.00	0.76	O
ATOM	158	CB	PRO	A	96	51.851	28.032	230.566	1.00	0.76	C
ATOM	159	CG	PRO	A	96	50.342	27.833	230.393	1.00	0.76	C
ATOM	160	CD	PRO	A	96	49.774	29.111	230.988	1.00	0.76	C
ATOM	161	N	GLU	A	97	51.989	30.185	228.049	1.00	0.70	N
ATOM	162	CA	GLU	A	97	52.516	30.374	226.713	1.00	0.70	C
ATOM	163	C	GLU	A	97	52.855	31.831	226.427	1.00	0.70	C
ATOM	164	O	GLU	A	97	53.188	32.232	225.312	1.00	0.70	O
ATOM	165	CB	GLU	A	97	51.448	29.908	225.697	1.00	0.70	C
ATOM	166	CG	GLU	A	97	51.175	28.385	225.762	1.00	0.70	C
ATOM	167	CD	GLU	A	97	52.433	27.560	225.475	1.00	0.70	C
ATOM	168	OE1	GLU	A	97	53.198	27.942	224.551	1.00	0.70	O
ATOM	169	OE2	GLU	A	97	52.634	26.547	226.191	1.00	0.70	O
ATOM	170	N	PHE	A	98	52.766	32.694	227.453	1.00	0.74	N
ATOM	171	CA	PHE	A	98	52.839	34.129	227.293	1.00	0.74	C
ATOM	172	C	PHE	A	98	54.244	34.612	227.614	1.00	0.74	C
ATOM	173	O	PHE	A	98	54.798	34.360	228.679	1.00	0.74	O
ATOM	174	CB	PHE	A	98	51.823	34.845	228.212	1.00	0.74	C
ATOM	175	CG	PHE	A	98	50.375	34.696	227.790	1.00	0.74	C
ATOM	176	CD1	PHE	A	98	49.836	33.667	226.992	1.00	0.74	C
ATOM	177	CD2	PHE	A	98	49.471	35.601	228.357	1.00	0.74	C
ATOM	178	CE1	PHE	A	98	48.454	33.560	226.791	1.00	0.74	C
ATOM	179	CE2	PHE	A	98	48.099	35.529	228.127	1.00	0.74	C
ATOM	180	CZ	PHE	A	98	47.589	34.495	227.350	1.00	0.74	C
ATOM	181	N	ARG	A	99	54.874	35.334	226.666	1.00	0.61	N
ATOM	182	CA	ARG	A	99	56.286	35.656	226.741	1.00	0.61	C

ATOM 183 C ARG A 99 56.554 37.128 226.992 1.00 0.61
C
ATOM 184 O ARG A 99 56.013 37.993 226.302 1.00 0.61
O
ATOM 185 CB ARG A 99 56.986 35.303 225.412 1.00 0.61
C
ATOM 186 CG ARG A 99 56.925 33.808 225.056 1.00 0.61
C
ATOM 187 CD ARG A 99 57.660 33.494 223.753 1.00 0.61
C
ATOM 188 NE ARG A 99 57.521 32.022 223.532 1.00 0.61
N
ATOM 189 CZ ARG A 99 58.073 31.360 222.507 1.00 0.61
C
ATOM 190 NH1 ARG A 99 57.879 30.048 222.400 1.00 0.61
N
ATOM 191 NH2 ARG A 99 58.812 31.986 221.593 1.00 0.61
N
ATOM 192 N ASP A 100 57.432 37.406 227.984 1.00 0.72
N
ATOM 193 CA ASP A 100 57.778 38.727 228.471 1.00 0.72
C
ATOM 194 C ASP A 100 56.560 39.542 228.899 1.00 0.72
C
ATOM 195 O ASP A 100 56.204 40.588 228.357 1.00 0.72
O
ATOM 196 CB ASP A 100 58.780 39.423 227.523 1.00 0.72
C
ATOM 197 CG ASP A 100 59.563 40.516 228.234 1.00 0.72
C
ATOM 198 OD1 ASP A 100 59.894 41.519 227.555 1.00 0.72
O
ATOM 199 OD2 ASP A 100 59.862 40.332 229.441 1.00 0.72
O
ATOM 200 N THR A 101 55.818 38.986 229.878 1.00 0.76
N
ATOM 201 CA THR A 101 54.682 39.646 230.496 1.00 0.76
C
ATOM 202 C THR A 101 55.151 40.781 231.387 1.00 0.76
C
ATOM 203 O THR A 101 55.995 40.529 232.247 1.00 0.76
O
ATOM 204 CB THR A 101 53.848 38.718 231.360 1.00 0.76
C
ATOM 205 OG1 THR A 101 53.292 37.687 230.555 1.00 0.76
O
ATOM 206 CG2 THR A 101 52.672 39.432 232.053 1.00 0.76
C
ATOM 207 N PRO A 102 54.663 42.013 231.276 1.00 0.82
N
ATOM 208 CA PRO A 102 54.984 43.102 232.192 1.00 0.82
C
ATOM 209 C PRO A 102 54.755 42.791 233.665 1.00 0.82
C

```
ATOM    210  O   PRO A 102      53.678  42.346 234.041  1.00  0.82
O
ATOM    211  CB  PRO A 102      54.108  44.273 231.709  1.00  0.82
C
ATOM    212  CG  PRO A 102      52.994  43.602 230.907  1.00  0.82
C
ATOM    213  CD  PRO A 102      53.717  42.441 230.251  1.00  0.82
C
ATOM    214  N   VAL A 103      55.757  43.047 234.524  1.00  0.80
N
ATOM    215  CA  VAL A 103      55.738  42.726 235.942  1.00  0.80
C
ATOM    216  C   VAL A 103      55.742  43.978 236.822  1.00  0.80
C
ATOM    217  O   VAL A 103      56.340  44.007 237.886  1.00  0.80
O
ATOM    218  CB  VAL A 103      56.860  41.759 236.333  1.00  0.80
C
ATOM    219  CG1 VAL A 103      56.525  40.353 235.790  1.00  0.80
C
ATOM    220  CG2 VAL A 103      58.237  42.241 235.829  1.00  0.80
C
ATOM    221  N   ILE A 104      55.062  45.072 236.401  1.00  0.76
N
ATOM    222  CA  ILE A 104      55.077  46.377 237.076  1.00  0.76
C
ATOM    223  C   ILE A 104      54.578  46.355 238.522  1.00  0.76
C
ATOM    224  O   ILE A 104      53.376  46.308 238.792  1.00  0.76
O
ATOM    225  CB  ILE A 104      54.324  47.426 236.243  1.00  0.76
C
ATOM    226  CG1 ILE A 104      55.068  47.613 234.905  1.00  0.76
C
ATOM    227  CG2 ILE A 104      54.042  48.768 236.974  1.00  0.76
C
ATOM    228  CD1 ILE A 104      54.464  48.653 233.958  1.00  0.76
C
ATOM    229  N   ILE A 105      55.540  46.412 239.472  1.00  0.79
N
ATOM    230  CA  ILE A 105      55.315  46.468 240.910  1.00  0.79
C
ATOM    231  C   ILE A 105      55.991  47.689 241.526  1.00  0.79
C
ATOM    232  O   ILE A 105      55.401  48.360 242.366  1.00  0.79
O
ATOM    233  CB  ILE A 105      55.794  45.175 241.570  1.00  0.79
C
ATOM    234  CG1 ILE A 105      54.825  44.037 241.168  1.00  0.79
C
ATOM    235  CG2 ILE A 105      55.861  45.297 243.110  1.00  0.79
C
ATOM    236  CD1 ILE A 105      55.290  42.627 241.511  1.00  0.79
C
```

ATOM 237 N ALA A 106 57.217 48.087 241.109 1.00 0.51
N
ATOM 238 CA ALA A 106 57.847 49.289 241.654 1.00 0.51
C
ATOM 239 C ALA A 106 57.129 50.626 241.399 1.00 0.51
C
ATOM 240 O ALA A 106 56.987 51.408 242.341 1.00 0.51
O
ATOM 241 CB ALA A 106 59.330 49.392 241.250 1.00 0.51
C
ATOM 242 N PRO A 107 56.604 50.944 240.211 1.00 0.36
N
ATOM 243 CA PRO A 107 55.843 52.174 239.950 1.00 0.36
C
ATOM 244 C PRO A 107 54.615 52.370 240.801 1.00 0.36
C
ATOM 245 O PRO A 107 54.057 53.462 240.801 1.00 0.36
O
ATOM 246 CB PRO A 107 55.408 52.008 238.486 1.00 0.36
C
ATOM 247 CG PRO A 107 56.563 51.229 237.876 1.00 0.36
C
ATOM 248 CD PRO A 107 56.921 50.237 238.969 1.00 0.36
C
ATOM 249 N VAL A 108 54.143 51.301 241.456 1.00 0.59
N
ATOM 250 CA VAL A 108 52.856 51.241 242.088 1.00 0.59
C
ATOM 251 C VAL A 108 52.954 50.916 243.569 1.00 0.59
C
ATOM 252 O VAL A 108 52.008 50.440 244.195 1.00 0.59
O
ATOM 253 CB VAL A 108 51.973 50.254 241.376 1.00 0.59
C
ATOM 254 CG1 VAL A 108 51.679 50.723 239.945 1.00 0.59
C
ATOM 255 CG2 VAL A 108 52.574 48.836 241.409 1.00 0.59
C
ATOM 256 N ASN A 109 54.125 51.180 244.189 1.00 0.79
N
ATOM 257 CA ASN A 109 54.358 51.076 245.620 1.00 0.79
C
ATOM 258 C ASN A 109 53.429 52.027 246.385 1.00 0.79
C
ATOM 259 O ASN A 109 53.615 53.240 246.372 1.00 0.79
O
ATOM 260 CB ASN A 109 55.869 51.359 245.871 1.00 0.79
C
ATOM 261 CG ASN A 109 56.328 51.141 247.309 1.00 0.79
C
ATOM 262 OD1 ASN A 109 55.604 51.327 248.285 1.00 0.79
O
ATOM 263 ND2 ASN A 109 57.627 50.789 247.472 1.00 0.79
N

```
ATOM     264  N   SER A 110      52.375  51.478 247.029  1.00  0.78
N
ATOM     265  CA  SER A 110      51.153  52.212 247.343  1.00  0.78
C
ATOM     266  C   SER A 110      51.322  53.449 248.213  1.00  0.78
C
ATOM     267  O   SER A 110      51.673  53.377 249.393  1.00  0.78
O
ATOM     268  CB  SER A 110      50.065  51.324 248.003  1.00  0.78
C
ATOM     269  OG  SER A 110      48.805  51.996 248.075  1.00  0.78
O
ATOM     270  N   THR A 111      51.028  54.627 247.624  1.00  0.67
N
ATOM     271  CA  THR A 111      50.982  55.930 248.278  1.00  0.67
C
ATOM     272  C   THR A 111      49.937  56.031 249.367  1.00  0.67
C
ATOM     273  O   THR A 111      50.150  56.648 250.407  1.00  0.67
O
ATOM     274  CB  THR A 111      50.700  57.045 247.277  1.00  0.67
C
ATOM     275  OG1 THR A 111      51.619  56.964 246.202  1.00  0.67
O
ATOM     276  CG2 THR A 111      50.858  58.446 247.881  1.00  0.67
C
ATOM     277  N   SER A 112      48.754  55.424 249.158  1.00  0.67
N
ATOM     278  CA  SER A 112      47.653  55.513 250.101  1.00  0.67
C
ATOM     279  C   SER A 112      47.388  54.144 250.670  1.00  0.67
C
ATOM     280  O   SER A 112      47.074  53.192 249.961  1.00  0.67
O
ATOM     281  CB  SER A 112      46.351  56.045 249.449  1.00  0.67
C
ATOM     282  OG  SER A 112      45.271  56.149 250.383  1.00  0.67
O
ATOM     283  N   ALA A 113      47.471  54.018 252.009  1.00  0.79
N
ATOM     284  CA  ALA A 113      47.346  52.765 252.726  1.00  0.79
C
ATOM     285  C   ALA A 113      45.968  52.109 252.630  1.00  0.79
C
ATOM     286  O   ALA A 113      45.775  50.963 253.017  1.00  0.79
O
ATOM     287  CB  ALA A 113      47.731  53.019 254.196  1.00  0.79
C
ATOM     288  N   LYS A 114      44.988  52.826 252.046  1.00  0.71
N
ATOM     289  CA  LYS A 114      43.695  52.315 251.651  1.00  0.71
C
ATOM     290  C   LYS A 114      43.779  51.172 250.657  1.00  0.71
C
```

```
ATOM     291  O   LYS A 114      42.959  50.263 250.683  1.00  0.71
O
ATOM     292  CB  LYS A 114      42.879  53.446 250.978  1.00  0.71
C
ATOM     293  CG  LYS A 114      42.322  54.495 251.951  1.00  0.71
C
ATOM     294  CD  LYS A 114      41.121  53.947 252.739  1.00  0.71
C
ATOM     295  CE  LYS A 114      40.455  54.993 253.634  1.00  0.71
C
ATOM     296  NZ  LYS A 114      39.269  54.406 254.297  1.00  0.71
N
ATOM     297  N   PHE A 115      44.770  51.221 249.748  1.00  0.71
N
ATOM     298  CA  PHE A 115      44.870  50.317 248.620  1.00  0.71
C
ATOM     299  C   PHE A 115      46.014  49.338 248.825  1.00  0.71
C
ATOM     300  O   PHE A 115      46.638  48.871 247.876  1.00  0.71
O
ATOM     301  CB  PHE A 115      45.102  51.082 247.287  1.00  0.71
C
ATOM     302  CG  PHE A 115      44.205  52.285 247.155  1.00  0.71
C
ATOM     303  CD1 PHE A 115      42.804  52.180 247.204  1.00  0.71
C
ATOM     304  CD2 PHE A 115      44.783  53.553 246.982  1.00  0.71
C
ATOM     305  CE1 PHE A 115      42.000  53.324 247.102  1.00  0.71
C
ATOM     306  CE2 PHE A 115      43.983  54.697 246.878  1.00  0.71
C
ATOM     307  CZ  PHE A 115      42.590  54.584 246.946  1.00  0.71
C
ATOM     308  N   GLY A 116      46.349  49.023 250.090  1.00  0.85
N
ATOM     309  CA  GLY A 116      47.445  48.126 250.421  1.00  0.85
C
ATOM     310  C   GLY A 116      48.783  48.808 250.552  1.00  0.85
C
ATOM     311  O   GLY A 116      48.898  50.012 250.793  1.00  0.85
O
ATOM     312  N   ASP A 117      49.861  48.017 250.431  1.00  0.83
N
ATOM     313  CA  ASP A 117      51.218  48.475 250.638  1.00  0.83
C
ATOM     314  C   ASP A 117      51.993  48.483 249.337  1.00  0.83
C
ATOM     315  O   ASP A 117      52.851  49.331 249.114  1.00  0.83
O
ATOM     316  CB  ASP A 117      51.932  47.542 251.637  1.00  0.83
C
ATOM     317  CG  ASP A 117      51.125  47.436 252.917  1.00  0.83
C
```

ATOM	318	OD1	ASP	A	117	50.837	48.495	253.545	1.00	0.83	O
ATOM	319	OD2	ASP	A	117	50.784	46.286	253.273	1.00	0.83	O
ATOM	320	N	TYR	A	118	51.639	47.574	248.421	1.00	0.83	N
ATOM	321	CA	TYR	A	118	52.162	47.511	247.076	1.00	0.83	C
ATOM	322	C	TYR	A	118	50.980	47.243	246.172	1.00	0.83	C
ATOM	323	O	TYR	A	118	49.876	46.942	246.618	1.00	0.83	O
ATOM	324	CB	TYR	A	118	53.238	46.399	246.874	1.00	0.83	C
ATOM	325	CG	TYR	A	118	54.570	46.794	247.451	1.00	0.83	C
ATOM	326	CD1	TYR	A	118	54.834	46.653	248.824	1.00	0.83	C
ATOM	327	CD2	TYR	A	118	55.589	47.281	246.615	1.00	0.83	C
ATOM	328	CE1	TYR	A	118	56.063	47.062	249.362	1.00	0.83	C
ATOM	329	CE2	TYR	A	118	56.831	47.655	247.145	1.00	0.83	C
ATOM	330	CZ	TYR	A	118	57.059	47.567	248.521	1.00	0.83	C
ATOM	331	OH	TYR	A	118	58.305	47.973	249.038	1.00	0.83	O
ATOM	332	N	GLN	A	119	51.193	47.363	244.862	1.00	0.80	N
ATOM	333	CA	GLN	A	119	50.204	47.054	243.869	1.00	0.80	C
ATOM	334	C	GLN	A	119	50.934	46.283	242.781	1.00	0.80	C
ATOM	335	O	GLN	A	119	52.156	46.191	242.777	1.00	0.80	O
ATOM	336	CB	GLN	A	119	49.590	48.354	243.328	1.00	0.80	C
ATOM	337	CG	GLN	A	119	48.329	48.240	242.454	1.00	0.80	C
ATOM	338	CD	GLN	A	119	47.843	49.643	242.099	1.00	0.80	C
ATOM	339	OE1	GLN	A	119	48.483	50.409	241.387	1.00	0.80	O
ATOM	340	NE2	GLN	A	119	46.672	50.014	242.669	1.00	0.80	N
ATOM	341	N	CYS	A	120	50.207	45.693	241.826	1.00	0.83	N
ATOM	342	CA	CYS	A	120	50.801	45.114	240.654	1.00	0.83	C
ATOM	343	C	CYS	A	120	49.899	45.401	239.471	1.00	0.83	C
ATOM	344	O	CYS	A	120	48.688	45.196	239.518	1.00	0.83	O

ATOM 345 CB CYS A 120 51.008 43.598 240.810 1.00 0.83
C
ATOM 346 SG CYS A 120 51.957 42.901 239.437 1.00 0.83
S
ATOM 347 N ASN A 121 50.496 45.884 238.364 1.00 0.80
N
ATOM 348 CA ASN A 121 49.753 46.356 237.210 1.00 0.80
C
ATOM 349 C ASN A 121 49.892 45.397 236.044 1.00 0.80
C
ATOM 350 O ASN A 121 49.571 45.720 234.900 1.00 0.80
O
ATOM 351 CB ASN A 121 50.252 47.754 236.768 1.00 0.80
C
ATOM 352 CG ASN A 121 49.572 48.836 237.595 1.00 0.80
C
ATOM 353 OD1 ASN A 121 48.968 48.589 238.632 1.00 0.80
O
ATOM 354 ND2 ASN A 121 49.651 50.093 237.096 1.00 0.80
N
ATOM 355 N ASN A 122 50.367 44.169 236.306 1.00 0.80
N
ATOM 356 CA ASN A 122 50.644 43.176 235.289 1.00 0.80
C
ATOM 357 C ASN A 122 49.451 42.790 234.431 1.00 0.80
C
ATOM 358 O ASN A 122 49.560 42.726 233.214 1.00 0.80
O
ATOM 359 CB ASN A 122 51.335 41.929 235.900 1.00 0.80
C
ATOM 360 CG ASN A 122 50.571 41.273 237.049 1.00 0.80
C
ATOM 361 OD1 ASN A 122 49.456 41.616 237.422 1.00 0.80
O
ATOM 362 ND2 ASN A 122 51.258 40.309 237.712 1.00 0.80
N
ATOM 363 N ALA A 123 48.264 42.596 235.030 1.00 0.83
N
ATOM 364 CA ALA A 123 47.043 42.316 234.311 1.00 0.83
C
ATOM 365 C ALA A 123 46.633 43.389 233.301 1.00 0.83
C
ATOM 366 O ALA A 123 46.244 43.087 232.174 1.00 0.83
O
ATOM 367 CB ALA A 123 45.931 42.112 235.348 1.00 0.83
C
ATOM 368 N MET A 124 46.773 44.680 233.671 1.00 0.78
N
ATOM 369 CA MET A 124 46.550 45.813 232.789 1.00 0.78
C
ATOM 370 C MET A 124 47.496 45.858 231.607 1.00 0.78
C
ATOM 371 O MET A 124 47.129 46.243 230.503 1.00 0.78
O

```
ATOM    372  CB  MET A 124      46.744  47.166 233.519  1.00  0.78
C
ATOM    373  CG  MET A 124      45.925  47.366 234.806  1.00  0.78
C
ATOM    374  SD  MET A 124      44.131  47.124 234.655  1.00  0.78
S
ATOM    375  CE  MET A 124      43.805  48.198 233.230  1.00  0.78
C
ATOM    376  N   GLY A 125      48.777  45.511 231.837  1.00  0.83
N
ATOM    377  CA  GLY A 125      49.771  45.424 230.781  1.00  0.83
C
ATOM    378  C   GLY A 125      49.584  44.217 229.902  1.00  0.83
C
ATOM    379  O   GLY A 125      49.868  44.255 228.710  1.00  0.83
O
ATOM    380  N   LEU A 126      49.106  43.099 230.480  1.00  0.80
N
ATOM    381  CA  LEU A 126      48.874  41.863 229.771  1.00  0.80
C
ATOM    382  C   LEU A 126      47.732  41.933 228.779  1.00  0.80
C
ATOM    383  O   LEU A 126      47.861  41.480 227.645  1.00  0.80
O
ATOM    384  CB  LEU A 126      48.695  40.664 230.734  1.00  0.80
C
ATOM    385  CG  LEU A 126      48.773  39.285 230.044  1.00  0.80
C
ATOM    386  CD1 LEU A 126      50.140  39.056 229.374  1.00  0.80
C
ATOM    387  CD2 LEU A 126      48.471  38.160 231.048  1.00  0.80
C
ATOM    388  N   SER A 127      46.591  42.555 229.144  1.00  0.76
N
ATOM    389  CA  SER A 127      45.511  42.786 228.191  1.00  0.76
C
ATOM    390  C   SER A 127      45.925  43.689 227.051  1.00  0.76
C
ATOM    391  O   SER A 127      45.605  43.436 225.896  1.00  0.76
O
ATOM    392  CB  SER A 127      44.228  43.364 228.827  1.00  0.76
C
ATOM    393  OG  SER A 127      44.492  44.568 229.544  1.00  0.76
O
ATOM    394  N   LYS A 128      46.716  44.734 227.354  1.00  0.71
N
ATOM    395  CA  LYS A 128      47.312  45.613 226.374  1.00  0.71
C
ATOM    396  C   LYS A 128      48.267  44.909 225.414  1.00  0.71
C
ATOM    397  O   LYS A 128      48.347  45.287 224.251  1.00  0.71
O
ATOM    398  CB  LYS A 128      47.976  46.830 227.062  1.00  0.71
C
```

ATOM 399 CG LYS A 128 46.959 47.765 227.741 1.00 0.71
C
ATOM 400 CD LYS A 128 47.647 48.873 228.556 1.00 0.71
C
ATOM 401 CE LYS A 128 46.671 49.700 229.395 1.00 0.71
C
ATOM 402 NZ LYS A 128 47.401 50.797 230.067 1.00 0.71
N
ATOM 403 N LYS A 129 48.993 43.856 225.857 1.00 0.70
N
ATOM 404 CA LYS A 129 49.781 43.008 224.975 1.00 0.70
C
ATOM 405 C LYS A 129 48.951 42.205 223.982 1.00 0.70
C
ATOM 406 O LYS A 129 49.332 42.063 222.823 1.00 0.70
O
ATOM 407 CB LYS A 129 50.685 42.020 225.765 1.00 0.70
C
ATOM 408 CG LYS A 129 51.627 41.189 224.869 1.00 0.70
C
ATOM 409 CD LYS A 129 52.495 40.187 225.646 1.00 0.70
C
ATOM 410 CE LYS A 129 53.358 39.300 224.741 1.00 0.70
C
ATOM 411 NZ LYS A 129 54.393 40.116 224.071 1.00 0.70
N
ATOM 412 N LEU A 130 47.800 41.651 224.415 1.00 0.69
N
ATOM 413 CA LEU A 130 46.993 40.770 223.584 1.00 0.69
C
ATOM 414 C LEU A 130 45.894 41.537 222.870 1.00 0.69
C
ATOM 415 O LEU A 130 44.985 40.965 222.274 1.00 0.69
O
ATOM 416 CB LEU A 130 46.296 39.670 224.419 1.00 0.69
C
ATOM 417 CG LEU A 130 47.182 38.875 225.393 1.00 0.69
C
ATOM 418 CD1 LEU A 130 46.297 37.821 226.068 1.00 0.69
C
ATOM 419 CD2 LEU A 130 48.395 38.215 224.719 1.00 0.69
C
ATOM 420 N LYS A 131 45.938 42.873 222.951 1.00 0.61
N
ATOM 421 CA LYS A 131 44.970 43.747 222.348 1.00 0.61
C
ATOM 422 C LYS A 131 45.059 43.749 220.832 1.00 0.61
C
ATOM 423 O LYS A 131 46.029 44.226 220.246 1.00 0.61
O
ATOM 424 CB LYS A 131 45.176 45.180 222.871 1.00 0.61
C
ATOM 425 CG LYS A 131 44.035 46.140 222.533 1.00 0.61
C

ATOM 426 CD LYS A 131 44.434 47.569 222.904 1.00 0.61
C
ATOM 427 CE LYS A 131 43.253 48.525 222.855 1.00 0.61
C
ATOM 428 NZ LYS A 131 43.687 49.851 223.333 1.00 0.61
N
ATOM 429 N GLU A 132 44.011 43.246 220.162 1.00 0.56
N
ATOM 430 CA GLU A 132 43.947 43.203 218.723 1.00 0.56
C
ATOM 431 C GLU A 132 42.893 44.168 218.222 1.00 0.56
C
ATOM 432 O GLU A 132 42.287 44.943 218.959 1.00 0.56
O
ATOM 433 CB GLU A 132 43.621 41.785 218.198 1.00 0.56
C
ATOM 434 CG GLU A 132 44.706 40.723 218.500 1.00 0.56
C
ATOM 435 CD GLU A 132 44.413 39.401 217.787 1.00 0.56
C
ATOM 436 OE1 GLU A 132 43.388 39.344 217.055 1.00 0.56
O
ATOM 437 OE2 GLU A 132 45.224 38.455 217.945 1.00 0.56
O
ATOM 438 N LYS A 133 42.661 44.154 216.896 1.00 0.55
N
ATOM 439 CA LYS A 133 41.681 44.984 216.229 1.00 0.55
C
ATOM 440 C LYS A 133 40.253 44.702 216.665 1.00 0.55
C
ATOM 441 O LYS A 133 39.440 45.612 216.782 1.00 0.55
O
ATOM 442 CB LYS A 133 41.783 44.821 214.693 1.00 0.55
C
ATOM 443 CG LYS A 133 43.206 45.004 214.140 1.00 0.55
C
ATOM 444 CD LYS A 133 43.229 44.903 212.607 1.00 0.55
C
ATOM 445 CE LYS A 133 44.634 45.050 212.018 1.00 0.55
C
ATOM 446 NZ LYS A 133 44.573 44.993 210.541 1.00 0.55
N
ATOM 447 N GLY A 134 39.917 43.414 216.897 1.00 0.53
N
ATOM 448 CA GLY A 134 38.546 43.002 217.164 1.00 0.53
C
ATOM 449 C GLY A 134 38.304 42.526 218.565 1.00 0.53
C
ATOM 450 O GLY A 134 37.159 42.366 218.972 1.00 0.53
O
ATOM 451 N ILE A 135 39.370 42.241 219.338 1.00 0.69
N
ATOM 452 CA ILE A 135 39.236 41.621 220.648 1.00 0.69
C

ATOM 453 C ILE A 135 40.169 42.299 221.639 1.00 0.69
C
ATOM 454 O ILE A 135 41.350 42.523 221.381 1.00 0.69
O
ATOM 455 CB ILE A 135 39.498 40.108 220.622 1.00 0.69
C
ATOM 456 CG1 ILE A 135 38.483 39.393 219.693 1.00 0.69
C
ATOM 457 CG2 ILE A 135 39.447 39.504 222.048 1.00 0.69
C
ATOM 458 CD1 ILE A 135 38.783 37.910 219.449 1.00 0.69
C
ATOM 459 N ASN A 136 39.632 42.658 222.824 1.00 0.65
N
ATOM 460 CA ASN A 136 40.401 43.167 223.939 1.00 0.65
C
ATOM 461 C ASN A 136 39.914 42.439 225.193 1.00 0.65
C
ATOM 462 O ASN A 136 38.742 42.523 225.551 1.00 0.65
O
ATOM 463 CB ASN A 136 40.180 44.702 224.031 1.00 0.65
C
ATOM 464 CG ASN A 136 41.096 45.379 225.040 1.00 0.65
C
ATOM 465 OD1 ASN A 136 41.983 44.787 225.641 1.00 0.65
O
ATOM 466 ND2 ASN A 136 40.886 46.707 225.224 1.00 0.65
N
ATOM 467 N LYS A 137 40.791 41.665 225.870 1.00 0.75
N
ATOM 468 CA LYS A 137 40.432 40.934 227.078 1.00 0.75
C
ATOM 469 C LYS A 137 40.256 41.855 228.279 1.00 0.75
C
ATOM 470 O LYS A 137 41.022 42.796 228.477 1.00 0.75
O
ATOM 471 CB LYS A 137 41.466 39.813 227.408 1.00 0.75
C
ATOM 472 CG LYS A 137 41.072 38.914 228.600 1.00 0.75
C
ATOM 473 CD LYS A 137 41.991 37.702 228.854 1.00 0.75
C
ATOM 474 CE LYS A 137 41.493 36.874 230.058 1.00 0.75
C
ATOM 475 NZ LYS A 137 42.407 35.769 230.411 1.00 0.75
N
ATOM 476 N ALA A 138 39.244 41.603 229.137 1.00 0.80
N
ATOM 477 CA ALA A 138 39.081 42.336 230.374 1.00 0.80
C
ATOM 478 C ALA A 138 40.260 42.164 231.349 1.00 0.80
C
ATOM 479 O ALA A 138 40.659 41.029 231.625 1.00 0.80
O

ATOM	480	CB	ALA	A	138	37.772	41.920	231.059	1.00	0.80	C
ATOM	481	N	PRO	A	139	40.861	43.215	231.919	1.00	0.85	N
ATOM	482	CA	PRO	A	139	42.021	43.057	232.788	1.00	0.85	C
ATOM	483	C	PRO	A	139	41.660	42.383	234.095	1.00	0.85	C
ATOM	484	O	PRO	A	139	42.493	41.720	234.700	1.00	0.85	O
ATOM	485	CB	PRO	A	139	42.544	44.483	233.015	1.00	0.85	C
ATOM	486	CG	PRO	A	139	41.962	45.322	231.874	1.00	0.85	C
ATOM	487	CD	PRO	A	139	40.669	44.605	231.499	1.00	0.85	C
ATOM	488	N	ARG	A	140	40.415	42.566	234.561	1.00	0.70	N
ATOM	489	CA	ARG	A	140	39.902	42.023	235.800	1.00	0.70	C
ATOM	490	C	ARG	A	140	39.891	40.507	235.831	1.00	0.70	C
ATOM	491	O	ARG	A	140	40.280	39.887	236.818	1.00	0.70	O
ATOM	492	CB	ARG	A	140	38.488	42.594	236.036	1.00	0.70	C
ATOM	493	CG	ARG	A	140	37.970	42.403	237.470	1.00	0.70	C
ATOM	494	CD	ARG	A	140	36.730	43.256	237.757	1.00	0.70	C
ATOM	495	NE	ARG	A	140	36.408	43.126	239.217	1.00	0.70	N
ATOM	496	CZ	ARG	A	140	37.013	43.825	240.186	1.00	0.70	C
ATOM	497	NH1	ARG	A	140	37.852	44.823	239.938	1.00	0.70	N
ATOM	498	NH2	ARG	A	140	36.784	43.518	241.463	1.00	0.70	N
ATOM	499	N	ASP	A	141	39.491	39.878	234.712	1.00	0.76	N
ATOM	500	CA	ASP	A	141	39.534	38.447	234.512	1.00	0.76	C
ATOM	501	C	ASP	A	141	40.955	37.912	234.569	1.00	0.76	C
ATOM	502	O	ASP	A	141	41.239	36.930	235.252	1.00	0.76	O
ATOM	503	CB	ASP	A	141	38.886	38.086	233.159	1.00	0.76	C
ATOM	504	CG	ASP	A	141	37.444	38.569	233.088	1.00	0.76	C
ATOM	505	OD1	ASP	A	141	36.831	38.844	234.150	1.00	0.76	O
ATOM	506	OD2	ASP	A	141	36.966	38.716	231.936	1.00	0.76	O

ATOM 507 N ILE A 142 41.911 38.628 233.927 1.00 0.81
N
ATOM 508 CA ILE A 142 43.330 38.316 234.012 1.00 0.81
C
ATOM 509 C ILE A 142 43.819 38.378 235.445 1.00 0.81
C
ATOM 510 O ILE A 142 44.439 37.445 235.938 1.00 0.81
O
ATOM 511 CB ILE A 142 44.178 39.260 233.154 1.00 0.81
C
ATOM 512 CG1 ILE A 142 43.766 39.181 231.668 1.00 0.81
C
ATOM 513 CG2 ILE A 142 45.684 38.960 233.329 1.00 0.81
C
ATOM 514 CD1 ILE A 142 44.521 40.172 230.776 1.00 0.81
C
ATOM 515 N ALA A 143 43.479 39.450 236.183 1.00 0.88
N
ATOM 516 CA ALA A 143 43.838 39.619 237.571 1.00 0.88
C
ATOM 517 C ALA A 143 43.294 38.506 238.464 1.00 0.88
C
ATOM 518 O ALA A 143 43.980 38.020 239.361 1.00 0.88
O
ATOM 519 CB ALA A 143 43.390 41.018 238.028 1.00 0.88
C
ATOM 520 N THR A 144 42.060 38.034 238.195 1.00 0.83
N
ATOM 521 CA THR A 144 41.469 36.854 238.827 1.00 0.83
C
ATOM 522 C THR A 144 42.247 35.581 238.578 1.00 0.83
C
ATOM 523 O THR A 144 42.557 34.852 239.521 1.00 0.83
O
ATOM 524 CB THR A 144 40.029 36.660 238.380 1.00 0.83
C
ATOM 525 OG1 THR A 144 39.228 37.742 238.836 1.00 0.83
O
ATOM 526 CG2 THR A 144 39.379 35.384 238.932 1.00 0.83
C
ATOM 527 N GLU A 145 42.647 35.309 237.318 1.00 0.81
N
ATOM 528 CA GLU A 145 43.490 34.174 236.980 1.00 0.81
C
ATOM 529 C GLU A 145 44.855 34.265 237.664 1.00 0.81
C
ATOM 530 O GLU A 145 45.311 33.330 238.313 1.00 0.81
O
ATOM 531 CB GLU A 145 43.636 34.053 235.433 1.00 0.81
C
ATOM 532 CG GLU A 145 42.303 33.678 234.720 1.00 0.81
C
ATOM 533 CD GLU A 145 42.256 33.966 233.221 1.00 0.81
C

ATOM 534 OE1 GLU A 145 42.621 33.101 232.389 1.00 0.81
O
ATOM 535 OE2 GLU A 145 41.848 35.097 232.838 1.00 0.81
O
ATOM 536 N LEU A 146 45.509 35.445 237.630 1.00 0.82
N
ATOM 537 CA LEU A 146 46.797 35.663 238.266 1.00 0.82
C
ATOM 538 C LEU A 146 46.793 35.483 239.771 1.00 0.82
C
ATOM 539 O LEU A 146 47.651 34.812 240.337 1.00 0.82
O
ATOM 540 CB LEU A 146 47.320 37.097 237.999 1.00 0.82
C
ATOM 541 CG LEU A 146 47.592 37.455 236.527 1.00 0.82
C
ATOM 542 CD1 LEU A 146 48.105 38.891 236.399 1.00 0.82
C
ATOM 543 CD2 LEU A 146 48.530 36.471 235.840 1.00 0.82
C
ATOM 544 N LYS A 147 45.803 36.063 240.474 1.00 0.78
N
ATOM 545 CA LYS A 147 45.688 35.919 241.909 1.00 0.78
C
ATOM 546 C LYS A 147 45.364 34.497 242.326 1.00 0.78
C
ATOM 547 O LYS A 147 45.845 34.013 243.349 1.00 0.78
O
ATOM 548 CB LYS A 147 44.652 36.905 242.487 1.00 0.78
C
ATOM 549 CG LYS A 147 44.562 36.857 244.019 1.00 0.78
C
ATOM 550 CD LYS A 147 43.616 37.908 244.605 1.00 0.78
C
ATOM 551 CE LYS A 147 42.140 37.668 244.310 1.00 0.78
C
ATOM 552 NZ LYS A 147 41.360 38.713 245.002 1.00 0.78
N
ATOM 553 N GLY A 148 44.574 33.772 241.506 1.00 0.83
N
ATOM 554 CA GLY A 148 44.200 32.392 241.790 1.00 0.83
C
ATOM 555 C GLY A 148 45.328 31.410 241.607 1.00 0.83
C
ATOM 556 O GLY A 148 45.246 30.270 242.052 1.00 0.83
O
ATOM 557 N HIS A 149 46.435 31.854 240.989 1.00 0.79
N
ATOM 558 CA HIS A 149 47.631 31.058 240.822 1.00 0.79
C
ATOM 559 C HIS A 149 48.812 31.592 241.611 1.00 0.79
C
ATOM 560 O HIS A 149 49.958 31.229 241.344 1.00 0.79
O

ATOM 561 CB HIS A 149 48.017 30.959 239.333 1.00 0.79
C
ATOM 562 CG HIS A 149 47.182 29.965 238.593 1.00 0.79
C
ATOM 563 ND1 HIS A 149 46.147 30.389 237.792 1.00 0.79
N
ATOM 564 CD2 HIS A 149 47.291 28.613 238.539 1.00 0.79
C
ATOM 565 CE1 HIS A 149 45.642 29.299 237.264 1.00 0.79
C
ATOM 566 NE2 HIS A 149 46.298 28.189 237.682 1.00 0.79
N
ATOM 567 N CYS A 150 48.590 32.454 242.623 1.00 0.84
N
ATOM 568 CA CYS A 150 49.660 32.914 243.495 1.00 0.84
C
ATOM 569 C CYS A 150 50.302 31.783 244.316 1.00 0.84
C
ATOM 570 O CYS A 150 49.578 31.072 245.015 1.00 0.84
O
ATOM 571 CB CYS A 150 49.147 34.019 244.449 1.00 0.84
C
ATOM 572 SG CYS A 150 50.490 34.912 245.318 1.00 0.84
S
ATOM 573 N PRO A 151 51.615 31.541 244.285 1.00 0.81
N
ATOM 574 CA PRO A 151 52.227 30.496 245.089 1.00 0.81
C
ATOM 575 C PRO A 151 52.285 30.884 246.552 1.00 0.81
C
ATOM 576 O PRO A 151 52.179 32.061 246.898 1.00 0.81
O
ATOM 577 CB PRO A 151 53.636 30.376 244.492 1.00 0.81
C
ATOM 578 CG PRO A 151 53.956 31.802 244.048 1.00 0.81
C
ATOM 579 CD PRO A 151 52.610 32.296 243.520 1.00 0.81
C
ATOM 580 N ALA A 152 52.451 29.895 247.452 1.00 0.79
N
ATOM 581 CA ALA A 152 52.660 30.167 248.856 1.00 0.79
C
ATOM 582 C ALA A 152 53.929 30.980 249.097 1.00 0.79
C
ATOM 583 O ALA A 152 54.998 30.672 248.574 1.00 0.79
O
ATOM 584 CB ALA A 152 52.693 28.857 249.663 1.00 0.79
C
ATOM 585 N SER A 153 53.825 32.066 249.884 1.00 0.78
N
ATOM 586 CA SER A 153 54.910 33.014 250.025 1.00 0.78
C
ATOM 587 C SER A 153 54.973 33.508 251.455 1.00 0.78
C

ATOM 588 O SER A 153 54.007 33.308 252.193 1.00 0.78
O
ATOM 589 CB SER A 153 54.814 34.197 249.008 1.00 0.78
C
ATOM 590 OG SER A 153 53.892 35.227 249.378 1.00 0.78
O
ATOM 591 N PRO A 154 56.059 34.142 251.916 1.00 0.81
N
ATOM 592 CA PRO A 154 56.146 34.492 253.324 1.00 0.81
C
ATOM 593 C PRO A 154 55.793 35.946 253.519 1.00 0.81
C
ATOM 594 O PRO A 154 55.701 36.386 254.661 1.00 0.81
O
ATOM 595 CB PRO A 154 57.606 34.195 253.693 1.00 0.81
C
ATOM 596 CG PRO A 154 58.392 34.428 252.402 1.00 0.81
C
ATOM 597 CD PRO A 154 57.393 34.066 251.295 1.00 0.81
C
ATOM 598 N ILE A 155 55.629 36.729 252.435 1.00 0.83
N
ATOM 599 CA ILE A 155 55.516 38.172 252.564 1.00 0.83
C
ATOM 600 C ILE A 155 54.128 38.725 252.290 1.00 0.83
C
ATOM 601 O ILE A 155 53.810 39.839 252.701 1.00 0.83
O
ATOM 602 CB ILE A 155 56.535 38.901 251.693 1.00 0.83
C
ATOM 603 CG1 ILE A 155 56.276 38.794 250.175 1.00 0.83
C
ATOM 604 CG2 ILE A 155 57.939 38.370 252.042 1.00 0.83
C
ATOM 605 CD1 ILE A 155 57.208 39.696 249.359 1.00 0.83
C
ATOM 606 N ILE A 156 53.241 37.961 251.626 1.00 0.83
N
ATOM 607 CA ILE A 156 51.916 38.430 251.248 1.00 0.83
C
ATOM 608 C ILE A 156 50.870 37.936 252.236 1.00 0.83
C
ATOM 609 O ILE A 156 50.704 36.738 252.442 1.00 0.83
O
ATOM 610 CB ILE A 156 51.539 37.985 249.831 1.00 0.83
C
ATOM 611 CG1 ILE A 156 52.544 38.552 248.797 1.00 0.83
C
ATOM 612 CG2 ILE A 156 50.097 38.425 249.495 1.00 0.83
C
ATOM 613 CD1 ILE A 156 52.278 38.128 247.347 1.00 0.83
C
ATOM 614 N GLU A 157 50.128 38.870 252.865 1.00 0.81
N

ATOM 615 CA GLU A 157 48.989 38.557 253.710 1.00 0.81
C
ATOM 616 C GLU A 157 47.762 38.252 252.864 1.00 0.81
C
ATOM 617 O GLU A 157 47.116 37.216 252.985 1.00 0.81
O
ATOM 618 CB GLU A 157 48.695 39.803 254.573 1.00 0.81
C
ATOM 619 CG GLU A 157 47.524 39.684 255.576 1.00 0.81
C
ATOM 620 CD GLU A 157 47.962 38.924 256.822 1.00 0.81
C
ATOM 621 OE1 GLU A 157 48.006 37.672 256.761 1.00 0.81
O
ATOM 622 OE2 GLU A 157 48.285 39.604 257.832 1.00 0.81
O
ATOM 623 N LYS A 158 47.440 39.154 251.910 1.00 0.80
N
ATOM 624 CA LYS A 158 46.334 38.941 251.005 1.00 0.80
C
ATOM 625 C LYS A 158 46.457 39.852 249.799 1.00 0.80
C
ATOM 626 O LYS A 158 47.191 40.841 249.820 1.00 0.80
O
ATOM 627 CB LYS A 158 44.946 39.159 251.677 1.00 0.80
C
ATOM 628 CG LYS A 158 44.618 40.627 251.989 1.00 0.80
C
ATOM 629 CD LYS A 158 43.194 40.866 252.514 1.00 0.80
C
ATOM 630 CE LYS A 158 42.929 42.373 252.578 1.00 0.80
C
ATOM 631 NZ LYS A 158 41.505 42.709 252.780 1.00 0.80
N
ATOM 632 N LEU A 159 45.711 39.533 248.717 1.00 0.85
N
ATOM 633 CA LEU A 159 45.679 40.323 247.500 1.00 0.85
C
ATOM 634 C LEU A 159 44.243 40.659 247.086 1.00 0.85
C
ATOM 635 O LEU A 159 43.332 39.824 247.129 1.00 0.85
O
ATOM 636 CB LEU A 159 46.347 39.589 246.310 1.00 0.85
C
ATOM 637 CG LEU A 159 47.798 39.123 246.516 1.00 0.85
C
ATOM 638 CD1 LEU A 159 48.274 38.322 245.298 1.00 0.85
C
ATOM 639 CD2 LEU A 159 48.758 40.274 246.815 1.00 0.85
C
ATOM 640 N GLU A 160 44.000 41.905 246.642 1.00 0.82
N
ATOM 641 CA GLU A 160 42.687 42.405 246.270 1.00 0.82
C

ATOM 642 C GLU A 160 42.694 42.822 244.805 1.00 0.82
C
ATOM 643 O GLU A 160 43.719 43.225 244.279 1.00 0.82
O
ATOM 644 CB GLU A 160 42.265 43.579 247.180 1.00 0.82
C
ATOM 645 CG GLU A 160 42.365 43.203 248.678 1.00 0.82
C
ATOM 646 CD GLU A 160 42.086 44.380 249.602 1.00 0.82
C
ATOM 647 OE1 GLU A 160 42.581 45.489 249.309 1.00 0.82
O
ATOM 648 OE2 GLU A 160 41.421 44.152 250.646 1.00 0.82
O
ATOM 649 N ILE A 161 41.563 42.701 244.069 1.00 0.82
N
ATOM 650 CA ILE A 161 41.516 43.128 242.665 1.00 0.82
C
ATOM 651 C ILE A 161 40.616 44.344 242.550 1.00 0.82
C
ATOM 652 O ILE A 161 39.399 44.282 242.747 1.00 0.82
O
ATOM 653 CB ILE A 161 41.030 42.058 241.694 1.00 0.82
C
ATOM 654 CG1 ILE A 161 41.960 40.829 241.713 1.00 0.82
C
ATOM 655 CG2 ILE A 161 40.976 42.631 240.254 1.00 0.82
C
ATOM 656 CD1 ILE A 161 41.255 39.575 241.196 1.00 0.82
C
ATOM 657 N ALA A 162 41.210 45.498 242.214 1.00 0.81
N
ATOM 658 CA ALA A 162 40.584 46.791 242.312 1.00 0.81
C
ATOM 659 C ALA A 162 40.443 47.451 240.955 1.00 0.81
C
ATOM 660 O ALA A 162 41.260 47.276 240.052 1.00 0.81
O
ATOM 661 CB ALA A 162 41.405 47.689 243.249 1.00 0.81
C
ATOM 662 N GLY A 163 39.356 48.235 240.770 1.00 0.76
N
ATOM 663 CA GLY A 163 39.064 48.921 239.513 1.00 0.76
C
ATOM 664 C GLY A 163 39.049 48.044 238.284 1.00 0.76
C
ATOM 665 O GLY A 163 38.423 46.986 238.253 1.00 0.76
O
ATOM 666 N ALA A 164 39.749 48.486 237.225 1.00 0.79
N
ATOM 667 CA ALA A 164 39.854 47.790 235.964 1.00 0.79
C
ATOM 668 C ALA A 164 40.503 46.415 236.069 1.00 0.79
C

ATOM 669 O ALA A 164 40.113 45.477 235.385 1.00 0.79
O
ATOM 670 CB ALA A 164 40.648 48.683 234.997 1.00 0.79
C
ATOM 671 N GLY A 165 41.500 46.280 236.962 1.00 0.83
N
ATOM 672 CA GLY A 165 42.255 45.049 237.132 1.00 0.83
C
ATOM 673 C GLY A 165 43.555 45.280 237.839 1.00 0.83
C
ATOM 674 O GLY A 165 44.516 44.543 237.645 1.00 0.83
O
ATOM 675 N PHE A 166 43.628 46.323 238.691 1.00 0.79
N
ATOM 676 CA PHE A 166 44.772 46.546 239.552 1.00 0.79
C
ATOM 677 C PHE A 166 44.821 45.508 240.651 1.00 0.79
C
ATOM 678 O PHE A 166 43.804 45.199 241.271 1.00 0.79
O
ATOM 679 CB PHE A 166 44.736 47.930 240.245 1.00 0.79
C
ATOM 680 CG PHE A 166 44.877 49.075 239.287 1.00 0.79
C
ATOM 681 CD1 PHE A 166 45.864 49.082 238.289 1.00 0.79
C
ATOM 682 CD2 PHE A 166 44.072 50.214 239.447 1.00 0.79
C
ATOM 683 CE1 PHE A 166 46.047 50.203 237.471 1.00 0.79
C
ATOM 684 CE2 PHE A 166 44.246 51.335 238.627 1.00 0.79
C
ATOM 685 CZ PHE A 166 45.237 51.330 237.638 1.00 0.79
C
ATOM 686 N VAL A 167 46.006 44.954 240.945 1.00 0.84
N
ATOM 687 CA VAL A 167 46.129 43.956 241.989 1.00 0.84
C
ATOM 688 C VAL A 167 46.758 44.607 243.197 1.00 0.84
C
ATOM 689 O VAL A 167 47.952 44.881 243.222 1.00 0.84
O
ATOM 690 CB VAL A 167 46.993 42.787 241.544 1.00 0.84
C
ATOM 691 CG1 VAL A 167 47.122 41.729 242.660 1.00 0.84
C
ATOM 692 CG2 VAL A 167 46.386 42.169 240.271 1.00 0.84
C
ATOM 693 N ASN A 168 45.981 44.879 244.255 1.00 0.83
N
ATOM 694 CA ASN A 168 46.506 45.502 245.457 1.00 0.83
C
ATOM 695 C ASN A 168 47.086 44.444 246.389 1.00 0.83
C

```
ATOM    696  O   ASN A 168      46.520  43.367 246.565  1.00  0.83
O
ATOM    697  CB  ASN A 168      45.433  46.324 246.205  1.00  0.83
C
ATOM    698  CG  ASN A 168      45.023  47.549 245.397  1.00  0.83
C
ATOM    699  OD1 ASN A 168      45.755  48.135 244.604  1.00  0.83
O
ATOM    700  ND2 ASN A 168      43.757  47.982 245.605  1.00  0.83
N
ATOM    701  N   VAL A 169      48.251  44.737 246.997  1.00  0.86
N
ATOM    702  CA  VAL A 169      49.043  43.800 247.779  1.00  0.86
C
ATOM    703  C   VAL A 169      49.165  44.307 249.203  1.00  0.86
C
ATOM    704  O   VAL A 169      49.652  45.414 249.440  1.00  0.86
O
ATOM    705  CB  VAL A 169      50.478  43.648 247.247  1.00  0.86
C
ATOM    706  CG1 VAL A 169      51.271  42.586 248.044  1.00  0.86
C
ATOM    707  CG2 VAL A 169      50.489  43.325 245.740  1.00  0.86
C
ATOM    708  N   PHE A 170      48.759  43.496 250.198  1.00  0.83
N
ATOM    709  CA  PHE A 170      48.937  43.827 251.603  1.00  0.83
C
ATOM    710  C   PHE A 170      49.998  42.911 252.190  1.00  0.83
C
ATOM    711  O   PHE A 170      49.968  41.691 252.005  1.00  0.83
O
ATOM    712  CB  PHE A 170      47.601  43.678 252.376  1.00  0.83
C
ATOM    713  CG  PHE A 170      47.679  44.267 253.762  1.00  0.83
C
ATOM    714  CD1 PHE A 170      47.801  43.450 254.897  1.00  0.83
C
ATOM    715  CD2 PHE A 170      47.645  45.658 253.941  1.00  0.83
C
ATOM    716  CE1 PHE A 170      47.886  44.004 256.179  1.00  0.83
C
ATOM    717  CE2 PHE A 170      47.730  46.221 255.219  1.00  0.83
C
ATOM    718  CZ  PHE A 170      47.851  45.393 256.339  1.00  0.83
C
ATOM    719  N   LEU A 171      50.995  43.489 252.884  1.00  0.85
N
ATOM    720  CA  LEU A 171      52.100  42.718 253.421  1.00  0.85
C
ATOM    721  C   LEU A 171      51.759  42.065 254.751  1.00  0.85
C
ATOM    722  O   LEU A 171      51.021  42.599 255.574  1.00  0.85
O
```

ATOM 723 CB LEU A 171 53.388 43.553 253.629 1.00 0.85
C
ATOM 724 CG LEU A 171 54.053 44.114 252.354 1.00 0.85
C
ATOM 725 CD1 LEU A 171 55.227 45.022 252.756 1.00 0.85
C
ATOM 726 CD2 LEU A 171 54.555 43.002 251.418 1.00 0.85
C
ATOM 727 N SER A 172 52.326 40.868 255.009 1.00 0.85
N
ATOM 728 CA SER A 172 52.239 40.224 256.319 1.00 0.85
C
ATOM 729 C SER A 172 52.884 41.053 257.426 1.00 0.85
C
ATOM 730 O SER A 172 53.994 41.575 257.284 1.00 0.85
O
ATOM 731 CB SER A 172 52.805 38.770 256.299 1.00 0.85
C
ATOM 732 OG SER A 172 52.946 38.191 257.602 1.00 0.85
O
ATOM 733 N LYS A 173 52.188 41.184 258.581 1.00 0.82
N
ATOM 734 CA LYS A 173 52.674 41.915 259.742 1.00 0.82
C
ATOM 735 C LYS A 173 53.939 41.304 260.330 1.00 0.82
C
ATOM 736 O LYS A 173 54.886 42.020 260.663 1.00 0.82
O
ATOM 737 CB LYS A 173 51.597 42.011 260.858 1.00 0.82
C
ATOM 738 CG LYS A 173 50.336 42.823 260.495 1.00 0.82
C
ATOM 739 CD LYS A 173 50.491 44.333 260.770 1.00 0.82
C
ATOM 740 CE LYS A 173 49.243 45.168 260.449 1.00 0.82
C
ATOM 741 NZ LYS A 173 49.523 46.618 260.589 1.00 0.82
N
ATOM 742 N ASP A 174 53.992 39.955 260.415 1.00 0.82
N
ATOM 743 CA ASP A 174 55.143 39.203 260.869 1.00 0.82
C
ATOM 744 C ASP A 174 56.364 39.450 260.007 1.00 0.82
C
ATOM 745 O ASP A 174 57.450 39.727 260.507 1.00 0.82
O
ATOM 746 CB ASP A 174 54.833 37.687 260.840 1.00 0.82
C
ATOM 747 CG ASP A 174 54.082 37.261 262.090 1.00 0.82
C
ATOM 748 OD1 ASP A 174 54.448 37.743 263.192 1.00 0.82
O
ATOM 749 OD2 ASP A 174 53.159 36.424 261.949 1.00 0.82
O

ATOM 750 N TYR A 175 56.202 39.433 258.668 1.00 0.82
N
ATOM 751 CA TYR A 175 57.291 39.688 257.743 1.00 0.82
C
ATOM 752 C TYR A 175 57.921 41.064 257.956 1.00 0.82
C
ATOM 753 O TYR A 175 59.140 41.189 258.075 1.00 0.82
O
ATOM 754 CB TYR A 175 56.768 39.520 256.281 1.00 0.82
C
ATOM 755 CG TYR A 175 57.729 40.048 255.246 1.00 0.82
C
ATOM 756 CD1 TYR A 175 59.052 39.580 255.204 1.00 0.82
C
ATOM 757 CD2 TYR A 175 57.348 41.105 254.401 1.00 0.82
C
ATOM 758 CE1 TYR A 175 59.988 40.182 254.355 1.00 0.82
C
ATOM 759 CE2 TYR A 175 58.285 41.705 253.547 1.00 0.82
C
ATOM 760 CZ TYR A 175 59.610 41.253 253.542 1.00 0.82
C
ATOM 761 OH TYR A 175 60.583 41.886 252.749 1.00 0.82
O
ATOM 762 N ALA A 176 57.094 42.120 258.055 1.00 0.90
N
ATOM 763 CA ALA A 176 57.585 43.456 258.289 1.00 0.90
C
ATOM 764 C ALA A 176 58.266 43.616 259.646 1.00 0.90
C
ATOM 765 O ALA A 176 59.337 44.210 259.749 1.00 0.90
O
ATOM 766 CB ALA A 176 56.429 44.450 258.096 1.00 0.90
C
ATOM 767 N SER A 177 57.690 43.031 260.716 1.00 0.86
N
ATOM 768 CA SER A 177 58.304 43.025 262.041 1.00 0.86
C
ATOM 769 C SER A 177 59.641 42.299 262.089 1.00 0.86
C
ATOM 770 O SER A 177 60.650 42.827 262.567 1.00 0.86
O
ATOM 771 CB SER A 177 57.344 42.398 263.084 1.00 0.86
C
ATOM 772 OG SER A 177 57.923 42.361 264.391 1.00 0.86
O
ATOM 773 N LEU A 178 59.711 41.082 261.519 1.00 0.84
N
ATOM 774 CA LEU A 178 60.929 40.302 261.463 1.00 0.84
C
ATOM 775 C LEU A 178 62.027 40.953 260.640 1.00 0.84
C
ATOM 776 O LEU A 178 63.193 40.958 261.029 1.00 0.84
O

```
ATOM    777  CB  LEU A 178      60.642  38.881 260.935  1.00  0.84
C
ATOM    778  CG  LEU A 178      59.779  38.017 261.880  1.00  0.84
C
ATOM    779  CD1 LEU A 178      59.342  36.733 261.161  1.00  0.84
C
ATOM    780  CD2 LEU A 178      60.492  37.669 263.196  1.00  0.84
C
ATOM    781  N   ALA A 179      61.688  41.558 259.487  1.00  0.89
N
ATOM    782  CA  ALA A 179      62.625  42.300 258.675  1.00  0.89
C
ATOM    783  C   ALA A 179      63.192  43.549 259.352  1.00  0.89
C
ATOM    784  O   ALA A 179      64.389  43.825 259.245  1.00  0.89
O
ATOM    785  CB  ALA A 179      61.984  42.614 257.317  1.00  0.89
C
ATOM    786  N   LEU A 180      62.369  44.303 260.116  1.00  0.88
N
ATOM    787  CA  LEU A 180      62.848  45.372 260.983  1.00  0.88
C
ATOM    788  C   LEU A 180      63.804  44.867 262.064  1.00  0.88
C
ATOM    789  O   LEU A 180      64.869  45.436 262.287  1.00  0.88
O
ATOM    790  CB  LEU A 180      61.666  46.111 261.653  1.00  0.88
C
ATOM    791  CG  LEU A 180      60.810  46.981 260.711  1.00  0.88
C
ATOM    792  CD1 LEU A 180      59.524  47.388 261.439  1.00  0.88
C
ATOM    793  CD2 LEU A 180      61.565  48.224 260.220  1.00  0.88
C
ATOM    794  N   SER A 181      63.488  43.728 262.722  1.00  0.86
N
ATOM    795  CA  SER A 181      64.400  43.065 263.662  1.00  0.86
C
ATOM    796  C   SER A 181      65.711  42.640 263.009  1.00  0.86
C
ATOM    797  O   SER A 181      66.793  42.841 263.557  1.00  0.86
O
ATOM    798  CB  SER A 181      63.725  41.838 264.358  1.00  0.86
C
ATOM    799  OG  SER A 181      64.594  41.061 265.202  1.00  0.86
O
ATOM    800  N   ASN A 182      65.650  42.080 261.783  1.00  0.84
N
ATOM    801  CA  ASN A 182      66.813  41.681 261.014  1.00  0.84
C
ATOM    802  C   ASN A 182      67.767  42.822 260.671  1.00  0.84
C
ATOM    803  O   ASN A 182      68.977  42.655 260.817  1.00  0.84
O
```

ATOM 804 CB ASN A 182 66.396 40.983 259.689 1.00 0.84
C
ATOM 805 CG ASN A 182 65.842 39.578 259.927 1.00 0.84
C
ATOM 806 OD1 ASN A 182 66.201 38.889 260.882 1.00 0.84
O
ATOM 807 ND2 ASN A 182 64.992 39.105 258.983 1.00 0.84
N
ATOM 808 N LEU A 183 67.267 44.000 260.223 1.00 0.85
N
ATOM 809 CA LEU A 183 68.125 45.145 259.944 1.00 0.85
C
ATOM 810 C LEU A 183 68.764 45.708 261.191 1.00 0.85
C
ATOM 811 O LEU A 183 69.904 46.150 261.172 1.00 0.85
O
ATOM 812 CB LEU A 183 67.440 46.251 259.083 1.00 0.85
C
ATOM 813 CG LEU A 183 66.533 47.275 259.805 1.00 0.85
C
ATOM 814 CD1 LEU A 183 67.303 48.509 260.311 1.00 0.85
C
ATOM 815 CD2 LEU A 183 65.410 47.756 258.876 1.00 0.85
C
ATOM 816 N LEU A 184 68.051 45.714 262.328 1.00 0.86
N
ATOM 817 CA LEU A 184 68.640 46.176 263.560 1.00 0.86
C
ATOM 818 C LEU A 184 69.730 45.249 264.109 1.00 0.86
C
ATOM 819 O LEU A 184 70.812 45.693 264.479 1.00 0.86
O
ATOM 820 CB LEU A 184 67.529 46.339 264.601 1.00 0.86
C
ATOM 821 CG LEU A 184 66.539 47.479 264.347 1.00 0.86
C
ATOM 822 CD1 LEU A 184 65.318 47.342 265.262 1.00 0.86
C
ATOM 823 CD2 LEU A 184 67.211 48.832 264.584 1.00 0.86
C
ATOM 824 N ARG A 185 69.491 43.918 264.151 1.00 0.76
N
ATOM 825 CA ARG A 185 70.474 42.957 264.633 1.00 0.76
C
ATOM 826 C ARG A 185 71.703 42.759 263.763 1.00 0.76
C
ATOM 827 O ARG A 185 72.810 42.606 264.270 1.00 0.76
O
ATOM 828 CB ARG A 185 69.848 41.560 264.851 1.00 0.76
C
ATOM 829 CG ARG A 185 68.874 41.518 266.039 1.00 0.76
C
ATOM 830 CD ARG A 185 68.294 40.134 266.337 1.00 0.76
C

```
ATOM    831  NE  ARG A 185      67.337  39.783 265.248  1.00  0.76
N
ATOM    832  CZ  ARG A 185      67.512  38.838 264.317  1.00  0.76
C
ATOM    833  NH1 ARG A 185      66.472  38.520 263.559  1.00  0.76
N
ATOM    834  NH2 ARG A 185      68.674  38.226 264.119  1.00  0.76
N
ATOM    835  N   ASN A 186      71.537  42.704 262.429  1.00  0.84
N
ATOM    836  CA  ASN A 186      72.631  42.360 261.535  1.00  0.84
C
ATOM    837  C   ASN A 186      73.200  43.590 260.858  1.00  0.84
C
ATOM    838  O   ASN A 186      74.073  43.489 259.991  1.00  0.84
O
ATOM    839  CB  ASN A 186      72.138  41.426 260.408  1.00  0.84
C
ATOM    840  CG  ASN A 186      71.637  40.109 260.974  1.00  0.84
C
ATOM    841  OD1 ASN A 186      72.410  39.196 261.256  1.00  0.84
O
ATOM    842  ND2 ASN A 186      70.296  39.977 261.084  1.00  0.84
N
ATOM    843  N   GLY A 187      72.687  44.774 261.239  1.00  0.87
N
ATOM    844  CA  GLY A 187      72.949  46.041 260.588  1.00  0.87
C
ATOM    845  C   GLY A 187      72.284  46.194 259.241  1.00  0.87
C
ATOM    846  O   GLY A 187      71.790  45.257 258.608  1.00  0.87
O
ATOM    847  N   VAL A 188      72.271  47.439 258.741  1.00  0.86
N
ATOM    848  CA  VAL A 188      71.715  47.750 257.441  1.00  0.86
C
ATOM    849  C   VAL A 188      72.740  47.436 256.367  1.00  0.86
C
ATOM    850  O   VAL A 188      73.779  48.084 256.261  1.00  0.86
O
ATOM    851  CB  VAL A 188      71.286  49.206 257.323  1.00  0.86
C
ATOM    852  CG1 VAL A 188      70.669  49.489 255.937  1.00  0.86
C
ATOM    853  CG2 VAL A 188      70.265  49.543 258.421  1.00  0.86
C
ATOM    854  N   LYS A 189      72.454  46.412 255.541  1.00  0.79
N
ATOM    855  CA  LYS A 189      73.369  45.949 254.523  1.00  0.79
C
ATOM    856  C   LYS A 189      72.623  45.224 253.404  1.00  0.79
C
ATOM    857  O   LYS A 189      71.528  44.702 253.657  1.00  0.79
O
```

ATOM 858 CB LYS A 189 74.442 45.005 255.124 1.00 0.79
C
ATOM 859 CG LYS A 189 73.895 43.717 255.764 1.00 0.79
C
ATOM 860 CD LYS A 189 75.014 42.863 256.378 1.00 0.79
C
ATOM 861 CE LYS A 189 74.489 41.576 257.014 1.00 0.79
C
ATOM 862 NZ LYS A 189 75.603 40.844 257.648 1.00 0.79
N
ATOM 863 N PRO A 190 73.136 45.156 252.176 1.00 0.72
N
ATOM 864 CA PRO A 190 72.422 44.556 251.056 1.00 0.72
C
ATOM 865 C PRO A 190 72.954 43.150 250.776 1.00 0.72
C
ATOM 866 O PRO A 190 73.902 42.741 251.447 1.00 0.72
O
ATOM 867 CB PRO A 190 72.748 45.521 249.910 1.00 0.72
C
ATOM 868 CG PRO A 190 74.165 45.996 250.201 1.00 0.72
C
ATOM 869 CD PRO A 190 74.305 45.923 251.720 1.00 0.72
C
ATOM 870 N PRO A 191 72.350 42.372 249.875 1.00 0.58
N
ATOM 871 CA PRO A 191 72.859 41.061 249.463 1.00 0.58
C
ATOM 872 C PRO A 191 73.872 41.132 248.321 1.00 0.58
C
ATOM 873 O PRO A 191 74.096 42.214 247.788 1.00 0.58
O
ATOM 874 CB PRO A 191 71.592 40.339 248.976 1.00 0.58
C
ATOM 875 CG PRO A 191 70.700 41.461 248.450 1.00 0.58
C
ATOM 876 CD PRO A 191 70.970 42.587 249.439 1.00 0.58
C
ATOM 877 N GLU A 192 74.479 39.973 247.933 1.00 0.44
N
ATOM 878 CA GLU A 192 75.576 39.895 246.966 1.00 0.44
C
ATOM 879 C GLU A 192 75.299 39.084 245.679 1.00 0.44
C
ATOM 880 O GLU A 192 74.190 39.118 245.156 1.00 0.44
O
ATOM 881 CB GLU A 192 76.906 39.484 247.650 1.00 0.44
C
ATOM 882 CG GLU A 192 76.952 38.163 248.449 1.00 0.44
C
ATOM 883 CD GLU A 192 78.356 37.878 249.001 1.00 0.44
C
ATOM 884 OE1 GLU A 192 78.442 36.970 249.865 1.00 0.44
O

```
ATOM     885  OE2 GLU A 192      79.334  38.533 248.558  1.00  0.44
O
ATOM     886  N   VAL A 193      76.313  38.381 245.093  1.00  0.40
N
ATOM     887  CA  VAL A 193      76.289  37.861 243.717  1.00  0.40
C
ATOM     888  C   VAL A 193      77.712  37.510 243.236  1.00  0.40
C
ATOM     889  O   VAL A 193      78.648  37.492 244.023  1.00  0.40
O
ATOM     890  CB  VAL A 193      75.285  36.716 243.526  1.00  0.40
C
ATOM     891  CG1 VAL A 193      75.036  36.388 242.040  1.00  0.40
C
ATOM     892  CG2 VAL A 193      75.701  35.465 244.315  1.00  0.40
C
ATOM     893  N   ILE A 194      77.951  37.230 241.921  1.00  0.50
N
ATOM     894  CA  ILE A 194      79.290  37.152 241.327  1.00  0.50
C
ATOM     895  C   ILE A 194      79.980  38.511 241.429  1.00  0.50
C
ATOM     896  O   ILE A 194      79.339  39.548 241.279  1.00  0.50
O
ATOM     897  CB  ILE A 194      79.282  36.657 239.863  1.00  0.50
C
ATOM     898  CG1 ILE A 194      78.740  35.211 239.766  1.00  0.50
C
ATOM     899  CG2 ILE A 194      80.683  36.735 239.204  1.00  0.50
C
ATOM     900  CD1 ILE A 194      78.522  34.738 238.321  1.00  0.50
C
ATOM     901  N   LYS A 195      81.307  38.549 241.693  1.00  0.61
N
ATOM     902  CA  LYS A 195      82.073  39.776 241.640  1.00  0.61
C
ATOM     903  C   LYS A 195      82.102  40.430 240.286  1.00  0.61
C
ATOM     904  O   LYS A 195      82.160  39.799 239.232  1.00  0.61
O
ATOM     905  CB  LYS A 195      83.545  39.607 242.047  1.00  0.61
C
ATOM     906  CG  LYS A 195      83.740  39.181 243.496  1.00  0.61
C
ATOM     907  CD  LYS A 195      85.238  39.134 243.788  1.00  0.61
C
ATOM     908  CE  LYS A 195      85.543  38.804 245.237  1.00  0.61
C
ATOM     909  NZ  LYS A 195      87.002  38.877 245.412  1.00  0.61
N
ATOM     910  N   LYS A 196      82.076  41.754 240.314  1.00  0.73
N
ATOM     911  CA  LYS A 196      81.925  42.523 239.132  1.00  0.73
C
```

```
ATOM     912  C   LYS A 196       82.674  43.817 239.321  1.00  0.73
C
ATOM     913  O   LYS A 196       82.697  44.367 240.421  1.00  0.73
O
ATOM     914  CB  LYS A 196       80.443  42.849 238.950  1.00  0.73
C
ATOM     915  CG  LYS A 196       79.466  41.720 238.588  1.00  0.73
C
ATOM     916  CD  LYS A 196       79.681  41.398 237.115  1.00  0.73
C
ATOM     917  CE  LYS A 196       78.772  40.392 236.435  1.00  0.73
C
ATOM     918  NZ  LYS A 196       79.234  40.304 235.036  1.00  0.73
N
ATOM     919  N   ARG A 197       83.310  44.335 238.257  1.00  0.79
N
ATOM     920  CA  ARG A 197       84.146  45.513 238.374  1.00  0.79
C
ATOM     921  C   ARG A 197       83.471  46.719 237.769  1.00  0.79
C
ATOM     922  O   ARG A 197       83.109  46.740 236.592  1.00  0.79
O
ATOM     923  CB  ARG A 197       85.508  45.311 237.682  1.00  0.79
C
ATOM     924  CG  ARG A 197       86.355  44.228 238.364  1.00  0.79
C
ATOM     925  CD  ARG A 197       87.672  44.004 237.633  1.00  0.79
C
ATOM     926  NE  ARG A 197       88.389  42.932 238.387  1.00  0.79
N
ATOM     927  CZ  ARG A 197       89.568  42.436 237.996  1.00  0.79
C
ATOM     928  NH1 ARG A 197       90.131  41.464 238.712  1.00  0.79
N
ATOM     929  NH2 ARG A 197       90.197  42.887 236.916  1.00  0.79
N
ATOM     930  N   VAL A 198       83.314  47.785 238.572  1.00  0.90
N
ATOM     931  CA  VAL A 198       82.793  49.044 238.085  1.00  0.90
C
ATOM     932  C   VAL A 198       83.900  50.058 238.133  1.00  0.90
C
ATOM     933  O   VAL A 198       84.563  50.253 239.150  1.00  0.90
O
ATOM     934  CB  VAL A 198       81.631  49.639 238.876  1.00  0.90
C
ATOM     935  CG1 VAL A 198       81.081  50.849 238.085  1.00  0.90
C
ATOM     936  CG2 VAL A 198       80.509  48.611 239.070  1.00  0.90
C
ATOM     937  N   LEU A 199       84.109  50.771 237.023  1.00  0.92
N
ATOM     938  CA  LEU A 199       84.970  51.919 237.014  1.00  0.92
C
```

```
ATOM    939  C   LEU A 199      84.126  53.162 236.966  1.00  0.92
C
ATOM    940  O   LEU A 199      83.205  53.281 236.159  1.00  0.92
O
ATOM    941  CB  LEU A 199      85.928  51.854 235.821  1.00  0.92
C
ATOM    942  CG  LEU A 199      87.057  52.897 235.831  1.00  0.92
C
ATOM    943  CD1 LEU A 199      88.310  52.226 235.271  1.00  0.92
C
ATOM    944  CD2 LEU A 199      86.715  54.169 235.040  1.00  0.92
C
ATOM    945  N   VAL A 200      84.427  54.120 237.851  1.00  0.93
N
ATOM    946  CA  VAL A 200      83.749  55.395 237.879  1.00  0.93
C
ATOM    947  C   VAL A 200      84.787  56.460 237.616  1.00  0.93
C
ATOM    948  O   VAL A 200      85.780  56.586 238.333  1.00  0.93
O
ATOM    949  CB  VAL A 200      83.029  55.665 239.190  1.00  0.93
C
ATOM    950  CG1 VAL A 200      82.138  56.918 239.058  1.00  0.93
C
ATOM    951  CG2 VAL A 200      82.161  54.443 239.559  1.00  0.93
C
ATOM    952  N   ASP A 201      84.573  57.232 236.541  1.00  0.90
N
ATOM    953  CA  ASP A 201      85.425  58.308 236.093  1.00  0.90
C
ATOM    954  C   ASP A 201      84.759  59.591 236.556  1.00  0.90
C
ATOM    955  O   ASP A 201      83.628  59.883 236.160  1.00  0.90
O
ATOM    956  CB  ASP A 201      85.531  58.231 234.548  1.00  0.90
C
ATOM    957  CG  ASP A 201      86.587  59.157 233.959  1.00  0.90
C
ATOM    958  OD1 ASP A 201      86.661  60.354 234.318  1.00  0.90
O
ATOM    959  OD2 ASP A 201      87.355  58.623 233.108  1.00  0.90
O
ATOM    960  N   PHE A 202      85.408  60.346 237.465  1.00  0.88
N
ATOM    961  CA  PHE A 202      84.761  61.492 238.063  1.00  0.88
C
ATOM    962  C   PHE A 202      85.721  62.498 238.671  1.00  0.88
C
ATOM    963  O   PHE A 202      86.907  62.240 238.860  1.00  0.88
O
ATOM    964  CB  PHE A 202      83.663  61.092 239.090  1.00  0.88
C
ATOM    965  CG  PHE A 202      84.193  60.504 240.373  1.00  0.88
C
```

ATOM 966 CD1 PHE A 202 84.696 59.196 240.430 1.00 0.88
C
ATOM 967 CD2 PHE A 202 84.142 61.258 241.555 1.00 0.88
C
ATOM 968 CE1 PHE A 202 85.138 58.653 241.641 1.00 0.88
C
ATOM 969 CE2 PHE A 202 84.577 60.717 242.769 1.00 0.88
C
ATOM 970 CZ PHE A 202 85.070 59.409 242.816 1.00 0.88
C
ATOM 971 N SER A 203 85.191 63.706 238.970 1.00 0.88
N
ATOM 972 CA SER A 203 85.929 64.913 239.321 1.00 0.88
C
ATOM 973 C SER A 203 86.607 65.486 238.101 1.00 0.88
C
ATOM 974 O SER A 203 86.110 66.418 237.476 1.00 0.88
O
ATOM 975 CB SER A 203 86.939 64.768 240.494 1.00 0.88
C
ATOM 976 OG SER A 203 87.518 66.026 240.861 1.00 0.88
O
ATOM 977 N SER A 204 87.768 64.912 237.745 1.00 0.89
N
ATOM 978 CA SER A 204 88.558 65.196 236.560 1.00 0.89
C
ATOM 979 C SER A 204 88.852 66.664 236.240 1.00 0.89
C
ATOM 980 O SER A 204 88.566 67.108 235.126 1.00 0.89
O
ATOM 981 CB SER A 204 87.999 64.445 235.324 1.00 0.89
C
ATOM 982 OG SER A 204 87.907 63.043 235.612 1.00 0.89
O
ATOM 983 N PRO A 205 89.414 67.491 237.133 1.00 0.88
N
ATOM 984 CA PRO A 205 89.859 68.828 236.792 1.00 0.88
C
ATOM 985 C PRO A 205 91.031 68.850 235.845 1.00 0.88
C
ATOM 986 O PRO A 205 91.749 67.867 235.669 1.00 0.88
O
ATOM 987 CB PRO A 205 90.220 69.475 238.133 1.00 0.88
C
ATOM 988 CG PRO A 205 90.519 68.305 239.062 1.00 0.88
C
ATOM 989 CD PRO A 205 89.556 67.242 238.565 1.00 0.88
C
ATOM 990 N ASN A 206 91.244 70.014 235.225 1.00 0.85
N
ATOM 991 CA ASN A 206 92.363 70.237 234.345 1.00 0.85
C
ATOM 992 C ASN A 206 93.496 70.875 235.126 1.00 0.85
C

```
ATOM    993  O   ASN A 206      93.305  71.859 235.834  1.00  0.85
O
ATOM    994  CB  ASN A 206      91.964  71.165 233.179  1.00  0.85
C
ATOM    995  CG  ASN A 206      90.871  70.488 232.365  1.00  0.85
C
ATOM    996  OD1 ASN A 206      91.012  69.360 231.908  1.00  0.85
O
ATOM    997  ND2 ASN A 206      89.737  71.200 232.159  1.00  0.85
N
ATOM    998  N   ILE A 207      94.718  70.322 235.027  1.00  0.85
N
ATOM    999  CA  ILE A 207      95.890  70.846 235.713  1.00  0.85
C
ATOM   1000  C   ILE A 207      96.233  72.259 235.257  1.00  0.85
C
ATOM   1001  O   ILE A 207      96.193  72.555 234.066  1.00  0.85
O
ATOM   1002  CB  ILE A 207      97.100  69.949 235.497  1.00  0.85
C
ATOM   1003  CG1 ILE A 207      96.827  68.457 235.810  1.00  0.85
C
ATOM   1004  CG2 ILE A 207      98.332  70.456 236.283  1.00  0.85
C
ATOM   1005  CD1 ILE A 207      96.123  68.168 237.133  1.00  0.85
C
ATOM   1006  N   ALA A 208      96.558  73.163 236.209  1.00  0.88
N
ATOM   1007  CA  ALA A 208      96.980  74.531 235.961  1.00  0.88
C
ATOM   1008  C   ALA A 208      95.858  75.482 235.584  1.00  0.88
C
ATOM   1009  O   ALA A 208      96.066  76.675 235.403  1.00  0.88
O
ATOM   1010  CB  ALA A 208      98.120  74.603 234.935  1.00  0.88
C
ATOM   1011  N   LYS A 209      94.622  74.971 235.526  1.00  0.78
N
ATOM   1012  CA  LYS A 209      93.428  75.758 235.398  1.00  0.78
C
ATOM   1013  C   LYS A 209      92.637  75.538 236.660  1.00  0.78
C
ATOM   1014  O   LYS A 209      92.700  74.490 237.294  1.00  0.78
O
ATOM   1015  CB  LYS A 209      92.575  75.325 234.179  1.00  0.78
C
ATOM   1016  CG  LYS A 209      93.049  75.962 232.868  1.00  0.78
C
ATOM   1017  CD  LYS A 209      92.289  75.416 231.649  1.00  0.78
C
ATOM   1018  CE  LYS A 209      92.441  76.272 230.389  1.00  0.78
C
ATOM   1019  NZ  LYS A 209      91.808  77.592 230.600  1.00  0.78
N
```

```
ATOM   1020  N   GLN A 210      91.885  76.561 237.086  1.00  0.75
N
ATOM   1021  CA  GLN A 210      91.078  76.493 238.278  1.00  0.75
C
ATOM   1022  C   GLN A 210      89.974  75.455 238.180  1.00  0.75
C
ATOM   1023  O   GLN A 210      89.092  75.530 237.322  1.00  0.75
O
ATOM   1024  CB  GLN A 210      90.488  77.892 238.583  1.00  0.75
C
ATOM   1025  CG  GLN A 210      89.669  78.018 239.890  1.00  0.75
C
ATOM   1026  CD  GLN A 210      90.512  77.726 241.127  1.00  0.75
C
ATOM   1027  OE1 GLN A 210      91.475  78.421 241.436  1.00  0.75
O
ATOM   1028  NE2 GLN A 210      90.146  76.658 241.874  1.00  0.75
N
ATOM   1029  N   MET A 211      90.003  74.456 239.090  1.00  0.79
N
ATOM   1030  CA  MET A 211      88.926  73.504 239.276  1.00  0.79
C
ATOM   1031  C   MET A 211      87.636  74.241 239.615  1.00  0.79
C
ATOM   1032  O   MET A 211      87.632  75.139 240.460  1.00  0.79
O
ATOM   1033  CB  MET A 211      89.307  72.489 240.384  1.00  0.79
C
ATOM   1034  CG  MET A 211      88.264  71.396 240.670  1.00  0.79
C
ATOM   1035  SD  MET A 211      88.792  70.237 241.971  1.00  0.79
S
ATOM   1036  CE  MET A 211      87.255  69.276 242.054  1.00  0.79
C
ATOM   1037  N   HIS A 212      86.534  73.909 238.917  1.00  0.80
N
ATOM   1038  CA  HIS A 212      85.330  74.714 238.916  1.00  0.80
C
ATOM   1039  C   HIS A 212      84.132  73.938 239.396  1.00  0.80
C
ATOM   1040  O   HIS A 212      84.201  72.770 239.764  1.00  0.80
O
ATOM   1041  CB  HIS A 212      85.055  75.333 237.519  1.00  0.80
C
ATOM   1042  CG  HIS A 212      84.955  74.374 236.373  1.00  0.80
C
ATOM   1043  ND1 HIS A 212      84.009  73.370 236.385  1.00  0.80
N
ATOM   1044  CD2 HIS A 212      85.659  74.341 235.214  1.00  0.80
C
ATOM   1045  CE1 HIS A 212      84.167  72.735 235.242  1.00  0.80
C
ATOM   1046  NE2 HIS A 212      85.147  73.287 234.490  1.00  0.80
N
```

```
ATOM   1047  N   VAL A 213      82.955  74.591 239.445  1.00  0.85
N
ATOM   1048  CA  VAL A 213      81.758  73.994 240.006  1.00  0.85
C
ATOM   1049  C   VAL A 213      81.175  72.863 239.173  1.00  0.85
C
ATOM   1050  O   VAL A 213      80.403  72.052 239.672  1.00  0.85
O
ATOM   1051  CB  VAL A 213      80.673  75.017 240.297  1.00  0.85
C
ATOM   1052  CG1 VAL A 213      81.065  75.840 241.537  1.00  0.85
C
ATOM   1053  CG2 VAL A 213      80.429  75.918 239.074  1.00  0.85
C
ATOM   1054  N   GLY A 214      81.572  72.726 237.893  1.00  0.86
N
ATOM   1055  CA  GLY A 214      81.226  71.564 237.091  1.00  0.86
C
ATOM   1056  C   GLY A 214      82.048  70.368 237.488  1.00  0.86
C
ATOM   1057  O   GLY A 214      81.549  69.249 237.558  1.00  0.86
O
ATOM   1058  N   HIS A 215      83.334  70.577 237.837  1.00  0.85
N
ATOM   1059  CA  HIS A 215      84.123  69.559 238.509  1.00  0.85
C
ATOM   1060  C   HIS A 215      83.576  69.205 239.884  1.00  0.85
C
ATOM   1061  O   HIS A 215      83.508  68.037 240.240  1.00  0.85
O
ATOM   1062  CB  HIS A 215      85.607  69.946 238.630  1.00  0.85
C
ATOM   1063  CG  HIS A 215      86.239  70.210 237.302  1.00  0.85
C
ATOM   1064  ND1 HIS A 215      86.830  71.434 237.043  1.00  0.85
N
ATOM   1065  CD2 HIS A 215      86.338  69.399 236.219  1.00  0.85
C
ATOM   1066  CE1 HIS A 215      87.267  71.340 235.802  1.00  0.85
C
ATOM   1067  NE2 HIS A 215      86.996  70.129 235.255  1.00  0.85
N
ATOM   1068  N   LEU A 216      83.109  70.197 240.677  1.00  0.88
N
ATOM   1069  CA  LEU A 216      82.465  69.954 241.961  1.00  0.88
C
ATOM   1070  C   LEU A 216      81.233  69.063 241.863  1.00  0.88
C
ATOM   1071  O   LEU A 216      81.116  68.106 242.628  1.00  0.88
O
ATOM   1072  CB  LEU A 216      82.073  71.286 242.648  1.00  0.88
C
ATOM   1073  CG  LEU A 216      81.412  71.162 244.039  1.00  0.88
C
```

```
ATOM   1074  CD1 LEU A 216       82.361  70.562 245.087  1.00  0.88
C
ATOM   1075  CD2 LEU A 216       80.879  72.520 244.520  1.00  0.88
C
ATOM   1076  N   ARG A 217       80.330  69.296 240.875  1.00  0.84
N
ATOM   1077  CA  ARG A 217       79.198  68.408 240.631  1.00  0.84
C
ATOM   1078  C   ARG A 217       79.646  66.986 240.337  1.00  0.84
C
ATOM   1079  O   ARG A 217       79.151  66.023 240.916  1.00  0.84
O
ATOM   1080  CB  ARG A 217       78.331  68.830 239.413  1.00  0.84
C
ATOM   1081  CG  ARG A 217       77.635  70.200 239.511  1.00  0.84
C
ATOM   1082  CD  ARG A 217       76.324  70.282 238.707  1.00  0.84
C
ATOM   1083  NE  ARG A 217       75.327  69.407 239.381  1.00  0.84
N
ATOM   1084  CZ  ARG A 217       74.063  69.231 238.973  1.00  0.84
C
ATOM   1085  NH1 ARG A 217       73.616  69.707 237.813  1.00  0.84
N
ATOM   1086  NH2 ARG A 217       73.236  68.569 239.775  1.00  0.84
N
ATOM   1087  N   SER A 218       80.665  66.836 239.467  1.00  0.90
N
ATOM   1088  CA  SER A 218       81.246  65.549 239.116  1.00  0.90
C
ATOM   1089  C   SER A 218       81.832  64.825 240.317  1.00  0.90
C
ATOM   1090  O   SER A 218       81.592  63.638 240.527  1.00  0.90
O
ATOM   1091  CB  SER A 218       82.322  65.711 238.012  1.00  0.90
C
ATOM   1092  OG  SER A 218       82.831  64.447 237.586  1.00  0.90
O
ATOM   1093  N   THR A 219       82.563  65.551 241.182  1.00  0.91
N
ATOM   1094  CA  THR A 219       83.121  65.037 242.428  1.00  0.91
C
ATOM   1095  C   THR A 219       82.070  64.526 243.394  1.00  0.91
C
ATOM   1096  O   THR A 219       82.159  63.410 243.903  1.00  0.91
O
ATOM   1097  CB  THR A 219       83.937  66.093 243.164  1.00  0.91
C
ATOM   1098  OG1 THR A 219       84.928  66.616 242.306  1.00  0.91
O
ATOM   1099  CG2 THR A 219       84.709  65.485 244.334  1.00  0.91
C
ATOM   1100  N   ILE A 220       81.012  65.323 243.649  1.00  0.90
N
```

ATOM 1101 CA ILE A 220 79.941 64.965 244.568 1.00 0.90
C
ATOM 1102 C ILE A 220 79.087 63.817 244.051 1.00 0.90
C
ATOM 1103 O ILE A 220 78.829 62.840 244.758 1.00 0.90
O
ATOM 1104 CB ILE A 220 79.064 66.180 244.863 1.00 0.90
C
ATOM 1105 CG1 ILE A 220 79.880 67.290 245.571 1.00 0.90
C
ATOM 1106 CG2 ILE A 220 77.843 65.776 245.719 1.00 0.90
C
ATOM 1107 CD1 ILE A 220 79.161 68.644 245.576 1.00 0.90
C
ATOM 1108 N ILE A 221 78.654 63.888 242.775 1.00 0.90
N
ATOM 1109 CA ILE A 221 77.852 62.853 242.142 1.00 0.90
C
ATOM 1110 C ILE A 221 78.618 61.558 242.020 1.00 0.90
C
ATOM 1111 O ILE A 221 78.120 60.497 242.387 1.00 0.90
O
ATOM 1112 CB ILE A 221 77.334 63.315 240.781 1.00 0.90
C
ATOM 1113 CG1 ILE A 221 76.285 64.435 240.972 1.00 0.90
C
ATOM 1114 CG2 ILE A 221 76.724 62.137 239.984 1.00 0.90
C
ATOM 1115 CD1 ILE A 221 75.980 65.208 239.685 1.00 0.90
C
ATOM 1116 N GLY A 222 79.881 61.611 241.556 1.00 0.93
N
ATOM 1117 CA GLY A 222 80.680 60.418 241.334 1.00 0.93
C
ATOM 1118 C GLY A 222 81.061 59.670 242.580 1.00 0.93
C
ATOM 1119 O GLY A 222 81.054 58.445 242.581 1.00 0.93
O
ATOM 1120 N GLU A 223 81.336 60.372 243.698 1.00 0.88
N
ATOM 1121 CA GLU A 223 81.582 59.741 244.986 1.00 0.88
C
ATOM 1122 C GLU A 223 80.372 58.977 245.476 1.00 0.88
C
ATOM 1123 O GLU A 223 80.446 57.825 245.887 1.00 0.88
O
ATOM 1124 CB GLU A 223 81.937 60.818 246.036 1.00 0.88
C
ATOM 1125 CG GLU A 223 82.152 60.307 247.484 1.00 0.88
C
ATOM 1126 CD GLU A 223 83.305 59.328 247.589 1.00 0.88
C
ATOM 1127 OE1 GLU A 223 84.427 59.619 247.115 1.00 0.88
O

ATOM 1128 OE2 GLU A 223 83.095 58.231 248.164 1.00 0.88
O
ATOM 1129 N SER A 224 79.190 59.604 245.374 1.00 0.92
N
ATOM 1130 CA SER A 224 77.936 59.003 245.782 1.00 0.92
C
ATOM 1131 C SER A 224 77.565 57.784 244.950 1.00 0.92
C
ATOM 1132 O SER A 224 77.126 56.757 245.465 1.00 0.92
O
ATOM 1133 CB SER A 224 76.822 60.071 245.800 1.00 0.92
C
ATOM 1134 OG SER A 224 75.567 59.542 246.228 1.00 0.92
O
ATOM 1135 N LEU A 225 77.816 57.836 243.625 1.00 0.92
N
ATOM 1136 CA LEU A 225 77.726 56.679 242.752 1.00 0.92
C
ATOM 1137 C LEU A 225 78.684 55.552 243.118 1.00 0.92
C
ATOM 1138 O LEU A 225 78.293 54.386 243.148 1.00 0.92
O
ATOM 1139 CB LEU A 225 78.005 57.073 241.287 1.00 0.92
C
ATOM 1140 CG LEU A 225 76.944 57.987 240.652 1.00 0.92
C
ATOM 1141 CD1 LEU A 225 77.429 58.460 239.281 1.00 0.92
C
ATOM 1142 CD2 LEU A 225 75.543 57.362 240.569 1.00 0.92
C
ATOM 1143 N CYS A 226 79.959 55.865 243.440 1.00 0.93
N
ATOM 1144 CA CYS A 226 80.910 54.870 243.910 1.00 0.93
C
ATOM 1145 C CYS A 226 80.464 54.207 245.188 1.00 0.93
C
ATOM 1146 O CYS A 226 80.391 52.985 245.254 1.00 0.93
O
ATOM 1147 CB CYS A 226 82.300 55.482 244.177 1.00 0.93
C
ATOM 1148 SG CYS A 226 83.077 56.057 242.648 1.00 0.93
S
ATOM 1149 N ARG A 227 80.051 55.007 246.191 1.00 0.83
N
ATOM 1150 CA ARG A 227 79.570 54.511 247.464 1.00 0.83
C
ATOM 1151 C ARG A 227 78.331 53.652 247.340 1.00 0.83
C
ATOM 1152 O ARG A 227 78.201 52.634 248.014 1.00 0.83
O
ATOM 1153 CB ARG A 227 79.272 55.642 248.466 1.00 0.83
C
ATOM 1154 CG ARG A 227 80.520 56.424 248.894 1.00 0.83
C

```
ATOM   1155  CD  ARG A 227      80.232  57.378 250.051  1.00  0.83
C
ATOM   1156  NE  ARG A 227      81.460  58.191 250.255  1.00  0.83
N
ATOM   1157  CZ  ARG A 227      81.787  58.844 251.373  1.00  0.83
C
ATOM   1158  NH1 ARG A 227      81.049  58.788 252.476  1.00  0.83
N
ATOM   1159  NH2 ARG A 227      82.872  59.605 251.396  1.00  0.83
N
ATOM   1160  N   LEU A 228      77.390  54.017 246.444  1.00  0.90
N
ATOM   1161  CA  LEU A 228      76.230  53.199 246.152  1.00  0.90
C
ATOM   1162  C   LEU A 228      76.610  51.816 245.643  1.00  0.90
C
ATOM   1163  O   LEU A 228      76.122  50.798 246.126  1.00  0.90
O
ATOM   1164  CB  LEU A 228      75.369  53.907 245.075  1.00  0.90
C
ATOM   1165  CG  LEU A 228      74.140  53.122 244.574  1.00  0.90
C
ATOM   1166  CD1 LEU A 228      73.132  52.866 245.698  1.00  0.90
C
ATOM   1167  CD2 LEU A 228      73.453  53.873 243.427  1.00  0.90
C
ATOM   1168  N   LEU A 229      77.538  51.748 244.673  1.00  0.87
N
ATOM   1169  CA  LEU A 229      78.029  50.509 244.114  1.00  0.87
C
ATOM   1170  C   LEU A 229      78.899  49.706 245.066  1.00  0.87
C
ATOM   1171  O   LEU A 229      78.769  48.486 245.122  1.00  0.87
O
ATOM   1172  CB  LEU A 229      78.737  50.763 242.771  1.00  0.87
C
ATOM   1173  CG  LEU A 229      77.822  51.375 241.688  1.00  0.87
C
ATOM   1174  CD1 LEU A 229      78.675  51.853 240.518  1.00  0.87
C
ATOM   1175  CD2 LEU A 229      76.741  50.402 241.186  1.00  0.87
C
ATOM   1176  N   GLU A 230      79.762  50.369 245.867  1.00  0.82
N
ATOM   1177  CA  GLU A 230      80.521  49.769 246.956  1.00  0.82
C
ATOM   1178  C   GLU A 230      79.612  49.134 247.998  1.00  0.82
C
ATOM   1179  O   GLU A 230      79.821  48.002 248.424  1.00  0.82
O
ATOM   1180  CB  GLU A 230      81.440  50.827 247.636  1.00  0.82
C
ATOM   1181  CG  GLU A 230      82.672  51.195 246.769  1.00  0.82
C
```

```
ATOM    1182  CD  GLU A 230      83.426  52.468 247.158  1.00  0.82
C
ATOM    1183  OE1 GLU A 230      82.883  53.368 247.848  1.00  0.82
O
ATOM    1184  OE2 GLU A 230      84.581  52.613 246.678  1.00  0.82
O
ATOM    1185  N   PHE A 231      78.517  49.821 248.386  1.00  0.82
N
ATOM    1186  CA  PHE A 231      77.495  49.259 249.243  1.00  0.82
C
ATOM    1187  C   PHE A 231      76.815  48.055 248.604  1.00  0.82
C
ATOM    1188  O   PHE A 231      76.685  47.015 249.228  1.00  0.82
O
ATOM    1189  CB  PHE A 231      76.474  50.367 249.614  1.00  0.82
C
ATOM    1190  CG  PHE A 231      75.566  49.997 250.752  1.00  0.82
C
ATOM    1191  CD1 PHE A 231      76.086  49.662 252.012  1.00  0.82
C
ATOM    1192  CD2 PHE A 231      74.174  50.050 250.584  1.00  0.82
C
ATOM    1193  CE1 PHE A 231      75.229  49.393 253.086  1.00  0.82
C
ATOM    1194  CE2 PHE A 231      73.314  49.793 251.659  1.00  0.82
C
ATOM    1195  CZ  PHE A 231      73.843  49.466 252.912  1.00  0.82
C
ATOM    1196  N   LEU A 232      76.456  48.129 247.305  1.00  0.81
N
ATOM    1197  CA  LEU A 232      75.833  47.035 246.576  1.00  0.81
C
ATOM    1198  C   LEU A 232      76.821  45.943 246.133  1.00  0.81
C
ATOM    1199  O   LEU A 232      76.509  45.167 245.233  1.00  0.81
O
ATOM    1200  CB  LEU A 232      75.136  47.570 245.292  1.00  0.81
C
ATOM    1201  CG  LEU A 232      73.928  48.514 245.474  1.00  0.81
C
ATOM    1202  CD1 LEU A 232      73.535  49.091 244.104  1.00  0.81
C
ATOM    1203  CD2 LEU A 232      72.715  47.812 246.099  1.00  0.81
C
ATOM    1204  N   GLN A 233      78.025  45.865 246.748  1.00  0.71
N
ATOM    1205  CA  GLN A 233      78.971  44.756 246.676  1.00  0.71
C
ATOM    1206  C   GLN A 233      79.894  44.770 245.464  1.00  0.71
C
ATOM    1207  O   GLN A 233      80.789  43.942 245.310  1.00  0.71
O
ATOM    1208  CB  GLN A 233      78.269  43.376 246.824  1.00  0.71
C
```

ATOM 1209 CG GLN A 233 79.203 42.161 247.011 1.00 0.71
C
ATOM 1210 CD GLN A 233 80.055 42.244 248.275 1.00 0.71
C
ATOM 1211 OE1 GLN A 233 79.616 42.640 249.354 1.00 0.71
O
ATOM 1212 NE2 GLN A 233 81.343 41.847 248.137 1.00 0.71
N
ATOM 1213 N HIS A 234 79.752 45.747 244.563 1.00 0.77
N
ATOM 1214 CA HIS A 234 80.638 45.859 243.420 1.00 0.77
C
ATOM 1215 C HIS A 234 82.085 46.174 243.798 1.00 0.77
C
ATOM 1216 O HIS A 234 82.340 46.956 244.711 1.00 0.77
O
ATOM 1217 CB HIS A 234 80.129 46.926 242.435 1.00 0.77
C
ATOM 1218 CG HIS A 234 78.762 46.610 241.900 1.00 0.77
C
ATOM 1219 ND1 HIS A 234 78.622 45.552 241.032 1.00 0.77
N
ATOM 1220 CD2 HIS A 234 77.556 47.205 242.103 1.00 0.77
C
ATOM 1221 CE1 HIS A 234 77.344 45.524 240.721 1.00 0.77
C
ATOM 1222 NE2 HIS A 234 76.649 46.506 241.338 1.00 0.77
N
ATOM 1223 N ASP A 235 83.077 45.606 243.074 1.00 0.84
N
ATOM 1224 CA ASP A 235 84.464 46.014 243.198 1.00 0.84
C
ATOM 1225 C ASP A 235 84.636 47.323 242.413 1.00 0.84
C
ATOM 1226 O ASP A 235 84.501 47.366 241.189 1.00 0.84
O
ATOM 1227 CB ASP A 235 85.449 44.935 242.652 1.00 0.84
C
ATOM 1228 CG ASP A 235 85.535 43.671 243.506 1.00 0.84
C
ATOM 1229 OD1 ASP A 235 85.853 43.779 244.716 1.00 0.84
O
ATOM 1230 OD2 ASP A 235 85.383 42.559 242.931 1.00 0.84
O
ATOM 1231 N VAL A 236 84.905 48.450 243.105 1.00 0.89
N
ATOM 1232 CA VAL A 236 84.830 49.773 242.497 1.00 0.89
C
ATOM 1233 C VAL A 236 86.194 50.423 242.345 1.00 0.89
C
ATOM 1234 O VAL A 236 86.975 50.575 243.282 1.00 0.89
O
ATOM 1235 CB VAL A 236 83.901 50.719 243.253 1.00 0.89
C

ATOM 1236 CG1 VAL A 236 83.888 52.136 242.639 1.00 0.89
C
ATOM 1237 CG2 VAL A 236 82.469 50.157 243.198 1.00 0.89
C
ATOM 1238 N ILE A 237 86.503 50.865 241.111 1.00 0.89
N
ATOM 1239 CA ILE A 237 87.720 51.583 240.790 1.00 0.89
C
ATOM 1240 C ILE A 237 87.363 53.041 240.544 1.00 0.89
C
ATOM 1241 O ILE A 237 86.677 53.406 239.589 1.00 0.89
O
ATOM 1242 CB ILE A 237 88.455 50.982 239.592 1.00 0.89
C
ATOM 1243 CG1 ILE A 237 88.833 49.504 239.863 1.00 0.89
C
ATOM 1244 CG2 ILE A 237 89.723 51.807 239.275 1.00 0.89
C
ATOM 1245 CD1 ILE A 237 89.376 48.769 238.631 1.00 0.89
C
ATOM 1246 N ARG A 238 87.833 53.926 241.440 1.00 0.84
N
ATOM 1247 CA ARG A 238 87.569 55.348 241.399 1.00 0.84
C
ATOM 1248 C ARG A 238 88.683 56.057 240.646 1.00 0.84
C
ATOM 1249 O ARG A 238 89.818 56.093 241.120 1.00 0.84
O
ATOM 1250 CB ARG A 238 87.532 55.903 242.846 1.00 0.84
C
ATOM 1251 CG ARG A 238 86.404 55.316 243.718 1.00 0.84
C
ATOM 1252 CD ARG A 238 86.811 55.048 245.174 1.00 0.84
C
ATOM 1253 NE ARG A 238 85.584 54.960 246.014 1.00 0.84
N
ATOM 1254 CZ ARG A 238 84.936 56.015 246.519 1.00 0.84
C
ATOM 1255 NH1 ARG A 238 85.334 57.258 246.274 1.00 0.84
N
ATOM 1256 NH2 ARG A 238 83.853 55.828 247.265 1.00 0.84
N
ATOM 1257 N ILE A 239 88.399 56.640 239.464 1.00 0.87
N
ATOM 1258 CA ILE A 239 89.421 57.233 238.609 1.00 0.87
C
ATOM 1259 C ILE A 239 89.224 58.729 238.462 1.00 0.87
C
ATOM 1260 O ILE A 239 88.148 59.216 238.125 1.00 0.87
O
ATOM 1261 CB ILE A 239 89.468 56.593 237.217 1.00 0.87
C
ATOM 1262 CG1 ILE A 239 89.861 55.099 237.280 1.00 0.87
C

```
ATOM   1263  CG2 ILE A 239      90.405  57.351 236.248  1.00  0.87
C
ATOM   1264  CD1 ILE A 239      91.252  54.808 237.860  1.00  0.87
C
ATOM   1265  N   ASN A 240      90.322  59.481 238.695  1.00  0.87
N
ATOM   1266  CA  ASN A 240      90.454  60.880 238.357  1.00  0.87
C
ATOM   1267  C   ASN A 240      91.082  60.978 236.978  1.00  0.87
C
ATOM   1268  O   ASN A 240      92.295  60.812 236.818  1.00  0.87
O
ATOM   1269  CB  ASN A 240      91.433  61.589 239.335  1.00  0.87
C
ATOM   1270  CG  ASN A 240      90.940  62.994 239.640  1.00  0.87
C
ATOM   1271  OD1 ASN A 240      90.092  63.573 238.969  1.00  0.87
O
ATOM   1272  ND2 ASN A 240      91.545  63.616 240.674  1.00  0.87
N
ATOM   1273  N   HIS A 241      90.306  61.262 235.931  1.00  0.86
N
ATOM   1274  CA  HIS A 241      90.854  61.347 234.591  1.00  0.86
C
ATOM   1275  C   HIS A 241      91.350  62.760 234.348  1.00  0.86
C
ATOM   1276  O   HIS A 241      90.820  63.502 233.528  1.00  0.86
O
ATOM   1277  CB  HIS A 241      89.792  60.916 233.562  1.00  0.86
C
ATOM   1278  CG  HIS A 241      90.284  60.640 232.173  1.00  0.86
C
ATOM   1279  ND1 HIS A 241      90.848  59.411 231.834  1.00  0.86
N
ATOM   1280  CD2 HIS A 241      90.208  61.443 231.082  1.00  0.86
C
ATOM   1281  CE1 HIS A 241      91.090  59.512 230.537  1.00  0.86
C
ATOM   1282  NE2 HIS A 241      90.728  60.716 230.036  1.00  0.86
N
ATOM   1283  N   LEU A 242      92.366  63.188 235.131  1.00  0.88
N
ATOM   1284  CA  LEU A 242      92.882  64.540 235.140  1.00  0.88
C
ATOM   1285  C   LEU A 242      93.407  64.999 233.791  1.00  0.88
C
ATOM   1286  O   LEU A 242      94.116  64.282 233.084  1.00  0.88
O
ATOM   1287  CB  LEU A 242      94.003  64.743 236.199  1.00  0.88
C
ATOM   1288  CG  LEU A 242      93.561  64.665 237.676  1.00  0.88
C
ATOM   1289  CD1 LEU A 242      94.751  64.855 238.629  1.00  0.88
C
```

```
ATOM   1290  CD2 LEU A 242      92.480  65.685 238.025  1.00  0.88
C
ATOM   1291  N   GLY A 243      93.086  66.257 233.430  1.00  0.87
N
ATOM   1292  CA  GLY A 243      93.573  66.879 232.208  1.00  0.87
C
ATOM   1293  C   GLY A 243      94.976  67.371 232.394  1.00  0.87
C
ATOM   1294  O   GLY A 243      95.214  68.555 232.633  1.00  0.87
O
ATOM   1295  N   ASP A 244      95.947  66.449 232.323  1.00  0.86
N
ATOM   1296  CA  ASP A 244      97.340  66.705 232.568  1.00  0.86
C
ATOM   1297  C   ASP A 244      98.202  66.628 231.315  1.00  0.86
C
ATOM   1298  O   ASP A 244      99.416  66.787 231.385  1.00  0.86
O
ATOM   1299  CB  ASP A 244      97.856  65.726 233.636  1.00  0.86
C
ATOM   1300  CG  ASP A 244      97.877  64.286 233.188  1.00  0.86
C
ATOM   1301  OD1 ASP A 244      97.350  63.916 232.112  1.00  0.86
O
ATOM   1302  OD2 ASP A 244      98.486  63.508 233.959  1.00  0.86
O
ATOM   1303  N   TRP A 245      97.597  66.407 230.134  1.00  0.80
N
ATOM   1304  CA  TRP A 245      98.365  66.204 228.921  1.00  0.80
C
ATOM   1305  C   TRP A 245      98.009  67.186 227.821  1.00  0.80
C
ATOM   1306  O   TRP A 245      98.405  67.046 226.667  1.00  0.80
O
ATOM   1307  CB  TRP A 245      98.197  64.732 228.478  1.00  0.80
C
ATOM   1308  CG  TRP A 245      99.320  64.160 227.628  1.00  0.80
C
ATOM   1309  CD1 TRP A 245      99.237  63.599 226.387  1.00  0.80
C
ATOM   1310  CD2 TRP A 245     100.704  64.087 228.016  1.00  0.80
C
ATOM   1311  NE1 TRP A 245     100.472  63.139 225.983  1.00  0.80
N
ATOM   1312  CE2 TRP A 245     101.389  63.431 226.965  1.00  0.80
C
ATOM   1313  CE3 TRP A 245     101.385  64.529 229.149  1.00  0.80
C
ATOM   1314  CZ2 TRP A 245     102.753  63.201 227.040  1.00  0.80
C
ATOM   1315  CZ3 TRP A 245     102.769  64.321 229.207  1.00  0.80
C
ATOM   1316  CH2 TRP A 245     103.443  63.661 228.170  1.00  0.80
C
```

```
ATOM   1317  N   GLY A 246      97.249  68.247 228.152  1.00  0.84
N
ATOM   1318  CA  GLY A 246      96.864  69.244 227.164  1.00  0.84
C
ATOM   1319  C   GLY A 246      97.976  70.124 226.632  1.00  0.84
C
ATOM   1320  O   GLY A 246      99.092  70.187 227.138  1.00  0.84
O
ATOM   1321  N   THR A 247      97.642  70.964 225.630  1.00  0.81
N
ATOM   1322  CA  THR A 247      98.567  71.924 225.027  1.00  0.81
C
ATOM   1323  C   THR A 247      98.764  73.134 225.906  1.00  0.81
C
ATOM   1324  O   THR A 247      99.667  73.943 225.704  1.00  0.81
O
ATOM   1325  CB  THR A 247      98.139  72.397 223.650  1.00  0.81
C
ATOM   1326  OG1 THR A 247      96.838  72.968 223.658  1.00  0.81
O
ATOM   1327  CG2 THR A 247      98.063  71.177 222.728  1.00  0.81
C
ATOM   1328  N   GLN A 248      97.934  73.199 226.964  1.00  0.78
N
ATOM   1329  CA  GLN A 248      97.957  74.100 228.090  1.00  0.78
C
ATOM   1330  C   GLN A 248      99.332  74.197 228.743  1.00  0.78
C
ATOM   1331  O   GLN A 248      99.757  75.264 229.171  1.00  0.78
O
ATOM   1332  CB  GLN A 248      96.869  73.615 229.104  1.00  0.78
C
ATOM   1333  CG  GLN A 248      97.185  72.281 229.838  1.00  0.78
C
ATOM   1334  CD  GLN A 248      95.942  71.514 230.309  1.00  0.78
C
ATOM   1335  OE1 GLN A 248      95.102  71.143 229.491  1.00  0.78
O
ATOM   1336  NE2 GLN A 248      95.845  71.209 231.620  1.00  0.78
N
ATOM   1337  N   PHE A 249     100.086  73.077 228.788  1.00  0.85
N
ATOM   1338  CA  PHE A 249     101.387  72.999 229.413  1.00  0.85
C
ATOM   1339  C   PHE A 249     102.498  73.711 228.689  1.00  0.85
C
ATOM   1340  O   PHE A 249     103.476  74.104 229.318  1.00  0.85
O
ATOM   1341  CB  PHE A 249     101.808  71.530 229.595  1.00  0.85
C
ATOM   1342  CG  PHE A 249     101.079  70.988 230.773  1.00  0.85
C
ATOM   1343  CD1 PHE A 249     101.563  71.235 232.069  1.00  0.85
C
```

```
ATOM   1344  CD2 PHE A 249      99.893  70.265 230.605  1.00  0.85
C
ATOM   1345  CE1 PHE A 249     100.894  70.715 233.181  1.00  0.85
C
ATOM   1346  CE2 PHE A 249      99.192  69.799 231.717  1.00  0.85
C
ATOM   1347  CZ  PHE A 249      99.718  69.984 232.997  1.00  0.85
C
ATOM   1348  N   GLY A 250     102.391  73.913 227.362  1.00  0.88
N
ATOM   1349  CA  GLY A 250     103.449  74.526 226.565  1.00  0.88
C
ATOM   1350  C   GLY A 250     103.958  75.846 227.068  1.00  0.88
C
ATOM   1351  O   GLY A 250     105.144  76.042 227.323  1.00  0.88
O
ATOM   1352  N   MET A 251     103.025  76.793 227.247  1.00  0.84
N
ATOM   1353  CA  MET A 251     103.301  78.104 227.786  1.00  0.84
C
ATOM   1354  C   MET A 251     103.747  78.080 229.242  1.00  0.84
C
ATOM   1355  O   MET A 251     104.622  78.838 229.655  1.00  0.84
O
ATOM   1356  CB  MET A 251     102.086  79.030 227.575  1.00  0.84
C
ATOM   1357  CG  MET A 251     100.813  78.630 228.336  1.00  0.84
C
ATOM   1358  SD  MET A 251      99.418  79.717 227.952  1.00  0.84
S
ATOM   1359  CE  MET A 251      98.491  79.208 229.417  1.00  0.84
C
ATOM   1360  N   LEU A 252     103.158  77.186 230.063  1.00  0.89
N
ATOM   1361  CA  LEU A 252     103.505  77.008 231.456  1.00  0.89
C
ATOM   1362  C   LEU A 252     104.922  76.512 231.678  1.00  0.89
C
ATOM   1363  O   LEU A 252     105.649  77.054 232.505  1.00  0.89
O
ATOM   1364  CB  LEU A 252     102.547  75.996 232.118  1.00  0.89
C
ATOM   1365  CG  LEU A 252     101.068  76.401 232.114  1.00  0.89
C
ATOM   1366  CD1 LEU A 252     100.207  75.203 232.518  1.00  0.89
C
ATOM   1367  CD2 LEU A 252     100.822  77.552 233.092  1.00  0.89
C
ATOM   1368  N   ILE A 253     105.359  75.488 230.916  1.00  0.87
N
ATOM   1369  CA  ILE A 253     106.714  74.953 230.950  1.00  0.87
C
ATOM   1370  C   ILE A 253     107.725  75.977 230.465  1.00  0.87
C
```

```
ATOM   1371  O   ILE A 253      108.727  76.230 231.125  1.00  0.87
O
ATOM   1372  CB  ILE A 253      106.804  73.646 230.159  1.00  0.87
C
ATOM   1373  CG1 ILE A 253      105.959  72.553 230.866  1.00  0.87
C
ATOM   1374  CG2 ILE A 253      108.274  73.192 229.996  1.00  0.87
C
ATOM   1375  CD1 ILE A 253      105.815  71.255 230.064  1.00  0.87
C
ATOM   1376  N   ALA A 254      107.439  76.670 229.342  1.00  0.91
N
ATOM   1377  CA  ALA A 254      108.297  77.713 228.819  1.00  0.91
C
ATOM   1378  C   ALA A 254      108.477  78.876 229.791  1.00  0.91
C
ATOM   1379  O   ALA A 254      109.575  79.366 230.021  1.00  0.91
O
ATOM   1380  CB  ALA A 254      107.718  78.190 227.471  1.00  0.91
C
ATOM   1381  N   HIS A 255      107.391  79.320 230.446  1.00  0.85
N
ATOM   1382  CA  HIS A 255      107.476  80.313 231.497  1.00  0.85
C
ATOM   1383  C   HIS A 255      108.189  79.831 232.758  1.00  0.85
C
ATOM   1384  O   HIS A 255      108.903  80.593 233.402  1.00  0.85
O
ATOM   1385  CB  HIS A 255      106.075  80.858 231.809  1.00  0.85
C
ATOM   1386  CG  HIS A 255      106.048  82.080 232.666  1.00  0.85
C
ATOM   1387  ND1 HIS A 255      106.641  83.259 232.238  1.00  0.85
N
ATOM   1388  CD2 HIS A 255      105.511  82.233 233.902  1.00  0.85
C
ATOM   1389  CE1 HIS A 255      106.454  84.100 233.245  1.00  0.85
C
ATOM   1390  NE2 HIS A 255      105.781  83.528 234.273  1.00  0.85
N
ATOM   1391  N   LEU A 256      108.038  78.544 233.145  1.00  0.88
N
ATOM   1392  CA  LEU A 256      108.753  77.963 234.274  1.00  0.88
C
ATOM   1393  C   LEU A 256      110.255  77.953 234.080  1.00  0.88
C
ATOM   1394  O   LEU A 256      111.003  78.326 234.981  1.00  0.88
O
ATOM   1395  CB  LEU A 256      108.271  76.518 234.559  1.00  0.88
C
ATOM   1396  CG  LEU A 256      108.781  75.923 235.888  1.00  0.88
C
ATOM   1397  CD1 LEU A 256      107.885  76.335 237.061  1.00  0.88
C
```

ATOM 1398 CD2 LEU A 256 108.865 74.394 235.814 1.00 0.88
C
ATOM 1399 N GLU A 257 110.718 77.578 232.873 1.00 0.82
N
ATOM 1400 CA GLU A 257 112.115 77.616 232.500 1.00 0.82
C
ATOM 1401 C GLU A 257 112.693 79.023 232.580 1.00 0.82
C
ATOM 1402 O GLU A 257 113.757 79.238 233.157 1.00 0.82
O
ATOM 1403 CB GLU A 257 112.254 77.162 231.035 1.00 0.82
C
ATOM 1404 CG GLU A 257 111.938 75.685 230.730 1.00 0.82
C
ATOM 1405 CD GLU A 257 111.867 75.493 229.230 1.00 0.82
C
ATOM 1406 OE1 GLU A 257 112.244 76.406 228.437 1.00 0.82
O
ATOM 1407 OE2 GLU A 257 111.389 74.421 228.797 1.00 0.82
O
ATOM 1408 N ASP A 258 111.966 80.020 232.026 1.00 0.84
N
ATOM 1409 CA ASP A 258 112.377 81.411 232.055 1.00 0.84
C
ATOM 1410 C ASP A 258 112.368 82.029 233.455 1.00 0.84
C
ATOM 1411 O ASP A 258 113.318 82.695 233.858 1.00 0.84
O
ATOM 1412 CB ASP A 258 111.496 82.300 231.126 1.00 0.84
C
ATOM 1413 CG ASP A 258 111.646 81.983 229.651 1.00 0.84
C
ATOM 1414 OD1 ASP A 258 112.174 80.919 229.274 1.00 0.84
O
ATOM 1415 OD2 ASP A 258 111.174 82.809 228.818 1.00 0.84
O
ATOM 1416 N ARG A 259 111.287 81.831 234.244 1.00 0.79
N
ATOM 1417 CA ARG A 259 111.171 82.399 235.580 1.00 0.79
C
ATOM 1418 C ARG A 259 112.092 81.741 236.592 1.00 0.79
C
ATOM 1419 O ARG A 259 112.665 82.400 237.459 1.00 0.79
O
ATOM 1420 CB ARG A 259 109.702 82.383 236.093 1.00 0.79
C
ATOM 1421 CG ARG A 259 109.491 83.084 237.457 1.00 0.79
C
ATOM 1422 CD ARG A 259 108.021 83.252 237.857 1.00 0.79
C
ATOM 1423 NE ARG A 259 108.010 83.876 239.221 1.00 0.79
N
ATOM 1424 CZ ARG A 259 106.873 84.127 239.883 1.00 0.79
C

ATOM 1425 NH1 ARG A 259 106.922 84.508 241.158 1.00 0.79
N
ATOM 1426 NH2 ARG A 259 105.697 83.941 239.320 1.00 0.79
N
ATOM 1427 N PHE A 260 112.254 80.409 236.512 1.00 0.82
N
ATOM 1428 CA PHE A 260 113.020 79.660 237.482 1.00 0.82
C
ATOM 1429 C PHE A 260 114.134 78.866 236.812 1.00 0.82
C
ATOM 1430 O PHE A 260 114.009 77.648 236.678 1.00 0.82
O
ATOM 1431 CB PHE A 260 112.122 78.657 238.240 1.00 0.82
C
ATOM 1432 CG PHE A 260 111.027 79.364 238.980 1.00 0.82
C
ATOM 1433 CD1 PHE A 260 111.308 80.134 240.118 1.00 0.82
C
ATOM 1434 CD2 PHE A 260 109.697 79.245 238.551 1.00 0.82
C
ATOM 1435 CE1 PHE A 260 110.274 80.770 240.815 1.00 0.82
C
ATOM 1436 CE2 PHE A 260 108.662 79.878 239.246 1.00 0.82
C
ATOM 1437 CZ PHE A 260 108.950 80.643 240.381 1.00 0.82
C
ATOM 1438 N PRO A 261 115.256 79.455 236.409 1.00 0.85
N
ATOM 1439 CA PRO A 261 116.214 78.798 235.520 1.00 0.85
C
ATOM 1440 C PRO A 261 116.963 77.637 236.153 1.00 0.85
C
ATOM 1441 O PRO A 261 117.641 76.907 235.436 1.00 0.85
O
ATOM 1442 CB PRO A 261 117.169 79.938 235.109 1.00 0.85
C
ATOM 1443 CG PRO A 261 116.992 81.007 236.190 1.00 0.85
C
ATOM 1444 CD PRO A 261 115.509 80.892 236.506 1.00 0.85
C
ATOM 1445 N ASN A 262 116.857 77.426 237.480 1.00 0.81
N
ATOM 1446 CA ASN A 262 117.490 76.314 238.158 1.00 0.81
C
ATOM 1447 C ASN A 262 116.467 75.265 238.600 1.00 0.81
C
ATOM 1448 O ASN A 262 116.761 74.431 239.452 1.00 0.81
O
ATOM 1449 CB ASN A 262 118.321 76.843 239.355 1.00 0.81
C
ATOM 1450 CG ASN A 262 119.362 75.836 239.852 1.00 0.81
C
ATOM 1451 OD1 ASN A 262 119.803 74.909 239.168 1.00 0.81
O

ATOM 1452 ND2 ASN A 262 119.785 76.037 241.117 1.00 0.81
N
ATOM 1453 N TYR A 263 115.250 75.240 237.997 1.00 0.80
N
ATOM 1454 CA TYR A 263 114.139 74.356 238.356 1.00 0.80
C
ATOM 1455 C TYR A 263 114.468 72.861 238.397 1.00 0.80
C
ATOM 1456 O TYR A 263 113.819 72.051 239.063 1.00 0.80
O
ATOM 1457 CB TYR A 263 112.904 74.597 237.428 1.00 0.80
C
ATOM 1458 CG TYR A 263 113.049 73.998 236.046 1.00 0.80
C
ATOM 1459 CD1 TYR A 263 113.670 74.691 234.997 1.00 0.80
C
ATOM 1460 CD2 TYR A 263 112.595 72.690 235.813 1.00 0.80
C
ATOM 1461 CE1 TYR A 263 113.833 74.085 233.741 1.00 0.80
C
ATOM 1462 CE2 TYR A 263 112.763 72.079 234.568 1.00 0.80
C
ATOM 1463 CZ TYR A 263 113.375 72.779 233.528 1.00 0.80
C
ATOM 1464 OH TYR A 263 113.523 72.144 232.280 1.00 0.80
O
ATOM 1465 N LEU A 264 115.490 72.452 237.623 1.00 0.80
N
ATOM 1466 CA LEU A 264 116.005 71.109 237.594 1.00 0.80
C
ATOM 1467 C LEU A 264 116.525 70.663 238.956 1.00 0.80
C
ATOM 1468 O LEU A 264 116.156 69.592 239.433 1.00 0.80
O
ATOM 1469 CB LEU A 264 117.097 71.021 236.499 1.00 0.80
C
ATOM 1470 CG LEU A 264 116.526 71.194 235.072 1.00 0.80
C
ATOM 1471 CD1 LEU A 264 117.621 71.354 234.009 1.00 0.80
C
ATOM 1472 CD2 LEU A 264 115.613 70.027 234.671 1.00 0.80
C
ATOM 1473 N ASN A 265 117.308 71.527 239.638 1.00 0.75
N
ATOM 1474 CA ASN A 265 117.889 71.238 240.934 1.00 0.75
C
ATOM 1475 C ASN A 265 117.084 71.843 242.075 1.00 0.75
C
ATOM 1476 O ASN A 265 116.554 71.127 242.922 1.00 0.75
O
ATOM 1477 CB ASN A 265 119.330 71.790 241.027 1.00 0.75
C
ATOM 1478 CG ASN A 265 120.184 71.111 239.966 1.00 0.75
C

```
ATOM   1479  OD1 ASN A 265     120.269  69.886 239.897  1.00  0.75
O
ATOM   1480  ND2 ASN A 265     120.842  71.919 239.104  1.00  0.75
N
ATOM   1481  N   GLU A 266     116.954  73.187 242.119  1.00  0.72
N
ATOM   1482  CA  GLU A 266     116.146  73.881 243.100  1.00  0.72
C
ATOM   1483  C   GLU A 266     114.690  73.759 242.720  1.00  0.72
C
ATOM   1484  O   GLU A 266     114.262  74.193 241.654  1.00  0.72
O
ATOM   1485  CB  GLU A 266     116.483  75.390 243.182  1.00  0.72
C
ATOM   1486  CG  GLU A 266     117.798  75.699 243.932  1.00  0.72
C
ATOM   1487  CD  GLU A 266     118.155  77.183 243.874  1.00  0.72
C
ATOM   1488  OE1 GLU A 266     118.242  77.811 244.955  1.00  0.72
O
ATOM   1489  OE2 GLU A 266     118.374  77.684 242.737  1.00  0.72
O
ATOM   1490  N   SER A 267     113.871  73.144 243.590  1.00  0.75
N
ATOM   1491  CA  SER A 267     112.445  73.019 243.337  1.00  0.75
C
ATOM   1492  C   SER A 267     111.761  74.386 243.302  1.00  0.75
C
ATOM   1493  O   SER A 267     111.874  75.121 244.283  1.00  0.75
O
ATOM   1494  CB  SER A 267     111.733  72.160 244.401  1.00  0.75
C
ATOM   1495  OG  SER A 267     110.418  71.800 243.972  1.00  0.75
O
ATOM   1496  N   PRO A 268     111.088  74.802 242.239  1.00  0.77
N
ATOM   1497  CA  PRO A 268     110.758  76.204 242.050  1.00  0.77
C
ATOM   1498  C   PRO A 268     109.641  76.657 242.982  1.00  0.77
C
ATOM   1499  O   PRO A 268     108.679  75.903 243.145  1.00  0.77
O
ATOM   1500  CB  PRO A 268     110.326  76.282 240.575  1.00  0.77
C
ATOM   1501  CG  PRO A 268     109.908  74.857 240.209  1.00  0.77
C
ATOM   1502  CD  PRO A 268     110.868  74.014 241.032  1.00  0.77
C
ATOM   1503  N   PRO A 269     109.676  77.845 243.579  1.00  0.78
N
ATOM   1504  CA  PRO A 269     108.570  78.348 244.377  1.00  0.78
C
ATOM   1505  C   PRO A 269     107.510  78.941 243.474  1.00  0.78
C
```

```
ATOM   1506  O   PRO A 269     107.322  80.157 243.419  1.00  0.78
O
ATOM   1507  CB  PRO A 269     109.234  79.408 245.272  1.00  0.78
C
ATOM   1508  CG  PRO A 269     110.385  79.942 244.418  1.00  0.78
C
ATOM   1509  CD  PRO A 269     110.867  78.685 243.696  1.00  0.78
C
ATOM   1510  N   ILE A 270     106.772  78.080 242.752  1.00  0.81
N
ATOM   1511  CA  ILE A 270     105.537  78.456 242.092  1.00  0.81
C
ATOM   1512  C   ILE A 270     104.520  78.788 243.175  1.00  0.81
C
ATOM   1513  O   ILE A 270     104.389  78.056 244.153  1.00  0.81
O
ATOM   1514  CB  ILE A 270     104.998  77.371 241.165  1.00  0.81
C
ATOM   1515  CG1 ILE A 270     106.098  76.746 240.274  1.00  0.81
C
ATOM   1516  CG2 ILE A 270     103.901  77.968 240.257  1.00  0.81
C
ATOM   1517  CD1 ILE A 270     106.100  75.212 240.307  1.00  0.81
C
ATOM   1518  N   SER A 271     103.817  79.930 243.068  1.00  0.77
N
ATOM   1519  CA  SER A 271     102.889  80.364 244.097  1.00  0.77
C
ATOM   1520  C   SER A 271     101.514  79.759 243.867  1.00  0.77
C
ATOM   1521  O   SER A 271     101.372  78.552 243.712  1.00  0.77
O
ATOM   1522  CB  SER A 271     102.830  81.915 244.173  1.00  0.77
C
ATOM   1523  OG  SER A 271     102.565  82.489 242.888  1.00  0.77
O
ATOM   1524  N   ASP A 272     100.441  80.576 243.835  1.00  0.78
N
ATOM   1525  CA  ASP A 272      99.164  80.168 243.291  1.00  0.78
C
ATOM   1526  C   ASP A 272      99.333  79.809 241.816  1.00  0.78
C
ATOM   1527  O   ASP A 272      99.948  80.542 241.036  1.00  0.78
O
ATOM   1528  CB  ASP A 272      98.145  81.317 243.528  1.00  0.78
C
ATOM   1529  CG  ASP A 272      96.841  81.108 242.777  1.00  0.78
C
ATOM   1530  OD1 ASP A 272      95.917  80.453 243.311  1.00  0.78
O
ATOM   1531  OD2 ASP A 272      96.786  81.606 241.627  1.00  0.78
O
ATOM   1532  N   LEU A 273      98.807  78.638 241.406  1.00  0.81
N
```

```
ATOM   1533  CA  LEU A 273      99.049  78.145 240.069  1.00  0.81
C
ATOM   1534  C   LEU A 273      98.324  78.958 239.007  1.00  0.81
C
ATOM   1535  O   LEU A 273      98.771  79.077 237.865  1.00  0.81
O
ATOM   1536  CB  LEU A 273      98.717  76.639 240.015  1.00  0.81
C
ATOM   1537  CG  LEU A 273      99.338  75.846 238.847  1.00  0.81
C
ATOM   1538  CD1 LEU A 273     100.842  76.120 238.698  1.00  0.81
C
ATOM   1539  CD2 LEU A 273      99.110  74.347 239.087  1.00  0.81
C
ATOM   1540  N   GLN A 274      97.203  79.593 239.392  1.00  0.76
N
ATOM   1541  CA  GLN A 274      96.412  80.455 238.542  1.00  0.76
C
ATOM   1542  C   GLN A 274      97.076  81.783 238.217  1.00  0.76
C
ATOM   1543  O   GLN A 274      97.029  82.249 237.078  1.00  0.76
O
ATOM   1544  CB  GLN A 274      95.003  80.684 239.129  1.00  0.76
C
ATOM   1545  CG  GLN A 274      94.350  79.405 239.702  1.00  0.76
C
ATOM   1546  CD  GLN A 274      94.329  78.272 238.681  1.00  0.76
C
ATOM   1547  OE1 GLN A 274      93.988  78.463 237.510  1.00  0.76
O
ATOM   1548  NE2 GLN A 274      94.672  77.041 239.135  1.00  0.76
N
ATOM   1549  N   LEU A 275      97.762  82.421 239.190  1.00  0.83
N
ATOM   1550  CA  LEU A 275      98.599  83.587 238.936  1.00  0.83
C
ATOM   1551  C   LEU A 275      99.707  83.283 237.942  1.00  0.83
C
ATOM   1552  O   LEU A 275      99.931  84.025 236.988  1.00  0.83
O
ATOM   1553  CB  LEU A 275      99.284  84.123 240.220  1.00  0.83
C
ATOM   1554  CG  LEU A 275      98.349  84.776 241.256  1.00  0.83
C
ATOM   1555  CD1 LEU A 275      99.151  85.123 242.520  1.00  0.83
C
ATOM   1556  CD2 LEU A 275      97.670  86.047 240.724  1.00  0.83
C
ATOM   1557  N   PHE A 276     100.380  82.131 238.119  1.00  0.84
N
ATOM   1558  CA  PHE A 276     101.425  81.646 237.240  1.00  0.84
C
ATOM   1559  C   PHE A 276     100.924  81.346 235.821  1.00  0.84
C
```

ATOM 1560 O PHE A 276 101.563 81.689 234.826 1.00 0.84
O
ATOM 1561 CB PHE A 276 102.067 80.408 237.915 1.00 0.84
C
ATOM 1562 CG PHE A 276 103.273 79.919 237.173 1.00 0.84
C
ATOM 1563 CD1 PHE A 276 104.536 80.484 237.409 1.00 0.84
C
ATOM 1564 CD2 PHE A 276 103.141 78.908 236.209 1.00 0.84
C
ATOM 1565 CE1 PHE A 276 105.648 80.053 236.678 1.00 0.84
C
ATOM 1566 CE2 PHE A 276 104.248 78.484 235.469 1.00 0.84
C
ATOM 1567 CZ PHE A 276 105.497 79.064 235.702 1.00 0.84
C
ATOM 1568 N TYR A 277 99.730 80.727 235.694 1.00 0.83
N
ATOM 1569 CA TYR A 277 99.038 80.535 234.431 1.00 0.83
C
ATOM 1570 C TYR A 277 98.716 81.866 233.758 1.00 0.83
C
ATOM 1571 O TYR A 277 98.899 82.043 232.558 1.00 0.83
O
ATOM 1572 CB TYR A 277 97.752 79.685 234.688 1.00 0.83
C
ATOM 1573 CG TYR A 277 96.935 79.371 233.453 1.00 0.83
C
ATOM 1574 CD1 TYR A 277 96.038 80.318 232.933 1.00 0.83
C
ATOM 1575 CD2 TYR A 277 97.024 78.119 232.824 1.00 0.83
C
ATOM 1576 CE1 TYR A 277 95.299 80.048 231.773 1.00 0.83
C
ATOM 1577 CE2 TYR A 277 96.286 77.844 231.664 1.00 0.83
C
ATOM 1578 CZ TYR A 277 95.441 78.818 231.123 1.00 0.83
C
ATOM 1579 OH TYR A 277 94.793 78.575 229.891 1.00 0.83
O
ATOM 1580 N LYS A 278 98.234 82.859 234.524 1.00 0.81
N
ATOM 1581 CA LYS A 278 97.935 84.181 234.016 1.00 0.81
C
ATOM 1582 C LYS A 278 99.168 84.963 233.557 1.00 0.81
C
ATOM 1583 O LYS A 278 99.129 85.618 232.513 1.00 0.81
O
ATOM 1584 CB LYS A 278 97.083 84.960 235.044 1.00 0.81
C
ATOM 1585 CG LYS A 278 96.381 86.203 234.474 1.00 0.81
C
ATOM 1586 CD LYS A 278 95.472 86.882 235.514 1.00 0.81
C

ATOM 1587 CE LYS A 278 94.829 88.189 235.031 1.00 0.81
C
ATOM 1588 NZ LYS A 278 93.844 87.932 233.955 1.00 0.81
N
ATOM 1589 N GLU A 279 100.301 84.870 234.299 1.00 0.81
N
ATOM 1590 CA GLU A 279 101.611 85.386 233.910 1.00 0.81
C
ATOM 1591 C GLU A 279 102.048 84.845 232.547 1.00 0.81
C
ATOM 1592 O GLU A 279 102.387 85.589 231.624 1.00 0.81
O
ATOM 1593 CB GLU A 279 102.708 84.924 234.930 1.00 0.81
C
ATOM 1594 CG GLU A 279 102.992 85.802 236.176 1.00 0.81
C
ATOM 1595 CD GLU A 279 103.956 85.155 237.169 1.00 0.81
C
ATOM 1596 OE1 GLU A 279 105.009 84.568 236.779 1.00 0.81
O
ATOM 1597 OE2 GLU A 279 103.663 85.254 238.391 1.00 0.81
O
ATOM 1598 N SER A 280 102.013 83.506 232.390 1.00 0.87
N
ATOM 1599 CA SER A 280 102.408 82.821 231.171 1.00 0.87
C
ATOM 1600 C SER A 280 101.468 83.032 230.006 1.00 0.87
C
ATOM 1601 O SER A 280 101.904 83.194 228.870 1.00 0.87
O
ATOM 1602 CB SER A 280 102.671 81.309 231.382 1.00 0.87
C
ATOM 1603 OG SER A 280 101.484 80.543 231.564 1.00 0.87
O
ATOM 1604 N LYS A 281 100.145 83.062 230.249 1.00 0.83
N
ATOM 1605 CA LYS A 281 99.165 83.330 229.218 1.00 0.83
C
ATOM 1606 C LYS A 281 99.298 84.707 228.595 1.00 0.83
C
ATOM 1607 O LYS A 281 99.175 84.857 227.384 1.00 0.83
O
ATOM 1608 CB LYS A 281 97.713 83.104 229.689 1.00 0.83
C
ATOM 1609 CG LYS A 281 96.750 83.134 228.493 1.00 0.83
C
ATOM 1610 CD LYS A 281 95.294 82.822 228.837 1.00 0.83
C
ATOM 1611 CE LYS A 281 94.432 82.609 227.583 1.00 0.83
C
ATOM 1612 NZ LYS A 281 94.486 83.761 226.667 1.00 0.83
N
ATOM 1613 N LYS A 282 99.615 85.750 229.393 1.00 0.83
N

ATOM 1614 CA LYS A 282 99.884 87.069 228.841 1.00 0.83
C
ATOM 1615 C LYS A 282 101.061 87.051 227.868 1.00 0.83
C
ATOM 1616 O LYS A 282 101.011 87.606 226.776 1.00 0.83
O
ATOM 1617 CB LYS A 282 100.127 88.111 229.965 1.00 0.83
C
ATOM 1618 CG LYS A 282 100.196 89.544 229.417 1.00 0.83
C
ATOM 1619 CD LYS A 282 100.581 90.631 230.435 1.00 0.83
C
ATOM 1620 CE LYS A 282 100.776 91.955 229.687 1.00 0.83
C
ATOM 1621 NZ LYS A 282 101.311 93.042 230.524 1.00 0.83
N
ATOM 1622 N ARG A 283 102.136 86.319 228.215 1.00 0.79
N
ATOM 1623 CA ARG A 283 103.250 86.078 227.319 1.00 0.79
C
ATOM 1624 C ARG A 283 102.866 85.323 226.055 1.00 0.79
C
ATOM 1625 O ARG A 283 103.299 85.660 224.958 1.00 0.79
O
ATOM 1626 CB ARG A 283 104.352 85.317 228.084 1.00 0.79
C
ATOM 1627 CG ARG A 283 104.962 86.165 229.215 1.00 0.79
C
ATOM 1628 CD ARG A 283 106.408 86.558 228.927 1.00 0.79
C
ATOM 1629 NE ARG A 283 107.195 85.364 229.330 1.00 0.79
N
ATOM 1630 CZ ARG A 283 108.388 85.003 228.850 1.00 0.79
C
ATOM 1631 NH1 ARG A 283 108.949 85.543 227.775 1.00 0.79
N
ATOM 1632 NH2 ARG A 283 109.016 84.036 229.505 1.00 0.79
N
ATOM 1633 N PHE A 284 102.009 84.295 226.177 1.00 0.84
N
ATOM 1634 CA PHE A 284 101.499 83.527 225.060 1.00 0.84
C
ATOM 1635 C PHE A 284 100.681 84.352 224.081 1.00 0.84
C
ATOM 1636 O PHE A 284 100.824 84.225 222.868 1.00 0.84
O
ATOM 1637 CB PHE A 284 100.659 82.354 225.620 1.00 0.84
C
ATOM 1638 CG PHE A 284 100.337 81.326 224.576 1.00 0.84
C
ATOM 1639 CD1 PHE A 284 101.299 80.381 224.192 1.00 0.84
C
ATOM 1640 CD2 PHE A 284 99.065 81.290 223.983 1.00 0.84
C

ATOM 1641 CE1 PHE A 284 100.990 79.402 223.240 1.00 0.84
C
ATOM 1642 CE2 PHE A 284 98.756 80.315 223.027 1.00 0.84
C
ATOM 1643 CZ PHE A 284 99.720 79.373 222.654 1.00 0.84
C
ATOM 1644 N ASP A 285 99.795 85.223 224.583 1.00 0.84
N
ATOM 1645 CA ASP A 285 98.962 86.051 223.748 1.00 0.84
C
ATOM 1646 C ASP A 285 99.728 87.195 223.066 1.00 0.84
C
ATOM 1647 O ASP A 285 99.436 87.531 221.921 1.00 0.84
O
ATOM 1648 CB ASP A 285 97.740 86.557 224.550 1.00 0.84
C
ATOM 1649 CG ASP A 285 96.818 85.429 224.989 1.00 0.84
C
ATOM 1650 OD1 ASP A 285 96.775 84.330 224.375 1.00 0.84
O
ATOM 1651 OD2 ASP A 285 96.068 85.656 225.975 1.00 0.84
O
ATOM 1652 N GLU A 286 100.733 87.800 223.744 1.00 0.80
N
ATOM 1653 CA GLU A 286 101.397 89.004 223.266 1.00 0.80
C
ATOM 1654 C GLU A 286 102.732 88.779 222.553 1.00 0.80
C
ATOM 1655 O GLU A 286 103.142 89.605 221.740 1.00 0.80
O
ATOM 1656 CB GLU A 286 101.660 89.967 224.456 1.00 0.80
C
ATOM 1657 CG GLU A 286 100.366 90.462 225.155 1.00 0.80
C
ATOM 1658 CD GLU A 286 100.611 91.430 226.310 1.00 0.80
C
ATOM 1659 OE1 GLU A 286 99.601 91.847 226.940 1.00 0.80
O
ATOM 1660 OE2 GLU A 286 101.786 91.765 226.617 1.00 0.80
O
ATOM 1661 N ASP A 287 103.441 87.654 222.792 1.00 0.83
N
ATOM 1662 CA ASP A 287 104.738 87.399 222.185 1.00 0.83
C
ATOM 1663 C ASP A 287 104.636 86.235 221.203 1.00 0.83
C
ATOM 1664 O ASP A 287 104.413 85.072 221.545 1.00 0.83
O
ATOM 1665 CB ASP A 287 105.806 87.193 223.301 1.00 0.83
C
ATOM 1666 CG ASP A 287 107.217 86.829 222.846 1.00 0.83
C
ATOM 1667 OD1 ASP A 287 107.432 86.535 221.646 1.00 0.83
O

ATOM 1668 OD2 ASP A 287 108.098 86.766 223.744 1.00 0.83
O
ATOM 1669 N GLU A 288 104.827 86.563 219.907 1.00 0.79
N
ATOM 1670 CA GLU A 288 104.801 85.629 218.805 1.00 0.79
C
ATOM 1671 C GLU A 288 105.868 84.550 218.902 1.00 0.79
C
ATOM 1672 O GLU A 288 105.602 83.374 218.657 1.00 0.79
O
ATOM 1673 CB GLU A 288 104.980 86.378 217.465 1.00 0.79
C
ATOM 1674 CG GLU A 288 103.772 87.254 217.054 1.00 0.79
C
ATOM 1675 CD GLU A 288 104.037 88.000 215.743 1.00 0.79
C
ATOM 1676 OE1 GLU A 288 104.675 87.398 214.841 1.00 0.79
O
ATOM 1677 OE2 GLU A 288 103.584 89.167 215.637 1.00 0.79
O
ATOM 1678 N GLU A 289 107.102 84.922 219.283 1.00 0.80
N
ATOM 1679 CA GLU A 289 108.219 84.012 219.440 1.00 0.80
C
ATOM 1680 C GLU A 289 108.080 83.107 220.659 1.00 0.80
C
ATOM 1681 O GLU A 289 108.378 81.909 220.615 1.00 0.80
O
ATOM 1682 CB GLU A 289 109.553 84.786 219.452 1.00 0.80
C
ATOM 1683 CG GLU A 289 110.022 85.241 218.042 1.00 0.80
C
ATOM 1684 CD GLU A 289 110.371 84.084 217.109 1.00 0.80
C
ATOM 1685 OE1 GLU A 289 110.969 83.075 217.548 1.00 0.80
O
ATOM 1686 OE2 GLU A 289 109.997 84.184 215.910 1.00 0.80
O
ATOM 1687 N PHE A 290 107.553 83.630 221.787 1.00 0.83
N
ATOM 1688 CA PHE A 290 107.221 82.828 222.958 1.00 0.83
C
ATOM 1689 C PHE A 290 106.198 81.735 222.650 1.00 0.83
C
ATOM 1690 O PHE A 290 106.331 80.592 223.091 1.00 0.83
O
ATOM 1691 CB PHE A 290 106.716 83.735 224.109 1.00 0.83
C
ATOM 1692 CG PHE A 290 106.444 82.980 225.378 1.00 0.83
C
ATOM 1693 CD1 PHE A 290 107.490 82.607 226.232 1.00 0.83
C
ATOM 1694 CD2 PHE A 290 105.132 82.610 225.705 1.00 0.83
C

ATOM 1695 CE1 PHE A 290 107.231 81.855 227.385 1.00 0.83
C
ATOM 1696 CE2 PHE A 290 104.865 81.864 226.857 1.00 0.83
C
ATOM 1697 CZ PHE A 290 105.918 81.478 227.693 1.00 0.83
C
ATOM 1698 N LYS A 291 105.176 82.049 221.832 1.00 0.83
N
ATOM 1699 CA LYS A 291 104.183 81.093 221.373 1.00 0.83
C
ATOM 1700 C LYS A 291 104.775 79.904 220.623 1.00 0.83
C
ATOM 1701 O LYS A 291 104.386 78.756 220.836 1.00 0.83
O
ATOM 1702 CB LYS A 291 103.143 81.844 220.510 1.00 0.83
C
ATOM 1703 CG LYS A 291 102.010 80.984 219.923 1.00 0.83
C
ATOM 1704 CD LYS A 291 100.869 81.816 219.301 1.00 0.83
C
ATOM 1705 CE LYS A 291 100.190 82.710 220.347 1.00 0.83
C
ATOM 1706 NZ LYS A 291 99.001 83.425 219.836 1.00 0.83
N
ATOM 1707 N LYS A 292 105.783 80.140 219.760 1.00 0.82
N
ATOM 1708 CA LYS A 292 106.508 79.083 219.075 1.00 0.82
C
ATOM 1709 C LYS A 292 107.285 78.178 220.012 1.00 0.82
C
ATOM 1710 O LYS A 292 107.278 76.955 219.873 1.00 0.82
O
ATOM 1711 CB LYS A 292 107.486 79.656 218.032 1.00 0.82
C
ATOM 1712 CG LYS A 292 106.808 80.584 217.019 1.00 0.82
C
ATOM 1713 CD LYS A 292 107.792 81.083 215.955 1.00 0.82
C
ATOM 1714 CE LYS A 292 107.317 82.371 215.282 1.00 0.82
C
ATOM 1715 NZ LYS A 292 108.367 82.907 214.401 1.00 0.82
N
ATOM 1716 N ARG A 293 107.949 78.758 221.037 1.00 0.79
N
ATOM 1717 CA ARG A 293 108.612 77.981 222.067 1.00 0.79
C
ATOM 1718 C ARG A 293 107.661 77.095 222.830 1.00 0.79
C
ATOM 1719 O ARG A 293 107.976 75.937 223.088 1.00 0.79
O
ATOM 1720 CB ARG A 293 109.281 78.855 223.146 1.00 0.79
C
ATOM 1721 CG ARG A 293 110.618 79.487 222.735 1.00 0.79
C

```
ATOM   1722  CD  ARG A 293     111.178  80.410 223.822  1.00  0.79
C
ATOM   1723  NE  ARG A 293     111.292  79.615 225.096  1.00  0.79
N
ATOM   1724  CZ  ARG A 293     111.193  80.148 226.318  1.00  0.79
C
ATOM   1725  NH1 ARG A 293     110.988  81.445 226.499  1.00  0.79
N
ATOM   1726  NH2 ARG A 293     111.347  79.375 227.386  1.00  0.79
N
ATOM   1727  N   ALA A 294     106.486  77.638 223.196  1.00  0.90
N
ATOM   1728  CA  ALA A 294     105.436  76.947 223.897  1.00  0.90
C
ATOM   1729  C   ALA A 294     104.902  75.738 223.140  1.00  0.90
C
ATOM   1730  O   ALA A 294     104.761  74.656 223.704  1.00  0.90
O
ATOM   1731  CB  ALA A 294     104.310  77.956 224.179  1.00  0.90
C
ATOM   1732  N   TYR A 295     104.661  75.864 221.821  1.00  0.81
N
ATOM   1733  CA  TYR A 295     104.315  74.735 220.973  1.00  0.81
C
ATOM   1734  C   TYR A 295     105.423  73.688 220.893  1.00  0.81
C
ATOM   1735  O   TYR A 295     105.174  72.494 221.039  1.00  0.81
O
ATOM   1736  CB  TYR A 295     103.894  75.201 219.556  1.00  0.81
C
ATOM   1737  CG  TYR A 295     102.601  75.990 219.547  1.00  0.81
C
ATOM   1738  CD1 TYR A 295     101.522  75.700 220.405  1.00  0.81
C
ATOM   1739  CD2 TYR A 295     102.439  77.017 218.601  1.00  0.81
C
ATOM   1740  CE1 TYR A 295     100.325  76.426 220.325  1.00  0.81
C
ATOM   1741  CE2 TYR A 295     101.240  77.738 218.513  1.00  0.81
C
ATOM   1742  CZ  TYR A 295     100.183  77.441 219.377  1.00  0.81
C
ATOM   1743  OH  TYR A 295      98.969  78.148 219.277  1.00  0.81
O
ATOM   1744  N   SER A 296     106.697  74.105 220.758  1.00  0.84
N
ATOM   1745  CA  SER A 296     107.840  73.200 220.852  1.00  0.84
C
ATOM   1746  C   SER A 296     107.973  72.496 222.194  1.00  0.84
C
ATOM   1747  O   SER A 296     108.419  71.353 222.255  1.00  0.84
O
ATOM   1748  CB  SER A 296     109.190  73.880 220.530  1.00  0.84
C
```

ATOM 1749 OG SER A 296 109.230 74.267 219.158 1.00 0.84
O
ATOM 1750 N ARG A 297 107.593 73.143 223.318 1.00 0.80
N
ATOM 1751 CA ARG A 297 107.487 72.479 224.608 1.00 0.80
C
ATOM 1752 C ARG A 297 106.438 71.383 224.657 1.00 0.80
C
ATOM 1753 O ARG A 297 106.679 70.330 225.234 1.00 0.80
O
ATOM 1754 CB ARG A 297 107.256 73.441 225.797 1.00 0.80
C
ATOM 1755 CG ARG A 297 108.376 74.467 226.045 1.00 0.80
C
ATOM 1756 CD ARG A 297 109.783 73.884 226.110 1.00 0.80
C
ATOM 1757 NE ARG A 297 110.688 75.025 226.372 1.00 0.80
N
ATOM 1758 CZ ARG A 297 111.394 75.709 225.479 1.00 0.80
C
ATOM 1759 NH1 ARG A 297 112.352 76.535 225.869 1.00 0.80
N
ATOM 1760 NH2 ARG A 297 111.117 75.635 224.185 1.00 0.80
N
ATOM 1761 N VAL A 298 105.258 71.573 224.031 1.00 0.85
N
ATOM 1762 CA VAL A 298 104.243 70.526 223.922 1.00 0.85
C
ATOM 1763 C VAL A 298 104.754 69.319 223.142 1.00 0.85
C
ATOM 1764 O VAL A 298 104.610 68.172 223.564 1.00 0.85
O
ATOM 1765 CB VAL A 298 102.961 71.050 223.274 1.00 0.85
C
ATOM 1766 CG1 VAL A 298 101.928 69.925 223.057 1.00 0.85
C
ATOM 1767 CG2 VAL A 298 102.350 72.155 224.155 1.00 0.85
C
ATOM 1768 N VAL A 299 105.434 69.550 221.999 1.00 0.82
N
ATOM 1769 CA VAL A 299 106.063 68.485 221.227 1.00 0.82
C
ATOM 1770 C VAL A 299 107.200 67.806 221.979 1.00 0.82
C
ATOM 1771 O VAL A 299 107.344 66.589 221.958 1.00 0.82
O
ATOM 1772 CB VAL A 299 106.559 68.947 219.859 1.00 0.82
C
ATOM 1773 CG1 VAL A 299 106.939 67.735 218.982 1.00 0.82
C
ATOM 1774 CG2 VAL A 299 105.445 69.724 219.134 1.00 0.82
C
ATOM 1775 N SER A 300 108.057 68.557 222.699 1.00 0.82
N

```
ATOM   1776  CA  SER A 300     109.086  67.959 223.535  1.00  0.82
C
ATOM   1777  C   SER A 300     108.541  67.159 224.704  1.00  0.82
C
ATOM   1778  O   SER A 300     109.086  66.111 225.041  1.00  0.82
O
ATOM   1779  CB  SER A 300     110.196  68.950 223.978  1.00  0.82
C
ATOM   1780  OG  SER A 300     109.771  69.918 224.934  1.00  0.82
O
ATOM   1781  N   LEU A 301     107.424  67.600 225.316  1.00  0.84
N
ATOM   1782  CA  LEU A 301     106.698  66.847 226.321  1.00  0.84
C
ATOM   1783  C   LEU A 301     106.138  65.523 225.805  1.00  0.84
C
ATOM   1784  O   LEU A 301     106.320  64.486 226.438  1.00  0.84
O
ATOM   1785  CB  LEU A 301     105.583  67.729 226.931  1.00  0.84
C
ATOM   1786  CG  LEU A 301     104.944  67.211 228.232  1.00  0.84
C
ATOM   1787  CD1 LEU A 301     105.963  67.039 229.367  1.00  0.84
C
ATOM   1788  CD2 LEU A 301     103.817  68.168 228.654  1.00  0.84
C
ATOM   1789  N   GLN A 302     105.520  65.497 224.597  1.00  0.77
N
ATOM   1790  CA  GLN A 302     105.069  64.258 223.961  1.00  0.77
C
ATOM   1791  C   GLN A 302     106.224  63.347 223.548  1.00  0.77
C
ATOM   1792  O   GLN A 302     106.096  62.127 223.506  1.00  0.77
O
ATOM   1793  CB  GLN A 302     104.104  64.520 222.764  1.00  0.77
C
ATOM   1794  CG  GLN A 302     103.508  63.231 222.125  1.00  0.77
C
ATOM   1795  CD  GLN A 302     102.375  63.505 221.123  1.00  0.77
C
ATOM   1796  OE1 GLN A 302     101.349  64.103 221.450  1.00  0.77
O
ATOM   1797  NE2 GLN A 302     102.548  63.016 219.872  1.00  0.77
N
ATOM   1798  N   LYS A 303     107.414  63.918 223.278  1.00  0.77
N
ATOM   1799  CA  LYS A 303     108.624  63.166 223.002  1.00  0.77
C
ATOM   1800  C   LYS A 303     109.281  62.567 224.239  1.00  0.77
C
ATOM   1801  O   LYS A 303     110.212  61.776 224.115  1.00  0.77
O
ATOM   1802  CB  LYS A 303     109.673  64.057 222.295  1.00  0.77
C
```

ATOM 1803 CG LYS A 303 109.349 64.362 220.826 1.00 0.77
C
ATOM 1804 CD LYS A 303 110.296 65.425 220.246 1.00 0.77
C
ATOM 1805 CE LYS A 303 110.100 65.649 218.745 1.00 0.77
C
ATOM 1806 NZ LYS A 303 110.948 66.770 218.280 1.00 0.77
N
ATOM 1807 N GLY A 304 108.845 62.934 225.464 1.00 0.83
N
ATOM 1808 CA GLY A 304 109.416 62.369 226.682 1.00 0.83
C
ATOM 1809 C GLY A 304 110.696 63.010 227.138 1.00 0.83
C
ATOM 1810 O GLY A 304 111.443 62.425 227.916 1.00 0.83
O
ATOM 1811 N VAL A 305 111.010 64.230 226.663 1.00 0.83
N
ATOM 1812 CA VAL A 305 112.269 64.906 226.960 1.00 0.83
C
ATOM 1813 C VAL A 305 112.500 65.132 228.467 1.00 0.83
C
ATOM 1814 O VAL A 305 111.661 65.776 229.096 1.00 0.83
O
ATOM 1815 CB VAL A 305 112.346 66.231 226.207 1.00 0.83
C
ATOM 1816 CG1 VAL A 305 113.530 67.127 226.629 1.00 0.83
C
ATOM 1817 CG2 VAL A 305 112.445 65.923 224.700 1.00 0.83
C
ATOM 1818 N PRO A 306 113.591 64.661 229.102 1.00 0.83
N
ATOM 1819 CA PRO A 306 113.741 64.642 230.559 1.00 0.83
C
ATOM 1820 C PRO A 306 113.542 65.968 231.260 1.00 0.83
C
ATOM 1821 O PRO A 306 112.802 66.038 232.238 1.00 0.83
O
ATOM 1822 CB PRO A 306 115.150 64.067 230.778 1.00 0.83
C
ATOM 1823 CG PRO A 306 115.322 63.094 229.609 1.00 0.83
C
ATOM 1824 CD PRO A 306 114.564 63.770 228.466 1.00 0.83
C
ATOM 1825 N ASN A 307 114.175 67.045 230.767 1.00 0.81
N
ATOM 1826 CA ASN A 307 114.060 68.373 231.339 1.00 0.81
C
ATOM 1827 C ASN A 307 112.649 68.952 231.237 1.00 0.81
C
ATOM 1828 O ASN A 307 112.151 69.569 232.180 1.00 0.81
O
ATOM 1829 CB ASN A 307 115.093 69.322 230.683 1.00 0.81
C

```
ATOM   1830  CG  ASN A 307     116.512  68.878 231.036  1.00  0.81
C
ATOM   1831  OD1 ASN A 307     116.755  68.037 231.898  1.00  0.81
O
ATOM   1832  ND2 ASN A 307     117.508  69.461 230.330  1.00  0.81
N
ATOM   1833  N   SER A 308     111.964  68.749 230.090  1.00  0.86
N
ATOM   1834  CA  SER A 308     110.575  69.135 229.877  1.00  0.86
C
ATOM   1835  C   SER A 308     109.614  68.364 230.760  1.00  0.86
C
ATOM   1836  O   SER A 308     108.697  68.926 231.355  1.00  0.86
O
ATOM   1837  CB  SER A 308     110.113  68.944 228.407  1.00  0.86
C
ATOM   1838  OG  SER A 308     110.985  69.608 227.494  1.00  0.86
O
ATOM   1839  N   ILE A 309     109.828  67.038 230.896  1.00  0.85
N
ATOM   1840  CA  ILE A 309     109.060  66.154 231.764  1.00  0.85
C
ATOM   1841  C   ILE A 309     109.199  66.543 233.225  1.00  0.85
C
ATOM   1842  O   ILE A 309     108.218  66.599 233.963  1.00  0.85
O
ATOM   1843  CB  ILE A 309     109.462  64.696 231.548  1.00  0.85
C
ATOM   1844  CG1 ILE A 309     109.021  64.191 230.150  1.00  0.85
C
ATOM   1845  CG2 ILE A 309     108.949  63.777 232.680  1.00  0.85
C
ATOM   1846  CD1 ILE A 309     107.528  63.869 229.999  1.00  0.85
C
ATOM   1847  N   LYS A 310     110.424  66.889 233.669  1.00  0.82
N
ATOM   1848  CA  LYS A 310     110.686  67.392 235.007  1.00  0.82
C
ATOM   1849  C   LYS A 310     109.921  68.673 235.314  1.00  0.82
C
ATOM   1850  O   LYS A 310     109.329  68.817 236.379  1.00  0.82
O
ATOM   1851  CB  LYS A 310     112.209  67.632 235.161  1.00  0.82
C
ATOM   1852  CG  LYS A 310     112.703  68.131 236.533  1.00  0.82
C
ATOM   1853  CD  LYS A 310     112.593  67.086 237.656  1.00  0.82
C
ATOM   1854  CE  LYS A 310     113.627  67.275 238.777  1.00  0.82
C
ATOM   1855  NZ  LYS A 310     113.474  68.583 239.453  1.00  0.82
N
ATOM   1856  N   ALA A 311     109.864  69.635 234.368  1.00  0.89
N
```

```
ATOM   1857  CA  ALA A 311     109.061  70.834 234.516  1.00  0.89
C
ATOM   1858  C   ALA A 311     107.566  70.552 234.629  1.00  0.89
C
ATOM   1859  O   ALA A 311     106.873  71.113 235.476  1.00  0.89
O
ATOM   1860  CB  ALA A 311     109.326  71.772 233.327  1.00  0.89
C
ATOM   1861  N   TRP A 312     107.056  69.622 233.800  1.00  0.84
N
ATOM   1862  CA  TRP A 312     105.690  69.142 233.845  1.00  0.84
C
ATOM   1863  C   TRP A 312     105.326  68.504 235.187  1.00  0.84
C
ATOM   1864  O   TRP A 312     104.303  68.846 235.779  1.00  0.84
O
ATOM   1865  CB  TRP A 312     105.491  68.154 232.667  1.00  0.84
C
ATOM   1866  CG  TRP A 312     104.154  67.439 232.607  1.00  0.84
C
ATOM   1867  CD1 TRP A 312     102.936  67.942 232.266  1.00  0.84
C
ATOM   1868  CD2 TRP A 312     103.941  66.069 232.974  1.00  0.84
C
ATOM   1869  NE1 TRP A 312     101.968  66.987 232.431  1.00  0.84
N
ATOM   1870  CE2 TRP A 312     102.548  65.837 232.881  1.00  0.84
C
ATOM   1871  CE3 TRP A 312     104.810  65.060 233.374  1.00  0.84
C
ATOM   1872  CZ2 TRP A 312     102.009  64.610 233.209  1.00  0.84
C
ATOM   1873  CZ3 TRP A 312     104.264  63.809 233.680  1.00  0.84
C
ATOM   1874  CH2 TRP A 312     102.881  63.590 233.610  1.00  0.84
C
ATOM   1875  N   GLU A 313     106.197  67.633 235.741  1.00  0.84
N
ATOM   1876  CA  GLU A 313     106.016  67.047 237.059  1.00  0.84
C
ATOM   1877  C   GLU A 313     105.930  68.091 238.158  1.00  0.84
C
ATOM   1878  O   GLU A 313     105.016  68.072 238.979  1.00  0.84
O
ATOM   1879  CB  GLU A 313     107.193  66.101 237.391  1.00  0.84
C
ATOM   1880  CG  GLU A 313     107.097  65.382 238.762  1.00  0.84
C
ATOM   1881  CD  GLU A 313     108.474  65.135 239.380  1.00  0.84
C
ATOM   1882  OE1 GLU A 313     108.639  65.501 240.574  1.00  0.84
O
ATOM   1883  OE2 GLU A 313     109.374  64.629 238.663  1.00  0.84
O
```

ATOM 1884 N LEU A 314 106.834 69.089 238.163 1.00 0.85
N
ATOM 1885 CA LEU A 314 106.833 70.150 239.153 1.00 0.85
C
ATOM 1886 C LEU A 314 105.553 70.983 239.144 1.00 0.85
C
ATOM 1887 O LEU A 314 104.979 71.279 240.191 1.00 0.85
O
ATOM 1888 CB LEU A 314 108.068 71.054 238.951 1.00 0.85
C
ATOM 1889 CG LEU A 314 109.415 70.346 239.214 1.00 0.85
C
ATOM 1890 CD1 LEU A 314 110.549 71.170 238.599 1.00 0.85
C
ATOM 1891 CD2 LEU A 314 109.668 70.088 240.706 1.00 0.85
C
ATOM 1892 N ILE A 315 105.036 71.332 237.949 1.00 0.86
N
ATOM 1893 CA ILE A 315 103.752 72.006 237.792 1.00 0.86
C
ATOM 1894 C ILE A 315 102.577 71.143 238.259 1.00 0.86
C
ATOM 1895 O ILE A 315 101.687 71.596 238.981 1.00 0.86
O
ATOM 1896 CB ILE A 315 103.570 72.474 236.348 1.00 0.86
C
ATOM 1897 CG1 ILE A 315 104.669 73.509 235.993 1.00 0.86
C
ATOM 1898 CG2 ILE A 315 102.166 73.088 236.131 1.00 0.86
C
ATOM 1899 CD1 ILE A 315 104.711 73.855 234.502 1.00 0.86
C
ATOM 1900 N CYS A 316 102.561 69.842 237.903 1.00 0.87
N
ATOM 1901 CA CYS A 316 101.561 68.891 238.372 1.00 0.87
C
ATOM 1902 C CYS A 316 101.590 68.653 239.878 1.00 0.87
C
ATOM 1903 O CYS A 316 100.546 68.506 240.512 1.00 0.87
O
ATOM 1904 CB CYS A 316 101.641 67.554 237.596 1.00 0.87
C
ATOM 1905 SG CYS A 316 101.118 67.786 235.859 1.00 0.87
S
ATOM 1906 N ASN A 317 102.785 68.638 240.503 1.00 0.85
N
ATOM 1907 CA ASN A 317 102.976 68.534 241.941 1.00 0.85
C
ATOM 1908 C ASN A 317 102.315 69.671 242.718 1.00 0.85
C
ATOM 1909 O ASN A 317 101.697 69.438 243.755 1.00 0.85
O
ATOM 1910 CB ASN A 317 104.488 68.489 242.309 1.00 0.85
C

```
ATOM   1911  CG  ASN A 317     105.115  67.143 241.927  1.00  0.85
C
ATOM   1912  OD1 ASN A 317     104.409  66.153 241.738  1.00  0.85
O
ATOM   1913  ND2 ASN A 317     106.469  67.111 241.860  1.00  0.85
N
ATOM   1914  N   VAL A 318     102.413  70.924 242.227  1.00  0.86
N
ATOM   1915  CA  VAL A 318     101.773  72.086 242.838  1.00  0.86
C
ATOM   1916  C   VAL A 318     100.253  71.987 242.811  1.00  0.86
C
ATOM   1917  O   VAL A 318      99.588  72.115 243.835  1.00  0.86
O
ATOM   1918  CB  VAL A 318     102.260  73.360 242.157  1.00  0.86
C
ATOM   1919  CG1 VAL A 318     101.460  74.605 242.592  1.00  0.86
C
ATOM   1920  CG2 VAL A 318     103.755  73.547 242.485  1.00  0.86
C
ATOM   1921  N   SER A 319      99.693  71.632 241.635  1.00  0.86
N
ATOM   1922  CA  SER A 319      98.264  71.438 241.402  1.00  0.86
C
ATOM   1923  C   SER A 319      97.676  70.353 242.283  1.00  0.86
C
ATOM   1924  O   SER A 319      96.599  70.473 242.860  1.00  0.86
O
ATOM   1925  CB  SER A 319      98.050  71.018 239.925  1.00  0.86
C
ATOM   1926  OG  SER A 319      96.722  71.237 239.444  1.00  0.86
O
ATOM   1927  N   ARG A 320      98.418  69.242 242.445  1.00  0.76
N
ATOM   1928  CA  ARG A 320      98.010  68.123 243.266  1.00  0.76
C
ATOM   1929  C   ARG A 320      97.826  68.443 244.738  1.00  0.76
C
ATOM   1930  O   ARG A 320      96.882  67.977 245.371  1.00  0.76
O
ATOM   1931  CB  ARG A 320      99.026  66.983 243.089  1.00  0.76
C
ATOM   1932  CG  ARG A 320      98.559  65.620 243.635  1.00  0.76
C
ATOM   1933  CD  ARG A 320      99.518  64.441 243.398  1.00  0.76
C
ATOM   1934  NE  ARG A 320     100.328  64.720 242.166  1.00  0.76
N
ATOM   1935  CZ  ARG A 320     100.694  63.789 241.277  1.00  0.76
C
ATOM   1936  NH1 ARG A 320     100.387  62.517 241.455  1.00  0.76
N
ATOM   1937  NH2 ARG A 320     101.251  64.164 240.123  1.00  0.76
N
```

```
ATOM   1938  N   LYS A 321      98.714  69.271 245.325  1.00  0.80
N
ATOM   1939  CA  LYS A 321      98.574  69.717 246.699  1.00  0.80
C
ATOM   1940  C   LYS A 321      97.338  70.588 246.910  1.00  0.80
C
ATOM   1941  O   LYS A 321      96.620  70.426 247.895  1.00  0.80
O
ATOM   1942  CB  LYS A 321      99.857  70.430 247.191  1.00  0.80
C
ATOM   1943  CG  LYS A 321     101.077  69.493 247.291  1.00  0.80
C
ATOM   1944  CD  LYS A 321     102.339  70.202 247.819  1.00  0.80
C
ATOM   1945  CE  LYS A 321     103.547  69.266 247.946  1.00  0.80
C
ATOM   1946  NZ  LYS A 321     104.728  70.013 248.435  1.00  0.80
N
ATOM   1947  N   GLU A 322      97.028  71.501 245.962  1.00  0.80
N
ATOM   1948  CA  GLU A 322      95.805  72.288 245.976  1.00  0.80
C
ATOM   1949  C   GLU A 322      94.545  71.431 245.873  1.00  0.80
C
ATOM   1950  O   GLU A 322      93.573  71.612 246.609  1.00  0.80
O
ATOM   1951  CB  GLU A 322      95.781  73.305 244.806  1.00  0.80
C
ATOM   1952  CG  GLU A 322      96.981  74.287 244.732  1.00  0.80
C
ATOM   1953  CD  GLU A 322      96.861  75.276 243.562  1.00  0.80
C
ATOM   1954  OE1 GLU A 322      97.830  76.049 243.340  1.00  0.80
O
ATOM   1955  OE2 GLU A 322      95.804  75.276 242.878  1.00  0.80
O
ATOM   1956  N   PHE A 323      94.549  70.435 244.962  1.00  0.83
N
ATOM   1957  CA  PHE A 323      93.464  69.489 244.779  1.00  0.83
C
ATOM   1958  C   PHE A 323      93.215  68.601 245.984  1.00  0.83
C
ATOM   1959  O   PHE A 323      92.068  68.347 246.349  1.00  0.83
O
ATOM   1960  CB  PHE A 323      93.694  68.594 243.533  1.00  0.83
C
ATOM   1961  CG  PHE A 323      93.568  69.312 242.209  1.00  0.83
C
ATOM   1962  CD1 PHE A 323      93.071  70.624 242.064  1.00  0.83
C
ATOM   1963  CD2 PHE A 323      93.948  68.615 241.050  1.00  0.83
C
ATOM   1964  CE1 PHE A 323      92.983  71.224 240.801  1.00  0.83
C
```

```
ATOM   1965  CE2 PHE A 323      93.846  69.208 239.787  1.00  0.83
C
ATOM   1966  CZ  PHE A 323      93.371  70.517 239.660  1.00  0.83
C
ATOM   1967  N   GLN A 324      94.284  68.128 246.653  1.00  0.82
N
ATOM   1968  CA  GLN A 324      94.191  67.291 247.837  1.00  0.82
C
ATOM   1969  C   GLN A 324      93.438  67.964 248.974  1.00  0.82
C
ATOM   1970  O   GLN A 324      92.574  67.354 249.595  1.00  0.82
O
ATOM   1971  CB  GLN A 324      95.597  66.827 248.282  1.00  0.82
C
ATOM   1972  CG  GLN A 324      95.608  65.774 249.416  1.00  0.82
C
ATOM   1973  CD  GLN A 324      94.901  64.464 249.051  1.00  0.82
C
ATOM   1974  OE1 GLN A 324      94.729  64.102 247.885  1.00  0.82
O
ATOM   1975  NE2 GLN A 324      94.481  63.714 250.095  1.00  0.82
N
ATOM   1976  N   THR A 325      93.661  69.282 249.183  1.00  0.85
N
ATOM   1977  CA  THR A 325      92.890  70.115 250.109  1.00  0.85
C
ATOM   1978  C   THR A 325      91.397  70.057 249.844  1.00  0.85
C
ATOM   1979  O   THR A 325      90.583  69.935 250.756  1.00  0.85
O
ATOM   1980  CB  THR A 325      93.287  71.585 249.991  1.00  0.85
C
ATOM   1981  OG1 THR A 325      94.645  71.774 250.351  1.00  0.85
O
ATOM   1982  CG2 THR A 325      92.476  72.520 250.904  1.00  0.85
C
ATOM   1983  N   ILE A 326      90.984  70.132 248.564  1.00  0.87
N
ATOM   1984  CA  ILE A 326      89.585  70.028 248.179  1.00  0.87
C
ATOM   1985  C   ILE A 326      89.016  68.633 248.429  1.00  0.87
C
ATOM   1986  O   ILE A 326      87.935  68.487 249.001  1.00  0.87
O
ATOM   1987  CB  ILE A 326      89.378  70.472 246.730  1.00  0.87
C
ATOM   1988  CG1 ILE A 326      89.853  71.935 246.542  1.00  0.87
C
ATOM   1989  CG2 ILE A 326      87.892  70.320 246.324  1.00  0.87
C
ATOM   1990  CD1 ILE A 326      89.954  72.356 245.071  1.00  0.87
C
ATOM   1991  N   TYR A 327      89.746  67.564 248.051  1.00  0.86
N
```

ATOM 1992 CA TYR A 327 89.313 66.188 248.249 1.00 0.86
C
ATOM 1993 C TYR A 327 89.190 65.769 249.711 1.00 0.86
C
ATOM 1994 O TYR A 327 88.228 65.097 250.080 1.00 0.86
O
ATOM 1995 CB TYR A 327 90.216 65.182 247.486 1.00 0.86
C
ATOM 1996 CG TYR A 327 90.149 65.374 245.988 1.00 0.86
C
ATOM 1997 CD1 TYR A 327 88.925 65.509 245.305 1.00 0.86
C
ATOM 1998 CD2 TYR A 327 91.336 65.392 245.238 1.00 0.86
C
ATOM 1999 CE1 TYR A 327 88.895 65.711 243.916 1.00 0.86
C
ATOM 2000 CE2 TYR A 327 91.310 65.608 243.855 1.00 0.86
C
ATOM 2001 CZ TYR A 327 90.093 65.788 243.197 1.00 0.86
C
ATOM 2002 OH TYR A 327 90.102 66.067 241.818 1.00 0.86
O
ATOM 2003 N GLU A 328 90.135 66.182 250.583 1.00 0.83
N
ATOM 2004 CA GLU A 328 90.088 65.945 252.019 1.00 0.83
C
ATOM 2005 C GLU A 328 88.910 66.617 252.698 1.00 0.83
C
ATOM 2006 O GLU A 328 88.196 65.998 253.483 1.00 0.83
O
ATOM 2007 CB GLU A 328 91.406 66.390 252.696 1.00 0.83
C
ATOM 2008 CG GLU A 328 92.594 65.481 252.294 1.00 0.83
C
ATOM 2009 CD GLU A 328 93.957 65.875 252.862 1.00 0.83
C
ATOM 2010 OE1 GLU A 328 94.052 66.889 253.594 1.00 0.83
O
ATOM 2011 OE2 GLU A 328 94.921 65.128 252.537 1.00 0.83
O
ATOM 2012 N ARG A 329 88.623 67.891 252.350 1.00 0.79
N
ATOM 2013 CA ARG A 329 87.448 68.599 252.831 1.00 0.79
C
ATOM 2014 C ARG A 329 86.151 67.921 252.416 1.00 0.79
C
ATOM 2015 O ARG A 329 85.198 67.818 253.181 1.00 0.79
O
ATOM 2016 CB ARG A 329 87.426 70.053 252.303 1.00 0.79
C
ATOM 2017 CG ARG A 329 88.477 70.990 252.935 1.00 0.79
C
ATOM 2018 CD ARG A 329 88.482 72.364 252.247 1.00 0.79
C

ATOM 2019 NE ARG A 329 89.562 73.210 252.849 1.00 0.79
N
ATOM 2020 CZ ARG A 329 89.673 74.534 252.687 1.00 0.79
C
ATOM 2021 NH1 ARG A 329 90.579 75.213 253.388 1.00 0.79
N
ATOM 2022 NH2 ARG A 329 88.921 75.225 251.851 1.00 0.79
N
ATOM 2023 N LEU A 330 86.092 67.409 251.174 1.00 0.89
N
ATOM 2024 CA LEU A 330 84.881 66.813 250.662 1.00 0.89
C
ATOM 2025 C LEU A 330 84.743 65.327 251.000 1.00 0.89
C
ATOM 2026 O LEU A 330 83.694 64.759 250.703 1.00 0.89
O
ATOM 2027 CB LEU A 330 84.798 67.012 249.125 1.00 0.89
C
ATOM 2028 CG LEU A 330 84.464 68.432 248.631 1.00 0.89
C
ATOM 2029 CD1 LEU A 330 84.780 68.549 247.131 1.00 0.89
C
ATOM 2030 CD2 LEU A 330 82.988 68.787 248.865 1.00 0.89
C
ATOM 2031 N ASP A 331 85.737 64.668 251.642 1.00 0.88
N
ATOM 2032 CA ASP A 331 85.687 63.257 252.023 1.00 0.88
C
ATOM 2033 C ASP A 331 85.678 62.341 250.787 1.00 0.88
C
ATOM 2034 O ASP A 331 84.857 61.439 250.639 1.00 0.88
O
ATOM 2035 CB ASP A 331 84.537 63.021 253.060 1.00 0.88
C
ATOM 2036 CG ASP A 331 84.471 61.641 253.702 1.00 0.88
C
ATOM 2037 OD1 ASP A 331 85.442 61.246 254.385 1.00 0.88
O
ATOM 2038 OD2 ASP A 331 83.394 60.989 253.564 1.00 0.88
O
ATOM 2039 N ILE A 332 86.597 62.590 249.826 1.00 0.88
N
ATOM 2040 CA ILE A 332 86.575 61.939 248.519 1.00 0.88
C
ATOM 2041 C ILE A 332 87.851 61.137 248.295 1.00 0.88
C
ATOM 2042 O ILE A 332 88.964 61.662 248.344 1.00 0.88
O
ATOM 2043 CB ILE A 332 86.423 62.958 247.387 1.00 0.88
C
ATOM 2044 CG1 ILE A 332 85.312 63.986 247.667 1.00 0.88
C
ATOM 2045 CG2 ILE A 332 86.174 62.276 246.031 1.00 0.88
C

```
ATOM   2046  CD1 ILE A 332      83.896  63.419 247.806  1.00  0.88
C
ATOM   2047  N   SER A 333      87.729  59.820 248.020  1.00  0.87
N
ATOM   2048  CA  SER A 333      88.881  58.960 247.749  1.00  0.87
C
ATOM   2049  C   SER A 333      88.932  58.572 246.286  1.00  0.87
C
ATOM   2050  O   SER A 333      87.956  58.129 245.683  1.00  0.87
O
ATOM   2051  CB  SER A 333      88.980  57.683 248.634  1.00  0.87
C
ATOM   2052  OG  SER A 333      87.947  56.733 248.363  1.00  0.87
O
ATOM   2053  N   VAL A 334      90.096  58.790 245.649  1.00  0.86
N
ATOM   2054  CA  VAL A 334      90.214  58.790 244.206  1.00  0.86
C
ATOM   2055  C   VAL A 334      91.603  58.313 243.828  1.00  0.86
C
ATOM   2056  O   VAL A 334      92.589  58.682 244.459  1.00  0.86
O
ATOM   2057  CB  VAL A 334      89.999  60.213 243.693  1.00  0.86
C
ATOM   2058  CG1 VAL A 334      90.524  60.426 242.281  1.00  0.86
C
ATOM   2059  CG2 VAL A 334      88.503  60.549 243.691  1.00  0.86
C
ATOM   2060  N   LYS A 335      91.733  57.489 242.765  1.00  0.80
N
ATOM   2061  CA  LYS A 335      93.023  57.163 242.196  1.00  0.80
C
ATOM   2062  C   LYS A 335      93.291  58.110 241.044  1.00  0.80
C
ATOM   2063  O   LYS A 335      92.549  58.163 240.062  1.00  0.80
O
ATOM   2064  CB  LYS A 335      93.076  55.695 241.696  1.00  0.80
C
ATOM   2065  CG  LYS A 335      94.446  55.227 241.166  1.00  0.80
C
ATOM   2066  CD  LYS A 335      94.431  53.742 240.756  1.00  0.80
C
ATOM   2067  CE  LYS A 335      95.825  53.157 240.506  1.00  0.80
C
ATOM   2068  NZ  LYS A 335      95.723  51.695 240.289  1.00  0.80
N
ATOM   2069  N   GLU A 336      94.361  58.917 241.144  1.00  0.79
N
ATOM   2070  CA  GLU A 336      94.827  59.745 240.052  1.00  0.79
C
ATOM   2071  C   GLU A 336      95.260  58.919 238.843  1.00  0.79
C
ATOM   2072  O   GLU A 336      95.972  57.920 238.963  1.00  0.79
O
```

ATOM 2073 CB GLU A 336 95.957 60.668 240.551 1.00 0.79
C
ATOM 2074 CG GLU A 336 96.586 61.582 239.479 1.00 0.79
C
ATOM 2075 CD GLU A 336 97.694 62.427 240.071 1.00 0.79
C
ATOM 2076 OE1 GLU A 336 98.712 62.612 239.353 1.00 0.79
O
ATOM 2077 OE2 GLU A 336 97.606 62.839 241.260 1.00 0.79
O
ATOM 2078 N ARG A 337 94.797 59.305 237.640 1.00 0.79
N
ATOM 2079 CA ARG A 337 95.126 58.600 236.424 1.00 0.79
C
ATOM 2080 C ARG A 337 94.778 59.475 235.240 1.00 0.79
C
ATOM 2081 O ARG A 337 93.853 59.206 234.475 1.00 0.79
O
ATOM 2082 CB ARG A 337 94.391 57.245 236.309 1.00 0.79
C
ATOM 2083 CG ARG A 337 94.915 56.378 235.155 1.00 0.79
C
ATOM 2084 CD ARG A 337 94.146 55.082 234.966 1.00 0.79
C
ATOM 2085 NE ARG A 337 94.487 54.189 236.124 1.00 0.79
N
ATOM 2086 CZ ARG A 337 94.033 52.935 236.177 1.00 0.79
C
ATOM 2087 NH1 ARG A 337 92.986 52.559 235.452 1.00 0.79
N
ATOM 2088 NH2 ARG A 337 94.662 51.983 236.871 1.00 0.79
N
ATOM 2089 N GLY A 338 95.519 60.590 235.103 1.00 0.87
N
ATOM 2090 CA GLY A 338 95.371 61.537 234.009 1.00 0.87
C
ATOM 2091 C GLY A 338 95.548 61.002 232.617 1.00 0.87
C
ATOM 2092 O GLY A 338 95.912 59.851 232.380 1.00 0.87
O
ATOM 2093 N GLU A 339 95.344 61.882 231.631 1.00 0.82
N
ATOM 2094 CA GLU A 339 95.525 61.606 230.223 1.00 0.82
C
ATOM 2095 C GLU A 339 96.940 61.147 229.880 1.00 0.82
C
ATOM 2096 O GLU A 339 97.151 60.305 229.003 1.00 0.82
O
ATOM 2097 CB GLU A 339 95.174 62.847 229.387 1.00 0.82
C
ATOM 2098 CG GLU A 339 93.677 63.223 229.425 1.00 0.82
C
ATOM 2099 CD GLU A 339 93.417 64.396 228.490 1.00 0.82
C

```
ATOM   2100  OE1 GLU A 339      93.145  65.517 228.984  1.00  0.82
O
ATOM   2101  OE2 GLU A 339      93.522  64.172 227.254  1.00  0.82
O
ATOM   2102  N   SER A 340      97.940  61.665 230.621  1.00  0.85
N
ATOM   2103  CA  SER A 340      99.346  61.331 230.518  1.00  0.85
C
ATOM   2104  C   SER A 340      99.666  59.866 230.701  1.00  0.85
C
ATOM   2105  O   SER A 340     100.568  59.318 230.071  1.00  0.85
O
ATOM   2106  CB  SER A 340     100.227  62.156 231.485  1.00  0.85
C
ATOM   2107  OG  SER A 340     100.076  61.801 232.861  1.00  0.85
O
ATOM   2108  N   PHE A 341      98.874  59.170 231.535  1.00  0.82
N
ATOM   2109  CA  PHE A 341      98.970  57.750 231.817  1.00  0.82
C
ATOM   2110  C   PHE A 341      98.835  56.884 230.563  1.00  0.82
C
ATOM   2111  O   PHE A 341      99.316  55.750 230.486  1.00  0.82
O
ATOM   2112  CB  PHE A 341      97.870  57.390 232.855  1.00  0.82
C
ATOM   2113  CG  PHE A 341      97.890  55.956 233.312  1.00  0.82
C
ATOM   2114  CD1 PHE A 341      98.696  55.540 234.381  1.00  0.82
C
ATOM   2115  CD2 PHE A 341      97.053  55.017 232.690  1.00  0.82
C
ATOM   2116  CE1 PHE A 341      98.674  54.206 234.810  1.00  0.82
C
ATOM   2117  CE2 PHE A 341      97.023  53.685 233.117  1.00  0.82
C
ATOM   2118  CZ  PHE A 341      97.842  53.276 234.174  1.00  0.82
C
ATOM   2119  N   TYR A 342      98.157  57.409 229.531  1.00  0.85
N
ATOM   2120  CA  TYR A 342      97.826  56.657 228.349  1.00  0.85
C
ATOM   2121  C   TYR A 342      98.780  56.909 227.197  1.00  0.85
C
ATOM   2122  O   TYR A 342      98.567  56.389 226.107  1.00  0.85
O
ATOM   2123  CB  TYR A 342      96.399  57.019 227.884  1.00  0.85
C
ATOM   2124  CG  TYR A 342      95.425  56.743 228.987  1.00  0.85
C
ATOM   2125  CD1 TYR A 342      95.078  55.437 229.346  1.00  0.85
C
ATOM   2126  CD2 TYR A 342      94.917  57.805 229.740  1.00  0.85
C
```

ATOM 2127 CE1 TYR A 342 94.235 55.218 230.442 1.00 0.85
C
ATOM 2128 CE2 TYR A 342 94.080 57.584 230.837 1.00 0.85
C
ATOM 2129 CZ TYR A 342 93.709 56.285 231.179 1.00 0.85
C
ATOM 2130 OH TYR A 342 92.802 56.116 232.249 1.00 0.85
O
ATOM 2131 N GLN A 343 99.873 57.676 227.390 1.00 0.79
N
ATOM 2132 CA GLN A 343 100.718 58.152 226.302 1.00 0.79
C
ATOM 2133 C GLN A 343 101.299 57.076 225.384 1.00 0.79
C
ATOM 2134 O GLN A 343 101.197 57.178 224.164 1.00 0.79
O
ATOM 2135 CB GLN A 343 101.804 59.111 226.856 1.00 0.79
C
ATOM 2136 CG GLN A 343 102.768 58.494 227.899 1.00 0.79
C
ATOM 2137 CD GLN A 343 103.651 59.568 228.547 1.00 0.79
C
ATOM 2138 OE1 GLN A 343 104.594 60.076 227.949 1.00 0.79
O
ATOM 2139 NE2 GLN A 343 103.350 59.918 229.818 1.00 0.79
N
ATOM 2140 N SER A 344 101.829 55.966 225.938 1.00 0.82
N
ATOM 2141 CA SER A 344 102.249 54.803 225.165 1.00 0.82
C
ATOM 2142 C SER A 344 101.120 54.120 224.411 1.00 0.82
C
ATOM 2143 O SER A 344 101.256 53.718 223.261 1.00 0.82
O
ATOM 2144 CB SER A 344 102.907 53.725 226.061 1.00 0.82
C
ATOM 2145 OG SER A 344 103.924 54.306 226.877 1.00 0.82
O
ATOM 2146 N ARG A 345 99.952 53.977 225.068 1.00 0.77
N
ATOM 2147 CA ARG A 345 98.766 53.341 224.533 1.00 0.77
C
ATOM 2148 C ARG A 345 98.128 54.128 223.398 1.00 0.77
C
ATOM 2149 O ARG A 345 97.559 53.548 222.482 1.00 0.77
O
ATOM 2150 CB ARG A 345 97.744 53.063 225.666 1.00 0.77
C
ATOM 2151 CG ARG A 345 98.188 51.924 226.608 1.00 0.77
C
ATOM 2152 CD ARG A 345 97.261 51.701 227.815 1.00 0.77
C
ATOM 2153 NE ARG A 345 97.931 52.250 229.050 1.00 0.77
N

```
ATOM   2154  CZ  ARG A 345      98.392  51.479 230.047  1.00  0.77
C
ATOM   2155  NH1 ARG A 345      98.319  50.161 230.033  1.00  0.77
N
ATOM   2156  NH2 ARG A 345      98.989  52.043 231.094  1.00  0.77
N
ATOM   2157  N   MET A 346      98.224  55.471 223.407  1.00  0.82
N
ATOM   2158  CA  MET A 346      97.722  56.329 222.350  1.00  0.82
C
ATOM   2159  C   MET A 346      98.353  56.087 220.984  1.00  0.82
C
ATOM   2160  O   MET A 346      97.648  56.119 219.978  1.00  0.82
O
ATOM   2161  CB  MET A 346      97.809  57.820 222.741  1.00  0.82
C
ATOM   2162  CG  MET A 346      96.778  58.217 223.816  1.00  0.82
C
ATOM   2163  SD  MET A 346      96.577  60.008 224.033  1.00  0.82
S
ATOM   2164  CE  MET A 346      98.108  60.349 224.929  1.00  0.82
C
ATOM   2165  N   LEU A 347      99.670  55.775 220.906  1.00  0.83
N
ATOM   2166  CA  LEU A 347     100.301  55.372 219.652  1.00  0.83
C
ATOM   2167  C   LEU A 347      99.640  54.128 219.050  1.00  0.83
C
ATOM   2168  O   LEU A 347      99.230  54.112 217.891  1.00  0.83
O
ATOM   2169  CB  LEU A 347     101.813  55.077 219.857  1.00  0.83
C
ATOM   2170  CG  LEU A 347     102.695  56.298 220.203  1.00  0.83
C
ATOM   2171  CD1 LEU A 347     104.030  55.838 220.813  1.00  0.83
C
ATOM   2172  CD2 LEU A 347     102.989  57.185 218.982  1.00  0.83
C
ATOM   2173  N   SER A 348      99.424  53.088 219.881  1.00  0.85
N
ATOM   2174  CA  SER A 348      98.744  51.854 219.513  1.00  0.85
C
ATOM   2175  C   SER A 348      97.303  52.038 219.074  1.00  0.85
C
ATOM   2176  O   SER A 348      96.815  51.363 218.171  1.00  0.85
O
ATOM   2177  CB  SER A 348      98.726  50.833 220.677  1.00  0.85
C
ATOM   2178  OG  SER A 348     100.042  50.639 221.194  1.00  0.85
O
ATOM   2179  N   VAL A 349      96.561  52.957 219.724  1.00  0.87
N
ATOM   2180  CA  VAL A 349      95.181  53.288 219.386  1.00  0.87
C
```

```
ATOM   2181  C   VAL A 349      95.053  53.844 217.982  1.00  0.87
C
ATOM   2182  O   VAL A 349      94.201  53.410 217.205  1.00  0.87
O
ATOM   2183  CB  VAL A 349      94.609  54.269 220.404  1.00  0.87
C
ATOM   2184  CG1 VAL A 349      93.258  54.886 219.983  1.00  0.87
C
ATOM   2185  CG2 VAL A 349      94.422  53.532 221.740  1.00  0.87
C
ATOM   2186  N   VAL A 350      95.943  54.779 217.604  1.00  0.85
N
ATOM   2187  CA  VAL A 350      95.982  55.358 216.275  1.00  0.85
C
ATOM   2188  C   VAL A 350      96.315  54.340 215.197  1.00  0.85
C
ATOM   2189  O   VAL A 350      95.644  54.241 214.168  1.00  0.85
O
ATOM   2190  CB  VAL A 350      96.992  56.489 216.250  1.00  0.85
C
ATOM   2191  CG1 VAL A 350      97.245  56.970 214.812  1.00  0.85
C
ATOM   2192  CG2 VAL A 350      96.470  57.635 217.137  1.00  0.85
C
ATOM   2193  N   GLU A 351      97.341  53.499 215.437  1.00  0.80
N
ATOM   2194  CA  GLU A 351      97.719  52.439 214.529  1.00  0.80
C
ATOM   2195  C   GLU A 351      96.641  51.394 214.341  1.00  0.80
C
ATOM   2196  O   GLU A 351      96.417  50.920 213.230  1.00  0.80
O
ATOM   2197  CB  GLU A 351      99.030  51.761 214.957  1.00  0.80
C
ATOM   2198  CG  GLU A 351     100.257  52.695 214.831  1.00  0.80
C
ATOM   2199  CD  GLU A 351     101.582  51.953 215.004  1.00  0.80
C
ATOM   2200  OE1 GLU A 351     101.545  50.719 215.228  1.00  0.80
O
ATOM   2201  OE2 GLU A 351     102.635  52.618 214.837  1.00  0.80
O
ATOM   2202  N   TYR A 352      95.916  51.029 215.414  1.00  0.82
N
ATOM   2203  CA  TYR A 352      94.783  50.131 215.336  1.00  0.82
C
ATOM   2204  C   TYR A 352      93.636  50.673 214.483  1.00  0.82
C
ATOM   2205  O   TYR A 352      93.105  49.964 213.633  1.00  0.82
O
ATOM   2206  CB  TYR A 352      94.298  49.805 216.773  1.00  0.82
C
ATOM   2207  CG  TYR A 352      93.332  48.652 216.788  1.00  0.82
C
```

```
ATOM   2208  CD1 TYR A 352      91.956  48.853 216.992  1.00  0.82
C
ATOM   2209  CD2 TYR A 352      93.806  47.352 216.560  1.00  0.82
C
ATOM   2210  CE1 TYR A 352      91.067  47.767 216.954  1.00  0.82
C
ATOM   2211  CE2 TYR A 352      92.921  46.268 216.534  1.00  0.82
C
ATOM   2212  CZ  TYR A 352      91.554  46.475 216.724  1.00  0.82
C
ATOM   2213  OH  TYR A 352      90.689  45.367 216.684  1.00  0.82
O
ATOM   2214  N   LEU A 353      93.255  51.958 214.645  1.00  0.85
N
ATOM   2215  CA  LEU A 353      92.249  52.584 213.797  1.00  0.85
C
ATOM   2216  C   LEU A 353      92.682  52.682 212.337  1.00  0.85
C
ATOM   2217  O   LEU A 353      91.909  52.392 211.425  1.00  0.85
O
ATOM   2218  CB  LEU A 353      91.829  53.974 214.335  1.00  0.85
C
ATOM   2219  CG  LEU A 353      91.088  53.941 215.693  1.00  0.85
C
ATOM   2220  CD1 LEU A 353      90.841  55.364 216.215  1.00  0.85
C
ATOM   2221  CD2 LEU A 353      89.747  53.193 215.619  1.00  0.85
C
ATOM   2222  N   ARG A 354      93.960  53.032 212.081  1.00  0.75
N
ATOM   2223  CA  ARG A 354      94.550  53.005 210.756  1.00  0.75
C
ATOM   2224  C   ARG A 354      94.570  51.613 210.137  1.00  0.75
C
ATOM   2225  O   ARG A 354      94.206  51.427 208.980  1.00  0.75
O
ATOM   2226  CB  ARG A 354      96.004  53.541 210.829  1.00  0.75
C
ATOM   2227  CG  ARG A 354      96.730  53.668 209.470  1.00  0.75
C
ATOM   2228  CD  ARG A 354      98.220  54.043 209.560  1.00  0.75
C
ATOM   2229  NE  ARG A 354      98.952  52.951 210.297  1.00  0.75
N
ATOM   2230  CZ  ARG A 354      99.332  51.776 209.775  1.00  0.75
C
ATOM   2231  NH1 ARG A 354      99.974  50.899 210.546  1.00  0.75
N
ATOM   2232  NH2 ARG A 354      99.093  51.462 208.504  1.00  0.75
N
ATOM   2233  N   GLY A 355      94.969  50.576 210.900  1.00  0.84
N
ATOM   2234  CA  GLY A 355      95.099  49.223 210.367  1.00  0.84
C
```

ATOM 2235 C GLY A 355 93.806 48.470 210.247 1.00 0.84
C
ATOM 2236 O GLY A 355 93.757 47.399 209.654 1.00 0.84
O
ATOM 2237 N LYS A 356 92.710 49.024 210.786 1.00 0.80
N
ATOM 2238 CA LYS A 356 91.373 48.517 210.560 1.00 0.80
C
ATOM 2239 C LYS A 356 90.633 49.365 209.549 1.00 0.80
C
ATOM 2240 O LYS A 356 89.459 49.127 209.277 1.00 0.80
O
ATOM 2241 CB LYS A 356 90.565 48.448 211.881 1.00 0.80
C
ATOM 2242 CG LYS A 356 91.071 47.381 212.869 1.00 0.80
C
ATOM 2243 CD LYS A 356 90.955 45.948 212.323 1.00 0.80
C
ATOM 2244 CE LYS A 356 91.291 44.886 213.369 1.00 0.80
C
ATOM 2245 NZ LYS A 356 91.203 43.543 212.759 1.00 0.80
N
ATOM 2246 N GLY A 357 91.308 50.357 208.928 1.00 0.86
N
ATOM 2247 CA GLY A 357 90.734 51.102 207.815 1.00 0.86
C
ATOM 2248 C GLY A 357 89.726 52.139 208.204 1.00 0.86
C
ATOM 2249 O GLY A 357 88.880 52.510 207.401 1.00 0.86
O
ATOM 2250 N LEU A 358 89.781 52.630 209.452 1.00 0.83
N
ATOM 2251 CA LEU A 358 88.813 53.568 209.981 1.00 0.83
C
ATOM 2252 C LEU A 358 89.403 54.964 210.075 1.00 0.83
C
ATOM 2253 O LEU A 358 89.012 55.750 210.929 1.00 0.83
O
ATOM 2254 CB LEU A 358 88.382 53.162 211.412 1.00 0.83
C
ATOM 2255 CG LEU A 358 87.835 51.734 211.558 1.00 0.83
C
ATOM 2256 CD1 LEU A 358 87.531 51.458 213.038 1.00 0.83
C
ATOM 2257 CD2 LEU A 358 86.622 51.474 210.648 1.00 0.83
C
ATOM 2258 N LEU A 359 90.431 55.267 209.260 1.00 0.82
N
ATOM 2259 CA LEU A 359 91.177 56.487 209.423 1.00 0.82
C
ATOM 2260 C LEU A 359 91.321 57.264 208.140 1.00 0.82
C
ATOM 2261 O LEU A 359 91.840 56.780 207.132 1.00 0.82
O

ATOM 2262 CB LEU A 359 92.581 56.206 209.999 1.00 0.82
C
ATOM 2263 CG LEU A 359 93.302 57.458 210.526 1.00 0.82
C
ATOM 2264 CD1 LEU A 359 92.581 58.010 211.758 1.00 0.82
C
ATOM 2265 CD2 LEU A 359 94.758 57.148 210.877 1.00 0.82
C
ATOM 2266 N GLU A 360 90.902 58.536 208.189 1.00 0.79
N
ATOM 2267 CA GLU A 360 90.900 59.438 207.074 1.00 0.79
C
ATOM 2268 C GLU A 360 91.869 60.589 207.336 1.00 0.79
C
ATOM 2269 O GLU A 360 91.968 61.124 208.440 1.00 0.79
O
ATOM 2270 CB GLU A 360 89.465 59.974 206.842 1.00 0.79
C
ATOM 2271 CG GLU A 360 88.406 58.872 206.530 1.00 0.79
C
ATOM 2272 CD GLU A 360 87.863 58.092 207.742 1.00 0.79
C
ATOM 2273 OE1 GLU A 360 87.847 56.839 207.661 1.00 0.79
O
ATOM 2274 OE2 GLU A 360 87.423 58.731 208.735 1.00 0.79
O
ATOM 2275 N VAL A 361 92.651 60.999 206.313 1.00 0.80
N
ATOM 2276 CA VAL A 361 93.590 62.115 206.433 1.00 0.80
C
ATOM 2277 C VAL A 361 92.948 63.400 205.941 1.00 0.80
C
ATOM 2278 O VAL A 361 92.575 63.516 204.777 1.00 0.80
O
ATOM 2279 CB VAL A 361 94.897 61.903 205.667 1.00 0.80
C
ATOM 2280 CG1 VAL A 361 95.801 63.159 205.735 1.00 0.80
C
ATOM 2281 CG2 VAL A 361 95.650 60.697 206.260 1.00 0.80
C
ATOM 2282 N ASP A 362 92.831 64.407 206.827 1.00 0.77
N
ATOM 2283 CA ASP A 362 92.162 65.656 206.533 1.00 0.77
C
ATOM 2284 C ASP A 362 92.963 66.831 207.092 1.00 0.77
C
ATOM 2285 O ASP A 362 93.203 66.930 208.289 1.00 0.77
O
ATOM 2286 CB ASP A 362 90.753 65.555 207.149 1.00 0.77
C
ATOM 2287 CG ASP A 362 89.892 66.744 206.793 1.00 0.77
C
ATOM 2288 OD1 ASP A 362 89.714 67.016 205.584 1.00 0.77
O

ATOM	2289	OD2	ASP	A	362	89.416	67.400	207.749	1.00	0.77	O
ATOM	2290	N	GLU	A	363	93.453	67.745	206.226	1.00	0.73	N
ATOM	2291	CA	GLU	A	363	94.282	68.897	206.598	1.00	0.73	C
ATOM	2292	C	GLU	A	363	95.552	68.561	207.397	1.00	0.73	C
ATOM	2293	O	GLU	A	363	96.070	69.326	208.211	1.00	0.73	O
ATOM	2294	CB	GLU	A	363	93.465	69.980	207.333	1.00	0.73	C
ATOM	2295	CG	GLU	A	363	92.263	70.532	206.533	1.00	0.73	C
ATOM	2296	CD	GLU	A	363	91.494	71.595	207.322	1.00	0.73	C
ATOM	2297	OE1	GLU	A	363	90.689	72.315	206.680	1.00	0.73	O
ATOM	2298	OE2	GLU	A	363	91.708	71.699	208.559	1.00	0.73	O
ATOM	2299	N	GLY	A	364	96.108	67.358	207.162	1.00	0.81	N
ATOM	2300	CA	GLY	A	364	97.233	66.825	207.919	1.00	0.81	C
ATOM	2301	C	GLY	A	364	96.835	66.195	209.227	1.00	0.81	C
ATOM	2302	O	GLY	A	364	97.677	65.662	209.931	1.00	0.81	O
ATOM	2303	N	ARG	A	365	95.539	66.199	209.575	1.00	0.73	N
ATOM	2304	CA	ARG	A	365	95.027	65.533	210.749	1.00	0.73	C
ATOM	2305	C	ARG	A	365	94.609	64.125	210.406	1.00	0.73	C
ATOM	2306	O	ARG	A	365	94.133	63.844	209.308	1.00	0.73	O
ATOM	2307	CB	ARG	A	365	93.764	66.224	211.314	1.00	0.73	C
ATOM	2308	CG	ARG	A	365	93.954	67.708	211.664	1.00	0.73	C
ATOM	2309	CD	ARG	A	365	92.653	68.381	212.108	1.00	0.73	C
ATOM	2310	NE	ARG	A	365	91.729	68.361	210.932	1.00	0.73	N
ATOM	2311	CZ	ARG	A	365	90.402	68.491	211.016	1.00	0.73	C
ATOM	2312	NH1	ARG	A	365	89.750	68.360	212.170	1.00	0.73	N
ATOM	2313	NH2	ARG	A	365	89.693	68.750	209.927	1.00	0.73	N
ATOM	2314	N	GLU	A	366	94.733	63.218	211.379	1.00	0.77	N
ATOM	2315	CA	GLU	A	366	94.196	61.885	211.277	1.00	0.77	C

ATOM 2316 C GLU A 366 92.860 61.869 211.990 1.00 0.77
C
ATOM 2317 O GLU A 366 92.774 62.085 213.202 1.00 0.77
O
ATOM 2318 CB GLU A 366 95.173 60.870 211.887 1.00 0.77
C
ATOM 2319 CG GLU A 366 96.491 60.746 211.088 1.00 0.77
C
ATOM 2320 CD GLU A 366 97.395 59.623 211.596 1.00 0.77
C
ATOM 2321 OE1 GLU A 366 97.074 59.032 212.655 1.00 0.77
O
ATOM 2322 OE2 GLU A 366 98.397 59.321 210.899 1.00 0.77
O
ATOM 2323 N ILE A 367 91.761 61.670 211.237 1.00 0.82
N
ATOM 2324 CA ILE A 367 90.417 61.780 211.766 1.00 0.82
C
ATOM 2325 C ILE A 367 89.659 60.503 211.498 1.00 0.82
C
ATOM 2326 O ILE A 367 89.961 59.765 210.567 1.00 0.82
O
ATOM 2327 CB ILE A 367 89.622 62.992 211.267 1.00 0.82
C
ATOM 2328 CG1 ILE A 367 89.108 62.830 209.815 1.00 0.82
C
ATOM 2329 CG2 ILE A 367 90.493 64.259 211.431 1.00 0.82
C
ATOM 2330 CD1 ILE A 367 88.275 64.018 209.323 1.00 0.82
C
ATOM 2331 N MET A 368 88.668 60.200 212.348 1.00 0.80
N
ATOM 2332 CA MET A 368 87.878 58.994 212.243 1.00 0.80
C
ATOM 2333 C MET A 368 86.411 59.348 212.174 1.00 0.80
C
ATOM 2334 O MET A 368 85.849 59.853 213.148 1.00 0.80
O
ATOM 2335 CB MET A 368 88.101 58.127 213.511 1.00 0.80
C
ATOM 2336 CG MET A 368 87.238 56.852 213.603 1.00 0.80
C
ATOM 2337 SD MET A 368 87.322 56.050 215.228 1.00 0.80
S
ATOM 2338 CE MET A 368 86.224 54.682 214.772 1.00 0.80
C
ATOM 2339 N TRP A 369 85.729 59.058 211.049 1.00 0.78
N
ATOM 2340 CA TRP A 369 84.277 59.155 210.985 1.00 0.78
C
ATOM 2341 C TRP A 369 83.593 57.876 211.510 1.00 0.78
C
ATOM 2342 O TRP A 369 83.812 56.797 210.956 1.00 0.78
O

ATOM 2343 CB TRP A 369 83.758 59.469 209.554 1.00 0.78
C
ATOM 2344 CG TRP A 369 84.133 60.850 209.021 1.00 0.78
C
ATOM 2345 CD1 TRP A 369 85.289 61.233 208.399 1.00 0.78
C
ATOM 2346 CD2 TRP A 369 83.288 62.016 209.049 1.00 0.78
C
ATOM 2347 NE1 TRP A 369 85.239 62.569 208.072 1.00 0.78
N
ATOM 2348 CE2 TRP A 369 84.023 63.071 208.451 1.00 0.78
C
ATOM 2349 CE3 TRP A 369 81.995 62.226 209.520 1.00 0.78
C
ATOM 2350 CZ2 TRP A 369 83.478 64.337 208.323 1.00 0.78
C
ATOM 2351 CZ3 TRP A 369 81.442 63.505 209.371 1.00 0.78
C
ATOM 2352 CH2 TRP A 369 82.173 64.549 208.785 1.00 0.78
C
ATOM 2353 N PRO A 370 82.752 57.884 212.555 1.00 0.79
N
ATOM 2354 CA PRO A 370 81.969 56.710 212.917 1.00 0.79
C
ATOM 2355 C PRO A 370 80.560 56.829 212.393 1.00 0.79
C
ATOM 2356 O PRO A 370 79.867 57.762 212.766 1.00 0.79
O
ATOM 2357 CB PRO A 370 81.956 56.688 214.455 1.00 0.79
C
ATOM 2358 CG PRO A 370 82.489 58.061 214.897 1.00 0.79
C
ATOM 2359 CD PRO A 370 82.695 58.885 213.617 1.00 0.79
C
ATOM 2360 N ASP A 371 80.085 55.878 211.567 1.00 0.75
N
ATOM 2361 CA ASP A 371 78.791 55.931 210.908 1.00 0.75
C
ATOM 2362 C ASP A 371 77.588 56.123 211.826 1.00 0.75
C
ATOM 2363 O ASP A 371 76.600 56.765 211.478 1.00 0.75
O
ATOM 2364 CB ASP A 371 78.553 54.604 210.141 1.00 0.75
C
ATOM 2365 CG ASP A 371 79.617 54.345 209.086 1.00 0.75
C
ATOM 2366 OD1 ASP A 371 80.172 55.306 208.513 1.00 0.75
O
ATOM 2367 OD2 ASP A 371 79.966 53.144 208.905 1.00 0.75
O
ATOM 2368 N ASP A 372 77.668 55.553 213.033 1.00 0.64
N
ATOM 2369 CA ASP A 372 76.666 55.499 214.053 1.00 0.64
C

ATOM 2370 C ASP A 372 76.797 56.622 215.082 1.00 0.64
C
ATOM 2371 O ASP A 372 76.112 56.623 216.108 1.00 0.64
O
ATOM 2372 CB ASP A 372 76.783 54.108 214.739 1.00 0.64
C
ATOM 2373 CG ASP A 372 78.187 53.711 215.207 1.00 0.64
C
ATOM 2374 OD1 ASP A 372 79.220 54.198 214.671 1.00 0.64
O
ATOM 2375 OD2 ASP A 372 78.216 52.873 216.137 1.00 0.64
O
ATOM 2376 N THR A 373 77.664 57.625 214.815 1.00 0.47
N
ATOM 2377 CA THR A 373 77.855 58.824 215.627 1.00 0.47
C
ATOM 2378 C THR A 373 76.561 59.584 215.900 1.00 0.47
C
ATOM 2379 O THR A 373 75.699 59.740 215.041 1.00 0.47
O
ATOM 2380 CB THR A 373 78.894 59.775 215.025 1.00 0.47
C
ATOM 2381 OG1 THR A 373 79.133 60.917 215.838 1.00 0.47
O
ATOM 2382 CG2 THR A 373 78.447 60.260 213.637 1.00 0.47
C
ATOM 2383 N LYS A 374 76.368 60.087 217.140 1.00 0.44
N
ATOM 2384 CA LYS A 374 75.129 60.763 217.494 1.00 0.44
C
ATOM 2385 C LYS A 374 75.000 62.149 216.876 1.00 0.44
C
ATOM 2386 O LYS A 374 73.898 62.647 216.656 1.00 0.44
O
ATOM 2387 CB LYS A 374 75.017 60.876 219.038 1.00 0.44
C
ATOM 2388 CG LYS A 374 73.733 61.531 219.597 1.00 0.44
C
ATOM 2389 CD LYS A 374 72.433 60.800 219.214 1.00 0.44
C
ATOM 2390 CE LYS A 374 71.174 61.467 219.783 1.00 0.44
C
ATOM 2391 NZ LYS A 374 69.965 60.736 219.338 1.00 0.44
N
ATOM 2392 N THR A 375 76.137 62.809 216.600 1.00 0.52
N
ATOM 2393 CA THR A 375 76.166 64.205 216.194 1.00 0.52
C
ATOM 2394 C THR A 375 76.690 64.441 214.788 1.00 0.52
C
ATOM 2395 O THR A 375 76.662 65.563 214.292 1.00 0.52
O
ATOM 2396 CB THR A 375 77.126 65.001 217.071 1.00 0.52
C

ATOM 2397 OG1 THR A 375 78.353 64.301 217.271 1.00 0.52
O
ATOM 2398 CG2 THR A 375 76.515 65.222 218.453 1.00 0.52
C
ATOM 2399 N GLY A 376 77.228 63.413 214.103 1.00 0.74
N
ATOM 2400 CA GLY A 376 77.947 63.607 212.842 1.00 0.74
C
ATOM 2401 C GLY A 376 79.367 64.081 213.016 1.00 0.74
C
ATOM 2402 O GLY A 376 80.047 64.386 212.048 1.00 0.74
O
ATOM 2403 N ILE A 377 79.874 64.161 214.260 1.00 0.78
N
ATOM 2404 CA ILE A 377 81.235 64.617 214.520 1.00 0.78
C
ATOM 2405 C ILE A 377 82.264 63.505 214.281 1.00 0.78
C
ATOM 2406 O ILE A 377 82.113 62.424 214.859 1.00 0.78
O
ATOM 2407 CB ILE A 377 81.385 65.190 215.931 1.00 0.78
C
ATOM 2408 CG1 ILE A 377 80.537 66.479 216.054 1.00 0.78
C
ATOM 2409 CG2 ILE A 377 82.867 65.482 216.260 1.00 0.78
C
ATOM 2410 CD1 ILE A 377 80.457 67.049 217.476 1.00 0.78
C
ATOM 2411 N PRO A 378 83.312 63.687 213.467 1.00 0.84
N
ATOM 2412 CA PRO A 378 84.460 62.792 213.446 1.00 0.84
C
ATOM 2413 C PRO A 378 85.374 63.018 214.641 1.00 0.84
C
ATOM 2414 O PRO A 378 85.552 64.152 215.078 1.00 0.84
O
ATOM 2415 CB PRO A 378 85.178 63.181 212.145 1.00 0.84
C
ATOM 2416 CG PRO A 378 84.917 64.683 212.009 1.00 0.84
C
ATOM 2417 CD PRO A 378 83.495 64.824 212.556 1.00 0.84
C
ATOM 2418 N LEU A 379 85.976 61.949 215.191 1.00 0.85
N
ATOM 2419 CA LEU A 379 87.022 62.072 216.194 1.00 0.85
C
ATOM 2420 C LEU A 379 88.339 62.527 215.548 1.00 0.85
C
ATOM 2421 O LEU A 379 88.748 61.981 214.524 1.00 0.85
O
ATOM 2422 CB LEU A 379 87.250 60.725 216.933 1.00 0.85
C
ATOM 2423 CG LEU A 379 86.026 60.153 217.684 1.00 0.85
C

```
ATOM   2424  CD1 LEU A 379      86.344  58.761 218.253  1.00  0.85
C
ATOM   2425  CD2 LEU A 379      85.558  61.073 218.821  1.00  0.85
C
ATOM   2426  N   THR A 380      89.054  63.523 216.115  1.00  0.85
N
ATOM   2427  CA  THR A 380      90.378  63.932 215.624  1.00  0.85
C
ATOM   2428  C   THR A 380      91.405  63.233 216.489  1.00  0.85
C
ATOM   2429  O   THR A 380      91.670  63.654 217.609  1.00  0.85
O
ATOM   2430  CB  THR A 380      90.716  65.438 215.701  1.00  0.85
C
ATOM   2431  OG1 THR A 380      89.878  66.252 214.885  1.00  0.85
O
ATOM   2432  CG2 THR A 380      92.168  65.686 215.236  1.00  0.85
C
ATOM   2433  N   ILE A 381      92.050  62.151 216.011  1.00  0.84
N
ATOM   2434  CA  ILE A 381      92.896  61.347 216.883  1.00  0.84
C
ATOM   2435  C   ILE A 381      94.366  61.706 216.784  1.00  0.84
C
ATOM   2436  O   ILE A 381      95.140  61.361 217.674  1.00  0.84
O
ATOM   2437  CB  ILE A 381      92.744  59.853 216.644  1.00  0.84
C
ATOM   2438  CG1 ILE A 381      93.094  59.459 215.196  1.00  0.84
C
ATOM   2439  CG2 ILE A 381      91.311  59.439 217.045  1.00  0.84
C
ATOM   2440  CD1 ILE A 381      93.165  57.947 215.013  1.00  0.84
C
ATOM   2441  N   VAL A 382      94.780  62.452 215.740  1.00  0.82
N
ATOM   2442  CA  VAL A 382      96.100  63.058 215.668  1.00  0.82
C
ATOM   2443  C   VAL A 382      95.956  64.392 214.970  1.00  0.82
C
ATOM   2444  O   VAL A 382      95.241  64.529 213.976  1.00  0.82
O
ATOM   2445  CB  VAL A 382      97.148  62.256 214.900  1.00  0.82
C
ATOM   2446  CG1 VAL A 382      98.547  62.855 215.114  1.00  0.82
C
ATOM   2447  CG2 VAL A 382      97.173  60.802 215.378  1.00  0.82
C
ATOM   2448  N   LYS A 383      96.618  65.436 215.491  1.00  0.75
N
ATOM   2449  CA  LYS A 383      96.624  66.748 214.891  1.00  0.75
C
ATOM   2450  C   LYS A 383      97.697  66.864 213.823  1.00  0.75
C
```

```
ATOM   2451  O   LYS A 383      98.531  65.981 213.653  1.00  0.75
O
ATOM   2452  CB  LYS A 383      96.832  67.840 215.960  1.00  0.75
C
ATOM   2453  CG  LYS A 383      95.901  67.700 217.173  1.00  0.75
C
ATOM   2454  CD  LYS A 383      95.904  68.970 218.035  1.00  0.75
C
ATOM   2455  CE  LYS A 383      97.247  69.218 218.729  1.00  0.75
C
ATOM   2456  NZ  LYS A 383      97.249  70.568 219.321  1.00  0.75
N
ATOM   2457  N   SER A 384      97.727  67.983 213.070  1.00  0.76
N
ATOM   2458  CA  SER A 384      98.661  68.218 211.973  1.00  0.76
C
ATOM   2459  C   SER A 384     100.123  68.165 212.386  1.00  0.76
C
ATOM   2460  O   SER A 384     101.012  67.871 211.594  1.00  0.76
O
ATOM   2461  CB  SER A 384      98.398  69.588 211.297  1.00  0.76
C
ATOM   2462  OG  SER A 384      96.999  69.781 211.055  1.00  0.76
O
ATOM   2463  N   ASP A 385     100.378  68.443 213.674  1.00  0.72
N
ATOM   2464  CA  ASP A 385     101.673  68.513 214.300  1.00  0.72
C
ATOM   2465  C   ASP A 385     102.125  67.176 214.898  1.00  0.72
C
ATOM   2466  O   ASP A 385     103.227  67.051 215.433  1.00  0.72
O
ATOM   2467  CB  ASP A 385     101.585  69.608 215.409  1.00  0.72
C
ATOM   2468  CG  ASP A 385     100.337  69.480 216.276  1.00  0.72
C
ATOM   2469  OD1 ASP A 385     100.189  68.438 216.957  1.00  0.72
O
ATOM   2470  OD2 ASP A 385      99.476  70.399 216.260  1.00  0.72
O
ATOM   2471  N   GLY A 386     101.283  66.128 214.788  1.00  0.78
N
ATOM   2472  CA  GLY A 386     101.532  64.807 215.347  1.00  0.78
C
ATOM   2473  C   GLY A 386     101.124  64.672 216.790  1.00  0.78
C
ATOM   2474  O   GLY A 386     101.249  63.604 217.386  1.00  0.78
O
ATOM   2475  N   GLY A 387     100.607  65.754 217.397  1.00  0.77
N
ATOM   2476  CA  GLY A 387     100.056  65.780 218.738  1.00  0.77
C
ATOM   2477  C   GLY A 387      98.787  65.004 218.920  1.00  0.77
C
```

ATOM 2478 O GLY A 387 97.877 65.024 218.090 1.00 0.77
O
ATOM 2479 N PHE A 388 98.671 64.343 220.079 1.00 0.77
N
ATOM 2480 CA PHE A 388 97.465 63.647 220.470 1.00 0.77
C
ATOM 2481 C PHE A 388 96.432 64.588 221.075 1.00 0.77
C
ATOM 2482 O PHE A 388 96.687 65.760 221.360 1.00 0.77
O
ATOM 2483 CB PHE A 388 97.771 62.459 221.421 1.00 0.77
C
ATOM 2484 CG PHE A 388 98.550 61.371 220.721 1.00 0.77
C
ATOM 2485 CD1 PHE A 388 98.142 60.873 219.472 1.00 0.77
C
ATOM 2486 CD2 PHE A 388 99.692 60.812 221.318 1.00 0.77
C
ATOM 2487 CE1 PHE A 388 98.868 59.869 218.827 1.00 0.77
C
ATOM 2488 CE2 PHE A 388 100.424 59.806 220.677 1.00 0.77
C
ATOM 2489 CZ PHE A 388 100.012 59.342 219.427 1.00 0.77
C
ATOM 2490 N THR A 389 95.190 64.092 221.221 1.00 0.80
N
ATOM 2491 CA THR A 389 94.038 64.883 221.613 1.00 0.80
C
ATOM 2492 C THR A 389 93.299 64.214 222.754 1.00 0.80
C
ATOM 2493 O THR A 389 93.613 63.098 223.168 1.00 0.80
O
ATOM 2494 CB THR A 389 93.038 65.134 220.470 1.00 0.80
C
ATOM 2495 OG1 THR A 389 92.266 63.982 220.161 1.00 0.80
O
ATOM 2496 CG2 THR A 389 93.778 65.528 219.187 1.00 0.80
C
ATOM 2497 N TYR A 390 92.252 64.904 223.250 1.00 0.85
N
ATOM 2498 CA TYR A 390 91.271 64.407 224.195 1.00 0.85
C
ATOM 2499 C TYR A 390 90.532 63.173 223.663 1.00 0.85
C
ATOM 2500 O TYR A 390 90.303 62.197 224.372 1.00 0.85
O
ATOM 2501 CB TYR A 390 90.302 65.587 224.475 1.00 0.85
C
ATOM 2502 CG TYR A 390 89.333 65.344 225.597 1.00 0.85
C
ATOM 2503 CD1 TYR A 390 89.784 65.073 226.899 1.00 0.85
C
ATOM 2504 CD2 TYR A 390 87.955 65.484 225.368 1.00 0.85
C

ATOM 2505 CE1 TYR A 390 88.870 64.960 227.957 1.00 0.85
C
ATOM 2506 CE2 TYR A 390 87.042 65.375 226.425 1.00 0.85
C
ATOM 2507 CZ TYR A 390 87.501 65.118 227.721 1.00 0.85
C
ATOM 2508 OH TYR A 390 86.585 65.073 228.789 1.00 0.85
O
ATOM 2509 N ASP A 391 90.192 63.154 222.356 1.00 0.86
N
ATOM 2510 CA ASP A 391 89.610 62.011 221.682 1.00 0.86
C
ATOM 2511 C ASP A 391 90.534 60.799 221.741 1.00 0.86
C
ATOM 2512 O ASP A 391 90.120 59.674 222.019 1.00 0.86
O
ATOM 2513 CB ASP A 391 89.336 62.314 220.179 1.00 0.86
C
ATOM 2514 CG ASP A 391 88.510 63.571 219.919 1.00 0.86
C
ATOM 2515 OD1 ASP A 391 88.015 64.201 220.878 1.00 0.86
O
ATOM 2516 OD2 ASP A 391 88.409 63.931 218.714 1.00 0.86
O
ATOM 2517 N THR A 392 91.845 61.010 221.505 1.00 0.86
N
ATOM 2518 CA THR A 392 92.853 59.966 221.543 1.00 0.86
C
ATOM 2519 C THR A 392 93.011 59.354 222.918 1.00 0.86
C
ATOM 2520 O THR A 392 93.085 58.134 223.076 1.00 0.86
O
ATOM 2521 CB THR A 392 94.239 60.439 221.102 1.00 0.86
C
ATOM 2522 OG1 THR A 392 94.178 61.495 220.155 1.00 0.86
O
ATOM 2523 CG2 THR A 392 95.018 59.283 220.471 1.00 0.86
C
ATOM 2524 N SER A 393 93.055 60.196 223.967 1.00 0.86
N
ATOM 2525 CA SER A 393 93.179 59.750 225.341 1.00 0.86
C
ATOM 2526 C SER A 393 91.960 59.008 225.839 1.00 0.86
C
ATOM 2527 O SER A 393 92.108 57.961 226.463 1.00 0.86
O
ATOM 2528 CB SER A 393 93.577 60.896 226.299 1.00 0.86
C
ATOM 2529 OG SER A 393 92.618 61.943 226.248 1.00 0.86
O
ATOM 2530 N ASP A 394 90.730 59.469 225.521 1.00 0.88
N
ATOM 2531 CA ASP A 394 89.506 58.741 225.806 1.00 0.88
C

```
ATOM   2532  C   ASP A 394      89.439  57.396 225.097  1.00  0.88
C
ATOM   2533  O   ASP A 394      89.071  56.382 225.686  1.00  0.88
O
ATOM   2534  CB  ASP A 394      88.254  59.580 225.451  1.00  0.88
C
ATOM   2535  CG  ASP A 394      87.509  60.022 226.702  1.00  0.88
C
ATOM   2536  OD1 ASP A 394      87.285  59.157 227.589  1.00  0.88
O
ATOM   2537  OD2 ASP A 394      87.102  61.209 226.745  1.00  0.88
O
ATOM   2538  N   MET A 395      89.849  57.326 223.814  1.00  0.86
N
ATOM   2539  CA  MET A 395      89.930  56.066 223.094  1.00  0.86
C
ATOM   2540  C   MET A 395      90.922  55.074 223.705  1.00  0.86
C
ATOM   2541  O   MET A 395      90.645  53.879 223.818  1.00  0.86
O
ATOM   2542  CB  MET A 395      90.250  56.288 221.596  1.00  0.86
C
ATOM   2543  CG  MET A 395      89.080  56.870 220.774  1.00  0.86
C
ATOM   2544  SD  MET A 395      87.578  55.839 220.727  1.00  0.86
S
ATOM   2545  CE  MET A 395      88.247  54.474 219.737  1.00  0.86
C
ATOM   2546  N   ALA A 396      92.103  55.549 224.150  1.00  0.91
N
ATOM   2547  CA  ALA A 396      93.044  54.745 224.902  1.00  0.91
C
ATOM   2548  C   ALA A 396      92.541  54.321 226.273  1.00  0.91
C
ATOM   2549  O   ALA A 396      92.667  53.160 226.664  1.00  0.91
O
ATOM   2550  CB  ALA A 396      94.376  55.509 225.029  1.00  0.91
C
ATOM   2551  N   ALA A 397      91.930  55.260 227.015  1.00  0.92
N
ATOM   2552  CA  ALA A 397      91.348  55.033 228.311  1.00  0.92
C
ATOM   2553  C   ALA A 397      90.173  54.073 228.289  1.00  0.92
C
ATOM   2554  O   ALA A 397      90.060  53.201 229.136  1.00  0.92
O
ATOM   2555  CB  ALA A 397      90.967  56.383 228.948  1.00  0.92
C
ATOM   2556  N   ILE A 398      89.249  54.155 227.314  1.00  0.88
N
ATOM   2557  CA  ILE A 398      88.151  53.204 227.229  1.00  0.88
C
ATOM   2558  C   ILE A 398      88.619  51.789 226.941  1.00  0.88
C
```

```
ATOM   2559  O   ILE A 398      88.162  50.831 227.559  1.00  0.88
O
ATOM   2560  CB  ILE A 398      87.001  53.653 226.327  1.00  0.88
C
ATOM   2561  CG1 ILE A 398      85.717  52.879 226.704  1.00  0.88
C
ATOM   2562  CG2 ILE A 398      87.329  53.498 224.828  1.00  0.88
C
ATOM   2563  CD1 ILE A 398      84.431  53.484 226.136  1.00  0.88
C
ATOM   2564  N   ARG A 399      89.603  51.633 226.034  1.00  0.81
N
ATOM   2565  CA  ARG A 399      90.160  50.344 225.696  1.00  0.81
C
ATOM   2566  C   ARG A 399      90.866  49.628 226.838  1.00  0.81
C
ATOM   2567  O   ARG A 399      90.599  48.457 227.095  1.00  0.81
O
ATOM   2568  CB  ARG A 399      91.118  50.504 224.498  1.00  0.81
C
ATOM   2569  CG  ARG A 399      91.592  49.160 223.929  1.00  0.81
C
ATOM   2570  CD  ARG A 399      92.402  49.292 222.644  1.00  0.81
C
ATOM   2571  NE  ARG A 399      92.759  47.903 222.279  1.00  0.81
N
ATOM   2572  CZ  ARG A 399      93.521  47.511 221.260  1.00  0.81
C
ATOM   2573  NH1 ARG A 399      93.761  46.210 221.139  1.00  0.81
N
ATOM   2574  NH2 ARG A 399      94.033  48.385 220.399  1.00  0.81
N
ATOM   2575  N   HIS A 400      91.747  50.307 227.607  1.00  0.85
N
ATOM   2576  CA  HIS A 400      92.453  49.659 228.706  1.00  0.85
C
ATOM   2577  C   HIS A 400      91.535  49.247 229.848  1.00  0.85
C
ATOM   2578  O   HIS A 400      91.724  48.225 230.496  1.00  0.85
O
ATOM   2579  CB  HIS A 400      93.664  50.485 229.207  1.00  0.85
C
ATOM   2580  CG  HIS A 400      93.460  51.345 230.404  1.00  0.85
C
ATOM   2581  ND1 HIS A 400      92.502  52.330 230.404  1.00  0.85
N
ATOM   2582  CD2 HIS A 400      94.036  51.254 231.621  1.00  0.85
C
ATOM   2583  CE1 HIS A 400      92.503  52.817 231.619  1.00  0.85
C
ATOM   2584  NE2 HIS A 400      93.418  52.203 232.407  1.00  0.85
N
ATOM   2585  N   ARG A 401      90.469  50.023 230.110  1.00  0.81
N
```

ATOM 2586 CA ARG A 401 89.451 49.690 231.086 1.00 0.81
C
ATOM 2587 C ARG A 401 88.731 48.385 230.745 1.00 0.81
C
ATOM 2588 O ARG A 401 88.406 47.576 231.609 1.00 0.81
O
ATOM 2589 CB ARG A 401 88.445 50.862 231.211 1.00 0.81
C
ATOM 2590 CG ARG A 401 89.082 52.141 231.793 1.00 0.81
C
ATOM 2591 CD ARG A 401 88.212 53.396 231.669 1.00 0.81
C
ATOM 2592 NE ARG A 401 89.011 54.540 232.215 1.00 0.81
N
ATOM 2593 CZ ARG A 401 88.500 55.748 232.466 1.00 0.81
C
ATOM 2594 NH1 ARG A 401 87.253 56.071 232.160 1.00 0.81
N
ATOM 2595 NH2 ARG A 401 89.255 56.706 233.000 1.00 0.81
N
ATOM 2596 N LEU A 402 88.469 48.157 229.449 1.00 0.85
N
ATOM 2597 CA LEU A 402 87.829 46.961 228.943 1.00 0.85
C
ATOM 2598 C LEU A 402 88.791 45.771 228.865 1.00 0.85
C
ATOM 2599 O LEU A 402 88.466 44.685 229.335 1.00 0.85
O
ATOM 2600 CB LEU A 402 87.148 47.287 227.581 1.00 0.85
C
ATOM 2601 CG LEU A 402 86.001 48.332 227.698 1.00 0.85
C
ATOM 2602 CD1 LEU A 402 85.659 49.040 226.374 1.00 0.85
C
ATOM 2603 CD2 LEU A 402 84.734 47.728 228.329 1.00 0.85
C
ATOM 2604 N GLU A 403 90.014 45.949 228.314 1.00 0.81
N
ATOM 2605 CA GLU A 403 90.985 44.872 228.143 1.00 0.81
C
ATOM 2606 C GLU A 403 91.867 44.568 229.366 1.00 0.81
C
ATOM 2607 O GLU A 403 92.082 43.407 229.705 1.00 0.81
O
ATOM 2608 CB GLU A 403 91.879 45.144 226.896 1.00 0.81
C
ATOM 2609 CG GLU A 403 91.093 45.162 225.552 1.00 0.81
C
ATOM 2610 CD GLU A 403 91.879 45.673 224.347 1.00 0.81
C
ATOM 2611 OE1 GLU A 403 91.252 45.883 223.276 1.00 0.81
O
ATOM 2612 OE2 GLU A 403 93.109 45.910 224.414 1.00 0.81
O

ATOM 2613 N GLU A 404 92.416 45.590 230.062 1.00 0.80
N
ATOM 2614 CA GLU A 404 93.345 45.415 231.177 1.00 0.80
C
ATOM 2615 C GLU A 404 92.642 45.345 232.528 1.00 0.80
C
ATOM 2616 O GLU A 404 92.853 44.424 233.315 1.00 0.80
O
ATOM 2617 CB GLU A 404 94.388 46.568 231.264 1.00 0.80
C
ATOM 2618 CG GLU A 404 95.179 46.842 229.962 1.00 0.80
C
ATOM 2619 CD GLU A 404 96.168 47.995 230.084 1.00 0.80
C
ATOM 2620 OE1 GLU A 404 96.570 48.395 231.208 1.00 0.80
O
ATOM 2621 OE2 GLU A 404 96.521 48.569 229.014 1.00 0.80
O
ATOM 2622 N GLU A 405 91.757 46.321 232.836 1.00 0.82
N
ATOM 2623 CA GLU A 405 91.046 46.363 234.110 1.00 0.82
C
ATOM 2624 C GLU A 405 89.912 45.336 234.157 1.00 0.82
C
ATOM 2625 O GLU A 405 89.489 44.888 235.224 1.00 0.82
O
ATOM 2626 CB GLU A 405 90.457 47.775 234.386 1.00 0.82
C
ATOM 2627 CG GLU A 405 91.486 48.939 234.485 1.00 0.82
C
ATOM 2628 CD GLU A 405 92.281 49.023 235.792 1.00 0.82
C
ATOM 2629 OE1 GLU A 405 92.088 48.202 236.714 1.00 0.82
O
ATOM 2630 OE2 GLU A 405 93.089 49.995 235.897 1.00 0.82
O
ATOM 2631 N LEU A 406 89.414 44.920 232.971 1.00 0.84
N
ATOM 2632 CA LEU A 406 88.332 43.967 232.774 1.00 0.84
C
ATOM 2633 C LEU A 406 87.032 44.420 233.419 1.00 0.84
C
ATOM 2634 O LEU A 406 86.383 43.689 234.162 1.00 0.84
O
ATOM 2635 CB LEU A 406 88.681 42.517 233.202 1.00 0.84
C
ATOM 2636 CG LEU A 406 89.937 41.918 232.539 1.00 0.84
C
ATOM 2637 CD1 LEU A 406 90.374 40.640 233.272 1.00 0.84
C
ATOM 2638 CD2 LEU A 406 89.714 41.608 231.052 1.00 0.84
C
ATOM 2639 N CYS A 407 86.644 45.680 233.168 1.00 0.88
N

ATOM	2640	CA	CYS	A	407	85.437	46.259	233.724	1.00	0.88	C
ATOM	2641	C	CYS	A	407	84.140	45.763	233.109	1.00	0.88	C
ATOM	2642	O	CYS	A	407	83.898	45.969	231.928	1.00	0.88	O
ATOM	2643	CB	CYS	A	407	85.440	47.796	233.557	1.00	0.88	C
ATOM	2644	SG	CYS	A	407	86.853	48.558	234.416	1.00	0.88	S
ATOM	2645	N	ASP	A	408	83.235	45.183	233.927	1.00	0.83	N
ATOM	2646	CA	ASP	A	408	81.852	44.932	233.567	1.00	0.83	C
ATOM	2647	C	ASP	A	408	81.055	46.212	233.316	1.00	0.83	C
ATOM	2648	O	ASP	A	408	80.090	46.244	232.548	1.00	0.83	O
ATOM	2649	CB	ASP	A	408	81.117	44.162	234.690	1.00	0.83	C
ATOM	2650	CG	ASP	A	408	81.809	42.858	235.025	1.00	0.83	C
ATOM	2651	OD1	ASP	A	408	82.756	42.901	235.852	1.00	0.83	O
ATOM	2652	OD2	ASP	A	408	81.318	41.794	234.555	1.00	0.83	O
ATOM	2653	N	TRP	A	409	81.428	47.304	234.013	1.00	0.84	N
ATOM	2654	CA	TRP	A	409	80.745	48.572	233.910	1.00	0.84	C
ATOM	2655	C	TRP	A	409	81.724	49.724	233.901	1.00	0.84	C
ATOM	2656	O	TRP	A	409	82.657	49.786	234.703	1.00	0.84	O
ATOM	2657	CB	TRP	A	409	79.792	48.787	235.111	1.00	0.84	C
ATOM	2658	CG	TRP	A	409	78.632	47.806	235.145	1.00	0.84	C
ATOM	2659	CD1	TRP	A	409	77.487	47.843	234.443	1.00	0.84	C
ATOM	2660	CD2	TRP	A	409	78.509	46.689	235.986	1.00	0.84	C
ATOM	2661	NE1	TRP	A	409	76.705	46.757	234.686	1.00	0.84	N
ATOM	2662	CE2	TRP	A	409	77.275	46.048	235.661	1.00	0.84	C
ATOM	2663	CE3	TRP	A	409	79.292	46.234	236.976	1.00	0.84	C
ATOM	2664	CZ2	TRP	A	409	76.841	44.937	236.335	1.00	0.84	C
ATOM	2665	CZ3	TRP	A	409	78.765	45.226	237.727	1.00	0.84	C
ATOM	2666	CH2	TRP	A	409	77.612	44.514	237.398	1.00	0.84	C

ATOM 2667 N ILE A 410 81.516 50.704 233.000 1.00 0.90
N
ATOM 2668 CA ILE A 410 82.278 51.943 233.017 1.00 0.90
C
ATOM 2669 C ILE A 410 81.321 53.118 233.060 1.00 0.90
C
ATOM 2670 O ILE A 410 80.486 53.312 232.177 1.00 0.90
O
ATOM 2671 CB ILE A 410 83.237 52.107 231.841 1.00 0.90
C
ATOM 2672 CG1 ILE A 410 84.052 50.817 231.609 1.00 0.90
C
ATOM 2673 CG2 ILE A 410 84.182 53.301 232.119 1.00 0.90
C
ATOM 2674 CD1 ILE A 410 84.895 50.855 230.333 1.00 0.90
C
ATOM 2675 N ILE A 411 81.430 53.943 234.113 1.00 0.92
N
ATOM 2676 CA ILE A 411 80.564 55.077 234.359 1.00 0.92
C
ATOM 2677 C ILE A 411 81.380 56.347 234.225 1.00 0.92
C
ATOM 2678 O ILE A 411 82.354 56.565 234.943 1.00 0.92
O
ATOM 2679 CB ILE A 411 79.946 55.006 235.756 1.00 0.92
C
ATOM 2680 CG1 ILE A 411 79.045 53.753 235.861 1.00 0.92
C
ATOM 2681 CG2 ILE A 411 79.138 56.285 236.058 1.00 0.92
C
ATOM 2682 CD1 ILE A 411 78.492 53.501 237.267 1.00 0.92
C
ATOM 2683 N TYR A 412 80.992 57.230 233.291 1.00 0.89
N
ATOM 2684 CA TYR A 412 81.648 58.505 233.066 1.00 0.89
C
ATOM 2685 C TYR A 412 80.803 59.623 233.644 1.00 0.89
C
ATOM 2686 O TYR A 412 79.722 59.907 233.145 1.00 0.89
O
ATOM 2687 CB TYR A 412 81.827 58.784 231.550 1.00 0.89
C
ATOM 2688 CG TYR A 412 82.890 57.905 230.960 1.00 0.89
C
ATOM 2689 CD1 TYR A 412 82.606 56.589 230.559 1.00 0.89
C
ATOM 2690 CD2 TYR A 412 84.187 58.406 230.777 1.00 0.89
C
ATOM 2691 CE1 TYR A 412 83.609 55.784 229.998 1.00 0.89
C
ATOM 2692 CE2 TYR A 412 85.182 57.613 230.194 1.00 0.89
C
ATOM 2693 CZ TYR A 412 84.902 56.295 229.832 1.00 0.89
C

```
ATOM   2694  OH  TYR A 412      85.949  55.505 229.317  1.00  0.89
O
ATOM   2695  N   VAL A 413      81.258  60.296 234.716  1.00  0.91
N
ATOM   2696  CA  VAL A 413      80.521  61.384 235.341  1.00  0.91
C
ATOM   2697  C   VAL A 413      81.084  62.704 234.840  1.00  0.91
C
ATOM   2698  O   VAL A 413      82.099  63.216 235.312  1.00  0.91
O
ATOM   2699  CB  VAL A 413      80.584  61.323 236.864  1.00  0.91
C
ATOM   2700  CG1 VAL A 413      79.603  62.332 237.495  1.00  0.91
C
ATOM   2701  CG2 VAL A 413      80.254  59.894 237.341  1.00  0.91
C
ATOM   2702  N   VAL A 414      80.441  63.304 233.826  1.00  0.89
N
ATOM   2703  CA  VAL A 414      81.003  64.432 233.101  1.00  0.89
C
ATOM   2704  C   VAL A 414      79.840  65.306 232.682  1.00  0.89
C
ATOM   2705  O   VAL A 414      78.718  64.820 232.568  1.00  0.89
O
ATOM   2706  CB  VAL A 414      81.803  64.025 231.858  1.00  0.89
C
ATOM   2707  CG1 VAL A 414      82.597  65.231 231.329  1.00  0.89
C
ATOM   2708  CG2 VAL A 414      82.789  62.880 232.166  1.00  0.89
C
ATOM   2709  N   ASP A 415      80.038  66.620 232.469  1.00  0.86
N
ATOM   2710  CA  ASP A 415      78.993  67.551 232.118  1.00  0.86
C
ATOM   2711  C   ASP A 415      78.229  67.220 230.832  1.00  0.86
C
ATOM   2712  O   ASP A 415      78.695  66.505 229.944  1.00  0.86
O
ATOM   2713  CB  ASP A 415      79.538  68.997 232.128  1.00  0.86
C
ATOM   2714  CG  ASP A 415      80.464  69.209 230.947  1.00  0.86
C
ATOM   2715  OD1 ASP A 415      79.958  69.732 229.925  1.00  0.86
O
ATOM   2716  OD2 ASP A 415      81.652  68.806 231.037  1.00  0.86
O
ATOM   2717  N   SER A 416      76.995  67.741 230.709  1.00  0.84
N
ATOM   2718  CA  SER A 416      76.154  67.486 229.552  1.00  0.84
C
ATOM   2719  C   SER A 416      76.593  68.174 228.274  1.00  0.84
C
ATOM   2720  O   SER A 416      76.070  67.890 227.199  1.00  0.84
O
```

ATOM 2721 CB SER A 416 74.658 67.795 229.812 1.00 0.84
C
ATOM 2722 OG SER A 416 74.441 69.169 230.135 1.00 0.84
O
ATOM 2723 N GLY A 417 77.616 69.057 228.317 1.00 0.86
N
ATOM 2724 CA GLY A 417 78.173 69.622 227.102 1.00 0.86
C
ATOM 2725 C GLY A 417 79.068 68.644 226.386 1.00 0.86
C
ATOM 2726 O GLY A 417 79.451 68.860 225.239 1.00 0.86
O
ATOM 2727 N GLN A 418 79.390 67.505 227.035 1.00 0.79
N
ATOM 2728 CA GLN A 418 80.261 66.493 226.486 1.00 0.79
C
ATOM 2729 C GLN A 418 79.509 65.229 226.133 1.00 0.79
C
ATOM 2730 O GLN A 418 80.111 64.212 225.794 1.00 0.79
O
ATOM 2731 CB GLN A 418 81.412 66.158 227.457 1.00 0.79
C
ATOM 2732 CG GLN A 418 82.207 67.422 227.848 1.00 0.79
C
ATOM 2733 CD GLN A 418 83.512 67.080 228.558 1.00 0.79
C
ATOM 2734 OE1 GLN A 418 84.241 66.170 228.164 1.00 0.79
O
ATOM 2735 NE2 GLN A 418 83.838 67.836 229.629 1.00 0.79
N
ATOM 2736 N SER A 419 78.158 65.267 226.121 1.00 0.83
N
ATOM 2737 CA SER A 419 77.322 64.136 225.718 1.00 0.83
C
ATOM 2738 C SER A 419 77.627 63.666 224.315 1.00 0.83
C
ATOM 2739 O SER A 419 77.669 62.480 224.005 1.00 0.83
O
ATOM 2740 CB SER A 419 75.806 64.463 225.757 1.00 0.83
C
ATOM 2741 OG SER A 419 75.423 64.911 227.060 1.00 0.83
O
ATOM 2742 N THR A 420 77.886 64.627 223.415 1.00 0.82
N
ATOM 2743 CA THR A 420 78.240 64.409 222.027 1.00 0.82
C
ATOM 2744 C THR A 420 79.532 63.629 221.887 1.00 0.82
C
ATOM 2745 O THR A 420 79.584 62.609 221.210 1.00 0.82
O
ATOM 2746 CB THR A 420 78.343 65.748 221.298 1.00 0.82
C
ATOM 2747 OG1 THR A 420 79.305 66.611 221.887 1.00 0.82
O

```
ATOM   2748  CG2 THR A 420      77.003  66.485 221.445  1.00  0.82
C
ATOM   2749  N   HIS A 421      80.570  64.055 222.630  1.00  0.83
N
ATOM   2750  CA  HIS A 421      81.868  63.421 222.728  1.00  0.83
C
ATOM   2751  C   HIS A 421      81.816  61.989 223.225  1.00  0.83
C
ATOM   2752  O   HIS A 421      82.278  61.074 222.549  1.00  0.83
O
ATOM   2753  CB  HIS A 421      82.738  64.252 223.702  1.00  0.83
C
ATOM   2754  CG  HIS A 421      84.091  63.697 223.981  1.00  0.83
C
ATOM   2755  ND1 HIS A 421      85.095  63.919 223.075  1.00  0.83
N
ATOM   2756  CD2 HIS A 421      84.539  62.954 225.021  1.00  0.83
C
ATOM   2757  CE1 HIS A 421      86.153  63.305 223.578  1.00  0.83
C
ATOM   2758  NE2 HIS A 421      85.865  62.701 224.760  1.00  0.83
N
ATOM   2759  N   PHE A 422      81.178  61.735 224.387  1.00  0.86
N
ATOM   2760  CA  PHE A 422      81.075  60.390 224.931  1.00  0.86
C
ATOM   2761  C   PHE A 422      80.275  59.457 224.058  1.00  0.86
C
ATOM   2762  O   PHE A 422      80.683  58.321 223.831  1.00  0.86
O
ATOM   2763  CB  PHE A 422      80.522  60.376 226.371  1.00  0.86
C
ATOM   2764  CG  PHE A 422      81.603  60.874 227.280  1.00  0.86
C
ATOM   2765  CD1 PHE A 422      82.766  60.108 227.478  1.00  0.86
C
ATOM   2766  CD2 PHE A 422      81.522  62.151 227.849  1.00  0.86
C
ATOM   2767  CE1 PHE A 422      83.857  60.641 228.174  1.00  0.86
C
ATOM   2768  CE2 PHE A 422      82.616  62.689 228.535  1.00  0.86
C
ATOM   2769  CZ  PHE A 422      83.787  61.938 228.690  1.00  0.86
C
ATOM   2770  N   ASN A 423      79.158  59.930 223.463  1.00  0.83
N
ATOM   2771  CA  ASN A 423      78.446  59.145 222.474  1.00  0.83
C
ATOM   2772  C   ASN A 423      79.333  58.747 221.296  1.00  0.83
C
ATOM   2773  O   ASN A 423      79.396  57.579 220.941  1.00  0.83
O
ATOM   2774  CB  ASN A 423      77.219  59.909 221.919  1.00  0.83
C
```

```
ATOM   2775  CG  ASN A 423      76.088  59.948 222.942  1.00  0.83
C
ATOM   2776  OD1 ASN A 423      75.866  59.020 223.713  1.00  0.83
O
ATOM   2777  ND2 ASN A 423      75.281  61.034 222.892  1.00  0.83
N
ATOM   2778  N   THR A 424      80.103  59.681 220.705  1.00  0.86
N
ATOM   2779  CA  THR A 424      81.007  59.382 219.595  1.00  0.86
C
ATOM   2780  C   THR A 424      82.109  58.399 219.967  1.00  0.86
C
ATOM   2781  O   THR A 424      82.407  57.471 219.218  1.00  0.86
O
ATOM   2782  CB  THR A 424      81.645  60.630 219.002  1.00  0.86
C
ATOM   2783  OG1 THR A 424      80.648  61.597 218.717  1.00  0.86
O
ATOM   2784  CG2 THR A 424      82.297  60.339 217.647  1.00  0.86
C
ATOM   2785  N   ILE A 425      82.716  58.547 221.165  1.00  0.88
N
ATOM   2786  CA  ILE A 425      83.720  57.633 221.709  1.00  0.88
C
ATOM   2787  C   ILE A 425      83.185  56.221 221.915  1.00  0.88
C
ATOM   2788  O   ILE A 425      83.826  55.237 221.544  1.00  0.88
O
ATOM   2789  CB  ILE A 425      84.294  58.169 223.025  1.00  0.88
C
ATOM   2790  CG1 ILE A 425      85.110  59.469 222.810  1.00  0.88
C
ATOM   2791  CG2 ILE A 425      85.147  57.113 223.771  1.00  0.88
C
ATOM   2792  CD1 ILE A 425      86.438  59.291 222.068  1.00  0.88
C
ATOM   2793  N   PHE A 426      81.972  56.072 222.486  1.00  0.85
N
ATOM   2794  CA  PHE A 426      81.342  54.776 222.693  1.00  0.85
C
ATOM   2795  C   PHE A 426      81.001  54.086 221.376  1.00  0.85
C
ATOM   2796  O   PHE A 426      81.249  52.895 221.205  1.00  0.85
O
ATOM   2797  CB  PHE A 426      80.077  54.882 223.593  1.00  0.85
C
ATOM   2798  CG  PHE A 426      80.313  55.509 224.956  1.00  0.85
C
ATOM   2799  CD1 PHE A 426      81.586  55.683 225.540  1.00  0.85
C
ATOM   2800  CD2 PHE A 426      79.193  55.963 225.676  1.00  0.85
C
ATOM   2801  CE1 PHE A 426      81.733  56.316 226.782  1.00  0.85
C
```

ATOM 2802 CE2 PHE A 426 79.334 56.587 226.922 1.00 0.85
C
ATOM 2803 CZ PHE A 426 80.605 56.767 227.476 1.00 0.85
C
ATOM 2804 N LYS A 427 80.478 54.849 220.390 1.00 0.80
N
ATOM 2805 CA LYS A 427 80.240 54.368 219.037 1.00 0.80
C
ATOM 2806 C LYS A 427 81.508 53.925 218.338 1.00 0.80
C
ATOM 2807 O LYS A 427 81.576 52.860 217.735 1.00 0.80
O
ATOM 2808 CB LYS A 427 79.542 55.451 218.177 1.00 0.80
C
ATOM 2809 CG LYS A 427 78.129 55.831 218.655 1.00 0.80
C
ATOM 2810 CD LYS A 427 77.194 54.622 218.730 1.00 0.80
C
ATOM 2811 CE LYS A 427 75.742 54.950 219.025 1.00 0.80
C
ATOM 2812 NZ LYS A 427 74.987 53.682 218.973 1.00 0.80
N
ATOM 2813 N ALA A 428 82.594 54.705 218.470 1.00 0.89
N
ATOM 2814 CA ALA A 428 83.887 54.348 217.938 1.00 0.89
C
ATOM 2815 C ALA A 428 84.453 53.061 218.523 1.00 0.89
C
ATOM 2816 O ALA A 428 84.993 52.227 217.799 1.00 0.89
O
ATOM 2817 CB ALA A 428 84.859 55.514 218.164 1.00 0.89
C
ATOM 2818 N ALA A 429 84.306 52.865 219.847 1.00 0.90
N
ATOM 2819 CA ALA A 429 84.665 51.644 220.533 1.00 0.90
C
ATOM 2820 C ALA A 429 83.852 50.411 220.125 1.00 0.90
C
ATOM 2821 O ALA A 429 84.408 49.328 219.942 1.00 0.90
O
ATOM 2822 CB ALA A 429 84.573 51.889 222.050 1.00 0.90
C
ATOM 2823 N GLU A 430 82.518 50.537 219.952 1.00 0.78
N
ATOM 2824 CA GLU A 430 81.676 49.472 219.419 1.00 0.78
C
ATOM 2825 C GLU A 430 82.033 49.131 217.975 1.00 0.78
C
ATOM 2826 O GLU A 430 82.222 47.971 217.611 1.00 0.78
O
ATOM 2827 CB GLU A 430 80.177 49.851 219.551 1.00 0.78
C
ATOM 2828 CG GLU A 430 79.185 48.721 219.166 1.00 0.78
C

ATOM 2829 CD GLU A 430 77.719 49.033 219.493 1.00 0.78
C
ATOM 2830 OE1 GLU A 430 77.405 50.163 219.955 1.00 0.78
O
ATOM 2831 OE2 GLU A 430 76.889 48.103 219.314 1.00 0.78
O
ATOM 2832 N ARG A 431 82.249 50.165 217.133 1.00 0.74
N
ATOM 2833 CA ARG A 431 82.638 50.052 215.739 1.00 0.74
C
ATOM 2834 C ARG A 431 83.938 49.298 215.507 1.00 0.74
C
ATOM 2835 O ARG A 431 84.075 48.550 214.542 1.00 0.74
O
ATOM 2836 CB ARG A 431 82.750 51.468 215.120 1.00 0.74
C
ATOM 2837 CG ARG A 431 83.088 51.509 213.618 1.00 0.74
C
ATOM 2838 CD ARG A 431 83.173 52.939 213.077 1.00 0.74
C
ATOM 2839 NE ARG A 431 83.519 52.872 211.616 1.00 0.74
N
ATOM 2840 CZ ARG A 431 82.599 52.674 210.675 1.00 0.74
C
ATOM 2841 NH1 ARG A 431 82.890 52.759 209.375 1.00 0.74
N
ATOM 2842 NH2 ARG A 431 81.334 52.442 211.005 1.00 0.74
N
ATOM 2843 N SER A 432 84.940 49.456 216.393 1.00 0.82
N
ATOM 2844 CA SER A 432 86.207 48.755 216.253 1.00 0.82
C
ATOM 2845 C SER A 432 86.218 47.435 217.007 1.00 0.82
C
ATOM 2846 O SER A 432 87.235 46.744 217.051 1.00 0.82
O
ATOM 2847 CB SER A 432 87.397 49.634 216.719 1.00 0.82
C
ATOM 2848 OG SER A 432 87.306 49.985 218.101 1.00 0.82
O
ATOM 2849 N ALA A 433 85.061 47.065 217.592 1.00 0.80
N
ATOM 2850 CA ALA A 433 84.779 45.783 218.196 1.00 0.80
C
ATOM 2851 C ALA A 433 85.378 45.582 219.590 1.00 0.80
C
ATOM 2852 O ALA A 433 85.525 44.452 220.050 1.00 0.80
O
ATOM 2853 CB ALA A 433 85.081 44.613 217.233 1.00 0.80
C
ATOM 2854 N ILE A 434 85.700 46.674 220.327 1.00 0.81
N
ATOM 2855 CA ILE A 434 86.261 46.622 221.682 1.00 0.81
C

ATOM 2856 C ILE A 434 85.272 46.058 222.691 1.00 0.81
C
ATOM 2857 O ILE A 434 85.626 45.337 223.621 1.00 0.81
O
ATOM 2858 CB ILE A 434 86.786 47.985 222.158 1.00 0.81
C
ATOM 2859 CG1 ILE A 434 87.920 48.449 221.214 1.00 0.81
C
ATOM 2860 CG2 ILE A 434 87.299 47.932 223.621 1.00 0.81
C
ATOM 2861 CD1 ILE A 434 88.375 49.898 221.416 1.00 0.81
C
ATOM 2862 N LEU A 435 83.975 46.366 222.525 1.00 0.79
N
ATOM 2863 CA LEU A 435 82.960 45.884 223.435 1.00 0.79
C
ATOM 2864 C LEU A 435 81.618 45.749 222.749 1.00 0.79
C
ATOM 2865 O LEU A 435 81.426 46.207 221.630 1.00 0.79
O
ATOM 2866 CB LEU A 435 82.808 46.764 224.707 1.00 0.79
C
ATOM 2867 CG LEU A 435 82.151 48.153 224.530 1.00 0.79
C
ATOM 2868 CD1 LEU A 435 81.820 48.749 225.901 1.00 0.79
C
ATOM 2869 CD2 LEU A 435 83.009 49.140 223.728 1.00 0.79
C
ATOM 2870 N ASN A 436 80.644 45.101 223.427 1.00 0.73
N
ATOM 2871 CA ASN A 436 79.259 45.035 223.002 1.00 0.73
C
ATOM 2872 C ASN A 436 78.425 45.673 224.108 1.00 0.73
C
ATOM 2873 O ASN A 436 78.585 45.265 225.260 1.00 0.73
O
ATOM 2874 CB ASN A 436 78.835 43.550 222.808 1.00 0.73
C
ATOM 2875 CG ASN A 436 77.413 43.363 222.275 1.00 0.73
C
ATOM 2876 OD1 ASN A 436 76.443 43.921 222.777 1.00 0.73
O
ATOM 2877 ND2 ASN A 436 77.266 42.495 221.251 1.00 0.73
N
ATOM 2878 N PRO A 437 77.533 46.630 223.855 1.00 0.73
N
ATOM 2879 CA PRO A 437 76.812 47.333 224.911 1.00 0.73
C
ATOM 2880 C PRO A 437 75.781 46.468 225.607 1.00 0.73
C
ATOM 2881 O PRO A 437 75.258 46.884 226.638 1.00 0.73
O
ATOM 2882 CB PRO A 437 76.161 48.529 224.191 1.00 0.73
C

ATOM 2883 CG PRO A 437 76.094 48.116 222.720 1.00 0.73
C
ATOM 2884 CD PRO A 437 77.352 47.276 222.553 1.00 0.73
C
ATOM 2885 N LEU A 438 75.472 45.265 225.087 1.00 0.70
N
ATOM 2886 CA LEU A 438 74.613 44.321 225.768 1.00 0.70
C
ATOM 2887 C LEU A 438 75.365 43.471 226.779 1.00 0.70
C
ATOM 2888 O LEU A 438 74.753 42.864 227.655 1.00 0.70
O
ATOM 2889 CB LEU A 438 73.933 43.354 224.772 1.00 0.70
C
ATOM 2890 CG LEU A 438 73.025 44.022 223.721 1.00 0.70
C
ATOM 2891 CD1 LEU A 438 72.597 42.981 222.676 1.00 0.70
C
ATOM 2892 CD2 LEU A 438 71.783 44.672 224.349 1.00 0.70
C
ATOM 2893 N SER A 439 76.713 43.400 226.701 1.00 0.72
N
ATOM 2894 CA SER A 439 77.486 42.599 227.637 1.00 0.72
C
ATOM 2895 C SER A 439 78.256 43.438 228.625 1.00 0.72
C
ATOM 2896 O SER A 439 78.469 43.020 229.760 1.00 0.72
O
ATOM 2897 CB SER A 439 78.490 41.649 226.929 1.00 0.72
C
ATOM 2898 OG SER A 439 79.441 42.337 226.109 1.00 0.72
O
ATOM 2899 N HIS A 440 78.644 44.660 228.232 1.00 0.76
N
ATOM 2900 CA HIS A 440 79.345 45.576 229.104 1.00 0.76
C
ATOM 2901 C HIS A 440 78.585 46.890 229.118 1.00 0.76
C
ATOM 2902 O HIS A 440 78.386 47.500 228.069 1.00 0.76
O
ATOM 2903 CB HIS A 440 80.773 45.834 228.574 1.00 0.76
C
ATOM 2904 CG HIS A 440 81.628 44.603 228.514 1.00 0.76
C
ATOM 2905 ND1 HIS A 440 82.223 44.146 229.667 1.00 0.76
N
ATOM 2906 CD2 HIS A 440 82.017 43.842 227.456 1.00 0.76
C
ATOM 2907 CE1 HIS A 440 82.966 43.126 229.302 1.00 0.76
C
ATOM 2908 NE2 HIS A 440 82.879 42.894 227.968 1.00 0.76
N
ATOM 2909 N ARG A 441 78.130 47.404 230.286 1.00 0.74
N

```
ATOM   2910  CA  ARG A 441      77.472  48.706 230.262  1.00  0.74
C
ATOM   2911  C   ARG A 441      78.506  49.822 230.295  1.00  0.74
C
ATOM   2912  O   ARG A 441      79.346  49.913 231.191  1.00  0.74
O
ATOM   2913  CB  ARG A 441      76.520  49.014 231.440  1.00  0.74
C
ATOM   2914  CG  ARG A 441      75.231  48.179 231.579  1.00  0.74
C
ATOM   2915  CD  ARG A 441      74.395  48.410 232.861  1.00  0.74
C
ATOM   2916  NE  ARG A 441      73.919  49.821 232.917  1.00  0.74
N
ATOM   2917  CZ  ARG A 441      73.375  50.414 233.991  1.00  0.74
C
ATOM   2918  NH1 ARG A 441      73.447  49.876 235.201  1.00  0.74
N
ATOM   2919  NH2 ARG A 441      72.728  51.566 233.820  1.00  0.74
N
ATOM   2920  N   VAL A 442      78.416  50.735 229.323  1.00  0.88
N
ATOM   2921  CA  VAL A 442      79.206  51.948 229.300  1.00  0.88
C
ATOM   2922  C   VAL A 442      78.233  53.109 229.281  1.00  0.88
C
ATOM   2923  O   VAL A 442      77.361  53.200 228.424  1.00  0.88
O
ATOM   2924  CB  VAL A 442      80.180  51.989 228.134  1.00  0.88
C
ATOM   2925  CG1 VAL A 442      80.868  53.359 228.060  1.00  0.88
C
ATOM   2926  CG2 VAL A 442      81.235  50.888 228.343  1.00  0.88
C
ATOM   2927  N   ASP A 443      78.348  53.992 230.290  1.00  0.87
N
ATOM   2928  CA  ASP A 443      77.290  54.881 230.709  1.00  0.87
C
ATOM   2929  C   ASP A 443      77.869  56.290 230.884  1.00  0.87
C
ATOM   2930  O   ASP A 443      78.912  56.481 231.513  1.00  0.87
O
ATOM   2931  CB  ASP A 443      76.683  54.283 232.016  1.00  0.87
C
ATOM   2932  CG  ASP A 443      75.318  54.852 232.370  1.00  0.87
C
ATOM   2933  OD1 ASP A 443      75.093  56.061 232.113  1.00  0.87
O
ATOM   2934  OD2 ASP A 443      74.487  54.065 232.899  1.00  0.87
O
ATOM   2935  N   HIS A 444      77.229  57.323 230.286  1.00  0.84
N
ATOM   2936  CA  HIS A 444      77.630  58.711 230.473  1.00  0.84
C
```

ATOM 2937 C HIS A 444 76.631 59.362 231.399 1.00 0.84
C
ATOM 2938 O HIS A 444 75.514 59.731 231.038 1.00 0.84
O
ATOM 2939 CB HIS A 444 77.751 59.564 229.178 1.00 0.84
C
ATOM 2940 CG HIS A 444 78.028 61.038 229.416 1.00 0.84
C
ATOM 2941 ND1 HIS A 444 77.524 61.963 228.526 1.00 0.84
N
ATOM 2942 CD2 HIS A 444 78.639 61.680 230.450 1.00 0.84
C
ATOM 2943 CE1 HIS A 444 77.825 63.144 229.035 1.00 0.84
C
ATOM 2944 NE2 HIS A 444 78.498 63.030 230.202 1.00 0.84
N
ATOM 2945 N VAL A 445 77.054 59.564 232.651 1.00 0.88
N
ATOM 2946 CA VAL A 445 76.242 60.141 233.686 1.00 0.88
C
ATOM 2947 C VAL A 445 76.394 61.644 233.605 1.00 0.88
C
ATOM 2948 O VAL A 445 77.181 62.285 234.300 1.00 0.88
O
ATOM 2949 CB VAL A 445 76.613 59.578 235.043 1.00 0.88
C
ATOM 2950 CG1 VAL A 445 75.819 60.258 236.165 1.00 0.88
C
ATOM 2951 CG2 VAL A 445 76.327 58.069 235.040 1.00 0.88
C
ATOM 2952 N GLN A 446 75.623 62.242 232.683 1.00 0.84
N
ATOM 2953 CA GLN A 446 75.603 63.667 232.463 1.00 0.84
C
ATOM 2954 C GLN A 446 74.950 64.466 233.574 1.00 0.84
C
ATOM 2955 O GLN A 446 74.062 63.979 234.285 1.00 0.84
O
ATOM 2956 CB GLN A 446 74.999 64.048 231.090 1.00 0.84
C
ATOM 2957 CG GLN A 446 73.464 63.905 230.978 1.00 0.84
C
ATOM 2958 CD GLN A 446 72.973 64.324 229.588 1.00 0.84
C
ATOM 2959 OE1 GLN A 446 73.340 63.774 228.551 1.00 0.84
O
ATOM 2960 NE2 GLN A 446 72.106 65.363 229.547 1.00 0.84
N
ATOM 2961 N PHE A 447 75.368 65.735 233.715 1.00 0.86
N
ATOM 2962 CA PHE A 447 74.760 66.673 234.629 1.00 0.86
C
ATOM 2963 C PHE A 447 74.686 68.049 233.997 1.00 0.86
C

```
ATOM   2964  O   PHE A 447      75.545  68.424 233.200  1.00  0.86
O
ATOM   2965  CB  PHE A 447      75.463  66.734 236.017  1.00  0.86
C
ATOM   2966  CG  PHE A 447      76.944  66.972 235.925  1.00  0.86
C
ATOM   2967  CD1 PHE A 447      77.453  68.271 235.776  1.00  0.86
C
ATOM   2968  CD2 PHE A 447      77.843  65.897 236.004  1.00  0.86
C
ATOM   2969  CE1 PHE A 447      78.830  68.496 235.713  1.00  0.86
C
ATOM   2970  CE2 PHE A 447      79.223  66.118 235.938  1.00  0.86
C
ATOM   2971  CZ  PHE A 447      79.716  67.418 235.788  1.00  0.86
C
ATOM   2972  N   GLY A 448      73.619  68.814 234.314  1.00  0.89
N
ATOM   2973  CA  GLY A 448      73.437  70.178 233.826  1.00  0.89
C
ATOM   2974  C   GLY A 448      74.348  71.170 234.501  1.00  0.89
C
ATOM   2975  O   GLY A 448      75.074  70.850 235.448  1.00  0.89
O
ATOM   2976  N   VAL A 449      74.320  72.433 234.051  1.00  0.87
N
ATOM   2977  CA  VAL A 449      75.305  73.424 234.433  1.00  0.87
C
ATOM   2978  C   VAL A 449      75.014  74.016 235.799  1.00  0.87
C
ATOM   2979  O   VAL A 449      73.916  73.921 236.342  1.00  0.87
O
ATOM   2980  CB  VAL A 449      75.550  74.529 233.396  1.00  0.87
C
ATOM   2981  CG1 VAL A 449      75.517  73.934 231.976  1.00  0.87
C
ATOM   2982  CG2 VAL A 449      74.522  75.668 233.503  1.00  0.87
C
ATOM   2983  N   VAL A 450      76.028  74.649 236.421  1.00  0.86
N
ATOM   2984  CA  VAL A 450      75.824  75.332 237.685  1.00  0.86
C
ATOM   2985  C   VAL A 450      75.569  76.793 237.405  1.00  0.86
C
ATOM   2986  O   VAL A 450      76.332  77.464 236.704  1.00  0.86
O
ATOM   2987  CB  VAL A 450      76.972  75.183 238.668  1.00  0.86
C
ATOM   2988  CG1 VAL A 450      76.585  75.772 240.041  1.00  0.86
C
ATOM   2989  CG2 VAL A 450      77.277  73.691 238.856  1.00  0.86
C
ATOM   2990  N   LEU A 451      74.458  77.305 237.944  1.00  0.82
N
```

ATOM 2991 CA LEU A 451 73.982 78.640 237.712 1.00 0.82
C
ATOM 2992 C LEU A 451 73.927 79.383 239.027 1.00 0.82
C
ATOM 2993 O LEU A 451 73.834 78.783 240.100 1.00 0.82
O
ATOM 2994 CB LEU A 451 72.550 78.632 237.137 1.00 0.82
C
ATOM 2995 CG LEU A 451 72.343 77.884 235.807 1.00 0.82
C
ATOM 2996 CD1 LEU A 451 70.845 77.840 235.476 1.00 0.82
C
ATOM 2997 CD2 LEU A 451 73.095 78.528 234.635 1.00 0.82
C
ATOM 2998 N GLY A 452 73.997 80.727 238.977 1.00 0.86
N
ATOM 2999 CA GLY A 452 73.641 81.567 240.111 1.00 0.86
C
ATOM 3000 C GLY A 452 72.150 81.733 240.229 1.00 0.86
C
ATOM 3001 O GLY A 452 71.362 81.179 239.463 1.00 0.86
O
ATOM 3002 N GLU A 453 71.712 82.564 241.187 1.00 0.79
N
ATOM 3003 CA GLU A 453 70.315 82.888 241.404 1.00 0.79
C
ATOM 3004 C GLU A 453 69.717 83.769 240.324 1.00 0.79
C
ATOM 3005 O GLU A 453 68.502 83.922 240.225 1.00 0.79
O
ATOM 3006 CB GLU A 453 70.156 83.489 242.801 1.00 0.79
C
ATOM 3007 CG GLU A 453 70.166 82.361 243.853 1.00 0.79
C
ATOM 3008 CD GLU A 453 70.563 82.953 245.203 1.00 0.79
C
ATOM 3009 OE1 GLU A 453 71.745 83.415 245.330 1.00 0.79
O
ATOM 3010 OE2 GLU A 453 69.728 82.775 246.136 1.00 0.79
O
ATOM 3011 N ASP A 454 70.569 84.319 239.440 1.00 0.79
N
ATOM 3012 CA ASP A 454 70.201 85.055 238.261 1.00 0.79
C
ATOM 3013 C ASP A 454 69.914 84.154 237.059 1.00 0.79
C
ATOM 3014 O ASP A 454 69.403 84.603 236.034 1.00 0.79
O
ATOM 3015 CB ASP A 454 71.344 86.060 237.932 1.00 0.79
C
ATOM 3016 CG ASP A 454 72.692 85.405 237.648 1.00 0.79
C
ATOM 3017 OD1 ASP A 454 73.099 84.500 238.422 1.00 0.79
O

ATOM 3018 OD2 ASP A 454 73.319 85.809 236.637 1.00 0.79
O
ATOM 3019 N GLY A 455 70.233 82.843 237.156 1.00 0.85
N
ATOM 3020 CA GLY A 455 69.980 81.879 236.091 1.00 0.85
C
ATOM 3021 C GLY A 455 71.018 81.880 235.002 1.00 0.85
C
ATOM 3022 O GLY A 455 70.870 81.204 233.988 1.00 0.85
O
ATOM 3023 N LYS A 456 72.114 82.632 235.189 1.00 0.77
N
ATOM 3024 CA LYS A 456 73.274 82.604 234.327 1.00 0.77
C
ATOM 3025 C LYS A 456 74.382 81.793 234.968 1.00 0.77
C
ATOM 3026 O LYS A 456 74.307 81.405 236.130 1.00 0.77
O
ATOM 3027 CB LYS A 456 73.789 84.020 234.004 1.00 0.77
C
ATOM 3028 CG LYS A 456 72.674 84.956 233.514 1.00 0.77
C
ATOM 3029 CD LYS A 456 73.185 86.062 232.581 1.00 0.77
C
ATOM 3030 CE LYS A 456 74.219 86.981 233.234 1.00 0.77
C
ATOM 3031 NZ LYS A 456 74.677 87.975 232.238 1.00 0.77
N
ATOM 3032 N LYS A 457 75.442 81.462 234.193 1.00 0.73
N
ATOM 3033 CA LYS A 457 76.550 80.644 234.661 1.00 0.73
C
ATOM 3034 C LYS A 457 77.178 81.097 235.968 1.00 0.73
C
ATOM 3035 O LYS A 457 77.571 82.251 236.115 1.00 0.73
O
ATOM 3036 CB LYS A 457 77.694 80.570 233.615 1.00 0.73
C
ATOM 3037 CG LYS A 457 77.262 79.936 232.288 1.00 0.73
C
ATOM 3038 CD LYS A 457 78.434 79.406 231.441 1.00 0.73
C
ATOM 3039 CE LYS A 457 79.167 78.228 232.098 1.00 0.73
C
ATOM 3040 NZ LYS A 457 80.049 77.542 231.125 1.00 0.73
N
ATOM 3041 N PHE A 458 77.314 80.166 236.934 1.00 0.77
N
ATOM 3042 CA PHE A 458 77.910 80.435 238.225 1.00 0.77
C
ATOM 3043 C PHE A 458 79.379 80.838 238.108 1.00 0.77
C
ATOM 3044 O PHE A 458 80.257 80.036 237.782 1.00 0.77
O

ATOM 3045 CB PHE A 458 77.720 79.202 239.141 1.00 0.77
C
ATOM 3046 CG PHE A 458 78.071 79.461 240.576 1.00 0.77
C
ATOM 3047 CD1 PHE A 458 77.143 80.059 241.441 1.00 0.77
C
ATOM 3048 CD2 PHE A 458 79.319 79.070 241.083 1.00 0.77
C
ATOM 3049 CE1 PHE A 458 77.461 80.272 242.788 1.00 0.77
C
ATOM 3050 CE2 PHE A 458 79.635 79.270 242.432 1.00 0.77
C
ATOM 3051 CZ PHE A 458 78.709 79.880 243.283 1.00 0.77
C
ATOM 3052 N LYS A 459 79.651 82.129 238.352 1.00 0.69
N
ATOM 3053 CA LYS A 459 80.947 82.742 238.213 1.00 0.69
C
ATOM 3054 C LYS A 459 81.135 83.688 239.381 1.00 0.69
C
ATOM 3055 O LYS A 459 80.186 84.036 240.080 1.00 0.69
O
ATOM 3056 CB LYS A 459 81.069 83.581 236.910 1.00 0.69
C
ATOM 3057 CG LYS A 459 80.964 82.807 235.583 1.00 0.69
C
ATOM 3058 CD LYS A 459 81.977 81.658 235.473 1.00 0.69
C
ATOM 3059 CE LYS A 459 82.574 81.468 234.080 1.00 0.69
C
ATOM 3060 NZ LYS A 459 83.594 82.500 233.817 1.00 0.69
N
ATOM 3061 N THR A 460 82.376 84.146 239.628 1.00 0.68
N
ATOM 3062 CA THR A 460 82.617 85.208 240.594 1.00 0.68
C
ATOM 3063 C THR A 460 82.654 86.552 239.885 1.00 0.68
C
ATOM 3064 O THR A 460 83.174 86.694 238.781 1.00 0.68
O
ATOM 3065 CB THR A 460 83.850 85.003 241.463 1.00 0.68
C
ATOM 3066 OG1 THR A 460 83.906 85.970 242.507 1.00 0.68
O
ATOM 3067 CG2 THR A 460 85.143 85.086 240.643 1.00 0.68
C
ATOM 3068 N ARG A 461 82.040 87.588 240.488 1.00 0.58
N
ATOM 3069 CA ARG A 461 81.958 88.900 239.876 1.00 0.58
C
ATOM 3070 C ARG A 461 83.312 89.629 239.870 1.00 0.58
C
ATOM 3071 O ARG A 461 83.833 89.988 240.919 1.00 0.58
O

ATOM 3072 CB ARG A 461 80.842 89.796 240.481 1.00 0.58
C
ATOM 3073 CG ARG A 461 80.898 89.973 242.010 1.00 0.58
C
ATOM 3074 CD ARG A 461 79.788 90.885 242.538 1.00 0.58
C
ATOM 3075 NE ARG A 461 80.101 91.260 243.957 1.00 0.58
N
ATOM 3076 CZ ARG A 461 79.813 90.521 245.038 1.00 0.58
C
ATOM 3077 NH1 ARG A 461 79.164 89.366 244.944 1.00 0.58
N
ATOM 3078 NH2 ARG A 461 80.178 90.962 246.242 1.00 0.58
N
ATOM 3079 N SER A 462 83.959 89.884 238.715 1.00 0.65
N
ATOM 3080 CA SER A 462 83.540 89.554 237.369 1.00 0.65
C
ATOM 3081 C SER A 462 84.752 89.202 236.547 1.00 0.65
C
ATOM 3082 O SER A 462 85.763 89.882 236.650 1.00 0.65
O
ATOM 3083 CB SER A 462 82.853 90.730 236.650 1.00 0.65
C
ATOM 3084 OG SER A 462 81.679 91.132 237.356 1.00 0.65
O
ATOM 3085 N GLY A 463 84.728 88.156 235.694 1.00 0.64
N
ATOM 3086 CA GLY A 463 83.595 87.288 235.338 1.00 0.64
C
ATOM 3087 C GLY A 463 84.004 85.859 235.448 1.00 0.64
C
ATOM 3088 O GLY A 463 83.556 84.990 234.703 1.00 0.64
O
ATOM 3089 N ASP A 464 84.903 85.609 236.398 1.00 0.65
N
ATOM 3090 CA ASP A 464 85.836 84.518 236.404 1.00 0.65
C
ATOM 3091 C ASP A 464 85.386 83.287 237.168 1.00 0.65
C
ATOM 3092 O ASP A 464 84.344 83.231 237.809 1.00 0.65
O
ATOM 3093 CB ASP A 464 87.176 85.038 236.957 1.00 0.65
C
ATOM 3094 CG ASP A 464 87.777 86.065 236.007 1.00 0.65
C
ATOM 3095 OD1 ASP A 464 87.421 86.042 234.800 1.00 0.65
O
ATOM 3096 OD2 ASP A 464 88.612 86.868 236.483 1.00 0.65
O
ATOM 3097 N THR A 465 86.159 82.195 237.043 1.00 0.73
N
ATOM 3098 CA THR A 465 85.931 80.951 237.778 1.00 0.73
C

ATOM 3099 C THR A 465 86.088 81.133 239.275 1.00 0.73
C
ATOM 3100 O THR A 465 87.032 81.758 239.746 1.00 0.73
O
ATOM 3101 CB THR A 465 86.846 79.822 237.321 1.00 0.73
C
ATOM 3102 OG1 THR A 465 86.511 79.447 235.992 1.00 0.73
O
ATOM 3103 CG2 THR A 465 86.688 78.558 238.177 1.00 0.73
C
ATOM 3104 N VAL A 466 85.164 80.573 240.079 1.00 0.78
N
ATOM 3105 CA VAL A 466 85.221 80.666 241.525 1.00 0.78
C
ATOM 3106 C VAL A 466 86.144 79.594 242.085 1.00 0.78
C
ATOM 3107 O VAL A 466 86.158 78.454 241.619 1.00 0.78
O
ATOM 3108 CB VAL A 466 83.824 80.631 242.179 1.00 0.78
C
ATOM 3109 CG1 VAL A 466 82.721 80.756 241.106 1.00 0.78
C
ATOM 3110 CG2 VAL A 466 83.564 79.364 243.024 1.00 0.78
C
ATOM 3111 N LYS A 467 86.951 79.918 243.115 1.00 0.76
N
ATOM 3112 CA LYS A 467 87.757 78.917 243.782 1.00 0.76
C
ATOM 3113 C LYS A 467 86.892 78.036 244.659 1.00 0.76
C
ATOM 3114 O LYS A 467 86.132 78.508 245.500 1.00 0.76
O
ATOM 3115 CB LYS A 467 88.895 79.572 244.593 1.00 0.76
C
ATOM 3116 CG LYS A 467 89.911 78.627 245.262 1.00 0.76
C
ATOM 3117 CD LYS A 467 90.968 79.461 246.002 1.00 0.76
C
ATOM 3118 CE LYS A 467 92.078 78.682 246.698 1.00 0.76
C
ATOM 3119 NZ LYS A 467 92.863 79.654 247.485 1.00 0.76
N
ATOM 3120 N LEU A 468 86.958 76.702 244.469 1.00 0.85
N
ATOM 3121 CA LEU A 468 86.139 75.770 245.224 1.00 0.85
C
ATOM 3122 C LEU A 468 86.400 75.823 246.705 1.00 0.85
C
ATOM 3123 O LEU A 468 85.484 75.723 247.510 1.00 0.85
O
ATOM 3124 CB LEU A 468 86.298 74.315 244.749 1.00 0.85
C
ATOM 3125 CG LEU A 468 85.816 74.062 243.315 1.00 0.85
C

```
ATOM   3126  CD1 LEU A 468      85.905  72.564 243.024  1.00  0.85
C
ATOM   3127  CD2 LEU A 468      84.385  74.562 243.081  1.00  0.85
C
ATOM   3128  N   SER A 469      87.663  76.043 247.104  1.00  0.86
N
ATOM   3129  CA  SER A 469      88.003  76.295 248.491  1.00  0.86
C
ATOM   3130  C   SER A 469      87.270  77.482 249.087  1.00  0.86
C
ATOM   3131  O   SER A 469      86.673  77.332 250.138  1.00  0.86
O
ATOM   3132  CB  SER A 469      89.515  76.498 248.710  1.00  0.86
C
ATOM   3133  OG  SER A 469      90.250  75.377 248.210  1.00  0.86
O
ATOM   3134  N   ASP A 470      87.188  78.635 248.386  1.00  0.85
N
ATOM   3135  CA  ASP A 470      86.441  79.806 248.814  1.00  0.85
C
ATOM   3136  C   ASP A 470      84.954  79.501 248.983  1.00  0.85
C
ATOM   3137  O   ASP A 470      84.300  79.917 249.938  1.00  0.85
O
ATOM   3138  CB  ASP A 470      86.602  80.965 247.795  1.00  0.85
C
ATOM   3139  CG  ASP A 470      88.048  81.407 247.609  1.00  0.85
C
ATOM   3140  OD1 ASP A 470      88.970  80.905 248.304  1.00  0.85
O
ATOM   3141  OD2 ASP A 470      88.257  82.215 246.669  1.00  0.85
O
ATOM   3142  N   LEU A 471      84.391  78.701 248.054  1.00  0.88
N
ATOM   3143  CA  LEU A 471      83.033  78.200 248.129  1.00  0.88
C
ATOM   3144  C   LEU A 471      82.783  77.325 249.359  1.00  0.88
C
ATOM   3145  O   LEU A 471      81.791  77.479 250.070  1.00  0.88
O
ATOM   3146  CB  LEU A 471      82.727  77.442 246.811  1.00  0.88
C
ATOM   3147  CG  LEU A 471      81.247  77.147 246.516  1.00  0.88
C
ATOM   3148  CD1 LEU A 471      80.455  78.450 246.366  1.00  0.88
C
ATOM   3149  CD2 LEU A 471      81.103  76.304 245.239  1.00  0.88
C
ATOM   3150  N   LEU A 472      83.716  76.408 249.679  1.00  0.88
N
ATOM   3151  CA  LEU A 472      83.680  75.615 250.897  1.00  0.88
C
ATOM   3152  C   LEU A 472      83.841  76.457 252.161  1.00  0.88
C
```

```
ATOM   3153  O   LEU A 472      83.110  76.281 253.135  1.00  0.88
O
ATOM   3154  CB  LEU A 472      84.737  74.480 250.855  1.00  0.88
C
ATOM   3155  CG  LEU A 472      84.508  73.430 249.741  1.00  0.88
C
ATOM   3156  CD1 LEU A 472      85.606  72.358 249.751  1.00  0.88
C
ATOM   3157  CD2 LEU A 472      83.136  72.753 249.846  1.00  0.88
C
ATOM   3158  N   ASP A 473      84.779  77.418 252.151  1.00  0.85
N
ATOM   3159  CA  ASP A 473      85.087  78.316 253.242  1.00  0.85
C
ATOM   3160  C   ASP A 473      83.894  79.240 253.613  1.00  0.85
C
ATOM   3161  O   ASP A 473      83.570  79.405 254.792  1.00  0.85
O
ATOM   3162  CB  ASP A 473      86.429  79.057 252.917  1.00  0.85
C
ATOM   3163  CG  ASP A 473      87.646  78.142 252.748  1.00  0.85
C
ATOM   3164  OD1 ASP A 473      87.533  76.940 253.052  1.00  0.85
O
ATOM   3165  OD2 ASP A 473      88.731  78.611 252.318  1.00  0.85
O
ATOM   3166  N   GLU A 474      83.149  79.795 252.622  1.00  0.84
N
ATOM   3167  CA  GLU A 474      81.864  80.487 252.800  1.00  0.84
C
ATOM   3168  C   GLU A 474      80.777  79.588 253.384  1.00  0.84
C
ATOM   3169  O   GLU A 474      80.050  79.952 254.311  1.00  0.84
O
ATOM   3170  CB  GLU A 474      81.403  81.049 251.430  1.00  0.84
C
ATOM   3171  CG  GLU A 474      79.999  81.725 251.343  1.00  0.84
C
ATOM   3172  CD  GLU A 474      79.810  83.068 252.062  1.00  0.84
C
ATOM   3173  OE1 GLU A 474      80.675  83.473 252.869  1.00  0.84
O
ATOM   3174  OE2 GLU A 474      78.728  83.685 251.821  1.00  0.84
O
ATOM   3175  N   GLY A 475      80.674  78.335 252.884  1.00  0.92
N
ATOM   3176  CA  GLY A 475      79.870  77.268 253.481  1.00  0.92
C
ATOM   3177  C   GLY A 475      80.107  77.039 254.957  1.00  0.92
C
ATOM   3178  O   GLY A 475      79.175  77.037 255.763  1.00  0.92
O
ATOM   3179  N   MET A 476      81.381  76.854 255.359  1.00  0.85
N
```

```
ATOM   3180  CA  MET A 476      81.786  76.704 256.746  1.00  0.85
C
ATOM   3181  C   MET A 476      81.455  77.924 257.595  1.00  0.85
C
ATOM   3182  O   MET A 476      80.996  77.800 258.732  1.00  0.85
O
ATOM   3183  CB  MET A 476      83.302  76.419 256.872  1.00  0.85
C
ATOM   3184  CG  MET A 476      83.749  75.028 256.385  1.00  0.85
C
ATOM   3185  SD  MET A 476      85.552  74.785 256.499  1.00  0.85
S
ATOM   3186  CE  MET A 476      85.721  74.691 258.309  1.00  0.85
C
ATOM   3187  N   LYS A 477      81.666  79.146 257.062  1.00  0.83
N
ATOM   3188  CA  LYS A 477      81.319  80.378 257.746  1.00  0.83
C
ATOM   3189  C   LYS A 477      79.834  80.507 258.039  1.00  0.83
C
ATOM   3190  O   LYS A 477      79.442  80.734 259.183  1.00  0.83
O
ATOM   3191  CB  LYS A 477      81.787  81.599 256.910  1.00  0.83
C
ATOM   3192  CG  LYS A 477      81.331  82.991 257.402  1.00  0.83
C
ATOM   3193  CD  LYS A 477      81.703  83.336 258.858  1.00  0.83
C
ATOM   3194  CE  LYS A 477      83.199  83.566 259.085  1.00  0.83
C
ATOM   3195  NZ  LYS A 477      83.468  83.750 260.531  1.00  0.83
N
ATOM   3196  N   ARG A 478      78.975  80.298 257.028  1.00  0.79
N
ATOM   3197  CA  ARG A 478      77.536  80.377 257.176  1.00  0.79
C
ATOM   3198  C   ARG A 478      76.964  79.332 258.105  1.00  0.79
C
ATOM   3199  O   ARG A 478      76.072  79.597 258.904  1.00  0.79
O
ATOM   3200  CB  ARG A 478      76.864  80.256 255.807  1.00  0.79
C
ATOM   3201  CG  ARG A 478      77.091  81.493 254.930  1.00  0.79
C
ATOM   3202  CD  ARG A 478      76.530  81.281 253.534  1.00  0.79
C
ATOM   3203  NE  ARG A 478      76.568  82.584 252.830  1.00  0.79
N
ATOM   3204  CZ  ARG A 478      75.497  83.271 252.425  1.00  0.79
C
ATOM   3205  NH1 ARG A 478      74.278  82.919 252.810  1.00  0.79
N
ATOM   3206  NH2 ARG A 478      75.684  84.316 251.630  1.00  0.79
N
```

```
ATOM   3207  N   SER A 479      77.504  78.105 258.046  1.00  0.84
N
ATOM   3208  CA  SER A 479      77.125  77.034 258.953  1.00  0.84
C
ATOM   3209  C   SER A 479      77.375  77.366 260.421  1.00  0.84
C
ATOM   3210  O   SER A 479      76.523  77.149 261.280  1.00  0.84
O
ATOM   3211  CB  SER A 479      77.890  75.755 258.574  1.00  0.84
C
ATOM   3212  OG  SER A 479      77.457  74.636 259.336  1.00  0.84
O
ATOM   3213  N   LEU A 480      78.539  77.976 260.737  1.00  0.81
N
ATOM   3214  CA  LEU A 480      78.827  78.472 262.073  1.00  0.81
C
ATOM   3215  C   LEU A 480      77.882  79.590 262.492  1.00  0.81
C
ATOM   3216  O   LEU A 480      77.345  79.585 263.593  1.00  0.81
O
ATOM   3217  CB  LEU A 480      80.298  78.934 262.167  1.00  0.81
C
ATOM   3218  CG  LEU A 480      80.798  79.303 263.580  1.00  0.81
C
ATOM   3219  CD1 LEU A 480      80.720  78.122 264.559  1.00  0.81
C
ATOM   3220  CD2 LEU A 480      82.247  79.806 263.523  1.00  0.81
C
ATOM   3221  N   GLN A 481      77.578  80.539 261.576  1.00  0.80
N
ATOM   3222  CA  GLN A 481      76.631  81.619 261.820  1.00  0.80
C
ATOM   3223  C   GLN A 481      75.241  81.129 262.202  1.00  0.80
C
ATOM   3224  O   GLN A 481      74.615  81.676 263.106  1.00  0.80
O
ATOM   3225  CB  GLN A 481      76.509  82.559 260.592  1.00  0.80
C
ATOM   3226  CG  GLN A 481      77.771  83.408 260.303  1.00  0.80
C
ATOM   3227  CD  GLN A 481      77.652  84.150 258.968  1.00  0.80
C
ATOM   3228  OE1 GLN A 481      77.028  83.694 258.013  1.00  0.80
O
ATOM   3229  NE2 GLN A 481      78.298  85.336 258.865  1.00  0.80
N
ATOM   3230  N   GLN A 482      74.743  80.055 261.556  1.00  0.76
N
ATOM   3231  CA  GLN A 482      73.509  79.407 261.955  1.00  0.76
C
ATOM   3232  C   GLN A 482      73.563  78.826 263.366  1.00  0.76
C
ATOM   3233  O   GLN A 482      72.663  79.024 264.180  1.00  0.76
O
```

```
ATOM   3234  CB  GLN A 482      73.172  78.290 260.938  1.00  0.76
C
ATOM   3235  CG  GLN A 482      71.783  77.632 261.119  1.00  0.76
C
ATOM   3236  CD  GLN A 482      70.654  78.656 260.965  1.00  0.76
C
ATOM   3237  OE1 GLN A 482      70.707  79.556 260.127  1.00  0.76
O
ATOM   3238  NE2 GLN A 482      69.609  78.535 261.813  1.00  0.76
N
ATOM   3239  N   LEU A 483      74.670  78.141 263.715  1.00  0.79
N
ATOM   3240  CA  LEU A 483      74.866  77.535 265.020  1.00  0.79
C
ATOM   3241  C   LEU A 483      75.006  78.539 266.150  1.00  0.79
C
ATOM   3242  O   LEU A 483      74.653  78.255 267.293  1.00  0.79
O
ATOM   3243  CB  LEU A 483      76.101  76.612 265.005  1.00  0.79
C
ATOM   3244  CG  LEU A 483      75.870  75.297 264.237  1.00  0.79
C
ATOM   3245  CD1 LEU A 483      77.212  74.602 263.982  1.00  0.79
C
ATOM   3246  CD2 LEU A 483      74.919  74.355 264.994  1.00  0.79
C
ATOM   3247  N   GLU A 484      75.468  79.773 265.871  1.00  0.73
N
ATOM   3248  CA  GLU A 484      75.662  80.773 266.905  1.00  0.73
C
ATOM   3249  C   GLU A 484      74.374  81.402 267.425  1.00  0.73
C
ATOM   3250  O   GLU A 484      74.405  82.190 268.366  1.00  0.73
O
ATOM   3251  CB  GLU A 484      76.653  81.894 266.504  1.00  0.73
C
ATOM   3252  CG  GLU A 484      78.115  81.391 266.296  1.00  0.73
C
ATOM   3253  CD  GLU A 484      79.192  82.441 266.466  1.00  0.73
C
ATOM   3254  OE1 GLU A 484      78.901  83.362 267.204  1.00  0.73
O
ATOM   3255  OE2 GLU A 484      80.262  82.400 265.819  1.00  0.73
O
ATOM   3256  N   SER A 485      73.187  81.024 266.899  1.00  0.77
N
ATOM   3257  CA  SER A 485      71.932  81.229 267.614  1.00  0.77
C
ATOM   3258  C   SER A 485      71.352  79.902 268.089  1.00  0.77
C
ATOM   3259  O   SER A 485      70.557  79.272 267.404  1.00  0.77
O
ATOM   3260  CB  SER A 485      70.835  81.909 266.762  1.00  0.77
C
```

ATOM 3261 OG SER A 485 69.747 82.359 267.579 1.00 0.77
O
ATOM 3262 N ARG A 486 71.678 79.372 269.277 1.00 0.65
N
ATOM 3263 CA ARG A 486 72.668 79.813 270.239 1.00 0.65
C
ATOM 3264 C ARG A 486 73.381 78.568 270.750 1.00 0.65
C
ATOM 3265 O ARG A 486 73.518 78.329 271.944 1.00 0.65
O
ATOM 3266 CB ARG A 486 72.027 80.587 271.426 1.00 0.65
C
ATOM 3267 CG ARG A 486 71.203 81.826 271.010 1.00 0.65
C
ATOM 3268 CD ARG A 486 70.595 82.626 272.164 1.00 0.65
C
ATOM 3269 NE ARG A 486 71.741 83.243 272.902 1.00 0.65
N
ATOM 3270 CZ ARG A 486 71.641 83.907 274.060 1.00 0.65
C
ATOM 3271 NH1 ARG A 486 72.750 84.360 274.641 1.00 0.65
N
ATOM 3272 NH2 ARG A 486 70.467 84.122 274.647 1.00 0.65
N
ATOM 3273 N GLY A 487 73.812 77.688 269.827 1.00 0.76
N
ATOM 3274 CA GLY A 487 74.270 76.340 270.145 1.00 0.76
C
ATOM 3275 C GLY A 487 75.649 76.265 270.723 1.00 0.76
C
ATOM 3276 O GLY A 487 75.960 75.334 271.455 1.00 0.76
O
ATOM 3277 N ARG A 488 76.477 77.298 270.480 1.00 0.59
N
ATOM 3278 CA ARG A 488 77.825 77.474 270.994 1.00 0.59
C
ATOM 3279 C ARG A 488 77.935 77.339 272.509 1.00 0.59
C
ATOM 3280 O ARG A 488 78.988 76.995 273.040 1.00 0.59
O
ATOM 3281 CB ARG A 488 78.415 78.833 270.513 1.00 0.59
C
ATOM 3282 CG ARG A 488 77.496 80.067 270.673 1.00 0.59
C
ATOM 3283 CD ARG A 488 78.039 81.349 270.022 1.00 0.59
C
ATOM 3284 NE ARG A 488 79.183 81.825 270.839 1.00 0.59
N
ATOM 3285 CZ ARG A 488 79.925 82.896 270.534 1.00 0.59
C
ATOM 3286 NH1 ARG A 488 79.671 83.703 269.532 1.00 0.59
N
ATOM 3287 NH2 ARG A 488 80.940 83.245 271.316 1.00 0.59
N

ATOM 3288 N ASP A 489 76.815 77.555 273.227 1.00 0.67
N
ATOM 3289 CA ASP A 489 76.746 77.458 274.663 1.00 0.67
C
ATOM 3290 C ASP A 489 76.208 76.097 275.167 1.00 0.67
C
ATOM 3291 O ASP A 489 76.131 75.860 276.371 1.00 0.67
O
ATOM 3292 CB ASP A 489 75.829 78.606 275.178 1.00 0.67
C
ATOM 3293 CG ASP A 489 76.237 79.983 274.648 1.00 0.67
C
ATOM 3294 OD1 ASP A 489 77.457 80.256 274.509 1.00 0.67
O
ATOM 3295 OD2 ASP A 489 75.312 80.793 274.361 1.00 0.67
O
ATOM 3296 N LYS A 490 75.825 75.145 274.277 1.00 0.69
N
ATOM 3297 CA LYS A 490 75.306 73.841 274.692 1.00 0.69
C
ATOM 3298 C LYS A 490 76.246 72.687 274.395 1.00 0.69
C
ATOM 3299 O LYS A 490 76.289 71.694 275.122 1.00 0.69
O
ATOM 3300 CB LYS A 490 74.012 73.455 273.919 1.00 0.69
C
ATOM 3301 CG LYS A 490 72.727 74.127 274.420 1.00 0.69
C
ATOM 3302 CD LYS A 490 71.457 73.495 273.813 1.00 0.69
C
ATOM 3303 CE LYS A 490 71.207 72.043 274.242 1.00 0.69
C
ATOM 3304 NZ LYS A 490 69.931 71.569 273.665 1.00 0.69
N
ATOM 3305 N VAL A 491 76.976 72.760 273.275 1.00 0.69
N
ATOM 3306 CA VAL A 491 77.741 71.649 272.752 1.00 0.69
C
ATOM 3307 C VAL A 491 79.231 71.865 272.989 1.00 0.69
C
ATOM 3308 O VAL A 491 79.720 72.972 273.176 1.00 0.69
O
ATOM 3309 CB VAL A 491 77.439 71.377 271.274 1.00 0.69
C
ATOM 3310 CG1 VAL A 491 76.252 70.400 271.136 1.00 0.69
C
ATOM 3311 CG2 VAL A 491 77.120 72.681 270.529 1.00 0.69
C
ATOM 3312 N LEU A 492 80.016 70.765 273.037 1.00 0.71
N
ATOM 3313 CA LEU A 492 81.465 70.802 273.180 1.00 0.71
C
ATOM 3314 C LEU A 492 82.156 71.519 272.030 1.00 0.71
C

ATOM   3315 O   LEU A 492   81.643 71.539 270.917 1.00 0.71
O
ATOM   3316 CB LEU A 492   82.111 69.393 273.206 1.00 0.71
C
ATOM   3317 CG LEU A 492   81.390 68.294 273.999 1.00 0.71
C
ATOM   3318 CD1 LEU A 492   82.126 66.975 273.730 1.00 0.71
C
ATOM   3319 CD2 LEU A 492   81.320 68.579 275.504 1.00 0.71
C
ATOM   3320 N   THR A 493   83.382 72.044 272.232 1.00 0.64
N
ATOM   3321 CA THR A 493   84.173 72.574 271.109 1.00 0.64
C
ATOM   3322 C   THR A 493   84.436 71.580 269.970 1.00 0.64
C
ATOM   3323 O   THR A 493   84.285 71.999 268.829 1.00 0.64
O
ATOM   3324 CB THR A 493   85.401 73.401 271.518 1.00 0.64
C
ATOM   3325 OG1 THR A 493   86.184 73.822 270.407 1.00 0.64
O
ATOM   3326 CG2 THR A 493   86.333 72.674 272.492 1.00 0.64
C
ATOM   3327 N   PRO A 494   84.729 70.282 270.112 1.00 0.68
N
ATOM   3328 CA PRO A 494   84.581 69.332 269.010 1.00 0.68
C
ATOM   3329 C   PRO A 494   83.213 69.296 268.350 1.00 0.68
C
ATOM   3330 O   PRO A 494   83.125 69.442 267.136 1.00 0.68
O
ATOM   3331 CB PRO A 494   84.974 67.969 269.605 1.00 0.68
C
ATOM   3332 CG PRO A 494   85.864 68.301 270.810 1.00 0.68
C
ATOM   3333 CD PRO A 494   85.402 69.687 271.261 1.00 0.68
C
ATOM   3334 N   GLN A 495   82.134 69.156 269.144 1.00 0.67
N
ATOM   3335 CA GLN A 495   80.779 68.992 268.652 1.00 0.67
C
ATOM   3336 C   GLN A 495   80.297 70.165 267.795 1.00 0.67
C
ATOM   3337 O   GLN A 495   79.554 69.977 266.844 1.00 0.67
O
ATOM   3338 CB GLN A 495   79.779 68.796 269.820 1.00 0.67
C
ATOM   3339 CG GLN A 495   79.978 67.527 270.686 1.00 0.67
C
ATOM   3340 CD GLN A 495   79.559 66.226 269.993 1.00 0.67
C
ATOM   3341 OE1 GLN A 495   78.438 66.093 269.512 1.00 0.67
O

ATOM 3342 NE2 GLN A 495 80.427 65.191 270.038 1.00 0.67
N
ATOM 3343 N GLU A 496 80.690 71.413 268.118 1.00 0.67
N
ATOM 3344 CA GLU A 496 80.185 72.589 267.431 1.00 0.67
C
ATOM 3345 C GLU A 496 81.201 73.171 266.481 1.00 0.67
C
ATOM 3346 O GLU A 496 81.024 73.122 265.263 1.00 0.67
O
ATOM 3347 CB GLU A 496 79.812 73.677 268.449 1.00 0.67
C
ATOM 3348 CG GLU A 496 79.047 74.886 267.852 1.00 0.67
C
ATOM 3349 CD GLU A 496 79.773 76.222 268.005 1.00 0.67
C
ATOM 3350 OE1 GLU A 496 79.125 77.237 267.649 1.00 0.67
O
ATOM 3351 OE2 GLU A 496 80.940 76.250 268.472 1.00 0.67
O
ATOM 3352 N LEU A 497 82.312 73.694 267.048 1.00 0.70
N
ATOM 3353 CA LEU A 497 83.453 74.315 266.395 1.00 0.70
C
ATOM 3354 C LEU A 497 84.181 73.431 265.390 1.00 0.70
C
ATOM 3355 O LEU A 497 85.121 73.865 264.726 1.00 0.70
O
ATOM 3356 CB LEU A 497 84.457 74.821 267.473 1.00 0.70
C
ATOM 3357 CG LEU A 497 84.455 76.340 267.736 1.00 0.70
C
ATOM 3358 CD1 LEU A 497 85.300 76.664 268.981 1.00 0.70
C
ATOM 3359 CD2 LEU A 497 84.970 77.129 266.523 1.00 0.70
C
ATOM 3360 N LYS A 498 83.733 72.178 265.211 1.00 0.74
N
ATOM 3361 CA LYS A 498 83.991 71.460 263.994 1.00 0.74
C
ATOM 3362 C LYS A 498 82.803 70.616 263.572 1.00 0.74
C
ATOM 3363 O LYS A 498 82.242 70.842 262.501 1.00 0.74
O
ATOM 3364 CB LYS A 498 85.240 70.574 264.138 1.00 0.74
C
ATOM 3365 CG LYS A 498 85.613 69.896 262.817 1.00 0.74
C
ATOM 3366 CD LYS A 498 86.957 69.170 262.869 1.00 0.74
C
ATOM 3367 CE LYS A 498 87.286 68.541 261.519 1.00 0.74
C
ATOM 3368 NZ LYS A 498 88.607 67.899 261.588 1.00 0.74
N

```
ATOM   3369  N   ASP A 499      82.396  69.606 264.377  1.00  0.78
N
ATOM   3370  CA  ASP A 499      81.527  68.532 263.922  1.00  0.78
C
ATOM   3371  C   ASP A 499      80.214  69.001 263.311  1.00  0.78
C
ATOM   3372  O   ASP A 499      79.924  68.716 262.150  1.00  0.78
O
ATOM   3373  CB  ASP A 499      81.212  67.545 265.082  1.00  0.78
C
ATOM   3374  CG  ASP A 499      82.430  66.767 265.563  1.00  0.78
C
ATOM   3375  OD1 ASP A 499      83.384  66.586 264.763  1.00  0.78
O
ATOM   3376  OD2 ASP A 499      82.380  66.297 266.732  1.00  0.78
O
ATOM   3377  N   ALA A 500      79.405  69.792 264.046  1.00  0.85
N
ATOM   3378  CA  ALA A 500      78.167  70.334 263.534  1.00  0.85
C
ATOM   3379  C   ALA A 500      78.374  71.300 262.379  1.00  0.85
C
ATOM   3380  O   ALA A 500      77.684  71.224 261.363  1.00  0.85
O
ATOM   3381  CB  ALA A 500      77.353  71.009 264.656  1.00  0.85
C
ATOM   3382  N   GLN A 501      79.369  72.206 262.485  1.00  0.79
N
ATOM   3383  CA  GLN A 501      79.659  73.169 261.444  1.00  0.79
C
ATOM   3384  C   GLN A 501      80.044  72.543 260.106  1.00  0.79
C
ATOM   3385  O   GLN A 501      79.437  72.841 259.074  1.00  0.79
O
ATOM   3386  CB  GLN A 501      80.815  74.086 261.904  1.00  0.79
C
ATOM   3387  CG  GLN A 501      81.243  75.164 260.879  1.00  0.79
C
ATOM   3388  CD  GLN A 501      82.614  75.741 261.231  1.00  0.79
C
ATOM   3389  OE1 GLN A 501      83.409  75.169 261.968  1.00  0.79
O
ATOM   3390  NE2 GLN A 501      82.933  76.918 260.647  1.00  0.79
N
ATOM   3391  N   GLU A 502      81.030  71.624 260.089  1.00  0.82
N
ATOM   3392  CA  GLU A 502      81.469  70.945 258.885  1.00  0.82
C
ATOM   3393  C   GLU A 502      80.405  70.019 258.327  1.00  0.82
C
ATOM   3394  O   GLU A 502      80.169  69.982 257.120  1.00  0.82
O
ATOM   3395  CB  GLU A 502      82.773  70.145 259.107  1.00  0.82
C
```

ATOM 3396 CG GLU A 502 84.033 71.024 259.315 1.00 0.82
C
ATOM 3397 CD GLU A 502 85.334 70.226 259.211 1.00 0.82
C
ATOM 3398 OE1 GLU A 502 85.282 68.968 259.167 1.00 0.82
O
ATOM 3399 OE2 GLU A 502 86.419 70.864 259.181 1.00 0.82
O
ATOM 3400 N SER A 503 79.701 69.272 259.206 1.00 0.87
N
ATOM 3401 CA SER A 503 78.640 68.341 258.814 1.00 0.87
C
ATOM 3402 C SER A 503 77.512 69.009 258.058 1.00 0.87
C
ATOM 3403 O SER A 503 77.140 68.573 256.971 1.00 0.87
O
ATOM 3404 CB SER A 503 78.030 67.659 260.074 1.00 0.87
C
ATOM 3405 OG SER A 503 76.964 66.741 259.874 1.00 0.87
O
ATOM 3406 N LEU A 504 76.991 70.139 258.577 1.00 0.88
N
ATOM 3407 CA LEU A 504 75.999 70.926 257.879 1.00 0.88
C
ATOM 3408 C LEU A 504 76.536 71.596 256.621 1.00 0.88
C
ATOM 3409 O LEU A 504 75.918 71.525 255.564 1.00 0.88
O
ATOM 3410 CB LEU A 504 75.418 72.009 258.814 1.00 0.88
C
ATOM 3411 CG LEU A 504 74.662 71.475 260.045 1.00 0.88
C
ATOM 3412 CD1 LEU A 504 74.435 72.604 261.060 1.00 0.88
C
ATOM 3413 CD2 LEU A 504 73.312 70.853 259.682 1.00 0.88
C
ATOM 3414 N ALA A 505 77.725 72.237 256.672 1.00 0.92
N
ATOM 3415 CA ALA A 505 78.254 72.972 255.536 1.00 0.92
C
ATOM 3416 C ALA A 505 78.535 72.115 254.315 1.00 0.92
C
ATOM 3417 O ALA A 505 78.044 72.375 253.218 1.00 0.92
O
ATOM 3418 CB ALA A 505 79.576 73.653 255.938 1.00 0.92
C
ATOM 3419 N TYR A 506 79.289 71.017 254.499 1.00 0.88
N
ATOM 3420 CA TYR A 506 79.588 70.079 253.438 1.00 0.88
C
ATOM 3421 C TYR A 506 78.373 69.271 253.061 1.00 0.88
C
ATOM 3422 O TYR A 506 78.174 68.918 251.899 1.00 0.88
O

```
ATOM   3423  CB  TYR A 506      80.718  69.107 253.851  1.00  0.88
C
ATOM   3424  CG  TYR A 506      82.012  69.819 254.143  1.00  0.88
C
ATOM   3425  CD1 TYR A 506      82.473  70.891 253.357  1.00  0.88
C
ATOM   3426  CD2 TYR A 506      82.812  69.378 255.210  1.00  0.88
C
ATOM   3427  CE1 TYR A 506      83.693  71.517 253.650  1.00  0.88
C
ATOM   3428  CE2 TYR A 506      84.030  70.003 255.504  1.00  0.88
C
ATOM   3429  CZ  TYR A 506      84.467  71.076 254.727  1.00  0.88
C
ATOM   3430  OH  TYR A 506      85.688  71.700 255.037  1.00  0.88
O
ATOM   3431  N   GLY A 507      77.506  68.976 254.048  1.00  0.89
N
ATOM   3432  CA  GLY A 507      76.304  68.207 253.825  1.00  0.89
C
ATOM   3433  C   GLY A 507      75.306  68.907 252.960  1.00  0.89
C
ATOM   3434  O   GLY A 507      74.726  68.328 252.050  1.00  0.89
O
ATOM   3435  N   CYS A 508      75.103  70.205 253.191  1.00  0.89
N
ATOM   3436  CA  CYS A 508      74.238  71.027 252.378  1.00  0.89
C
ATOM   3437  C   CYS A 508      74.741  71.248 250.971  1.00  0.89
C
ATOM   3438  O   CYS A 508      73.963  71.163 250.026  1.00  0.89
O
ATOM   3439  CB  CYS A 508      73.933  72.350 253.087  1.00  0.89
C
ATOM   3440  SG  CYS A 508      72.959  71.994 254.575  1.00  0.89
S
ATOM   3441  N   ILE A 509      76.060  71.473 250.780  1.00  0.88
N
ATOM   3442  CA  ILE A 509      76.658  71.603 249.453  1.00  0.88
C
ATOM   3443  C   ILE A 509      76.460  70.344 248.622  1.00  0.88
C
ATOM   3444  O   ILE A 509      76.064  70.405 247.459  1.00  0.88
O
ATOM   3445  CB  ILE A 509      78.139  71.986 249.551  1.00  0.88
C
ATOM   3446  CG1 ILE A 509      78.248  73.441 250.071  1.00  0.88
C
ATOM   3447  CG2 ILE A 509      78.868  71.832 248.192  1.00  0.88
C
ATOM   3448  CD1 ILE A 509      79.684  73.926 250.293  1.00  0.88
C
ATOM   3449  N   LYS A 510      76.680  69.160 249.227  1.00  0.87
N
```

ATOM 3450 CA LYS A 510 76.392 67.898 248.581 1.00 0.87
C
ATOM 3451 C LYS A 510 74.913 67.602 248.377 1.00 0.87
C
ATOM 3452 O LYS A 510 74.493 67.233 247.285 1.00 0.87
O
ATOM 3453 CB LYS A 510 77.022 66.747 249.386 1.00 0.87
C
ATOM 3454 CG LYS A 510 78.553 66.800 249.423 1.00 0.87
C
ATOM 3455 CD LYS A 510 79.129 65.712 250.337 1.00 0.87
C
ATOM 3456 CE LYS A 510 80.636 65.863 250.529 1.00 0.87
C
ATOM 3457 NZ LYS A 510 81.149 64.803 251.411 1.00 0.87
N
ATOM 3458 N TYR A 511 74.060 67.776 249.407 1.00 0.89
N
ATOM 3459 CA TYR A 511 72.644 67.464 249.318 1.00 0.89
C
ATOM 3460 C TYR A 511 71.912 68.331 248.314 1.00 0.89
C
ATOM 3461 O TYR A 511 71.072 67.847 247.572 1.00 0.89
O
ATOM 3462 CB TYR A 511 71.971 67.590 250.705 1.00 0.89
C
ATOM 3463 CG TYR A 511 70.611 66.956 250.732 1.00 0.89
C
ATOM 3464 CD1 TYR A 511 70.497 65.566 250.865 1.00 0.89
C
ATOM 3465 CD2 TYR A 511 69.443 67.725 250.606 1.00 0.89
C
ATOM 3466 CE1 TYR A 511 69.245 64.951 250.899 1.00 0.89
C
ATOM 3467 CE2 TYR A 511 68.184 67.110 250.635 1.00 0.89
C
ATOM 3468 CZ TYR A 511 68.093 65.722 250.769 1.00 0.89
C
ATOM 3469 OH TYR A 511 66.858 65.061 250.738 1.00 0.89
O
ATOM 3470 N SER A 512 72.238 69.634 248.261 1.00 0.89
N
ATOM 3471 CA SER A 512 71.620 70.592 247.352 1.00 0.89
C
ATOM 3472 C SER A 512 71.839 70.242 245.893 1.00 0.89
C
ATOM 3473 O SER A 512 70.949 70.405 245.067 1.00 0.89
O
ATOM 3474 CB SER A 512 72.120 72.027 247.637 1.00 0.89
C
ATOM 3475 OG SER A 512 71.345 73.045 246.999 1.00 0.89
O
ATOM 3476 N ASP A 513 73.030 69.715 245.535 1.00 0.88
N

ATOM 3477 CA ASP A 513 73.236 69.127 244.228 1.00 0.88
C
ATOM 3478 C ASP A 513 72.466 67.816 244.041 1.00 0.88
C
ATOM 3479 O ASP A 513 71.675 67.661 243.110 1.00 0.88
O
ATOM 3480 CB ASP A 513 74.757 68.894 244.051 1.00 0.88
C
ATOM 3481 CG ASP A 513 75.111 68.668 242.596 1.00 0.88
C
ATOM 3482 OD1 ASP A 513 74.573 67.736 241.946 1.00 0.88
O
ATOM 3483 OD2 ASP A 513 75.908 69.469 242.057 1.00 0.88
O
ATOM 3484 N LEU A 514 72.639 66.853 244.973 1.00 0.88
N
ATOM 3485 CA LEU A 514 72.171 65.498 244.767 1.00 0.88
C
ATOM 3486 C LEU A 514 70.674 65.322 244.927 1.00 0.88
C
ATOM 3487 O LEU A 514 70.127 64.320 244.483 1.00 0.88
O
ATOM 3488 CB LEU A 514 72.865 64.496 245.730 1.00 0.88
C
ATOM 3489 CG LEU A 514 74.399 64.383 245.601 1.00 0.88
C
ATOM 3490 CD1 LEU A 514 74.968 63.410 246.645 1.00 0.88
C
ATOM 3491 CD2 LEU A 514 74.849 63.946 244.204 1.00 0.88
C
ATOM 3492 N CYS A 515 69.952 66.266 245.567 1.00 0.87
N
ATOM 3493 CA CYS A 515 68.526 66.137 245.797 1.00 0.87
C
ATOM 3494 C CYS A 515 67.682 66.539 244.609 1.00 0.87
C
ATOM 3495 O CYS A 515 66.489 66.258 244.573 1.00 0.87
O
ATOM 3496 CB CYS A 515 68.059 66.910 247.062 1.00 0.87
C
ATOM 3497 SG CYS A 515 68.064 68.738 246.889 1.00 0.87
S
ATOM 3498 N HIS A 516 68.298 67.169 243.597 1.00 0.80
N
ATOM 3499 CA HIS A 516 67.654 67.464 242.337 1.00 0.80
C
ATOM 3500 C HIS A 516 68.143 66.494 241.287 1.00 0.80
C
ATOM 3501 O HIS A 516 69.173 65.843 241.440 1.00 0.80
O
ATOM 3502 CB HIS A 516 67.960 68.899 241.874 1.00 0.80
C
ATOM 3503 CG HIS A 516 67.388 69.901 242.818 1.00 0.80
C

```
ATOM   3504  ND1 HIS A 516      66.018  69.976 242.974  1.00  0.80
N
ATOM   3505  CD2 HIS A 516      68.009  70.761 243.663  1.00  0.80
C
ATOM   3506  CE1 HIS A 516      65.834  70.875 243.919  1.00  0.80
C
ATOM   3507  NE2 HIS A 516      67.006  71.383 244.371  1.00  0.80
N
ATOM   3508  N   ASN A 517      67.411  66.344 240.160  1.00  0.82
N
ATOM   3509  CA  ASN A 517      67.890  65.535 239.052  1.00  0.82
C
ATOM   3510  C   ASN A 517      69.205  66.089 238.520  1.00  0.82
C
ATOM   3511  O   ASN A 517      69.332  67.269 238.217  1.00  0.82
O
ATOM   3512  CB  ASN A 517      66.815  65.421 237.927  1.00  0.82
C
ATOM   3513  CG  ASN A 517      67.137  64.367 236.861  1.00  0.82
C
ATOM   3514  OD1 ASN A 517      68.259  64.207 236.382  1.00  0.82
O
ATOM   3515  ND2 ASN A 517      66.087  63.623 236.439  1.00  0.82
N
ATOM   3516  N   ARG A 518      70.228  65.230 238.383  1.00  0.80
N
ATOM   3517  CA  ARG A 518      71.539  65.629 237.926  1.00  0.80
C
ATOM   3518  C   ARG A 518      71.525  66.280 236.549  1.00  0.80
C
ATOM   3519  O   ARG A 518      72.250  67.239 236.310  1.00  0.80
O
ATOM   3520  CB  ARG A 518      72.514  64.428 237.964  1.00  0.80
C
ATOM   3521  CG  ARG A 518      71.966  63.145 237.309  1.00  0.80
C
ATOM   3522  CD  ARG A 518      73.028  62.062 237.125  1.00  0.80
C
ATOM   3523  NE  ARG A 518      72.351  60.849 236.555  1.00  0.80
N
ATOM   3524  CZ  ARG A 518      72.263  60.554 235.252  1.00  0.80
C
ATOM   3525  NH1 ARG A 518      72.683  61.397 234.312  1.00  0.80
N
ATOM   3526  NH2 ARG A 518      71.775  59.375 234.872  1.00  0.80
N
ATOM   3527  N   ILE A 519      70.651  65.806 235.632  1.00  0.85
N
ATOM   3528  CA  ILE A 519      70.471  66.326 234.284  1.00  0.85
C
ATOM   3529  C   ILE A 519      70.100  67.797 234.257  1.00  0.85
C
ATOM   3530  O   ILE A 519      70.563  68.535 233.391  1.00  0.85
O
```

ATOM 3531 CB ILE A 519 69.419 65.514 233.520 1.00 0.85
C
ATOM 3532 CG1 ILE A 519 69.933 64.083 233.236 1.00 0.85
C
ATOM 3533 CG2 ILE A 519 68.983 66.207 232.202 1.00 0.85
C
ATOM 3534 CD1 ILE A 519 68.828 63.145 232.736 1.00 0.85
C
ATOM 3535 N SER A 520 69.248 68.263 235.189 1.00 0.85
N
ATOM 3536 CA SER A 520 68.850 69.654 235.220 1.00 0.85
C
ATOM 3537 C SER A 520 69.943 70.587 235.683 1.00 0.85
C
ATOM 3538 O SER A 520 70.843 70.232 236.450 1.00 0.85
O
ATOM 3539 CB SER A 520 67.552 69.927 236.029 1.00 0.85
C
ATOM 3540 OG SER A 520 67.653 69.530 237.395 1.00 0.85
O
ATOM 3541 N ASP A 521 69.888 71.836 235.181 1.00 0.85
N
ATOM 3542 CA ASP A 521 70.710 72.918 235.658 1.00 0.85
C
ATOM 3543 C ASP A 521 70.451 73.208 237.127 1.00 0.85
C
ATOM 3544 O ASP A 521 69.326 73.143 237.625 1.00 0.85
O
ATOM 3545 CB ASP A 521 70.504 74.194 234.816 1.00 0.85
C
ATOM 3546 CG ASP A 521 70.872 73.933 233.366 1.00 0.85
C
ATOM 3547 OD1 ASP A 521 71.962 73.353 233.120 1.00 0.85
O
ATOM 3548 OD2 ASP A 521 70.055 74.298 232.490 1.00 0.85
O
ATOM 3549 N TYR A 522 71.525 73.504 237.867 1.00 0.85
N
ATOM 3550 CA TYR A 522 71.495 73.555 239.306 1.00 0.85
C
ATOM 3551 C TYR A 522 71.759 74.983 239.749 1.00 0.85
C
ATOM 3552 O TYR A 522 72.813 75.565 239.495 1.00 0.85
O
ATOM 3553 CB TYR A 522 72.529 72.527 239.859 1.00 0.85
C
ATOM 3554 CG TYR A 522 72.888 72.669 241.318 1.00 0.85
C
ATOM 3555 CD1 TYR A 522 71.911 72.879 242.305 1.00 0.85
C
ATOM 3556 CD2 TYR A 522 74.236 72.606 241.706 1.00 0.85
C
ATOM 3557 CE1 TYR A 522 72.284 73.099 243.639 1.00 0.85
C

```
ATOM   3558  CE2 TYR A 522      74.608  72.806 243.041  1.00  0.85
C
ATOM   3559  CZ  TYR A 522      73.632  73.083 244.002  1.00  0.85
C
ATOM   3560  OH  TYR A 522      74.016  73.335 245.333  1.00  0.85
O
ATOM   3561  N   ILE A 523      70.778  75.587 240.450  1.00  0.85
N
ATOM   3562  CA  ILE A 523      70.936  76.899 241.048  1.00  0.85
C
ATOM   3563  C   ILE A 523      71.694  76.751 242.354  1.00  0.85
C
ATOM   3564  O   ILE A 523      71.178  76.226 243.341  1.00  0.85
O
ATOM   3565  CB  ILE A 523      69.598  77.577 241.345  1.00  0.85
C
ATOM   3566  CG1 ILE A 523      68.664  77.648 240.111  1.00  0.85
C
ATOM   3567  CG2 ILE A 523      69.827  78.981 241.948  1.00  0.85
C
ATOM   3568  CD1 ILE A 523      69.151  78.556 238.978  1.00  0.85
C
ATOM   3569  N   PHE A 524      72.952  77.219 242.408  1.00  0.86
N
ATOM   3570  CA  PHE A 524      73.718  77.158 243.631  1.00  0.86
C
ATOM   3571  C   PHE A 524      73.329  78.337 244.507  1.00  0.86
C
ATOM   3572  O   PHE A 524      73.660  79.486 244.228  1.00  0.86
O
ATOM   3573  CB  PHE A 524      75.240  77.128 243.326  1.00  0.86
C
ATOM   3574  CG  PHE A 524      76.064  76.846 244.556  1.00  0.86
C
ATOM   3575  CD1 PHE A 524      76.301  77.866 245.489  1.00  0.86
C
ATOM   3576  CD2 PHE A 524      76.608  75.573 244.796  1.00  0.86
C
ATOM   3577  CE1 PHE A 524      77.008  77.608 246.663  1.00  0.86
C
ATOM   3578  CE2 PHE A 524      77.343  75.317 245.963  1.00  0.86
C
ATOM   3579  CZ  PHE A 524      77.535  76.336 246.904  1.00  0.86
C
ATOM   3580  N   SER A 525      72.616  78.058 245.607  1.00  0.87
N
ATOM   3581  CA  SER A 525      72.217  79.071 246.539  1.00  0.87
C
ATOM   3582  C   SER A 525      72.499  78.630 247.952  1.00  0.87
C
ATOM   3583  O   SER A 525      71.933  77.649 248.434  1.00  0.87
O
ATOM   3584  CB  SER A 525      70.718  79.350 246.480  1.00  0.87
C
```

ATOM	3585	OG	SER	A	525	70.365	80.212	247.551	1.00	0.87
O										
ATOM	3586	N	PHE	A	526	73.294	79.434	248.683	1.00	0.86
N										
ATOM	3587	CA	PHE	A	526	73.581	79.181	250.075	1.00	0.86
C										
ATOM	3588	C	PHE	A	526	72.353	79.278	250.957	1.00	0.86
C										
ATOM	3589	O	PHE	A	526	72.138	78.442	251.831	1.00	0.86
O										
ATOM	3590	CB	PHE	A	526	74.669	80.134	250.613	1.00	0.86
C										
ATOM	3591	CG	PHE	A	526	76.042	79.636	250.263	1.00	0.86
C										
ATOM	3592	CD1	PHE	A	526	76.475	78.386	250.739	1.00	0.86
C										
ATOM	3593	CD2	PHE	A	526	76.929	80.418	249.506	1.00	0.86
C										
ATOM	3594	CE1	PHE	A	526	77.751	77.907	250.430	1.00	0.86
C										
ATOM	3595	CE2	PHE	A	526	78.217	79.950	249.216	1.00	0.86
C										
ATOM	3596	CZ	PHE	A	526	78.626	78.694	249.676	1.00	0.86
C										
ATOM	3597	N	ASP	A	527	71.514	80.297	250.721	1.00	0.86
N										
ATOM	3598	CA	ASP	A	527	70.315	80.560	251.477	1.00	0.86
C										
ATOM	3599	C	ASP	A	527	69.269	79.452	251.323	1.00	0.86
C										
ATOM	3600	O	ASP	A	527	68.704	78.993	252.311	1.00	0.86
O										
ATOM	3601	CB	ASP	A	527	69.757	81.961	251.112	1.00	0.86
C										
ATOM	3602	CG	ASP	A	527	70.704	83.115	251.458	1.00	0.86
C										
ATOM	3603	OD1	ASP	A	527	71.848	82.889	251.928	1.00	0.86
O										
ATOM	3604	OD2	ASP	A	527	70.277	84.269	251.235	1.00	0.86
O										
ATOM	3605	N	LYS	A	528	69.017	78.931	250.096	1.00	0.83
N										
ATOM	3606	CA	LYS	A	528	68.160	77.758	249.944	1.00	0.83
C										
ATOM	3607	C	LYS	A	528	68.714	76.485	250.579	1.00	0.83
C										
ATOM	3608	O	LYS	A	528	67.989	75.740	251.228	1.00	0.83
O										
ATOM	3609	CB	LYS	A	528	67.800	77.430	248.467	1.00	0.83
C										
ATOM	3610	CG	LYS	A	528	67.247	78.593	247.627	1.00	0.83
C										
ATOM	3611	CD	LYS	A	528	66.002	79.293	248.192	1.00	0.83
C										

ATOM 3612 CE LYS A 528 65.730 80.601 247.445 1.00 0.83
C
ATOM 3613 NZ LYS A 528 64.704 81.400 248.147 1.00 0.83
N
ATOM 3614 N MET A 529 70.024 76.186 250.424 1.00 0.83
N
ATOM 3615 CA MET A 529 70.564 74.930 250.924 1.00 0.83
C
ATOM 3616 C MET A 529 70.730 74.881 252.434 1.00 0.83
C
ATOM 3617 O MET A 529 70.726 73.806 253.032 1.00 0.83
O
ATOM 3618 CB MET A 529 71.911 74.565 250.252 1.00 0.83
C
ATOM 3619 CG MET A 529 73.139 75.361 250.730 1.00 0.83
C
ATOM 3620 SD MET A 529 74.712 74.839 249.995 1.00 0.83
S
ATOM 3621 CE MET A 529 74.477 75.623 248.381 1.00 0.83
C
ATOM 3622 N LEU A 530 70.874 76.051 253.089 1.00 0.85
N
ATOM 3623 CA LEU A 530 71.085 76.129 254.520 1.00 0.85
C
ATOM 3624 C LEU A 530 69.798 76.411 255.286 1.00 0.85
C
ATOM 3625 O LEU A 530 69.835 76.549 256.501 1.00 0.85
O
ATOM 3626 CB LEU A 530 72.164 77.183 254.897 1.00 0.85
C
ATOM 3627 CG LEU A 530 73.598 76.844 254.434 1.00 0.85
C
ATOM 3628 CD1 LEU A 530 74.567 77.961 254.823 1.00 0.85
C
ATOM 3629 CD2 LEU A 530 74.100 75.523 255.028 1.00 0.85
C
ATOM 3630 N GLU A 531 68.625 76.472 254.617 1.00 0.79
N
ATOM 3631 CA GLU A 531 67.333 76.635 255.273 1.00 0.79
C
ATOM 3632 C GLU A 531 66.976 75.475 256.213 1.00 0.79
C
ATOM 3633 O GLU A 531 67.094 74.303 255.853 1.00 0.79
O
ATOM 3634 CB GLU A 531 66.212 76.853 254.207 1.00 0.79
C
ATOM 3635 CG GLU A 531 64.796 77.162 254.771 1.00 0.79
C
ATOM 3636 CD GLU A 531 63.728 77.548 253.734 1.00 0.79
C
ATOM 3637 OE1 GLU A 531 62.543 77.615 254.169 1.00 0.79
O
ATOM 3638 OE2 GLU A 531 64.056 77.806 252.548 1.00 0.79
O

```
ATOM   3639  N   ASP A 532      66.512  75.768 257.452  1.00  0.78
N
ATOM   3640  CA  ASP A 532      66.240  74.771 258.480  1.00  0.78
C
ATOM   3641  C   ASP A 532      65.029  73.877 258.228  1.00  0.78
C
ATOM   3642  O   ASP A 532      64.860  72.842 258.871  1.00  0.78
O
ATOM   3643  CB  ASP A 532      65.998  75.436 259.868  1.00  0.78
C
ATOM   3644  CG  ASP A 532      67.243  76.073 260.467  1.00  0.78
C
ATOM   3645  OD1 ASP A 532      68.255  76.238 259.752  1.00  0.78
O
ATOM   3646  OD2 ASP A 532      67.183  76.411 261.677  1.00  0.78
O
ATOM   3647  N   ARG A 533      64.130  74.250 257.302  1.00  0.71
N
ATOM   3648  CA  ARG A 533      62.912  73.510 257.056  1.00  0.71
C
ATOM   3649  C   ARG A 533      62.848  73.029 255.622  1.00  0.71
C
ATOM   3650  O   ARG A 533      63.395  73.634 254.709  1.00  0.71
O
ATOM   3651  CB  ARG A 533      61.644  74.342 257.395  1.00  0.71
C
ATOM   3652  CG  ARG A 533      61.534  75.686 256.642  1.00  0.71
C
ATOM   3653  CD  ARG A 533      60.135  76.316 256.600  1.00  0.71
C
ATOM   3654  NE  ARG A 533      59.714  76.601 258.013  1.00  0.71
N
ATOM   3655  CZ  ARG A 533      58.507  77.064 258.369  1.00  0.71
C
ATOM   3656  NH1 ARG A 533      57.557  77.304 257.470  1.00  0.71
N
ATOM   3657  NH2 ARG A 533      58.243  77.286 259.656  1.00  0.71
N
ATOM   3658  N   GLY A 534      62.154  71.897 255.378  1.00  0.85
N
ATOM   3659  CA  GLY A 534      61.987  71.395 254.020  1.00  0.85
C
ATOM   3660  C   GLY A 534      63.073  70.436 253.615  1.00  0.85
C
ATOM   3661  O   GLY A 534      63.724  69.805 254.442  1.00  0.85
O
ATOM   3662  N   ASN A 535      63.256  70.240 252.297  1.00  0.86
N
ATOM   3663  CA  ASN A 535      64.169  69.242 251.773  1.00  0.86
C
ATOM   3664  C   ASN A 535      65.618  69.737 251.704  1.00  0.86
C
ATOM   3665  O   ASN A 535      66.167  69.969 250.627  1.00  0.86
O
```

ATOM 3666 CB ASN A 535 63.651 68.732 250.399 1.00 0.86
C
ATOM 3667 CG ASN A 535 64.257 67.378 250.062 1.00 0.86
C
ATOM 3668 OD1 ASN A 535 64.903 66.747 250.895 1.00 0.86
O
ATOM 3669 ND2 ASN A 535 64.039 66.895 248.819 1.00 0.86
N
ATOM 3670 N THR A 536 66.273 69.889 252.868 1.00 0.86
N
ATOM 3671 CA THR A 536 67.655 70.334 252.977 1.00 0.86
C
ATOM 3672 C THR A 536 68.423 69.383 253.869 1.00 0.86
C
ATOM 3673 O THR A 536 67.853 68.639 254.666 1.00 0.86
O
ATOM 3674 CB THR A 536 67.829 71.737 253.574 1.00 0.86
C
ATOM 3675 OG1 THR A 536 67.365 71.814 254.921 1.00 0.86
O
ATOM 3676 CG2 THR A 536 67.047 72.760 252.744 1.00 0.86
C
ATOM 3677 N ALA A 537 69.773 69.397 253.805 1.00 0.91
N
ATOM 3678 CA ALA A 537 70.552 68.662 254.780 1.00 0.91
C
ATOM 3679 C ALA A 537 70.499 69.276 256.172 1.00 0.91
C
ATOM 3680 O ALA A 537 70.637 68.549 257.148 1.00 0.91
O
ATOM 3681 CB ALA A 537 72.013 68.457 254.348 1.00 0.91
C
ATOM 3682 N VAL A 538 70.238 70.598 256.326 1.00 0.86
N
ATOM 3683 CA VAL A 538 70.041 71.197 257.644 1.00 0.86
C
ATOM 3684 C VAL A 538 68.853 70.589 258.370 1.00 0.86
C
ATOM 3685 O VAL A 538 68.976 70.160 259.518 1.00 0.86
O
ATOM 3686 CB VAL A 538 69.876 72.715 257.605 1.00 0.86
C
ATOM 3687 CG1 VAL A 538 69.687 73.299 259.019 1.00 0.86
C
ATOM 3688 CG2 VAL A 538 71.103 73.370 256.960 1.00 0.86
C
ATOM 3689 N TYR A 539 67.693 70.443 257.688 1.00 0.84
N
ATOM 3690 CA TYR A 539 66.554 69.751 258.262 1.00 0.84
C
ATOM 3691 C TYR A 539 66.856 68.284 258.535 1.00 0.84
C
ATOM 3692 O TYR A 539 66.630 67.778 259.629 1.00 0.84
O

ATOM 3693 CB TYR A 539 65.316 69.865 257.320 1.00 0.84
C
ATOM 3694 CG TYR A 539 64.114 69.112 257.850 1.00 0.84
C
ATOM 3695 CD1 TYR A 539 63.514 69.503 259.054 1.00 0.84
C
ATOM 3696 CD2 TYR A 539 63.656 67.946 257.211 1.00 0.84
C
ATOM 3697 CE1 TYR A 539 62.486 68.737 259.621 1.00 0.84
C
ATOM 3698 CE2 TYR A 539 62.628 67.176 257.776 1.00 0.84
C
ATOM 3699 CZ TYR A 539 62.046 67.571 258.987 1.00 0.84
C
ATOM 3700 OH TYR A 539 61.046 66.785 259.598 1.00 0.84
O
ATOM 3701 N LEU A 540 67.411 67.556 257.553 1.00 0.88
N
ATOM 3702 CA LEU A 540 67.624 66.130 257.698 1.00 0.88
C
ATOM 3703 C LEU A 540 68.650 65.753 258.757 1.00 0.88
C
ATOM 3704 O LEU A 540 68.439 64.816 259.527 1.00 0.88
O
ATOM 3705 CB LEU A 540 67.948 65.495 256.334 1.00 0.88
C
ATOM 3706 CG LEU A 540 66.788 65.587 255.321 1.00 0.88
C
ATOM 3707 CD1 LEU A 540 67.230 65.014 253.977 1.00 0.88
C
ATOM 3708 CD2 LEU A 540 65.509 64.875 255.781 1.00 0.88
C
ATOM 3709 N LEU A 541 69.770 66.495 258.858 1.00 0.87
N
ATOM 3710 CA LEU A 541 70.757 66.315 259.909 1.00 0.87
C
ATOM 3711 C LEU A 541 70.207 66.649 261.288 1.00 0.87
C
ATOM 3712 O LEU A 541 70.444 65.920 262.247 1.00 0.87
O
ATOM 3713 CB LEU A 541 72.053 67.109 259.614 1.00 0.87
C
ATOM 3714 CG LEU A 541 72.876 66.547 258.431 1.00 0.87
C
ATOM 3715 CD1 LEU A 541 73.968 67.534 257.989 1.00 0.87
C
ATOM 3716 CD2 LEU A 541 73.502 65.189 258.779 1.00 0.87
C
ATOM 3717 N TYR A 542 69.400 67.726 261.416 1.00 0.84
N
ATOM 3718 CA TYR A 542 68.680 68.034 262.640 1.00 0.84
C
ATOM 3719 C TYR A 542 67.686 66.941 263.028 1.00 0.84
C

```
ATOM   3720  O   TYR A 542      67.627  66.509 264.174  1.00  0.84
O
ATOM   3721  CB  TYR A 542      67.959  69.403 262.482  1.00  0.84
C
ATOM   3722  CG  TYR A 542      67.175  69.807 263.703  1.00  0.84
C
ATOM   3723  CD1 TYR A 542      67.806  70.402 264.806  1.00  0.84
C
ATOM   3724  CD2 TYR A 542      65.796  69.546 263.765  1.00  0.84
C
ATOM   3725  CE1 TYR A 542      67.070  70.728 265.955  1.00  0.84
C
ATOM   3726  CE2 TYR A 542      65.059  69.871 264.911  1.00  0.84
C
ATOM   3727  CZ  TYR A 542      65.698  70.461 266.007  1.00  0.84
C
ATOM   3728  OH  TYR A 542      64.968  70.717 267.185  1.00  0.84
O
ATOM   3729  N   THR A 543      66.896  66.424 262.070  1.00  0.86
N
ATOM   3730  CA  THR A 543      65.932  65.349 262.296  1.00  0.86
C
ATOM   3731  C   THR A 543      66.604  64.087 262.772  1.00  0.86
C
ATOM   3732  O   THR A 543      66.134  63.426 263.693  1.00  0.86
O
ATOM   3733  CB  THR A 543      65.080  65.071 261.071  1.00  0.86
C
ATOM   3734  OG1 THR A 543      64.311  66.227 260.785  1.00  0.86
O
ATOM   3735  CG2 THR A 543      64.063  63.945 261.296  1.00  0.86
C
ATOM   3736  N   TYR A 544      67.782  63.763 262.209  1.00  0.86
N
ATOM   3737  CA  TYR A 544      68.617  62.669 262.662  1.00  0.86
C
ATOM   3738  C   TYR A 544      69.059  62.791 264.122  1.00  0.86
C
ATOM   3739  O   TYR A 544      68.959  61.830 264.887  1.00  0.86
O
ATOM   3740  CB  TYR A 544      69.839  62.567 261.712  1.00  0.86
C
ATOM   3741  CG  TYR A 544      70.723  61.389 262.020  1.00  0.86
C
ATOM   3742  CD1 TYR A 544      71.891  61.558 262.784  1.00  0.86
C
ATOM   3743  CD2 TYR A 544      70.387  60.106 261.566  1.00  0.86
C
ATOM   3744  CE1 TYR A 544      72.701  60.457 263.101  1.00  0.86
C
ATOM   3745  CE2 TYR A 544      71.198  59.010 261.884  1.00  0.86
C
ATOM   3746  CZ  TYR A 544      72.352  59.180 262.653  1.00  0.86
C
```

```
ATOM   3747  OH  TYR A 544      73.138  58.054 262.988  1.00  0.86
O
ATOM   3748  N   THR A 545      69.498  63.985 264.580  1.00  0.85
N
ATOM   3749  CA  THR A 545      69.854  64.192 265.982  1.00  0.85
C
ATOM   3750  C   THR A 545      68.668  64.034 266.901  1.00  0.85
C
ATOM   3751  O   THR A 545      68.788  63.567 268.033  1.00  0.85
O
ATOM   3752  CB  THR A 545      70.568  65.499 266.312  1.00  0.85
C
ATOM   3753  OG1 THR A 545      69.752  66.651 266.149  1.00  0.85
O
ATOM   3754  CG2 THR A 545      71.791  65.660 265.405  1.00  0.85
C
ATOM   3755  N   ARG A 546      67.458  64.408 266.437  1.00  0.80
N
ATOM   3756  CA  ARG A 546      66.242  64.208 267.200  1.00  0.80
C
ATOM   3757  C   ARG A 546      65.888  62.752 267.391  1.00  0.80
C
ATOM   3758  O   ARG A 546      65.542  62.357 268.499  1.00  0.80
O
ATOM   3759  CB  ARG A 546      65.014  64.988 266.670  1.00  0.80
C
ATOM   3760  CG  ARG A 546      65.256  66.490 266.406  1.00  0.80
C
ATOM   3761  CD  ARG A 546      66.246  67.210 267.329  1.00  0.80
C
ATOM   3762  NE  ARG A 546      65.670  67.214 268.708  1.00  0.80
N
ATOM   3763  CZ  ARG A 546      66.263  67.836 269.732  1.00  0.80
C
ATOM   3764  NH1 ARG A 546      67.488  68.339 269.614  1.00  0.80
N
ATOM   3765  NH2 ARG A 546      65.612  67.958 270.886  1.00  0.80
N
ATOM   3766  N   ILE A 547      66.023  61.926 266.332  1.00  0.87
N
ATOM   3767  CA  ILE A 547      65.750  60.494 266.356  1.00  0.87
C
ATOM   3768  C   ILE A 547      66.682  59.774 267.313  1.00  0.87
C
ATOM   3769  O   ILE A 547      66.245  58.990 268.155  1.00  0.87
O
ATOM   3770  CB  ILE A 547      65.825  59.906 264.947  1.00  0.87
C
ATOM   3771  CG1 ILE A 547      64.717  60.535 264.073  1.00  0.87
C
ATOM   3772  CG2 ILE A 547      65.651  58.373 264.975  1.00  0.87
C
ATOM   3773  CD1 ILE A 547      64.837  60.237 262.578  1.00  0.87
C
```

```
ATOM   3774  N   CYS A 548      67.988  60.108 267.264  1.00   0.88
N
ATOM   3775  CA  CYS A 548      68.979  59.651 268.225  1.00   0.88
C
ATOM   3776  C   CYS A 548      68.725  60.144 269.644  1.00   0.88
C
ATOM   3777  O   CYS A 548      69.024  59.467 270.626  1.00   0.88
O
ATOM   3778  CB  CYS A 548      70.414  60.043 267.791  1.00   0.88
C
ATOM   3779  SG  CYS A 548      70.835  59.315 266.167  1.00   0.88
S
ATOM   3780  N   SER A 549      68.161  61.361 269.814  1.00   0.87
N
ATOM   3781  CA  SER A 549      67.933  61.931 271.135  1.00   0.87
C
ATOM   3782  C   SER A 549      66.896  61.214 271.980  1.00   0.87
C
ATOM   3783  O   SER A 549      66.959  61.268 273.204  1.00   0.87
O
ATOM   3784  CB  SER A 549      67.760  63.486 271.173  1.00   0.87
C
ATOM   3785  OG  SER A 549      66.439  63.991 270.984  1.00   0.87
O
ATOM   3786  N   ILE A 550      65.948  60.476 271.367  1.00   0.83
N
ATOM   3787  CA  ILE A 550      64.919  59.738 272.090  1.00   0.83
C
ATOM   3788  C   ILE A 550      65.462  58.659 273.010  1.00   0.83
C
ATOM   3789  O   ILE A 550      65.117  58.621 274.188  1.00   0.83
O
ATOM   3790  CB  ILE A 550      63.914  59.130 271.116  1.00   0.83
C
ATOM   3791  CG1 ILE A 550      63.193  60.278 270.380  1.00   0.83
C
ATOM   3792  CG2 ILE A 550      62.892  58.225 271.850  1.00   0.83
C
ATOM   3793  CD1 ILE A 550      62.333  59.806 269.212  1.00   0.83
C
ATOM   3794  N   ALA A 551      66.360  57.783 272.515  1.00   0.86
N
ATOM   3795  CA  ALA A 551      67.013  56.780 273.333  1.00   0.86
C
ATOM   3796  C   ALA A 551      68.013  57.396 274.302  1.00   0.86
C
ATOM   3797  O   ALA A 551      68.136  57.021 275.465  1.00   0.86
O
ATOM   3798  CB  ALA A 551      67.679  55.742 272.417  1.00   0.86
C
ATOM   3799  N   ARG A 552      68.729  58.435 273.837  1.00   0.74
N
ATOM   3800  CA  ARG A 552      69.703  59.161 274.620  1.00   0.74
C
```

```
ATOM   3801  C   ARG A 552      69.117  59.823 275.861  1.00  0.74
C
ATOM   3802  O   ARG A 552      69.697  59.784 276.942  1.00  0.74
O
ATOM   3803  CB  ARG A 552      70.326  60.244 273.720  1.00  0.74
C
ATOM   3804  CG  ARG A 552      71.692  60.764 274.197  1.00  0.74
C
ATOM   3805  CD  ARG A 552      72.096  62.048 273.475  1.00  0.74
C
ATOM   3806  NE  ARG A 552      71.262  63.123 274.095  1.00  0.74
N
ATOM   3807  CZ  ARG A 552      70.877  64.258 273.502  1.00  0.74
C
ATOM   3808  NH1 ARG A 552      71.099  64.497 272.215  1.00  0.74
N
ATOM   3809  NH2 ARG A 552      70.217  65.149 274.238  1.00  0.74
N
ATOM   3810  N   ASN A 553      67.920  60.425 275.729  1.00  0.80
N
ATOM   3811  CA  ASN A 553      67.275  61.157 276.798  1.00  0.80
C
ATOM   3812  C   ASN A 553      66.322  60.273 277.597  1.00  0.80
C
ATOM   3813  O   ASN A 553      65.613  60.759 278.475  1.00  0.80
O
ATOM   3814  CB  ASN A 553      66.465  62.352 276.235  1.00  0.80
C
ATOM   3815  CG  ASN A 553      67.343  63.410 275.569  1.00  0.80
C
ATOM   3816  OD1 ASN A 553      68.519  63.654 275.855  1.00  0.80
O
ATOM   3817  ND2 ASN A 553      66.708  64.144 274.620  1.00  0.80
N
ATOM   3818  N   SER A 554      66.289  58.951 277.335  1.00  0.78
N
ATOM   3819  CA  SER A 554      65.623  57.989 278.198  1.00  0.78
C
ATOM   3820  C   SER A 554      66.624  57.098 278.894  1.00  0.78
C
ATOM   3821  O   SER A 554      66.269  56.281 279.735  1.00  0.78
O
ATOM   3822  CB  SER A 554      64.639  57.076 277.419  1.00  0.78
C
ATOM   3823  OG  SER A 554      65.286  56.358 276.367  1.00  0.78
O
ATOM   3824  N   GLY A 555      67.929  57.275 278.610  1.00  0.82
N
ATOM   3825  CA  GLY A 555      68.969  56.501 279.269  1.00  0.82
C
ATOM   3826  C   GLY A 555      69.191  55.134 278.683  1.00  0.82
C
ATOM   3827  O   GLY A 555      69.924  54.329 279.242  1.00  0.82
O
```

ATOM 3828 N GLU A 556 68.593 54.843 277.519 1.00 0.77
N
ATOM 3829 CA GLU A 556 68.723 53.556 276.873 1.00 0.77
C
ATOM 3830 C GLU A 556 69.952 53.498 275.964 1.00 0.77
C
ATOM 3831 O GLU A 556 69.951 53.965 274.825 1.00 0.77
O
ATOM 3832 CB GLU A 556 67.435 53.225 276.083 1.00 0.77
C
ATOM 3833 CG GLU A 556 66.173 53.060 276.976 1.00 0.77
C
ATOM 3834 CD GLU A 556 66.175 51.826 277.884 1.00 0.77
C
ATOM 3835 OE1 GLU A 556 67.195 51.091 277.924 1.00 0.77
O
ATOM 3836 OE2 GLU A 556 65.128 51.611 278.548 1.00 0.77
O
ATOM 3837 N ASP A 557 71.069 52.943 276.483 1.00 0.77
N
ATOM 3838 CA ASP A 557 72.369 52.945 275.847 1.00 0.77
C
ATOM 3839 C ASP A 557 72.540 51.983 274.672 1.00 0.77
C
ATOM 3840 O ASP A 557 71.665 51.202 274.299 1.00 0.77
O
ATOM 3841 CB ASP A 557 73.511 52.795 276.902 1.00 0.77
C
ATOM 3842 CG ASP A 557 73.738 51.378 277.420 1.00 0.77
C
ATOM 3843 OD1 ASP A 557 72.995 50.451 277.015 1.00 0.77
O
ATOM 3844 OD2 ASP A 557 74.729 51.203 278.166 1.00 0.77
O
ATOM 3845 N PHE A 558 73.740 52.000 274.056 1.00 0.68
N
ATOM 3846 CA PHE A 558 74.065 51.084 272.997 1.00 0.68
C
ATOM 3847 C PHE A 558 74.347 49.658 273.485 1.00 0.68
C
ATOM 3848 O PHE A 558 74.897 48.893 272.723 1.00 0.68
O
ATOM 3849 CB PHE A 558 75.274 51.547 272.136 1.00 0.68
C
ATOM 3850 CG PHE A 558 75.042 52.876 271.451 1.00 0.68
C
ATOM 3851 CD1 PHE A 558 74.223 52.954 270.315 1.00 0.68
C
ATOM 3852 CD2 PHE A 558 75.717 54.039 271.870 1.00 0.68
C
ATOM 3853 CE1 PHE A 558 74.109 54.146 269.588 1.00 0.68
C
ATOM 3854 CE2 PHE A 558 75.609 55.237 271.145 1.00 0.68
C

```
ATOM   3855  CZ  PHE A 558      74.815  55.286 269.992  1.00  0.68
C
ATOM   3856  N   THR A 559      73.974  49.229 274.711  1.00  0.73
N
ATOM   3857  CA  THR A 559      74.032  47.828 275.154  1.00  0.73
C
ATOM   3858  C   THR A 559      72.614  47.375 275.402  1.00  0.73
C
ATOM   3859  O   THR A 559      72.223  46.291 274.971  1.00  0.73
O
ATOM   3860  CB  THR A 559      74.839  47.627 276.428  1.00  0.73
C
ATOM   3861  OG1 THR A 559      76.218  47.861 276.175  1.00  0.73
O
ATOM   3862  CG2 THR A 559      74.740  46.176 276.922  1.00  0.73
C
ATOM   3863  N   ASN A 560      71.767  48.240 276.011  1.00  0.74
N
ATOM   3864  CA  ASN A 560      70.340  48.007 276.158  1.00  0.74
C
ATOM   3865  C   ASN A 560      69.653  47.871 274.808  1.00  0.74
C
ATOM   3866  O   ASN A 560      68.903  46.927 274.576  1.00  0.74
O
ATOM   3867  CB  ASN A 560      69.644  49.159 276.927  1.00  0.74
C
ATOM   3868  CG  ASN A 560      70.098  49.243 278.383  1.00  0.74
C
ATOM   3869  OD1 ASN A 560      70.792  48.391 278.933  1.00  0.74
O
ATOM   3870  ND2 ASN A 560      69.649  50.326 279.061  1.00  0.74
N
ATOM   3871  N   LEU A 561      69.941  48.767 273.838  1.00  0.81
N
ATOM   3872  CA  LEU A 561      69.376  48.685 272.501  1.00  0.81
C
ATOM   3873  C   LEU A 561      69.763  47.408 271.747  1.00  0.81
C
ATOM   3874  O   LEU A 561      68.862  46.740 271.256  1.00  0.81
O
ATOM   3875  CB  LEU A 561      69.657  49.960 271.665  1.00  0.81
C
ATOM   3876  CG  LEU A 561      69.156  51.271 272.293  1.00  0.81
C
ATOM   3877  CD1 LEU A 561      69.821  52.474 271.612  1.00  0.81
C
ATOM   3878  CD2 LEU A 561      67.639  51.413 272.229  1.00  0.81
C
ATOM   3879  N   PRO A 562      71.009  46.939 271.673  1.00  0.80
N
ATOM   3880  CA  PRO A 562      71.301  45.625 271.127  1.00  0.80
C
ATOM   3881  C   PRO A 562      70.697  44.472 271.890  1.00  0.80
C
```

ATOM 3882 O PRO A 562 70.397 43.462 271.258 1.00 0.80
O
ATOM 3883 CB PRO A 562 72.822 45.528 271.064 1.00 0.80
C
ATOM 3884 CG PRO A 562 73.294 46.973 270.977 1.00 0.80
C
ATOM 3885 CD PRO A 562 72.185 47.794 271.637 1.00 0.80
C
ATOM 3886 N GLU A 563 70.523 44.554 273.227 1.00 0.76
N
ATOM 3887 CA GLU A 563 69.807 43.511 273.940 1.00 0.76
C
ATOM 3888 C GLU A 563 68.340 43.457 273.564 1.00 0.76
C
ATOM 3889 O GLU A 563 67.813 42.402 273.215 1.00 0.76
O
ATOM 3890 CB GLU A 563 69.932 43.611 275.477 1.00 0.76
C
ATOM 3891 CG GLU A 563 69.244 42.427 276.211 1.00 0.76
C
ATOM 3892 CD GLU A 563 69.432 42.447 277.739 1.00 0.76
C
ATOM 3893 OE1 GLU A 563 70.181 43.323 278.256 1.00 0.76
O
ATOM 3894 OE2 GLU A 563 68.820 41.520 278.349 1.00 0.76
O
ATOM 3895 N ILE A 564 67.672 44.625 273.534 1.00 0.81
N
ATOM 3896 CA ILE A 564 66.279 44.797 273.171 1.00 0.81
C
ATOM 3897 C ILE A 564 66.022 44.299 271.752 1.00 0.81
C
ATOM 3898 O ILE A 564 65.029 43.631 271.477 1.00 0.81
O
ATOM 3899 CB ILE A 564 65.901 46.262 273.393 1.00 0.81
C
ATOM 3900 CG1 ILE A 564 65.699 46.572 274.891 1.00 0.81
C
ATOM 3901 CG2 ILE A 564 64.649 46.673 272.616 1.00 0.81
C
ATOM 3902 CD1 ILE A 564 65.594 48.072 275.200 1.00 0.81
C
ATOM 3903 N LEU A 565 66.965 44.543 270.828 1.00 0.82
N
ATOM 3904 CA LEU A 565 66.969 44.054 269.460 1.00 0.82
C
ATOM 3905 C LEU A 565 66.869 42.539 269.320 1.00 0.82
C
ATOM 3906 O LEU A 565 66.286 42.020 268.371 1.00 0.82
O
ATOM 3907 CB LEU A 565 68.288 44.517 268.794 1.00 0.82
C
ATOM 3908 CG LEU A 565 68.218 45.802 267.961 1.00 0.82
C

ATOM   3909 CD1 LEU A 565   67.241 46.852 268.443 1.00 0.82
C
ATOM   3910 CD2 LEU A 565   69.601 46.426 267.741 1.00 0.82
C
ATOM   3911 N   LYS A 566   67.455 41.782 270.261 1.00 0.74
N
ATOM   3912 CA LYS A 566   67.506 40.338 270.188 1.00 0.74
C
ATOM   3913 C   LYS A 566   66.342 39.676 270.898 1.00 0.74
C
ATOM   3914 O   LYS A 566   66.222 38.454 270.875 1.00 0.74
O
ATOM   3915 CB LYS A 566   68.833 39.843 270.812 1.00 0.74
C
ATOM   3916 CG LYS A 566   70.074 40.247 269.999 1.00 0.74
C
ATOM   3917 CD LYS A 566   71.384 39.833 270.685 1.00 0.74
C
ATOM   3918 CE LYS A 566   72.631 40.287 269.923 1.00 0.74
C
ATOM   3919 NZ LYS A 566   73.840 39.830 270.643 1.00 0.74
N
ATOM   3920 N   LYS A 567   65.450 40.465 271.532 1.00 0.74
N
ATOM   3921 CA LYS A 567   64.391 39.913 272.355 1.00 0.74
C
ATOM   3922 C   LYS A 567   63.022 40.538 272.119 1.00 0.74
C
ATOM   3923 O   LYS A 567   61.994 39.895 272.317 1.00 0.74
O
ATOM   3924 CB LYS A 567   64.731 40.141 273.851 1.00 0.74
C
ATOM   3925 CG LYS A 567   66.015 39.440 274.312 1.00 0.74
C
ATOM   3926 CD LYS A 567   66.329 39.694 275.790 1.00 0.74
C
ATOM   3927 CE LYS A 567   67.587 38.932 276.229 1.00 0.74
C
ATOM   3928 NZ LYS A 567   67.864 39.204 277.649 1.00 0.74
N
ATOM   3929 N   THR A 568   62.940 41.804 271.682 1.00 0.78
N
ATOM   3930 CA THR A 568   61.659 42.493 271.570 1.00 0.78
C
ATOM   3931 C   THR A 568   61.015 42.274 270.219 1.00 0.78
C
ATOM   3932 O   THR A 568   61.543 42.657 269.180 1.00 0.78
O
ATOM   3933 CB THR A 568   61.774 43.998 271.768 1.00 0.78
C
ATOM   3934 OG1 THR A 568   62.254 44.279 273.075 1.00 0.78
O
ATOM   3935 CG2 THR A 568   60.411 44.705 271.667 1.00 0.78
C

```
ATOM   3936  N   ASN A 569      59.802  41.683 270.194  1.00  0.78
N
ATOM   3937  CA  ASN A 569      58.954  41.652 269.013  1.00  0.78
C
ATOM   3938  C   ASN A 569      58.450  43.061 268.669  1.00  0.78
C
ATOM   3939  O   ASN A 569      57.837  43.744 269.488  1.00  0.78
O
ATOM   3940  CB  ASN A 569      57.796  40.638 269.231  1.00  0.78
C
ATOM   3941  CG  ASN A 569      57.053  40.294 267.940  1.00  0.78
C
ATOM   3942  OD1 ASN A 569      57.102  41.003 266.938  1.00  0.78
O
ATOM   3943  ND2 ASN A 569      56.332  39.147 267.963  1.00  0.78
N
ATOM   3944  N   ILE A 570      58.742  43.551 267.448  1.00  0.80
N
ATOM   3945  CA  ILE A 570      58.423  44.909 267.049  1.00  0.80
C
ATOM   3946  C   ILE A 570      56.959  44.981 266.639  1.00  0.80
C
ATOM   3947  O   ILE A 570      56.472  44.243 265.791  1.00  0.80
O
ATOM   3948  CB  ILE A 570      59.376  45.439 265.978  1.00  0.80
C
ATOM   3949  CG1 ILE A 570      60.818  45.482 266.545  1.00  0.80
C
ATOM   3950  CG2 ILE A 570      58.944  46.846 265.499  1.00  0.80
C
ATOM   3951  CD1 ILE A 570      61.895  45.614 265.464  1.00  0.80
C
ATOM   3952  N   VAL A 571      56.180  45.865 267.279  1.00  0.82
N
ATOM   3953  CA  VAL A 571      54.734  45.854 267.154  1.00  0.82
C
ATOM   3954  C   VAL A 571      54.293  46.906 266.158  1.00  0.82
C
ATOM   3955  O   VAL A 571      54.828  48.011 266.125  1.00  0.82
O
ATOM   3956  CB  VAL A 571      54.075  46.068 268.504  1.00  0.82
C
ATOM   3957  CG1 VAL A 571      52.542  46.105 268.397  1.00  0.82
C
ATOM   3958  CG2 VAL A 571      54.490  44.924 269.451  1.00  0.82
C
ATOM   3959  N   LEU A 572      53.322  46.562 265.284  1.00  0.82
N
ATOM   3960  CA  LEU A 572      53.032  47.340 264.094  1.00  0.82
C
ATOM   3961  C   LEU A 572      51.538  47.501 263.889  1.00  0.82
C
ATOM   3962  O   LEU A 572      51.020  47.499 262.763  1.00  0.82
O
```

ATOM 3963 CB LEU A 572 53.644 46.700 262.830 1.00 0.82
C
ATOM 3964 CG LEU A 572 55.171 46.520 262.865 1.00 0.82
C
ATOM 3965 CD1 LEU A 572 55.640 45.766 261.615 1.00 0.82
C
ATOM 3966 CD2 LEU A 572 55.889 47.872 262.961 1.00 0.82
C
ATOM 3967 N ASP A 573 50.808 47.603 265.008 1.00 0.81
N
ATOM 3968 CA ASP A 573 49.370 47.710 265.040 1.00 0.81
C
ATOM 3969 C ASP A 573 48.865 49.098 264.679 1.00 0.81
C
ATOM 3970 O ASP A 573 47.923 49.231 263.896 1.00 0.81
O
ATOM 3971 CB ASP A 573 48.844 47.254 266.417 1.00 0.81
C
ATOM 3972 CG ASP A 573 49.203 45.789 266.633 1.00 0.81
C
ATOM 3973 OD1 ASP A 573 49.165 45.017 265.638 1.00 0.81
O
ATOM 3974 OD2 ASP A 573 49.548 45.441 267.787 1.00 0.81
O
ATOM 3975 N HIS A 574 49.486 50.183 265.203 1.00 0.78
N
ATOM 3976 CA HIS A 574 49.108 51.541 264.834 1.00 0.78
C
ATOM 3977 C HIS A 574 49.389 51.844 263.367 1.00 0.78
C
ATOM 3978 O HIS A 574 50.342 51.345 262.762 1.00 0.78
O
ATOM 3979 CB HIS A 574 49.735 52.613 265.771 1.00 0.78
C
ATOM 3980 CG HIS A 574 49.139 53.984 265.780 1.00 0.78
C
ATOM 3981 ND1 HIS A 574 49.306 54.841 264.707 1.00 0.78
N
ATOM 3982 CD2 HIS A 574 48.461 54.604 266.777 1.00 0.78
C
ATOM 3983 CE1 HIS A 574 48.716 55.963 265.072 1.00 0.78
C
ATOM 3984 NE2 HIS A 574 48.191 55.874 266.318 1.00 0.78
N
ATOM 3985 N GLU A 575 48.547 52.699 262.756 1.00 0.78
N
ATOM 3986 CA GLU A 575 48.638 53.133 261.377 1.00 0.78
C
ATOM 3987 C GLU A 575 49.999 53.722 261.053 1.00 0.78
C
ATOM 3988 O GLU A 575 50.621 53.398 260.040 1.00 0.78
O
ATOM 3989 CB GLU A 575 47.556 54.225 261.153 1.00 0.78
C

ATOM 3990 CG GLU A 575 47.693 55.092 259.866 1.00 0.78
C
ATOM 3991 CD GLU A 575 46.951 56.435 259.917 1.00 0.78
C
ATOM 3992 OE1 GLU A 575 46.431 56.795 261.001 1.00 0.78
O
ATOM 3993 OE2 GLU A 575 47.025 57.164 258.892 1.00 0.78
O
ATOM 3994 N LYS A 576 50.517 54.597 261.931 1.00 0.81
N
ATOM 3995 CA LYS A 576 51.764 55.275 261.689 1.00 0.81
C
ATOM 3996 C LYS A 576 52.989 54.394 261.845 1.00 0.81
C
ATOM 3997 O LYS A 576 53.906 54.462 261.035 1.00 0.81
O
ATOM 3998 CB LYS A 576 51.873 56.555 262.531 1.00 0.81
C
ATOM 3999 CG LYS A 576 50.753 57.580 262.273 1.00 0.81
C
ATOM 4000 CD LYS A 576 50.596 57.976 260.794 1.00 0.81
C
ATOM 4001 CE LYS A 576 49.737 59.230 260.597 1.00 0.81
C
ATOM 4002 NZ LYS A 576 49.507 59.456 259.164 1.00 0.81
N
ATOM 4003 N GLU A 577 53.031 53.505 262.858 1.00 0.83
N
ATOM 4004 CA GLU A 577 54.083 52.507 262.972 1.00 0.83
C
ATOM 4005 C GLU A 577 54.076 51.502 261.814 1.00 0.83
C
ATOM 4006 O GLU A 577 55.136 51.151 261.303 1.00 0.83
O
ATOM 4007 CB GLU A 577 54.214 51.898 264.403 1.00 0.83
C
ATOM 4008 CG GLU A 577 52.952 51.234 265.003 1.00 0.83
C
ATOM 4009 CD GLU A 577 52.786 51.279 266.534 1.00 0.83
C
ATOM 4010 OE1 GLU A 577 53.514 52.016 267.253 1.00 0.83
O
ATOM 4011 OE2 GLU A 577 51.812 50.636 267.009 1.00 0.83
O
ATOM 4012 N TRP A 578 52.899 51.085 261.285 1.00 0.83
N
ATOM 4013 CA TRP A 578 52.829 50.393 260.001 1.00 0.83
C
ATOM 4014 C TRP A 578 53.341 51.200 258.802 1.00 0.83
C
ATOM 4015 O TRP A 578 54.105 50.677 257.991 1.00 0.83
O
ATOM 4016 CB TRP A 578 51.380 49.912 259.703 1.00 0.83
C

ATOM 4017 CG TRP A 578 51.208 49.067 258.438 1.00 0.83
C
ATOM 4018 CD1 TRP A 578 50.552 49.369 257.275 1.00 0.83
C
ATOM 4019 CD2 TRP A 578 51.780 47.764 258.242 1.00 0.83
C
ATOM 4020 NE1 TRP A 578 50.659 48.327 256.379 1.00 0.83
N
ATOM 4021 CE2 TRP A 578 51.404 47.335 256.953 1.00 0.83
C
ATOM 4022 CE3 TRP A 578 52.568 46.967 259.059 1.00 0.83
C
ATOM 4023 CZ2 TRP A 578 51.788 46.094 256.472 1.00 0.83
C
ATOM 4024 CZ3 TRP A 578 52.981 45.726 258.561 1.00 0.83
C
ATOM 4025 CH2 TRP A 578 52.588 45.288 257.291 1.00 0.83
C
ATOM 4026 N LYS A 579 52.974 52.499 258.664 1.00 0.82
N
ATOM 4027 CA LYS A 579 53.502 53.353 257.603 1.00 0.82
C
ATOM 4028 C LYS A 579 55.019 53.482 257.692 1.00 0.82
C
ATOM 4029 O LYS A 579 55.719 53.324 256.697 1.00 0.82
O
ATOM 4030 CB LYS A 579 52.827 54.761 257.585 1.00 0.82
C
ATOM 4031 CG LYS A 579 53.246 55.688 256.422 1.00 0.82
C
ATOM 4032 CD LYS A 579 52.664 57.114 256.522 1.00 0.82
C
ATOM 4033 CE LYS A 579 53.098 58.007 255.345 1.00 0.82
C
ATOM 4034 NZ LYS A 579 52.574 59.370 255.475 1.00 0.82
N
ATOM 4035 N LEU A 580 55.567 53.699 258.905 1.00 0.88
N
ATOM 4036 CA LEU A 580 56.995 53.770 259.146 1.00 0.88
C
ATOM 4037 C LEU A 580 57.726 52.484 258.832 1.00 0.88
C
ATOM 4038 O LEU A 580 58.722 52.479 258.112 1.00 0.88
O
ATOM 4039 CB LEU A 580 57.251 54.203 260.610 1.00 0.88
C
ATOM 4040 CG LEU A 580 58.723 54.478 260.974 1.00 0.88
C
ATOM 4041 CD1 LEU A 580 59.288 55.673 260.196 1.00 0.88
C
ATOM 4042 CD2 LEU A 580 58.854 54.735 262.481 1.00 0.88
C
ATOM 4043 N ALA A 581 57.199 51.346 259.306 1.00 0.92
N

ATOM	4044	CA	ALA	A	581	57.792	50.047	259.107	1.00	0.92	C
ATOM	4045	C	ALA	A	581	57.951	49.639	257.664	1.00	0.92	C
ATOM	4046	O	ALA	A	581	59.032	49.249	257.226	1.00	0.92	O
ATOM	4047	CB	ALA	A	581	56.877	49.020	259.780	1.00	0.92	C
ATOM	4048	N	LYS	A	582	56.878	49.777	256.866	1.00	0.84	N
ATOM	4049	CA	LYS	A	582	56.951	49.502	255.453	1.00	0.84	C
ATOM	4050	C	LYS	A	582	57.856	50.462	254.703	1.00	0.84	C
ATOM	4051	O	LYS	A	582	58.594	50.043	253.822	1.00	0.84	O
ATOM	4052	CB	LYS	A	582	55.553	49.369	254.814	1.00	0.84	C
ATOM	4053	CG	LYS	A	582	54.768	50.670	254.604	1.00	0.84	C
ATOM	4054	CD	LYS	A	582	53.381	50.340	254.046	1.00	0.84	C
ATOM	4055	CE	LYS	A	582	52.507	51.543	253.709	1.00	0.84	C
ATOM	4056	NZ	LYS	A	582	51.248	51.043	253.131	1.00	0.84	N
ATOM	4057	N	THR	A	583	57.867	51.767	255.059	1.00	0.88	N
ATOM	4058	CA	THR	A	583	58.769	52.755	254.471	1.00	0.88	C
ATOM	4059	C	THR	A	583	60.222	52.431	254.705	1.00	0.88	C
ATOM	4060	O	THR	A	583	61.012	52.441	253.771	1.00	0.88	O
ATOM	4061	CB	THR	A	583	58.477	54.164	254.949	1.00	0.88	C
ATOM	4062	OG1	THR	A	583	57.179	54.539	254.514	1.00	0.88	O
ATOM	4063	CG2	THR	A	583	59.426	55.220	254.368	1.00	0.88	C
ATOM	4064	N	LEU	A	584	60.617	52.034	255.931	1.00	0.91	N
ATOM	4065	CA	LEU	A	584	61.987	51.657	256.240	1.00	0.91	C
ATOM	4066	C	LEU	A	584	62.498	50.507	255.387	1.00	0.91	C
ATOM	4067	O	LEU	A	584	63.649	50.485	254.949	1.00	0.91	O
ATOM	4068	CB	LEU	A	584	62.155	51.278	257.730	1.00	0.91	C
ATOM	4069	CG	LEU	A	584	61.978	52.441	258.727	1.00	0.91	C
ATOM	4070	CD1	LEU	A	584	62.178	51.938	260.163	1.00	0.91	C

ATOM 4071 CD2 LEU A 584 62.916 53.621 258.441 1.00 0.91
C
ATOM 4072 N LEU A 585 61.615 49.547 255.064 1.00 0.89
N
ATOM 4073 CA LEU A 585 61.991 48.370 254.317 1.00 0.89
C
ATOM 4074 C LEU A 585 61.972 48.590 252.810 1.00 0.89
C
ATOM 4075 O LEU A 585 62.319 47.699 252.036 1.00 0.89
O
ATOM 4076 CB LEU A 585 61.054 47.203 254.684 1.00 0.89
C
ATOM 4077 CG LEU A 585 61.155 46.753 256.155 1.00 0.89
C
ATOM 4078 CD1 LEU A 585 60.169 45.608 256.412 1.00 0.89
C
ATOM 4079 CD2 LEU A 585 62.580 46.343 256.564 1.00 0.89
C
ATOM 4080 N LYS A 586 61.662 49.820 252.349 1.00 0.84
N
ATOM 4081 CA LYS A 586 61.763 50.182 250.948 1.00 0.84
C
ATOM 4082 C LYS A 586 63.179 50.574 250.551 1.00 0.84
C
ATOM 4083 O LYS A 586 63.470 50.709 249.367 1.00 0.84
O
ATOM 4084 CB LYS A 586 60.853 51.376 250.570 1.00 0.84
C
ATOM 4085 CG LYS A 586 59.354 51.120 250.742 1.00 0.84
C
ATOM 4086 CD LYS A 586 58.511 52.383 250.501 1.00 0.84
C
ATOM 4087 CE LYS A 586 57.046 52.211 250.906 1.00 0.84
C
ATOM 4088 NZ LYS A 586 56.242 53.321 250.351 1.00 0.84
N
ATOM 4089 N LEU A 587 64.123 50.736 251.511 1.00 0.88
N
ATOM 4090 CA LEU A 587 65.473 51.221 251.238 1.00 0.88
C
ATOM 4091 C LEU A 587 66.208 50.468 250.133 1.00 0.88
C
ATOM 4092 O LEU A 587 66.799 51.068 249.242 1.00 0.88
O
ATOM 4093 CB LEU A 587 66.326 51.170 252.537 1.00 0.88
C
ATOM 4094 CG LEU A 587 67.810 51.594 252.391 1.00 0.88
C
ATOM 4095 CD1 LEU A 587 67.968 53.074 252.013 1.00 0.88
C
ATOM 4096 CD2 LEU A 587 68.613 51.303 253.666 1.00 0.88
C
ATOM 4097 N HIS A 588 66.148 49.122 250.128 1.00 0.83
N

ATOM 4098 CA HIS A 588 66.791 48.294 249.120 1.00 0.83
C
ATOM 4099 C HIS A 588 66.247 48.526 247.707 1.00 0.83
C
ATOM 4100 O HIS A 588 67.012 48.649 246.755 1.00 0.83
O
ATOM 4101 CB HIS A 588 66.665 46.818 249.562 1.00 0.83
C
ATOM 4102 CG HIS A 588 67.484 45.803 248.821 1.00 0.83
C
ATOM 4103 ND1 HIS A 588 67.573 44.543 249.375 1.00 0.83
N
ATOM 4104 CD2 HIS A 588 68.040 45.809 247.582 1.00 0.83
C
ATOM 4105 CE1 HIS A 588 68.171 43.807 248.460 1.00 0.83
C
ATOM 4106 NE2 HIS A 588 68.477 44.523 247.355 1.00 0.83
N
ATOM 4107 N ASP A 589 64.912 48.657 247.568 1.00 0.85
N
ATOM 4108 CA ASP A 589 64.195 48.923 246.333 1.00 0.85
C
ATOM 4109 C ASP A 589 64.585 50.275 245.715 1.00 0.85
C
ATOM 4110 O ASP A 589 64.849 50.402 244.519 1.00 0.85
O
ATOM 4111 CB ASP A 589 62.691 48.762 246.670 1.00 0.85
C
ATOM 4112 CG ASP A 589 61.748 49.007 245.507 1.00 0.85
C
ATOM 4113 OD1 ASP A 589 60.683 49.624 245.759 1.00 0.85
O
ATOM 4114 OD2 ASP A 589 62.008 48.505 244.387 1.00 0.85
O
ATOM 4115 N ILE A 590 64.731 51.321 246.552 1.00 0.85
N
ATOM 4116 CA ILE A 590 65.241 52.615 246.122 1.00 0.85
C
ATOM 4117 C ILE A 590 66.670 52.543 245.577 1.00 0.85
C
ATOM 4118 O ILE A 590 66.970 53.038 244.490 1.00 0.85
O
ATOM 4119 CB ILE A 590 65.173 53.607 247.281 1.00 0.85
C
ATOM 4120 CG1 ILE A 590 63.724 53.777 247.805 1.00 0.85
C
ATOM 4121 CG2 ILE A 590 65.773 54.977 246.895 1.00 0.85
C
ATOM 4122 CD1 ILE A 590 62.724 54.337 246.787 1.00 0.85
C
ATOM 4123 N LEU A 591 67.593 51.867 246.294 1.00 0.86
N
ATOM 4124 CA LEU A 591 68.977 51.719 245.867 1.00 0.86
C

ATOM	4125	C	LEU	A	591	69.164	50.903	244.594	1.00	0.86	C
ATOM	4126	O	LEU	A	591	69.970	51.254	243.731	1.00	0.86	O
ATOM	4127	CB	LEU	A	591	69.863	51.110	246.980	1.00	0.86	C
ATOM	4128	CG	LEU	A	591	69.891	51.888	248.313	1.00	0.86	C
ATOM	4129	CD1	LEU	A	591	70.830	51.191	249.305	1.00	0.86	C
ATOM	4130	CD2	LEU	A	591	70.293	53.361	248.160	1.00	0.86	C
ATOM	4131	N	ILE	A	592	68.419	49.791	244.425	1.00	0.83	N
ATOM	4132	CA	ILE	A	592	68.480	48.989	243.213	1.00	0.83	C
ATOM	4133	C	ILE	A	592	67.953	49.723	241.986	1.00	0.83	C
ATOM	4134	O	ILE	A	592	68.465	49.577	240.880	1.00	0.83	O
ATOM	4135	CB	ILE	A	592	67.855	47.609	243.386	1.00	0.83	C
ATOM	4136	CG1	ILE	A	592	68.283	46.614	242.288	1.00	0.83	C
ATOM	4137	CG2	ILE	A	592	66.322	47.679	243.493	1.00	0.83	C
ATOM	4138	CD1	ILE	A	592	69.743	46.156	242.388	1.00	0.83	C
ATOM	4139	N	LYS	A	593	66.929	50.594	242.126	1.00	0.81	N
ATOM	4140	CA	LYS	A	593	66.518	51.459	241.036	1.00	0.81	C
ATOM	4141	C	LYS	A	593	67.588	52.472	240.657	1.00	0.81	C
ATOM	4142	O	LYS	A	593	67.843	52.720	239.476	1.00	0.81	O
ATOM	4143	CB	LYS	A	593	65.162	52.149	241.319	1.00	0.81	C
ATOM	4144	CG	LYS	A	593	64.570	52.918	240.119	1.00	0.81	C
ATOM	4145	CD	LYS	A	593	64.192	52.016	238.928	1.00	0.81	C
ATOM	4146	CE	LYS	A	593	64.287	52.695	237.562	1.00	0.81	C
ATOM	4147	NZ	LYS	A	593	63.408	53.874	237.460	1.00	0.81	N
ATOM	4148	N	CYS	A	594	68.295	53.040	241.653	1.00	0.87	N
ATOM	4149	CA	CYS	A	594	69.397	53.953	241.413	1.00	0.87	C
ATOM	4150	C	CYS	A	594	70.597	53.330	240.722	1.00	0.87	C
ATOM	4151	O	CYS	A	594	71.326	54.024	240.026	1.00	0.87	O

ATOM 4152 CB CYS A 594 69.862 54.674 242.696 1.00 0.87
C
ATOM 4153 SG CYS A 594 68.514 55.695 243.386 1.00 0.87
S
ATOM 4154 N SER A 595 70.829 52.007 240.829 1.00 0.85
N
ATOM 4155 CA SER A 595 71.892 51.350 240.072 1.00 0.85
C
ATOM 4156 C SER A 595 71.611 51.289 238.575 1.00 0.85
C
ATOM 4157 O SER A 595 72.515 51.204 237.738 1.00 0.85
O
ATOM 4158 CB SER A 595 72.197 49.919 240.601 1.00 0.85
C
ATOM 4159 OG SER A 595 71.213 48.955 240.220 1.00 0.85
O
ATOM 4160 N LYS A 596 70.318 51.331 238.203 1.00 0.80
N
ATOM 4161 CA LYS A 596 69.882 51.309 236.829 1.00 0.80
C
ATOM 4162 C LYS A 596 69.957 52.676 236.161 1.00 0.80
C
ATOM 4163 O LYS A 596 70.560 52.798 235.101 1.00 0.80
O
ATOM 4164 CB LYS A 596 68.457 50.704 236.735 1.00 0.80
C
ATOM 4165 CG LYS A 596 68.401 49.248 237.243 1.00 0.80
C
ATOM 4166 CD LYS A 596 67.003 48.601 237.166 1.00 0.80
C
ATOM 4167 CE LYS A 596 66.999 47.148 237.674 1.00 0.80
C
ATOM 4168 NZ LYS A 596 65.640 46.551 237.622 1.00 0.80
N
ATOM 4169 N GLU A 597 69.403 53.745 236.772 1.00 0.82
N
ATOM 4170 CA GLU A 597 69.340 55.037 236.095 1.00 0.82
C
ATOM 4171 C GLU A 597 70.339 56.051 236.617 1.00 0.82
C
ATOM 4172 O GLU A 597 70.502 57.144 236.069 1.00 0.82
O
ATOM 4173 CB GLU A 597 67.930 55.639 236.250 1.00 0.82
C
ATOM 4174 CG GLU A 597 66.858 54.832 235.491 1.00 0.82
C
ATOM 4175 CD GLU A 597 65.453 55.232 235.886 1.00 0.82
C
ATOM 4176 OE1 GLU A 597 64.496 54.805 235.186 1.00 0.82
O
ATOM 4177 OE2 GLU A 597 65.254 55.796 236.989 1.00 0.82
O
ATOM 4178 N LEU A 598 71.052 55.723 237.711 1.00 0.87
N

ATOM 4179 CA LEU A 598 72.058 56.573 238.327 1.00 0.87
C
ATOM 4180 C LEU A 598 71.511 57.948 238.701 1.00 0.87
C
ATOM 4181 O LEU A 598 72.174 58.978 238.591 1.00 0.87
O
ATOM 4182 CB LEU A 598 73.381 56.597 237.510 1.00 0.87
C
ATOM 4183 CG LEU A 598 73.928 55.189 237.158 1.00 0.87
C
ATOM 4184 CD1 LEU A 598 75.107 55.251 236.178 1.00 0.87
C
ATOM 4185 CD2 LEU A 598 74.335 54.372 238.394 1.00 0.87
C
ATOM 4186 N PHE A 599 70.246 57.974 239.165 1.00 0.85
N
ATOM 4187 CA PHE A 599 69.560 59.151 239.652 1.00 0.85
C
ATOM 4188 C PHE A 599 69.493 59.126 241.168 1.00 0.85
C
ATOM 4189 O PHE A 599 68.586 58.564 241.775 1.00 0.85
O
ATOM 4190 CB PHE A 599 68.106 59.223 239.108 1.00 0.85
C
ATOM 4191 CG PHE A 599 68.018 59.638 237.663 1.00 0.85
C
ATOM 4192 CD1 PHE A 599 68.790 60.698 237.156 1.00 0.85
C
ATOM 4193 CD2 PHE A 599 67.108 59.002 236.801 1.00 0.85
C
ATOM 4194 CE1 PHE A 599 68.721 61.055 235.805 1.00 0.85
C
ATOM 4195 CE2 PHE A 599 67.029 59.365 235.450 1.00 0.85
C
ATOM 4196 CZ PHE A 599 67.847 60.380 234.948 1.00 0.85
C
ATOM 4197 N LEU A 600 70.455 59.792 241.832 1.00 0.88
N
ATOM 4198 CA LEU A 600 70.598 59.783 243.275 1.00 0.88
C
ATOM 4199 C LEU A 600 69.561 60.615 244.002 1.00 0.88
C
ATOM 4200 O LEU A 600 69.346 60.457 245.202 1.00 0.88
O
ATOM 4201 CB LEU A 600 71.999 60.315 243.630 1.00 0.88
C
ATOM 4202 CG LEU A 600 73.126 59.334 243.261 1.00 0.88
C
ATOM 4203 CD1 LEU A 600 74.458 60.086 243.174 1.00 0.88
C
ATOM 4204 CD2 LEU A 600 73.212 58.174 244.268 1.00 0.88
C
ATOM 4205 N HIS A 601 68.842 61.492 243.285 1.00 0.84
N

ATOM 4206 CA HIS A 601 67.807 62.325 243.854 1.00 0.84
C
ATOM 4207 C HIS A 601 66.648 61.548 244.437 1.00 0.84
C
ATOM 4208 O HIS A 601 66.057 61.955 245.429 1.00 0.84
O
ATOM 4209 CB HIS A 601 67.322 63.382 242.846 1.00 0.84
C
ATOM 4210 CG HIS A 601 66.559 62.866 241.674 1.00 0.84
C
ATOM 4211 ND1 HIS A 601 67.220 62.393 240.553 1.00 0.84
N
ATOM 4212 CD2 HIS A 601 65.217 62.755 241.517 1.00 0.84
C
ATOM 4213 CE1 HIS A 601 66.257 62.007 239.737 1.00 0.84
C
ATOM 4214 NE2 HIS A 601 65.030 62.201 240.273 1.00 0.84
N
ATOM 4215 N PHE A 602 66.324 60.365 243.883 1.00 0.85
N
ATOM 4216 CA PHE A 602 65.328 59.490 244.474 1.00 0.85
C
ATOM 4217 C PHE A 602 65.746 58.903 245.831 1.00 0.85
C
ATOM 4218 O PHE A 602 64.908 58.632 246.687 1.00 0.85
O
ATOM 4219 CB PHE A 602 64.916 58.379 243.475 1.00 0.85
C
ATOM 4220 CG PHE A 602 64.222 58.949 242.256 1.00 0.85
C
ATOM 4221 CD1 PHE A 602 63.135 59.831 242.385 1.00 0.85
C
ATOM 4222 CD2 PHE A 602 64.614 58.559 240.962 1.00 0.85
C
ATOM 4223 CE1 PHE A 602 62.459 60.314 241.258 1.00 0.85
C
ATOM 4224 CE2 PHE A 602 63.933 59.028 239.829 1.00 0.85
C
ATOM 4225 CZ PHE A 602 62.852 59.907 239.979 1.00 0.85
C
ATOM 4226 N LEU A 603 67.064 58.736 246.105 1.00 0.89
N
ATOM 4227 CA LEU A 603 67.561 58.431 247.444 1.00 0.89
C
ATOM 4228 C LEU A 603 67.353 59.593 248.404 1.00 0.89
C
ATOM 4229 O LEU A 603 66.939 59.422 249.549 1.00 0.89
O
ATOM 4230 CB LEU A 603 69.056 58.026 247.427 1.00 0.89
C
ATOM 4231 CG LEU A 603 69.699 57.782 248.811 1.00 0.89
C
ATOM 4232 CD1 LEU A 603 69.009 56.662 249.604 1.00 0.89
C

ATOM 4233 CD2 LEU A 603 71.197 57.490 248.660 1.00 0.89
C
ATOM 4234 N CYS A 604 67.605 60.830 247.942 1.00 0.88
N
ATOM 4235 CA CYS A 604 67.308 62.029 248.705 1.00 0.88
C
ATOM 4236 C CYS A 604 65.828 62.212 248.999 1.00 0.88
C
ATOM 4237 O CYS A 604 65.436 62.466 250.135 1.00 0.88
O
ATOM 4238 CB CYS A 604 67.853 63.270 247.978 1.00 0.88
C
ATOM 4239 SG CYS A 604 69.666 63.163 247.811 1.00 0.88
S
ATOM 4240 N GLU A 605 64.953 61.995 247.995 1.00 0.84
N
ATOM 4241 CA GLU A 605 63.510 62.020 248.163 1.00 0.84
C
ATOM 4242 C GLU A 605 63.047 61.008 249.198 1.00 0.84
C
ATOM 4243 O GLU A 605 62.275 61.328 250.097 1.00 0.84
O
ATOM 4244 CB GLU A 605 62.812 61.777 246.805 1.00 0.84
C
ATOM 4245 CG GLU A 605 61.271 61.925 246.833 1.00 0.84
C
ATOM 4246 CD GLU A 605 60.651 61.909 245.434 1.00 0.84
C
ATOM 4247 OE1 GLU A 605 59.402 62.023 245.365 1.00 0.84
O
ATOM 4248 OE2 GLU A 605 61.406 61.806 244.433 1.00 0.84
O
ATOM 4249 N PHE A 606 63.611 59.785 249.160 1.00 0.87
N
ATOM 4250 CA PHE A 606 63.406 58.761 250.163 1.00 0.87
C
ATOM 4251 C PHE A 606 63.888 59.131 251.566 1.00 0.87
C
ATOM 4252 O PHE A 606 63.198 58.894 252.550 1.00 0.87
O
ATOM 4253 CB PHE A 606 64.061 57.453 249.664 1.00 0.87
C
ATOM 4254 CG PHE A 606 63.750 56.299 250.566 1.00 0.87
C
ATOM 4255 CD1 PHE A 606 64.750 55.740 251.376 1.00 0.87
C
ATOM 4256 CD2 PHE A 606 62.444 55.797 250.638 1.00 0.87
C
ATOM 4257 CE1 PHE A 606 64.450 54.685 252.242 1.00 0.87
C
ATOM 4258 CE2 PHE A 606 62.139 54.743 251.502 1.00 0.87
C
ATOM 4259 CZ PHE A 606 63.146 54.186 252.299 1.00 0.87
C

```
ATOM   4260  N   CYS A 607      65.065  59.764 251.727  1.00  0.89
N
ATOM   4261  CA  CYS A 607      65.500  60.255 253.027  1.00  0.89
C
ATOM   4262  C   CYS A 607      64.536  61.271 253.614  1.00  0.89
C
ATOM   4263  O   CYS A 607      64.178  61.202 254.788  1.00  0.89
O
ATOM   4264  CB  CYS A 607      66.917  60.877 252.946  1.00  0.89
C
ATOM   4265  SG  CYS A 607      68.180  59.874 253.781  1.00  0.89
S
ATOM   4266  N   PHE A 608      64.047  62.208 252.776  1.00  0.87
N
ATOM   4267  CA  PHE A 608      63.005  63.139 253.148  1.00  0.87
C
ATOM   4268  C   PHE A 608      61.682  62.453 253.478  1.00  0.87
C
ATOM   4269  O   PHE A 608      61.064  62.770 254.496  1.00  0.87
O
ATOM   4270  CB  PHE A 608      62.857  64.203 252.034  1.00  0.87
C
ATOM   4271  CG  PHE A 608      61.895  65.287 252.425  1.00  0.87
C
ATOM   4272  CD1 PHE A 608      60.579  65.260 251.942  1.00  0.87
C
ATOM   4273  CD2 PHE A 608      62.274  66.298 253.322  1.00  0.87
C
ATOM   4274  CE1 PHE A 608      59.644  66.210 252.368  1.00  0.87
C
ATOM   4275  CE2 PHE A 608      61.341  67.249 253.754  1.00  0.87
C
ATOM   4276  CZ  PHE A 608      60.023  67.201 253.282  1.00  0.87
C
ATOM   4277  N   GLU A 609      61.247  61.454 252.671  1.00  0.86
N
ATOM   4278  CA  GLU A 609      60.063  60.660 252.955  1.00  0.86
C
ATOM   4279  C   GLU A 609      60.179  59.987 254.315  1.00  0.86
C
ATOM   4280  O   GLU A 609      59.352  60.213 255.185  1.00  0.86
O
ATOM   4281  CB  GLU A 609      59.780  59.605 251.844  1.00  0.86
C
ATOM   4282  CG  GLU A 609      58.421  58.849 251.980  1.00  0.86
C
ATOM   4283  CD  GLU A 609      58.316  57.534 251.185  1.00  0.86
C
ATOM   4284  OE1 GLU A 609      59.270  57.183 250.451  1.00  0.86
O
ATOM   4285  OE2 GLU A 609      57.277  56.830 251.349  1.00  0.86
O
ATOM   4286  N   VAL A 610      61.286  59.267 254.593  1.00  0.90
N
```

```
ATOM   4287  CA  VAL A 610      61.531  58.542 255.833  1.00  0.90
C
ATOM   4288  C   VAL A 610      61.448  59.443 257.055  1.00  0.90
C
ATOM   4289  O   VAL A 610      60.812  59.109 258.056  1.00  0.90
O
ATOM   4290  CB  VAL A 610      62.903  57.877 255.754  1.00  0.90
C
ATOM   4291  CG1 VAL A 610      63.403  57.357 257.113  1.00  0.90
C
ATOM   4292  CG2 VAL A 610      62.877  56.696 254.766  1.00  0.90
C
ATOM   4293  N   CYS A 611      62.041  60.650 256.977  1.00  0.90
N
ATOM   4294  CA  CYS A 611      61.937  61.654 258.021  1.00  0.90
C
ATOM   4295  C   CYS A 611      60.525  62.159 258.269  1.00  0.90
C
ATOM   4296  O   CYS A 611      60.089  62.246 259.414  1.00  0.90
O
ATOM   4297  CB  CYS A 611      62.885  62.840 257.737  1.00  0.90
C
ATOM   4298  SG  CYS A 611      64.617  62.359 258.069  1.00  0.90
S
ATOM   4299  N   THR A 612      59.745  62.453 257.212  1.00  0.87
N
ATOM   4300  CA  THR A 612      58.328  62.815 257.334  1.00  0.87
C
ATOM   4301  C   THR A 612      57.499  61.701 257.939  1.00  0.87
C
ATOM   4302  O   THR A 612      56.671  61.925 258.822  1.00  0.87
O
ATOM   4303  CB  THR A 612      57.717  63.208 255.994  1.00  0.87
C
ATOM   4304  OG1 THR A 612      58.262  64.452 255.579  1.00  0.87
O
ATOM   4305  CG2 THR A 612      56.192  63.399 256.060  1.00  0.87
C
ATOM   4306  N   VAL A 613      57.739  60.441 257.516  1.00  0.88
N
ATOM   4307  CA  VAL A 613      57.074  59.278 258.084  1.00  0.88
C
ATOM   4308  C   VAL A 613      57.385  59.112 259.564  1.00  0.88
C
ATOM   4309  O   VAL A 613      56.501  58.821 260.370  1.00  0.88
O
ATOM   4310  CB  VAL A 613      57.379  57.977 257.344  1.00  0.88
C
ATOM   4311  CG1 VAL A 613      56.507  56.844 257.897  1.00  0.88
C
ATOM   4312  CG2 VAL A 613      57.075  58.110 255.846  1.00  0.88
C
ATOM   4313  N   PHE A 614      58.651  59.343 259.975  1.00  0.87
N
```

ATOM 4314 CA PHE A 614 59.040 59.345 261.371 1.00 0.87
C
ATOM 4315 C PHE A 614 58.298 60.397 262.205 1.00 0.87
C
ATOM 4316 O PHE A 614 57.774 60.079 263.270 1.00 0.87
O
ATOM 4317 CB PHE A 614 60.588 59.494 261.476 1.00 0.87
C
ATOM 4318 CG PHE A 614 61.027 59.638 262.906 1.00 0.87
C
ATOM 4319 CD1 PHE A 614 61.050 58.534 263.770 1.00 0.87
C
ATOM 4320 CD2 PHE A 614 61.240 60.925 263.427 1.00 0.87
C
ATOM 4321 CE1 PHE A 614 61.224 58.718 265.147 1.00 0.87
C
ATOM 4322 CE2 PHE A 614 61.399 61.113 264.802 1.00 0.87
C
ATOM 4323 CZ PHE A 614 61.376 60.011 265.660 1.00 0.87
C
ATOM 4324 N THR A 615 58.194 61.652 261.726 1.00 0.86
N
ATOM 4325 CA THR A 615 57.466 62.719 262.417 1.00 0.86
C
ATOM 4326 C THR A 615 55.998 62.389 262.606 1.00 0.86
C
ATOM 4327 O THR A 615 55.462 62.504 263.703 1.00 0.86
O
ATOM 4328 CB THR A 615 57.609 64.056 261.704 1.00 0.86
C
ATOM 4329 OG1 THR A 615 58.952 64.509 261.813 1.00 0.86
O
ATOM 4330 CG2 THR A 615 56.728 65.146 262.326 1.00 0.86
C
ATOM 4331 N GLU A 616 55.327 61.852 261.564 1.00 0.82
N
ATOM 4332 CA GLU A 616 53.959 61.365 261.679 1.00 0.82
C
ATOM 4333 C GLU A 616 53.779 60.270 262.728 1.00 0.82
C
ATOM 4334 O GLU A 616 52.751 60.195 263.401 1.00 0.82
O
ATOM 4335 CB GLU A 616 53.459 60.753 260.358 1.00 0.82
C
ATOM 4336 CG GLU A 616 53.192 61.752 259.228 1.00 0.82
C
ATOM 4337 CD GLU A 616 52.624 61.039 258.042 1.00 0.82
C
ATOM 4338 OE1 GLU A 616 51.791 60.098 258.125 1.00 0.82
O
ATOM 4339 OE2 GLU A 616 53.071 61.414 256.937 1.00 0.82
O
ATOM 4340 N PHE A 617 54.769 59.361 262.864 1.00 0.84
N

```
ATOM   4341  CA  PHE A 617      54.802  58.363 263.917  1.00  0.84
C
ATOM   4342  C   PHE A 617      54.953  58.970 265.302  1.00  0.84
C
ATOM   4343  O   PHE A 617      54.145  58.691 266.184  1.00  0.84
O
ATOM   4344  CB  PHE A 617      55.917  57.321 263.581  1.00  0.84
C
ATOM   4345  CG  PHE A 617      56.373  56.460 264.736  1.00  0.84
C
ATOM   4346  CD1 PHE A 617      55.574  55.423 265.239  1.00  0.84
C
ATOM   4347  CD2 PHE A 617      57.618  56.710 265.338  1.00  0.84
C
ATOM   4348  CE1 PHE A 617      56.007  54.658 266.331  1.00  0.84
C
ATOM   4349  CE2 PHE A 617      58.059  55.942 266.420  1.00  0.84
C
ATOM   4350  CZ  PHE A 617      57.249  54.919 266.920  1.00  0.84
C
ATOM   4351  N   TYR A 618      55.947  59.850 265.510  1.00  0.82
N
ATOM   4352  CA  TYR A 618      56.280  60.413 266.806  1.00  0.82
C
ATOM   4353  C   TYR A 618      55.139  61.238 267.391  1.00  0.82
C
ATOM   4354  O   TYR A 618      54.794  61.107 268.564  1.00  0.82
O
ATOM   4355  CB  TYR A 618      57.590  61.225 266.641  1.00  0.82
C
ATOM   4356  CG  TYR A 618      58.165  61.698 267.945  1.00  0.82
C
ATOM   4357  CD1 TYR A 618      58.770  60.799 268.839  1.00  0.82
C
ATOM   4358  CD2 TYR A 618      58.122  63.062 268.274  1.00  0.82
C
ATOM   4359  CE1 TYR A 618      59.317  61.260 270.047  1.00  0.82
C
ATOM   4360  CE2 TYR A 618      58.677  63.523 269.474  1.00  0.82
C
ATOM   4361  CZ  TYR A 618      59.274  62.621 270.361  1.00  0.82
C
ATOM   4362  OH  TYR A 618      59.836  63.101 271.560  1.00  0.82
O
ATOM   4363  N   ASP A 619      54.465  62.025 266.535  1.00  0.81
N
ATOM   4364  CA  ASP A 619      53.367  62.888 266.907  1.00  0.81
C
ATOM   4365  C   ASP A 619      52.067  62.126 267.221  1.00  0.81
C
ATOM   4366  O   ASP A 619      51.133  62.681 267.795  1.00  0.81
O
ATOM   4367  CB  ASP A 619      53.146  63.936 265.783  1.00  0.81
C
```

ATOM 4368 CG ASP A 619 54.315 64.913 265.660 1.00 0.81
C
ATOM 4369 OD1 ASP A 619 55.218 64.919 266.536 1.00 0.81
O
ATOM 4370 OD2 ASP A 619 54.295 65.694 264.672 1.00 0.81
O
ATOM 4371 N SER A 620 51.982 60.816 266.881 1.00 0.82
N
ATOM 4372 CA SER A 620 50.832 59.977 267.225 1.00 0.82
C
ATOM 4373 C SER A 620 51.151 58.899 268.252 1.00 0.82
C
ATOM 4374 O SER A 620 50.243 58.269 268.796 1.00 0.82
O
ATOM 4375 CB SER A 620 50.299 59.175 266.002 1.00 0.82
C
ATOM 4376 OG SER A 620 49.600 59.994 265.068 1.00 0.82
O
ATOM 4377 N CYS A 621 52.437 58.627 268.548 1.00 0.85
N
ATOM 4378 CA CYS A 621 52.822 57.472 269.343 1.00 0.85
C
ATOM 4379 C CYS A 621 53.917 57.779 270.348 1.00 0.85
C
ATOM 4380 O CYS A 621 55.084 57.915 269.998 1.00 0.85
O
ATOM 4381 CB CYS A 621 53.382 56.333 268.445 1.00 0.85
C
ATOM 4382 SG CYS A 621 52.116 55.515 267.418 1.00 0.85
S
ATOM 4383 N TYR A 622 53.599 57.800 271.663 1.00 0.75
N
ATOM 4384 CA TYR A 622 54.621 57.924 272.694 1.00 0.75
C
ATOM 4385 C TYR A 622 55.640 56.784 272.703 1.00 0.75
C
ATOM 4386 O TYR A 622 55.288 55.602 272.780 1.00 0.75
O
ATOM 4387 CB TYR A 622 54.043 57.991 274.133 1.00 0.75
C
ATOM 4388 CG TYR A 622 53.028 59.083 274.285 1.00 0.75
C
ATOM 4389 CD1 TYR A 622 53.426 60.427 274.351 1.00 0.75
C
ATOM 4390 CD2 TYR A 622 51.664 58.769 274.400 1.00 0.75
C
ATOM 4391 CE1 TYR A 622 52.472 61.442 274.510 1.00 0.75
C
ATOM 4392 CE2 TYR A 622 50.710 59.783 274.556 1.00 0.75
C
ATOM 4393 CZ TYR A 622 51.116 61.119 274.609 1.00 0.75
C
ATOM 4394 OH TYR A 622 50.161 62.136 274.790 1.00 0.75
O

```
ATOM   4395  N   CYS A 623      56.942  57.134 272.640  1.00  0.81
N
ATOM   4396  CA  CYS A 623      58.043  56.208 272.857  1.00  0.81
C
ATOM   4397  C   CYS A 623      58.352  56.059 274.333  1.00  0.81
C
ATOM   4398  O   CYS A 623      58.709  54.984 274.812  1.00  0.81
O
ATOM   4399  CB  CYS A 623      59.323  56.652 272.110  1.00  0.81
C
ATOM   4400  SG  CYS A 623      59.010  56.819 270.318  1.00  0.81
S
ATOM   4401  N   ILE A 624      58.173  57.158 275.087  1.00  0.72
N
ATOM   4402  CA  ILE A 624      58.296  57.184 276.527  1.00  0.72
C
ATOM   4403  C   ILE A 624      57.138  57.983 277.083  1.00  0.72
C
ATOM   4404  O   ILE A 624      56.760  59.024 276.542  1.00  0.72
O
ATOM   4405  CB  ILE A 624      59.599  57.790 277.069  1.00  0.72
C
ATOM   4406  CG1 ILE A 624      60.013  59.099 276.354  1.00  0.72
C
ATOM   4407  CG2 ILE A 624      60.714  56.730 277.015  1.00  0.72
C
ATOM   4408  CD1 ILE A 624      61.240  59.774 276.982  1.00  0.72
C
ATOM   4409  N   GLU A 625      56.555  57.500 278.192  1.00  0.61
N
ATOM   4410  CA  GLU A 625      55.571  58.219 278.973  1.00  0.61
C
ATOM   4411  C   GLU A 625      56.281  58.724 280.212  1.00  0.61
C
ATOM   4412  O   GLU A 625      57.094  58.023 280.810  1.00  0.61
O
ATOM   4413  CB  GLU A 625      54.353  57.347 279.356  1.00  0.61
C
ATOM   4414  CG  GLU A 625      53.547  56.878 278.119  1.00  0.61
C
ATOM   4415  CD  GLU A 625      52.303  56.070 278.489  1.00  0.61
C
ATOM   4416  OE1 GLU A 625      52.431  55.135 279.317  1.00  0.61
O
ATOM   4417  OE2 GLU A 625      51.231  56.367 277.902  1.00  0.61
O
ATOM   4418  N   LYS A 626      56.046  60.002 280.578  1.00  0.59
N
ATOM   4419  CA  LYS A 626      56.779  60.648 281.652  1.00  0.59
C
ATOM   4420  C   LYS A 626      55.888  61.033 282.820  1.00  0.59
C
ATOM   4421  O   LYS A 626      56.340  61.566 283.831  1.00  0.59
O
```

ATOM 4422 CB LYS A 626 57.500 61.907 281.133 1.00 0.59
C
ATOM 4423 CG LYS A 626 58.472 61.647 279.971 1.00 0.59
C
ATOM 4424 CD LYS A 626 59.151 62.952 279.523 1.00 0.59
C
ATOM 4425 CE LYS A 626 60.037 62.804 278.285 1.00 0.59
C
ATOM 4426 NZ LYS A 626 60.454 64.138 277.802 1.00 0.59
N
ATOM 4427 N ASN A 627 54.576 60.748 282.705 1.00 0.60
N
ATOM 4428 CA ASN A 627 53.539 61.070 283.669 1.00 0.60
C
ATOM 4429 C ASN A 627 53.589 62.464 284.308 1.00 0.60
C
ATOM 4430 O ASN A 627 54.059 63.439 283.731 1.00 0.60
O
ATOM 4431 CB ASN A 627 53.480 59.952 284.743 1.00 0.60
C
ATOM 4432 CG ASN A 627 53.210 58.621 284.047 1.00 0.60
C
ATOM 4433 OD1 ASN A 627 52.285 58.528 283.245 1.00 0.60
O
ATOM 4434 ND2 ASN A 627 54.007 57.576 284.358 1.00 0.60
N
ATOM 4435 N LYS A 628 53.061 62.576 285.541 1.00 0.61
N
ATOM 4436 CA LYS A 628 52.944 63.796 286.317 1.00 0.61
C
ATOM 4437 C LYS A 628 54.254 64.496 286.643 1.00 0.61
C
ATOM 4438 O LYS A 628 54.322 65.719 286.661 1.00 0.61
O
ATOM 4439 CB LYS A 628 52.222 63.426 287.640 1.00 0.61
C
ATOM 4440 CG LYS A 628 52.020 64.575 288.642 1.00 0.61
C
ATOM 4441 CD LYS A 628 51.350 64.101 289.944 1.00 0.61
C
ATOM 4442 CE LYS A 628 51.212 65.231 290.971 1.00 0.61
C
ATOM 4443 NZ LYS A 628 50.536 64.749 292.197 1.00 0.61
N
ATOM 4444 N GLN A 629 55.322 63.738 286.959 1.00 0.40
N
ATOM 4445 CA GLN A 629 56.568 64.339 287.397 1.00 0.40
C
ATOM 4446 C GLN A 629 57.502 64.693 286.254 1.00 0.40
C
ATOM 4447 O GLN A 629 58.454 65.444 286.424 1.00 0.40
O
ATOM 4448 CB GLN A 629 57.310 63.370 288.348 1.00 0.40
C

ATOM 4449 CG GLN A 629 56.576 63.099 289.683 1.00 0.40
C
ATOM 4450 CD GLN A 629 56.402 64.378 290.504 1.00 0.40
C
ATOM 4451 OE1 GLN A 629 57.352 65.055 290.884 1.00 0.40
O
ATOM 4452 NE2 GLN A 629 55.132 64.735 290.812 1.00 0.40
N
ATOM 4453 N GLY A 630 57.257 64.163 285.040 1.00 0.73
N
ATOM 4454 CA GLY A 630 58.091 64.440 283.880 1.00 0.73
C
ATOM 4455 C GLY A 630 59.353 63.614 283.801 1.00 0.73
C
ATOM 4456 O GLY A 630 60.080 63.689 282.813 1.00 0.73
O
ATOM 4457 N ASP A 631 59.591 62.752 284.802 1.00 0.63
N
ATOM 4458 CA ASP A 631 60.751 61.905 284.921 1.00 0.63
C
ATOM 4459 C ASP A 631 60.399 60.450 284.621 1.00 0.63
C
ATOM 4460 O ASP A 631 60.256 59.606 285.504 1.00 0.63
O
ATOM 4461 CB ASP A 631 61.359 62.083 286.330 1.00 0.63
C
ATOM 4462 CG ASP A 631 62.795 61.582 286.386 1.00 0.63
C
ATOM 4463 OD1 ASP A 631 63.326 61.495 287.522 1.00 0.63
O
ATOM 4464 OD2 ASP A 631 63.376 61.306 285.304 1.00 0.63
O
ATOM 4465 N ILE A 632 60.250 60.142 283.314 1.00 0.56
N
ATOM 4466 CA ILE A 632 60.058 58.800 282.765 1.00 0.56
C
ATOM 4467 C ILE A 632 58.968 57.969 283.477 1.00 0.56
C
ATOM 4468 O ILE A 632 57.857 58.459 283.641 1.00 0.56
O
ATOM 4469 CB ILE A 632 61.405 58.097 282.509 1.00 0.56
C
ATOM 4470 CG1 ILE A 632 62.451 59.134 282.004 1.00 0.56
C
ATOM 4471 CG2 ILE A 632 61.257 56.972 281.456 1.00 0.56
C
ATOM 4472 CD1 ILE A 632 63.824 58.562 281.628 1.00 0.56
C
ATOM 4473 N ILE A 633 59.236 56.694 283.853 1.00 0.56
N
ATOM 4474 CA ILE A 633 58.325 55.705 284.459 1.00 0.56
C
ATOM 4475 C ILE A 633 57.784 54.714 283.426 1.00 0.56
C

ATOM	4476	O	ILE	A	633	57.358	53.611	283.759	1.00	0.56	O
ATOM	4477	CB	ILE	A	633	57.249	56.259	285.423	1.00	0.56	C
ATOM	4478	CG1	ILE	A	633	57.920	56.926	286.649	1.00	0.56	C
ATOM	4479	CG2	ILE	A	633	56.229	55.195	285.890	1.00	0.56	C
ATOM	4480	CD1	ILE	A	633	56.993	57.796	287.512	1.00	0.56	C
ATOM	4481	N	GLY	A	634	57.889	55.014	282.116	1.00	0.66	N
ATOM	4482	CA	GLY	A	634	57.525	54.039	281.092	1.00	0.66	C
ATOM	4483	C	GLY	A	634	58.308	54.188	279.810	1.00	0.66	C
ATOM	4484	O	GLY	A	634	58.392	55.270	279.236	1.00	0.66	O
ATOM	4485	N	VAL	A	635	58.879	53.073	279.305	1.00	0.80	N
ATOM	4486	CA	VAL	A	635	59.569	52.991	278.021	1.00	0.80	C
ATOM	4487	C	VAL	A	635	58.908	51.921	277.166	1.00	0.80	C
ATOM	4488	O	VAL	A	635	58.862	50.748	277.535	1.00	0.80	O
ATOM	4489	CB	VAL	A	635	61.044	52.606	278.172	1.00	0.80	C
ATOM	4490	CG1	VAL	A	635	61.728	52.351	276.810	1.00	0.80	C
ATOM	4491	CG2	VAL	A	635	61.814	53.706	278.925	1.00	0.80	C
ATOM	4492	N	ASN	A	636	58.395	52.279	275.968	1.00	0.81	N
ATOM	4493	CA	ASN	A	636	57.871	51.285	275.046	1.00	0.81	C
ATOM	4494	C	ASN	A	636	58.995	50.815	274.140	1.00	0.81	C
ATOM	4495	O	ASN	A	636	59.252	51.412	273.095	1.00	0.81	O
ATOM	4496	CB	ASN	A	636	56.729	51.827	274.143	1.00	0.81	C
ATOM	4497	CG	ASN	A	636	55.457	52.053	274.949	1.00	0.81	C
ATOM	4498	OD1	ASN	A	636	55.020	51.177	275.688	1.00	0.81	O
ATOM	4499	ND2	ASN	A	636	54.804	53.225	274.758	1.00	0.81	N
ATOM	4500	N	HIS	A	637	59.678	49.708	274.509	1.00	0.83	N
ATOM	4501	CA	HIS	A	637	60.911	49.248	273.872	1.00	0.83	C
ATOM	4502	C	HIS	A	637	60.841	49.058	272.366	1.00	0.83	C

ATOM 4503 O HIS A 637 61.749 49.443 271.642 1.00 0.83
O
ATOM 4504 CB HIS A 637 61.427 47.937 274.512 1.00 0.83
C
ATOM 4505 CG HIS A 637 61.617 48.069 275.985 1.00 0.83
C
ATOM 4506 ND1 HIS A 637 62.524 49.007 276.409 1.00 0.83
N
ATOM 4507 CD2 HIS A 637 61.028 47.464 277.048 1.00 0.83
C
ATOM 4508 CE1 HIS A 637 62.484 48.968 277.725 1.00 0.83
C
ATOM 4509 NE2 HIS A 637 61.588 48.048 278.166 1.00 0.83
N
ATOM 4510 N SER A 638 59.732 48.515 271.829 1.00 0.86
N
ATOM 4511 CA SER A 638 59.513 48.378 270.391 1.00 0.86
C
ATOM 4512 C SER A 638 59.506 49.692 269.624 1.00 0.86
C
ATOM 4513 O SER A 638 60.018 49.784 268.512 1.00 0.86
O
ATOM 4514 CB SER A 638 58.191 47.632 270.070 1.00 0.86
C
ATOM 4515 OG SER A 638 57.070 48.268 270.691 1.00 0.86
O
ATOM 4516 N ARG A 639 58.936 50.759 270.204 1.00 0.79
N
ATOM 4517 CA ARG A 639 58.938 52.079 269.612 1.00 0.79
C
ATOM 4518 C ARG A 639 60.289 52.755 269.627 1.00 0.79
C
ATOM 4519 O ARG A 639 60.647 53.468 268.694 1.00 0.79
O
ATOM 4520 CB ARG A 639 57.875 52.963 270.265 1.00 0.79
C
ATOM 4521 CG ARG A 639 56.469 52.427 269.963 1.00 0.79
C
ATOM 4522 CD ARG A 639 55.427 53.518 270.119 1.00 0.79
C
ATOM 4523 NE ARG A 639 54.112 52.934 269.736 1.00 0.79
N
ATOM 4524 CZ ARG A 639 52.957 53.240 270.329 1.00 0.79
C
ATOM 4525 NH1 ARG A 639 52.904 54.094 271.351 1.00 0.79
N
ATOM 4526 NH2 ARG A 639 51.844 52.687 269.853 1.00 0.79
N
ATOM 4527 N ILE A 640 61.105 52.488 270.664 1.00 0.86
N
ATOM 4528 CA ILE A 640 62.510 52.870 270.708 1.00 0.86
C
ATOM 4529 C ILE A 640 63.275 52.246 269.540 1.00 0.86
C

```
ATOM   4530  O   ILE A 640      64.083  52.890 268.876  1.00  0.86
O
ATOM   4531  CB  ILE A 640      63.130  52.462 272.042  1.00  0.86
C
ATOM   4532  CG1 ILE A 640      62.455  53.129 273.266  1.00  0.86
C
ATOM   4533  CG2 ILE A 640      64.649  52.692 272.073  1.00  0.86
C
ATOM   4534  CD1 ILE A 640      62.615  54.650 273.354  1.00  0.86
C
ATOM   4535  N   LEU A 641      62.980  50.971 269.219  1.00  0.86
N
ATOM   4536  CA  LEU A 641      63.566  50.261 268.101  1.00  0.86
C
ATOM   4537  C   LEU A 641      63.205  50.807 266.742  1.00  0.86
C
ATOM   4538  O   LEU A 641      64.040  50.851 265.846  1.00  0.86
O
ATOM   4539  CB  LEU A 641      63.227  48.762 268.141  1.00  0.86
C
ATOM   4540  CG  LEU A 641      63.744  48.031 269.385  1.00  0.86
C
ATOM   4541  CD1 LEU A 641      63.698  46.521 269.124  1.00  0.86
C
ATOM   4542  CD2 LEU A 641      65.123  48.559 269.813  1.00  0.86
C
ATOM   4543  N   LEU A 642      61.957  51.274 266.550  1.00  0.88
N
ATOM   4544  CA  LEU A 642      61.564  51.976 265.341  1.00  0.88
C
ATOM   4545  C   LEU A 642      62.400  53.226 265.108  1.00  0.88
C
ATOM   4546  O   LEU A 642      62.826  53.491 263.987  1.00  0.88
O
ATOM   4547  CB  LEU A 642      60.061  52.324 265.368  1.00  0.88
C
ATOM   4548  CG  LEU A 642      59.137  51.124 265.079  1.00  0.88
C
ATOM   4549  CD1 LEU A 642      57.693  51.460 265.472  1.00  0.88
C
ATOM   4550  CD2 LEU A 642      59.192  50.694 263.602  1.00  0.88
C
ATOM   4551  N   CYS A 643      62.724  53.988 266.176  1.00  0.88
N
ATOM   4552  CA  CYS A 643      63.657  55.102 266.082  1.00  0.88
C
ATOM   4553  C   CYS A 643      65.048  54.690 265.631  1.00  0.88
C
ATOM   4554  O   CYS A 643      65.604  55.275 264.703  1.00  0.88
O
ATOM   4555  CB  CYS A 643      63.789  55.868 267.419  1.00  0.88
C
ATOM   4556  SG  CYS A 643      62.168  56.433 268.030  1.00  0.88
S
```

ATOM 4557 N GLU A 644 65.607 53.617 266.219 1.00 0.85
N
ATOM 4558 CA GLU A 644 66.892 53.071 265.817 1.00 0.85
C
ATOM 4559 C GLU A 644 66.922 52.589 264.381 1.00 0.85
C
ATOM 4560 O GLU A 644 67.866 52.840 263.634 1.00 0.85
O
ATOM 4561 CB GLU A 644 67.295 51.900 266.740 1.00 0.85
C
ATOM 4562 CG GLU A 644 67.754 52.372 268.135 1.00 0.85
C
ATOM 4563 CD GLU A 644 69.063 53.157 268.052 1.00 0.85
C
ATOM 4564 OE1 GLU A 644 70.117 52.519 267.796 1.00 0.85
O
ATOM 4565 OE2 GLU A 644 69.014 54.397 268.250 1.00 0.85
O
ATOM 4566 N ALA A 645 65.855 51.904 263.930 1.00 0.92
N
ATOM 4567 CA ALA A 645 65.739 51.414 262.578 1.00 0.92
C
ATOM 4568 C ALA A 645 65.693 52.541 261.565 1.00 0.92
C
ATOM 4569 O ALA A 645 66.370 52.505 260.537 1.00 0.92
O
ATOM 4570 CB ALA A 645 64.518 50.483 262.468 1.00 0.92
C
ATOM 4571 N THR A 646 64.947 53.616 261.883 1.00 0.90
N
ATOM 4572 CA THR A 646 64.959 54.848 261.107 1.00 0.90
C
ATOM 4573 C THR A 646 66.329 55.490 261.039 1.00 0.90
C
ATOM 4574 O THR A 646 66.827 55.805 259.960 1.00 0.90
O
ATOM 4575 CB THR A 646 63.975 55.870 261.661 1.00 0.90
C
ATOM 4576 OG1 THR A 646 62.655 55.356 261.626 1.00 0.90
O
ATOM 4577 CG2 THR A 646 63.936 57.150 260.825 1.00 0.90
C
ATOM 4578 N ALA A 647 67.020 55.646 262.185 1.00 0.93
N
ATOM 4579 CA ALA A 647 68.350 56.216 262.227 1.00 0.93
C
ATOM 4580 C ALA A 647 69.393 55.384 261.485 1.00 0.93
C
ATOM 4581 O ALA A 647 70.233 55.915 260.764 1.00 0.93
O
ATOM 4582 CB ALA A 647 68.782 56.477 263.681 1.00 0.93
C
ATOM 4583 N ALA A 648 69.348 54.046 261.595 1.00 0.93
N

```
ATOM   4584  CA  ALA A 648      70.218  53.159 260.859  1.00  0.93
C
ATOM   4585  C   ALA A 648      70.069  53.272 259.341  1.00  0.93
C
ATOM   4586  O   ALA A 648      71.057  53.375 258.612  1.00  0.93
O
ATOM   4587  CB  ALA A 648      69.957  51.720 261.338  1.00  0.93
C
ATOM   4588  N   VAL A 649      68.820  53.317 258.834  1.00  0.91
N
ATOM   4589  CA  VAL A 649      68.524  53.568 257.429  1.00  0.91
C
ATOM   4590  C   VAL A 649      68.944  54.956 256.954  1.00  0.91
C
ATOM   4591  O   VAL A 649      69.589  55.103 255.913  1.00  0.91
O
ATOM   4592  CB  VAL A 649      67.045  53.309 257.161  1.00  0.91
C
ATOM   4593  CG1 VAL A 649      66.571  53.887 255.814  1.00  0.91
C
ATOM   4594  CG2 VAL A 649      66.809  51.785 257.179  1.00  0.91
C
ATOM   4595  N   LEU A 650      68.652  56.020 257.733  1.00  0.89
N
ATOM   4596  CA  LEU A 650      69.089  57.373 257.428  1.00  0.89
C
ATOM   4597  C   LEU A 650      70.599  57.516 257.400  1.00  0.89
C
ATOM   4598  O   LEU A 650      71.158  58.179 256.534  1.00  0.89
O
ATOM   4599  CB  LEU A 650      68.524  58.394 258.436  1.00  0.89
C
ATOM   4600  CG  LEU A 650      67.008  58.641 258.328  1.00  0.89
C
ATOM   4601  CD1 LEU A 650      66.557  59.530 259.492  1.00  0.89
C
ATOM   4602  CD2 LEU A 650      66.594  59.294 257.001  1.00  0.89
C
ATOM   4603  N   ARG A 651      71.313  56.861 258.332  1.00  0.79
N
ATOM   4604  CA  ARG A 651      72.759  56.866 258.386  1.00  0.79
C
ATOM   4605  C   ARG A 651      73.411  56.274 257.145  1.00  0.79
C
ATOM   4606  O   ARG A 651      74.361  56.842 256.614  1.00  0.79
O
ATOM   4607  CB  ARG A 651      73.224  56.135 259.668  1.00  0.79
C
ATOM   4608  CG  ARG A 651      74.706  56.339 260.031  1.00  0.79
C
ATOM   4609  CD  ARG A 651      75.093  55.709 261.376  1.00  0.79
C
ATOM   4610  NE  ARG A 651      76.525  56.067 261.644  1.00  0.79
N
```

ATOM 4611 CZ ARG A 651 76.933 57.243 262.147 1.00 0.79
C
ATOM 4612 NH1 ARG A 651 78.241 57.488 262.229 1.00 0.79
N
ATOM 4613 NH2 ARG A 651 76.087 58.178 262.568 1.00 0.79
N
ATOM 4614 N GLN A 652 72.886 55.146 256.617 1.00 0.83
N
ATOM 4615 CA GLN A 652 73.327 54.611 255.339 1.00 0.83
C
ATOM 4616 C GLN A 652 72.996 55.514 254.167 1.00 0.83
C
ATOM 4617 O GLN A 652 73.832 55.753 253.302 1.00 0.83
O
ATOM 4618 CB GLN A 652 72.751 53.206 255.064 1.00 0.83
C
ATOM 4619 CG GLN A 652 73.260 52.121 256.041 1.00 0.83
C
ATOM 4620 CD GLN A 652 74.765 51.853 255.933 1.00 0.83
C
ATOM 4621 OE1 GLN A 652 75.481 52.333 255.060 1.00 0.83
O
ATOM 4622 NE2 GLN A 652 75.281 51.033 256.880 1.00 0.83
N
ATOM 4623 N CYS A 653 71.773 56.090 254.141 1.00 0.88
N
ATOM 4624 CA CYS A 653 71.351 57.043 253.125 1.00 0.88
C
ATOM 4625 C CYS A 653 72.269 58.257 253.061 1.00 0.88
C
ATOM 4626 O CYS A 653 72.753 58.626 251.995 1.00 0.88
O
ATOM 4627 CB CYS A 653 69.884 57.477 253.411 1.00 0.88
C
ATOM 4628 SG CYS A 653 69.207 58.828 252.377 1.00 0.88
S
ATOM 4629 N PHE A 654 72.607 58.852 254.220 1.00 0.86
N
ATOM 4630 CA PHE A 654 73.601 59.898 254.327 1.00 0.86
C
ATOM 4631 C PHE A 654 74.991 59.455 253.904 1.00 0.86
C
ATOM 4632 O PHE A 654 75.665 60.150 253.147 1.00 0.86
O
ATOM 4633 CB PHE A 654 73.678 60.409 255.787 1.00 0.86
C
ATOM 4634 CG PHE A 654 72.438 61.132 256.240 1.00 0.86
C
ATOM 4635 CD1 PHE A 654 71.650 61.932 255.392 1.00 0.86
C
ATOM 4636 CD2 PHE A 654 72.082 61.043 257.593 1.00 0.86
C
ATOM 4637 CE1 PHE A 654 70.546 62.635 255.892 1.00 0.86
C

ATOM 4638 CE2 PHE A 654 70.981 61.739 258.095 1.00 0.86
C
ATOM 4639 CZ PHE A 654 70.214 62.540 257.248 1.00 0.86
C
ATOM 4640 N TYR A 655 75.438 58.259 254.338 1.00 0.86
N
ATOM 4641 CA TYR A 655 76.745 57.720 254.016 1.00 0.86
C
ATOM 4642 C TYR A 655 76.949 57.547 252.519 1.00 0.86
C
ATOM 4643 O TYR A 655 77.989 57.948 251.995 1.00 0.86
O
ATOM 4644 CB TYR A 655 76.972 56.376 254.766 1.00 0.86
C
ATOM 4645 CG TYR A 655 78.335 55.787 254.506 1.00 0.86
C
ATOM 4646 CD1 TYR A 655 78.485 54.737 253.583 1.00 0.86
C
ATOM 4647 CD2 TYR A 655 79.475 56.301 255.145 1.00 0.86
C
ATOM 4648 CE1 TYR A 655 79.755 54.223 253.291 1.00 0.86
C
ATOM 4649 CE2 TYR A 655 80.747 55.781 254.857 1.00 0.86
C
ATOM 4650 CZ TYR A 655 80.884 54.745 253.927 1.00 0.86
C
ATOM 4651 OH TYR A 655 82.153 54.215 253.628 1.00 0.86
O
ATOM 4652 N ILE A 656 75.939 57.014 251.797 1.00 0.88
N
ATOM 4653 CA ILE A 656 75.952 56.870 250.347 1.00 0.88
C
ATOM 4654 C ILE A 656 76.101 58.217 249.659 1.00 0.88
C
ATOM 4655 O ILE A 656 76.935 58.379 248.779 1.00 0.88
O
ATOM 4656 CB ILE A 656 74.716 56.121 249.851 1.00 0.88
C
ATOM 4657 CG1 ILE A 656 74.778 54.644 250.314 1.00 0.88
C
ATOM 4658 CG2 ILE A 656 74.596 56.203 248.310 1.00 0.88
C
ATOM 4659 CD1 ILE A 656 73.449 53.896 250.150 1.00 0.88
C
ATOM 4660 N LEU A 657 75.372 59.250 250.122 1.00 0.89
N
ATOM 4661 CA LEU A 657 75.450 60.598 249.588 1.00 0.89
C
ATOM 4662 C LEU A 657 76.733 61.348 249.958 1.00 0.89
C
ATOM 4663 O LEU A 657 76.996 62.447 249.470 1.00 0.89
O
ATOM 4664 CB LEU A 657 74.234 61.426 250.057 1.00 0.89
C

ATOM 4665 CG LEU A 657 72.856 60.887 249.627 1.00 0.89
C
ATOM 4666 CD1 LEU A 657 71.763 61.660 250.378 1.00 0.89
C
ATOM 4667 CD2 LEU A 657 72.613 60.959 248.114 1.00 0.89
C
ATOM 4668 N GLY A 658 77.579 60.790 250.854 1.00 0.91
N
ATOM 4669 CA GLY A 658 78.792 61.451 251.320 1.00 0.91
C
ATOM 4670 C GLY A 658 78.543 62.414 252.444 1.00 0.91
C
ATOM 4671 O GLY A 658 79.382 63.255 252.764 1.00 0.91
O
ATOM 4672 N LEU A 659 77.365 62.339 253.068 1.00 0.86
N
ATOM 4673 CA LEU A 659 76.964 63.196 254.154 1.00 0.86
C
ATOM 4674 C LEU A 659 77.335 62.555 255.469 1.00 0.86
C
ATOM 4675 O LEU A 659 77.207 61.348 255.665 1.00 0.86
O
ATOM 4676 CB LEU A 659 75.445 63.458 254.142 1.00 0.86
C
ATOM 4677 CG LEU A 659 74.900 63.905 252.779 1.00 0.86
C
ATOM 4678 CD1 LEU A 659 73.391 64.118 252.831 1.00 0.86
C
ATOM 4679 CD2 LEU A 659 75.530 65.183 252.252 1.00 0.86
C
ATOM 4680 N LYS A 660 77.836 63.357 256.420 1.00 0.79
N
ATOM 4681 CA LYS A 660 78.410 62.815 257.622 1.00 0.79
C
ATOM 4682 C LYS A 660 77.538 63.206 258.783 1.00 0.79
C
ATOM 4683 O LYS A 660 77.568 64.370 259.112 1.00 0.79
O
ATOM 4684 CB LYS A 660 79.860 63.329 257.765 1.00 0.79
C
ATOM 4685 CG LYS A 660 80.815 62.379 258.501 1.00 0.79
C
ATOM 4686 CD LYS A 660 82.252 62.923 258.456 1.00 0.79
C
ATOM 4687 CE LYS A 660 83.283 61.967 259.062 1.00 0.79
C
ATOM 4688 NZ LYS A 660 84.280 62.714 259.861 1.00 0.79
N
ATOM 4689 N PRO A 661 76.757 62.360 259.430 1.00 0.82
N
ATOM 4690 CA PRO A 661 75.779 62.835 260.403 1.00 0.82
C
ATOM 4691 C PRO A 661 76.360 62.939 261.798 1.00 0.82
C

ATOM 4692 O PRO A 661 77.041 62.024 262.266 1.00 0.82
O
ATOM 4693 CB PRO A 661 74.664 61.781 260.344 1.00 0.82
C
ATOM 4694 CG PRO A 661 75.360 60.520 259.843 1.00 0.82
C
ATOM 4695 CD PRO A 661 76.350 61.092 258.850 1.00 0.82
C
ATOM 4696 N VAL A 662 76.085 64.057 262.491 1.00 0.81
N
ATOM 4697 CA VAL A 662 76.484 64.250 263.871 1.00 0.81
C
ATOM 4698 C VAL A 662 75.450 63.703 264.834 1.00 0.81
C
ATOM 4699 O VAL A 662 74.304 63.431 264.484 1.00 0.81
O
ATOM 4700 CB VAL A 662 76.785 65.699 264.221 1.00 0.81
C
ATOM 4701 CG1 VAL A 662 77.969 66.166 263.365 1.00 0.81
C
ATOM 4702 CG2 VAL A 662 75.560 66.607 263.999 1.00 0.81
C
ATOM 4703 N SER A 663 75.857 63.482 266.098 1.00 0.79
N
ATOM 4704 CA SER A 663 74.988 62.935 267.126 1.00 0.79
C
ATOM 4705 C SER A 663 74.193 63.971 267.902 1.00 0.79
C
ATOM 4706 O SER A 663 73.057 63.713 268.300 1.00 0.79
O
ATOM 4707 CB SER A 663 75.796 62.062 268.121 1.00 0.79
C
ATOM 4708 OG SER A 663 76.864 62.787 268.737 1.00 0.79
O
ATOM 4709 N LYS A 664 74.771 65.165 268.154 1.00 0.72
N
ATOM 4710 CA LYS A 664 74.161 66.172 269.004 1.00 0.72
C
ATOM 4711 C LYS A 664 73.605 67.369 268.246 1.00 0.72
C
ATOM 4712 O LYS A 664 72.384 67.498 268.172 1.00 0.72
O
ATOM 4713 CB LYS A 664 75.141 66.613 270.118 1.00 0.72
C
ATOM 4714 CG LYS A 664 75.472 65.456 271.077 1.00 0.72
C
ATOM 4715 CD LYS A 664 76.368 65.877 272.250 1.00 0.72
C
ATOM 4716 CE LYS A 664 76.705 64.697 273.164 1.00 0.72
C
ATOM 4717 NZ LYS A 664 77.615 65.124 274.248 1.00 0.72
N
ATOM 4718 N MET A 665 74.483 68.236 267.688 1.00 0.70
N

```
ATOM   4719  CA  MET A 665      74.104  69.467 267.005  1.00  0.70
C
ATOM   4720  C   MET A 665      73.589  70.585 267.968  1.00  0.70
C
ATOM   4721  O   MET A 665      73.395  70.317 269.187  1.00  0.70
O
ATOM   4722  CB  MET A 665      73.153  69.164 265.815  1.00  0.70
C
ATOM   4723  CG  MET A 665      72.896  70.273 264.787  1.00  0.70
C
ATOM   4724  SD  MET A 665      72.046  69.598 263.325  1.00  0.70
S
ATOM   4725  CE  MET A 665      71.322  71.135 262.691  1.00  0.70
C
ATOM   4726  OXT MET A 665      73.432  71.741 267.490  1.00  0.70
O
TER    4727      MET A 665
HETATM 4728  N   ARG _   1      89.271  66.972 232.482  1.00 33.91
N
HETATM 4729  CA  ARG _   1      88.059  66.978 231.654  1.00 33.78
C
HETATM 4730  C   ARG _   1      87.384  68.346 231.701  1.00 36.91
C
HETATM 4731  O   ARG _   1      86.362  68.554 231.055  1.00 42.10
O
HETATM 4732  CB  ARG _   1      87.060  65.890 232.091  1.00 27.08
C
HETATM 4733  CG  ARG _   1      87.493  64.455 231.809  1.00 26.90
C
HETATM 4734  CD  ARG _   1      86.358  63.510 232.091  1.00 26.47
C
HETATM 4735  NE  ARG _   1      86.658  62.090 231.877  1.00 28.20
N
HETATM 4736  CZ  ARG _   1      86.913  61.538 230.687  1.00 27.45
C
HETATM 4737  NH1 ARG _   1      87.137  60.238 230.581  1.00 24.94
N
HETATM 4738  NH2 ARG _   1      86.958  62.287 229.600  1.00 29.92
N
HETATM 4739  OXT ARG _   1      87.825  69.285 232.367  1.00 35.89
O
TER    4740      ARG _   1
CONECT 4728 4729
CONECT 4729 4728 4730 4732
CONECT 4730 4729 4731 4739
CONECT 4731 4730
CONECT 4732 4729 4733
CONECT 4733 4732 4734
CONECT 4734 4733 4735
CONECT 4735 4734 4736
CONECT 4736 4735 4737 4738
CONECT 4737 4736
CONECT 4738 4736
CONECT 4739 4730
```

END
